# PICA: Pixel Intensity Correlation Analysis for Deconvolution and Metabolite Identification in Mass Spectrometry Imaging

**DOI:** 10.1021/acs.analchem.2c04778

**Published:** 2023-01-03

**Authors:** Yonghui Dong, Nir Shachaf, Liron Feldberg, Ilana Rogachev, Uwe Heinig, Asaph Aharoni

**Affiliations:** † Department of Plant Sciences, Weizmann Institute of Science, Rehovot7610001, Israel; ‡ Department of Life Sciences Core Facilities, Weizmann Institute of Science, Rehovot7610001, Israel; § Department of Analytical Chemistry, Israel Institute for Biological Research, Ness Ziona7410001, Israel

## Abstract

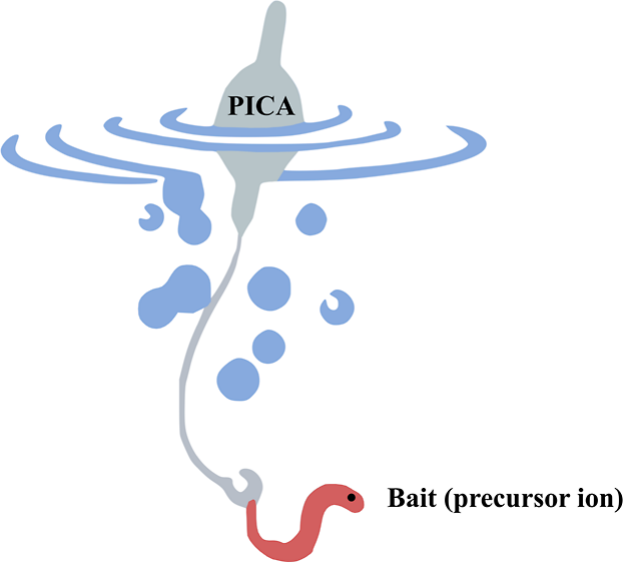

In-source fragmentation (ISF) is a naturally occurring phenomenon in various ion sources including soft ionization techniques such as matrix-assisted laser desorption/ionization (MALDI). It has traditionally been minimized as it makes the dataset more complex and often leads to mis-annotation of metabolites. Here, we introduce an approach termed PICA (for pixel intensity correlation analysis) that takes advantage of ISF in MALDI imaging to increase confidence in metabolite identification. In PICA, the extraction and association of in-source fragments to their precursor ion results in “pseudo-MS/MS spectra” that can be used for identification. We examined PICA using three different datasets, two of which were published previously and included validated metabolites annotation. We show that highly colocalized ions possessing Pearson correlation coefficient (PCC) ≥ 0.9 for a given precursor ion are mainly its in-source fragments, natural isotopes, adduct ions, or multimers. These ions provide rich information for their precursor ion identification. In addition, our results show that moderately colocalized ions (PCC < 0.9) may be structurally related to the precursor ion, which allows for the identification of unknown metabolites through known ones. Finally, we propose three strategies to reduce the total computation time for PICA in MALDI imaging. To conclude, PICA provides an efficient approach to extract and group ions stemming from the same metabolites in MALDI imaging and thus allows for high-confidence metabolite identification.

## Introduction

Mass spectrometry imaging (MSI) is a powerful analytical tool that allows for mapping the spatial distribution of a broad range of metabolites directly from a sample tissue.^1−3^ However, one major limitation associated with MSI is that metabolite identification is challenging.^4,5^ Although the use of ultra-high-mass-resolution mass spectrometry (MS) in MSI experiments, such as Fourier transform ion cyclotron resonance, allows for direct determination of the elemental composition for metabolites, it does not provide high confidence and unambiguous identification.^4,6,7^ Current methods for metabolite identification in MSI rely heavily on additional workflows for either manual on-tissue MS/MS of each ion of interest, or liquid chromatography (LC)–MS/MS analysis of a tissue homogenate, both of which require extra steps and are often problematic in case of limited sample quantities.^6^ Although several tandem (MS/MS) MSI approaches have been proposed,^8−11^ they are limited in precursor coverage and lack automated interpretation of the resulting complex datasets, therefore hampering untargeted applications of MSI.

In-source fragmentation (ISF) is a naturally occurring phenomenon during electrospray ionization (ESI) in LC–MS analysis.^12,13^ Despite the “soft nature” of ESI, it has been reported that more than 80% of metabolites in the METLIN database could readily dissociate at low collision energy, implying that ISF widely exists in ESI.^14^ Because in-source fragments can be easily mis-annotated as molecular ions, and they typically increase the data complexity, efforts have been made to minimize or eliminate ISF by adjusting in-source parameters.^15^ However, in recent years, the value of ISF is increasingly recognized in LC-ESI-MS analysis as ISF and low-energy MS/MS fragmentation are similar, and ISF can hence be used for metabolite identification.^15−17^

Although matrix-assisted laser desorption/ionization (MALDI) has been generally considered a “soft ionization” technique, it is also known that ISF widely exists in MALDI.^18−20^ In particular, the use of 1,5-diaminonaphthalene and 2,5-dihydroxybenzoic acid (DHB), the two most common MALDI matrices,^21,22^ have been reported to enhance ISF in MALDI experiments.^22,23^ Apart from MALDI, two other commonly used ion sources in MSI, that is, desorption electrospray ionization (DESI) and secondary ion mass spectrometry (SIMS), also have different degrees of ISF. DESI has been described as following an ESI-like ionization mechanism,^23,24^ implying that the extent of ISF in DESI is similar to ESI. The use of high-energy primary ion beams undoubtedly leads to extensive ISF in SIMS.^25^ Metabolite identification in MSI could also benefit from ISF. However, unlike LC–MS, the lack of chromatographic separation in MSI makes it practically challenging to extract, interpret, and assign the in-source fragments to their precursor ions. In this study, we used pixel intensity correlation analysis (PICA) to cluster ions stemming from the same precursor or different ions with similar substructures (i.e., ions sharing the same in-source fragments). These clustered ions provide valuable information on multimers, adducts, natural isotopes, and in-source fragments, which significantly facilitates metabolite identification in MSI experiments.

## Experimental Section

### Test Datasets

Three datasets were used in this study for MALDI analysis: (i) WT tomato fruit (*Solanum lycopersicum*, cultivar Microtom) grown in a climate-controlled greenhouse [24/18 °C (day/night) at natural day length conditions] collected at the mature green stage (ca. 30 days after anthesis); (ii) transgenic tomato fruit partially accumulating anthocyanins at the red ripe stage (ca. 45 days after anthesis).^26^ Sample preparation and data acquisition methods were the same for (i) and (ii). Fresh fruit was embedded with M1 embedding matrix (Thermo Scientific, Waltham, MA) in Peel-A-Way disposable embedding molds (Peel-A-Way Scientific, South El Monte, California) and flash-frozen in liquid nitrogen. The embedded fruit was transferred to a cryostat (Leica CM3050) and allowed to thermally equilibrate at −18 °C for at least 2 h. The frozen fruit was then cut into 35-μm-thick sections. The sections were thaw-mounted onto Superfrost Plus slides (Fisher Scientific, Pittsburg, PA) and vacuum-dried in a desiccator. An HTX TM sprayer (HTX Technologies, USA) was used to coat slides with a DHB matrix (40 mg mL^–1^ in water/methanol, v/v 30/70, containing 0.2% trifluoroacetic acid), nozzle temperature was set at 70 °C, and a DHB matrix solution sprayed 16 passes over the tissue sections at a linear velocity of 120 cm min^–1^ with a flow rate of 50 μL min^–1^. MSI data were collected in positive ion mode using a 7T Solarix FT-ICR-MS system (Bruker Daltonics, Bremen, Germany) using lock mass calibration (DHB matrix peak: [3DHB + H – 3H_2_O]^+^, *m*/*z* 409.0554) at a frequency of 1 kHz and a laser power of 18%–22%, with 100 laser shots per pixel. Each mass spectrum was recorded at a mass range of 150–3000 Da in the broadband mode and with a time domain for acquisition of 1 M (mega-word), providing an estimated resolving power of 115,000 at 400 *m*/*z*. The acquired raw data (.mis) were converted to imzML format using FlexImaging software (V 4.1, Bruker Daltonics, Germany). (iii) Data were downloaded from EMBL-EBI MetaboLights (https://ebi.ac.uk/metabolights/MTBLS487). This dataset was acquired on a wild-type mouse cerebellum section. The sample section was coated with DHB matrix (10 mg mL^–1^), and analyzed in a positive ion mode over a mass range of 300–2000 Da (MALDI LTQ Orbitrap XL instrument; 50 μm spatial resolution; see also^27^).

### Data Analysis

Data preprocessing, including total ion current (TIC) normalization, peak picking, and peak alignment, was performed using the R package Cardinal.^28^ For datasets (i) and (ii), recalibration was implemented to account for any possible mass shifts during peak alignment using several known internal metabolites. PICA was performed using Pearson correlation analysis at each pixel for all the detected mass features, and the top 100 colocalized mass features were selected for further analysis. Colocalized ions with Pearson correlation coefficient (PCC) value ≥ 0.9 were chosen to create the “pseudo MS/MS” using a home-written R script. MALDI images were plotted with the R package Cardinal and mass bin width of ±0.003 Da. Images were optimized with Gaussian smoothing and contrast enhancement. The colocalization network was produced with the R package igraph.^29^ All the R scripts are publicly available on GitHub (https://github.com/YonghuiDong/MSI_Colocalization), and a demo workflow including all data analysis steps using dataset (iii) is provided in Supporting Information S1.

## Results and Discussion

### Rationale and Workflow of Using PICA in Mass Spectrometry Imaging

In fluorescence microscopy, colocalization analysis is widely used to compare the degree of spatial overlaps between two fluorescently labeled molecules.^30^ Colocalization consists of two different phenomena, co-occurrence, which refers to the simple spatial overlap of two molecules, and correlation, in which two molecules overlap and codistribute in proportion to each other.^31,32^ Co-occurrence analysis is often applied to determine what proportion of a molecule is present within the sample area, while it does not provide insights into any intensity relationship between two molecules. By contrast, correlation analysis is mostly used to assess the functional or stoichiometric relationship between two overlapping species.^32^ PCC is a common metric to quantitatively measure colocalization.^32^ The formula for PCC is given below for a typical image consisting of red and green channels.
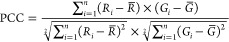
where *R*_*i*_ and *R̅* refer to *i*th pixel intensity and mean intensity of the red channel, respectively. Likewise, *G*_*i*_ and *G̅* are the *i*th pixel intensity and mean intensity of the green channel, respectively. The value n represents the total number of pixels in the image. PCC values range from 1 for two images whose fluorescence intensities are perfectly, linearly related, to −1 for two images, whose fluorescence intensities are perfectly, but inversely, related. Values near 0 indicate that the two images are uncorrelated to each other.

**Figure 1:**
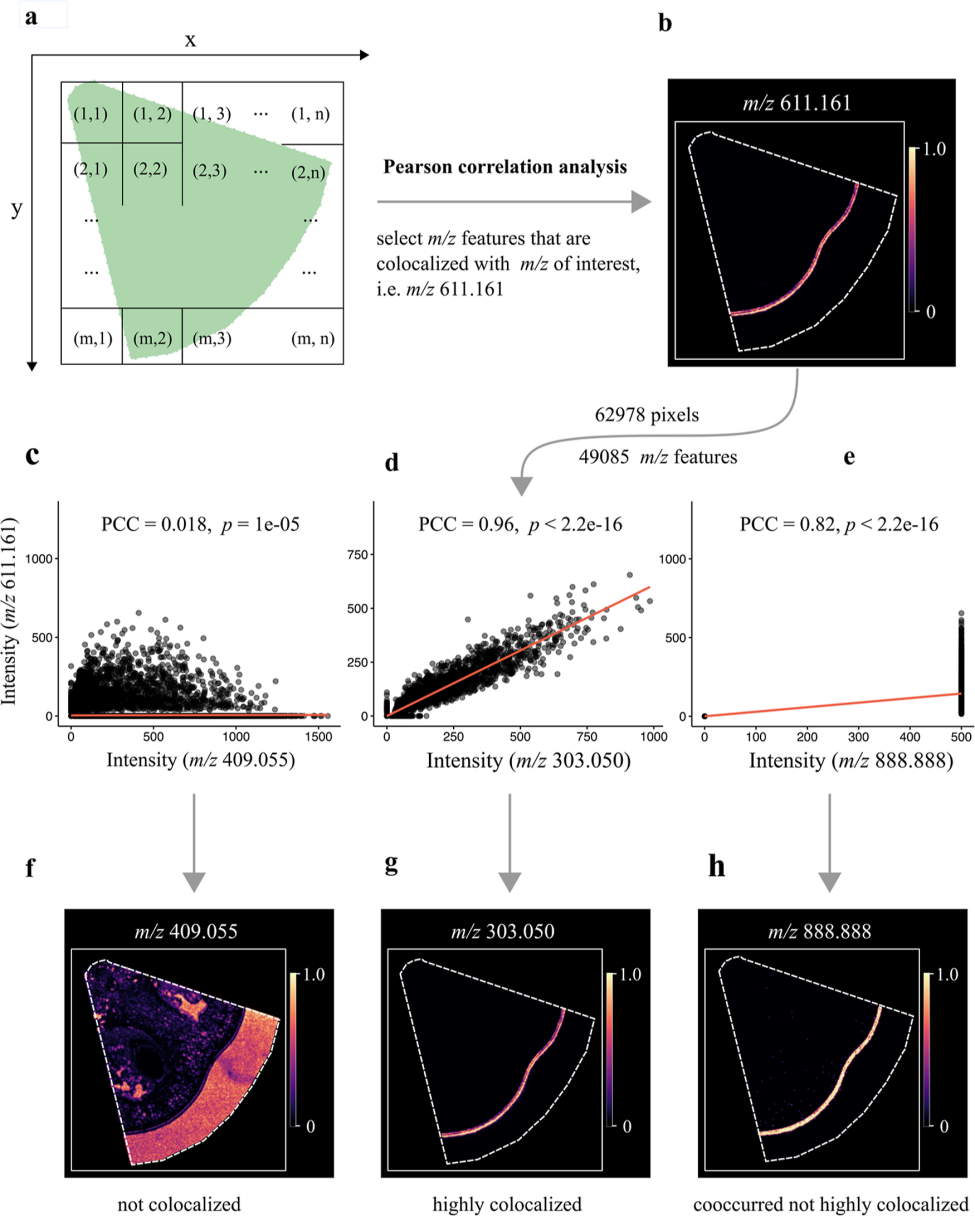
Schematic of the PICA workflow. A “pizza slice-shape” region of a tomato fruit section was analyzed by MALDI imaging. The preprocessed dataset contained 62,978 pixels and 49,085 mass features. Rutin (*m*/*z* 611.161, [M + H]^+^), which is exclusively localized in tomato skin, was used for colocalization analysis. PICA was applied for colocalization analysis and PCC was used to quantify the degree of colocalization with the rutin ion. Colocalization plots of the three ions (*m*/*z* 409.055, *m*/*z* 303.050, and *m*/*z* 888.888) demonstrate the different degrees of colocalization with rutin. The ion *m*/*z* 888.888 is an artificially created ion that has the exact spatial distribution as rutin, but its ion intensity is kept the same across all the pixels. The MALDI images at the bottom part represent the three ions generated using the exact *m*/*z* value with a mass bin width of ±0.003 Da. The images were optimized with Gaussian smoothing and contrast enhancement. The color scale indicates the range of TIC normalized intensity.

MSI allows mapping the distribution of hundreds of metabolites simultaneously. In this regard, MSI can be viewed as a high-throughput multichannel fluorescence microscopy, with each individual ion image being a unique image channel. As such, correlation analysis can be also applied in MSI to quantify the colocalization of different ions. Indeed, colocalization analysis has been applied to cluster mass features and classify sample tissues using colocalized mass features to represent the entire molecular information in MSI.^33,34^Figure 1 presents a general workflow of correlation analysis for MALDI imaging. A “pizza slice-shaped” region of a tomato fruit section was selected and analyzed by MALDI imaging. Following laser ablation, each ablated pixel corresponds to a specific (*x*, *y*) coordinate in the tissue, with a total number of 62,978 pixels analyzed (Figure 1). The raw MSI dataset was then preprocessed with R package Cardinal and in total 49,085 unique mass features (with signal-to-noise ratio, S/N ≥ 6) were selected. Rutin (*m*/*z* 611.161, [M + H]^+^), a core flavonoid in tomato fruit,^35^ was used as an example for colocalization analysis. Three examples are given here to describe the different degrees of colocalization with rutin. The MALDI image shows that rutin accumulates almost exclusively in tomato fruit skin (Figure 1). The PCC between *m*/*z* 611.161 and *m*/*z* 409.055 is 0.018, indicating that the two ions are not colocalized (Figure 1). The MALDI image of *m*/*z* 409.055 confirms that it has a very different distribution as compared to rutin. In contrast, the PCC between *m*/*z* 611.161 and *m*/*z* 303.050 is 0.96 (Figure 1) and the MALDI image also demonstrates that it has the same distribution as rutin (Figure 1). It is important to note that co-occurrence does not always guarantee high correlation. For instance, we have created an artificial mass feature *m*/*z* 888.888, which has the exact spatial distribution as rutin (perfect co-occurrence), except that its ion intensity at each pixel was kept constant (i.e., 500 arbitrary units; Figure 1). The correlation analysis of the two ions showed that their PCC is only 0.82 rather than close to 1 (Figure 1).

### PICA Enables In-source Fragment Extraction and Assignment

In theory, ions that are perfectly colocalized (PCC = 1) to a given precursor ion (or bait) in MSI are likely to be its natural isotope peaks (e.g., ^13^C isotope peaks), in-source fragments, multiply charged ions (e.g., doubly charged ions), multimers (e.g., dimers and trimers), and adduct ions (e.g., Na^+^ and K^+^ adducts) because their ion intensities are proportional to the precursor ion regardless of the abundance and ionization efficiency variations among different pixels. Therefore, it is possible to use PICA to extract, interpret, and assign in-source fragments to their corresponding precursor ions.

**Figure 2:**
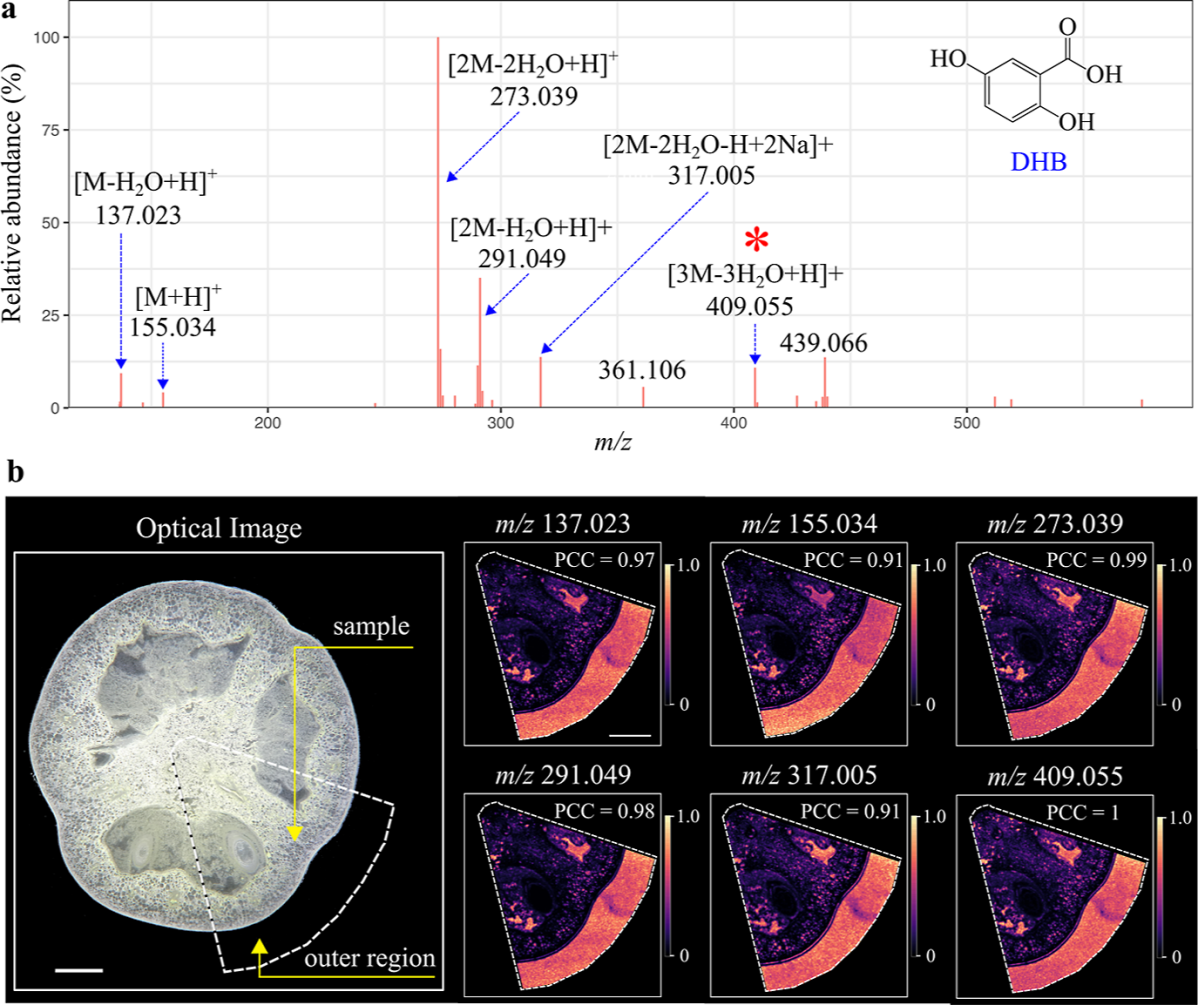
Colocalization analysis result for the DHB matrix peak. (a) The “pseudo-DHB spectrum” was generated by PICA. Twenty-six ions found to be highly colocalized with *m*/*z* 409.055 (PCC ≥ 0.9) were used to construct the “pseudo-DHB spectrum”. The ion intensity of each peak was the mean ion intensity from MALDI imaging. (b) Optical image of the analyzed tomato fruit section, and MALDI images of six representative colocalized ions. MALDI images were generated using the exact *m*/*z* value with a mass bin width of ±0.003 Da. Images were optimized with Gaussian smoothing and contrast enhancement, and the color scale indicates the range of TIC normalized intensity. Scale bar, 2 mm.

**Figure 3:**
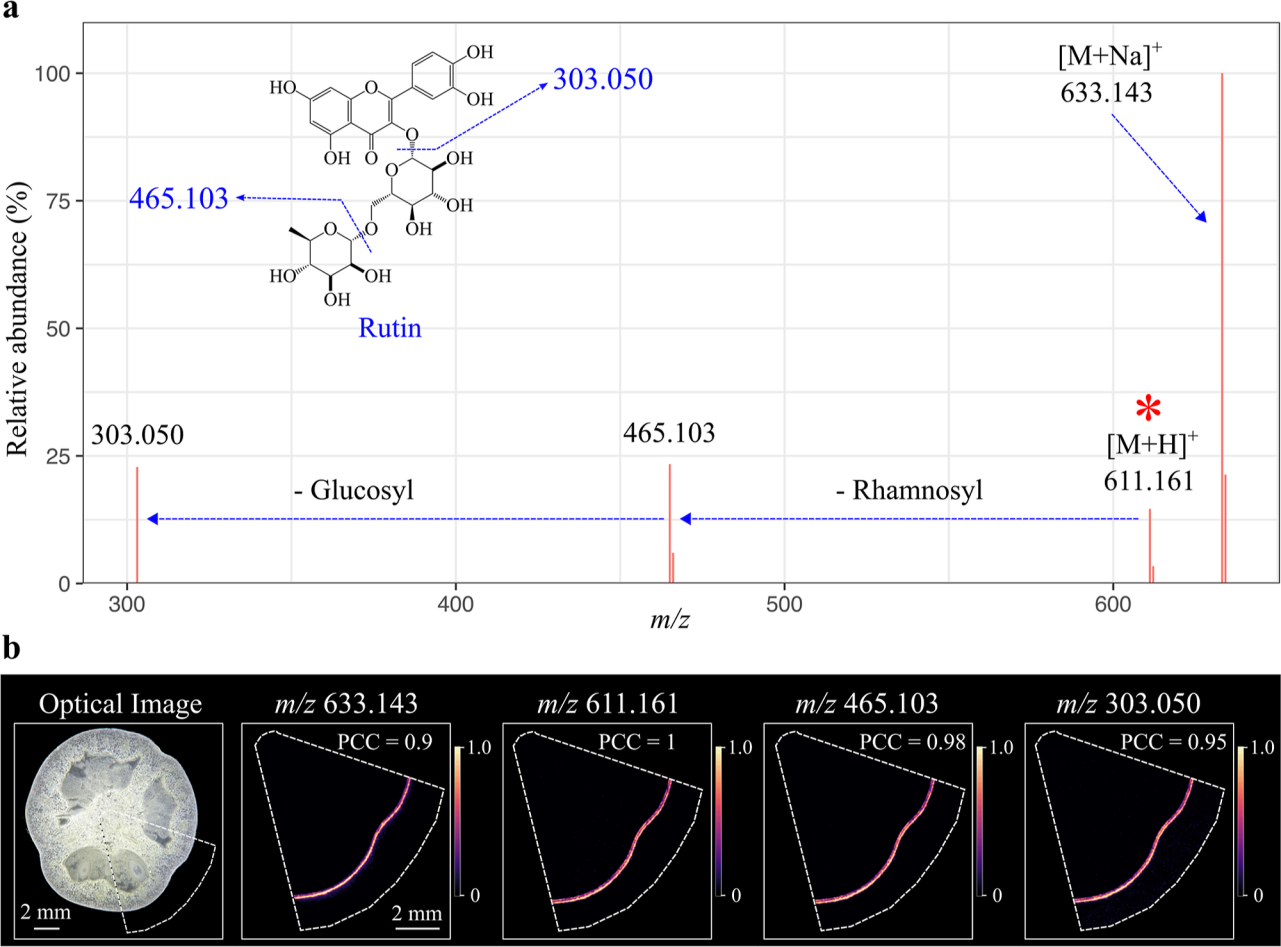
Colocalization analysis of the protonated rutin peak. (a) “Pseudo-rutin MS/MS spectrum” was generated by Pearson correlation analysis. Six ions found highly colocalized with rutin (PCC ≥ 0.9) were used to construct the pseudo MS/MS spectrum. Ion intensity of each peak is the averaged ion intensity from MALDI imaging. (b) Optical image of the analyzed tomato fruit section, and MALDI images of four representative colocalized ions. MALDI images were generated using the exact *m*/*z* value with a mass bin width of ±0.003 Da and optimized with Gaussian smoothing and contrast enhancement. The color scale indicates the range of TIC normalized intensity. Scale bar, 2 mm.

Three datasets were used to test the PICA approach; the first was acquired with tomato fruit at the mature green stage as shown in Figure 1. First, the DHB peak *m*/*z* 409.055 ([3DH – 3H_2_O + H]^+^) was used for colocalization analysis. Of all the 49,085 mass features at 62,978 pixels, 26 mass peaks were found highly colocalized with *m*/*z* 409.055 with PCC ≥ 0.9 (Table S1 and Figure S1). The average ion intensity of the 26 mass peaks across the analyzed sample region was calculated and used to create a “pseudo DHB mass spectrum” (Figure 2a). Most major peaks in the mass spectrum were identified as H^+^ or Na^+^ adducts of different combinations of up to three DHB units (oligomerization). Only two abundant peaks, *m*/*z* 361.106 and *m*/*z* 439.066, remained unidentified; nevertheless, they are likely to be DHB-related ions because they were also detected in fresh, pure DHB matrix, and a previous study has also reported that they were DHB-related ions.^36^ It is important to note that although the DHB matrix was homogeneously coated over the tomato tissue surface, it was unevenly detected in the measured region. For instance, a significant DHB ion intensity reduction was observed in the tomato sample compared to that in the outer sample region (Figure 2b). This phenomenon has been discussed previously.^22,37,38^ The reason is that a large portion of DHB matrix was absorbed in the porous tomato fruit section, leading to the reduced DHB peak intensities over the sample surface. By contrast, the glass slide (outer sample region in Figure 2b) did not absorb any DHB matrix, therefore favoring the detection of DHB ions by MALDI imaging. Nevertheless, the spatial distribution of all 26 extracted ions was determined, confirming that they have the same spatial distribution as *m*/*z* 409.055. The representative MALDI images of six extracted ions are shown in Figure 2b. The same approach was also applied to extract ions that are correlated to the rutin signal. In total, six ions were found highly colocalized with the protonated rutin peak (*m*/*z* 611.161, [M + H]^+^) with PCC ≥ 0.9 (Table S2 and Figure S2), including two major rutin fragments, *m*/*z* 465.103 and *m*/*z* 303.050, one Na^+^ adduct peak *m*/*z* 633.143 and their corresponding natural ^13^C isotope peaks (Figure 3a). The reconstructed “pseudo rutin MS/MS spectrum” is very similar to the spectrum acquired by LC–MS/MS of the rutin standard at low collision energy, allowing retrieval of level-2 putative identification of rutin according to the Metabolomics Standard Initiatives.^39−41^ MALDI images confirm that these ions have identical distribution to the rutin ion; they are all exclusively detected in tomato skin tissues (Figure 3b).

**Figure 4:**
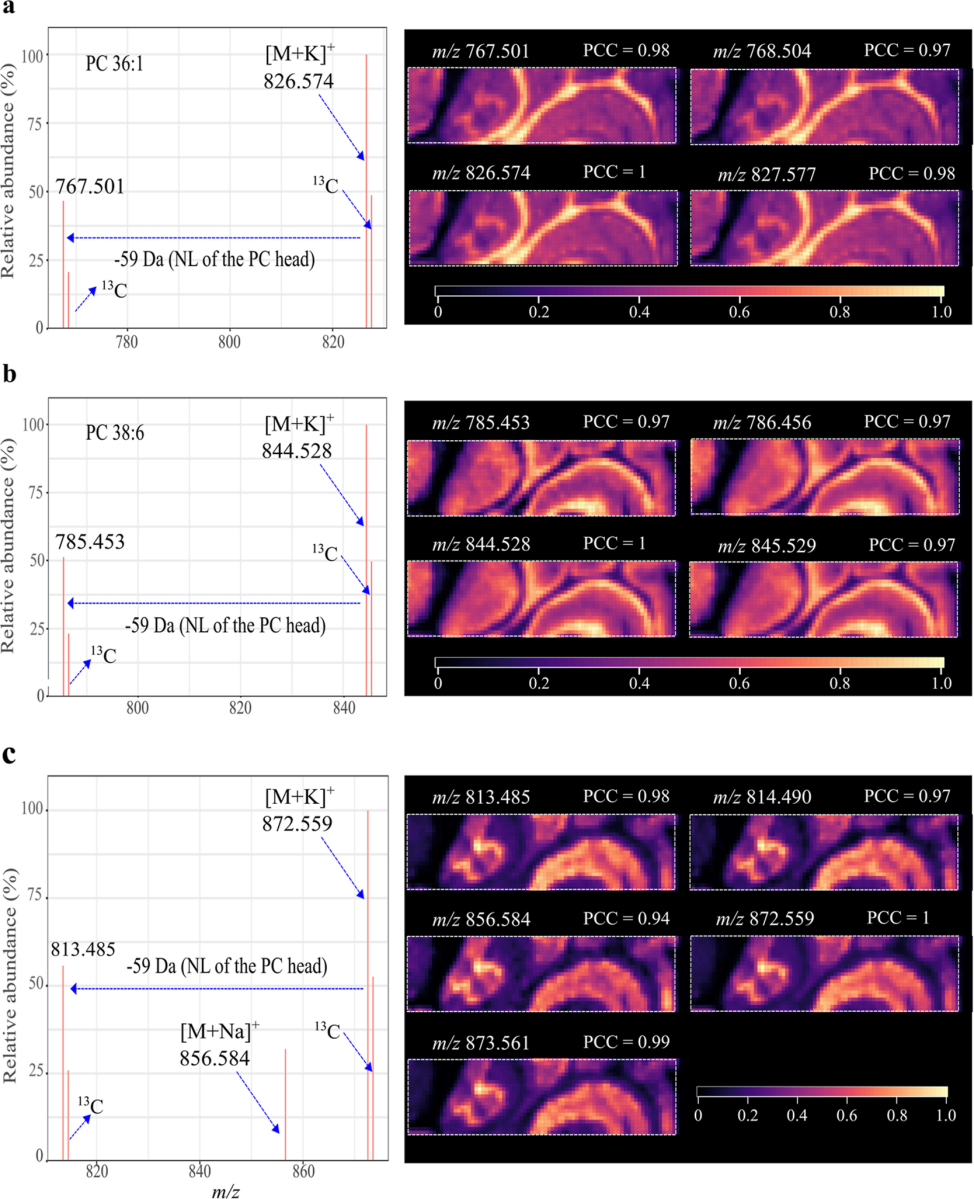
PICA-assisted lipid class identification. Left panels; “pseudo MS/MS spectrum” of three lipid species *m*/*z* 826.574 (a), *m*/*z* 844.528 (b) and *m*/*z* 872.559 (c). The pseudo spectrum was generated with their highly colocalized ions (PCC ≥ 0.9). Ion intensity of each peak is the averaged ion intensity from MALDI imaging. Right panels; MALDI images of colocalized ions for each lipid species. MALDI images were generated using the exact *m*/*z* value with a mass bin width of ±0.003 Da and optimized with Gaussian smoothing and contrast enhancement. The color scale indicates the range of TIC normalized intensity. ^13^C denotes the natural ^13^C isotopic peak. This MALDI imaging data set was obtained from a mouse cerebellum section analyzed by LTQ Orbitrap XL instrument.^27^

To test if PICA can be applied to additional organisms and datasets obtained from other MSI instruments, we downloaded a public dataset from MetaboLights (https://ebi.ac.uk/metabolights/)^42^ acquired on a wild-type mouse cerebellum section. In the corresponding experiment, the sample was analyzed in positive ion mode over a mass range of 300–2000 Da using MALDI LTQ Orbitrap XL.^27^ In their study, the authors detected three ions, *m*/*z* 826.572, *m*/*z* 844.525, and *m*/*z* 872.557, which were predominantly located in the white matter, granular layer, and molecular layer, respectively (Figure S3). The three ions were tentatively identified by the authors as phosphatidylcholines (PC), that is, [PC (36:1) + K]^+^, [PC (38:6) + K]^+^, and [PC (40:6) + K]^+^, based on accurate mass search in an in-house database. However, accurate mass search alone provides poor evidence for metabolite identity, and as the authors stated, this was only the first step toward lipid identification.^27^ Indeed, lipid class isomers are fundamentally indistinguishable based solely on accurate masses. For instance, PC and phosphatidylethanolamines (PE) could share a common elemental composition (PC = PE + 3CH_2_); therefore, the identified lipids PC (36:1), PC (38:6), and PC (40:6) in their study can be also annotated as PE (39:1), PE (41:6), and PE (43:6), respectively. We have therefore performed PICA on these three ions in order to extract their in-source fragments and identify the three ions according to their fragments. The resulting “pseudo-MS/MS spectra” are shown in Figure 4. A neutral loss of 59 Da, which corresponds to the PC head trimethylamine, was detected for all three ions, confirming that they are PC-class lipids (Figure 4). By contrast, a neutral loss of 141 Da, which corresponds to the PE head, would be expected if these ions were PE-class lipids. The MALDI images confirmed that the in-source fragments and their respective precursor ions possess the same spatial distribution (Figure 4).

### PICA can be Used to Identify Moderately Colocalized Ions

In the abovementioned examples, we showed that ions with a PCC value ≥0.9 are mostly in-source fragments, natural isotopes, or alkali metal adducts (e.g., Na^+^ and K^+^ adducts) of the precursor ion, and they can be used for precursor ion identification. In addition, many additional ions with PCC values <0.9 were also extracted using PICA, and they showed spatial distribution similar to the precursor ion. Next, we investigated if PICA could assist in identifying these “moderately colocalized” ions.

**Figure 5:**
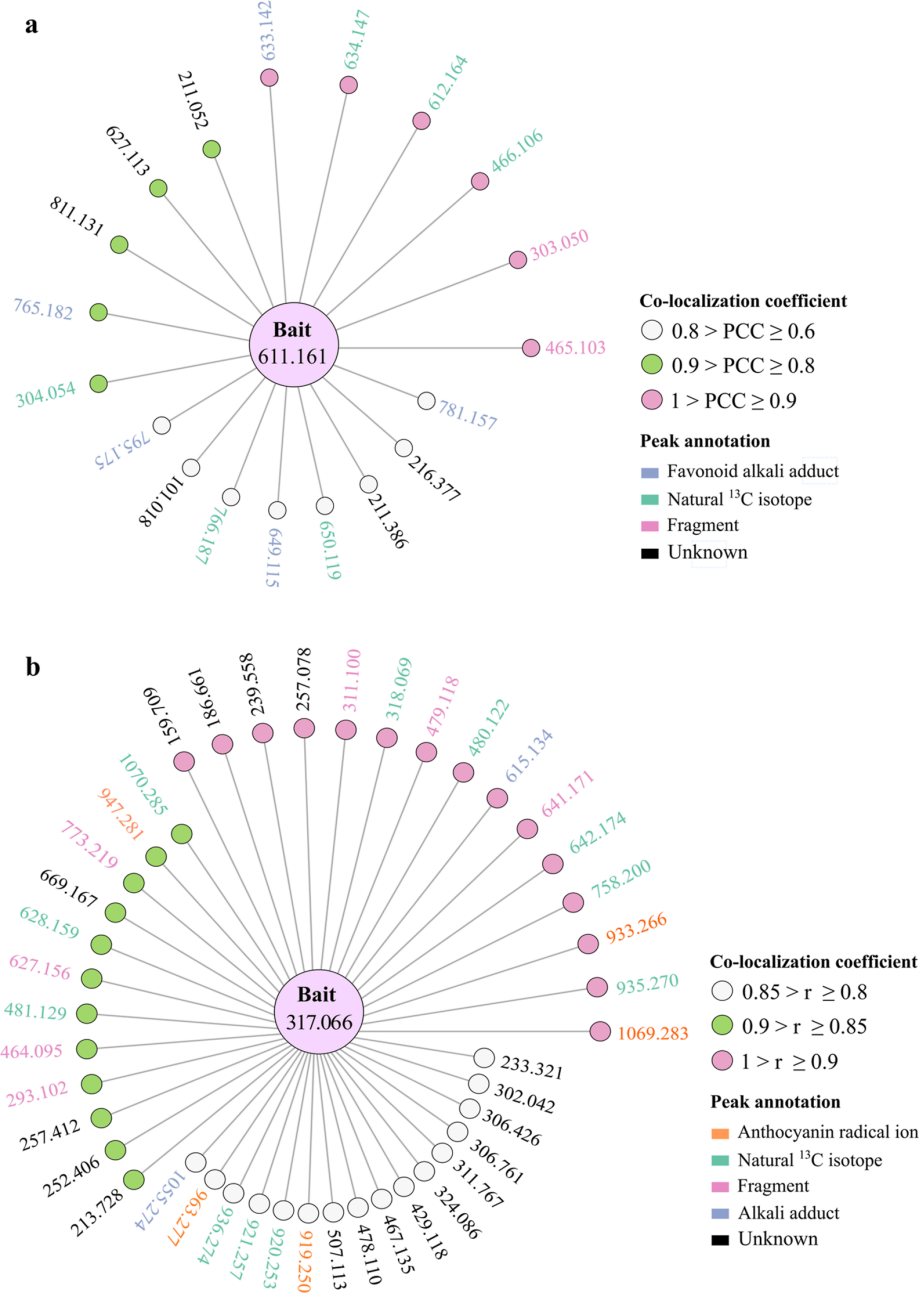
Colocalization networks for the anthocyanidins rutin and petunidin. (a) The protonated rutin ion ([M + H]^+^, *m*/*z* 611.161) was used as bait, and 19 ions were found colocalized with it. (b) The radical ion of petunidin ([M]^+^, *m*/*z* 317.066) was used as bait, and 43 ions were found colocalized with it. These colocalized ions were classified into three groups according to their PCC values: group 1 with PCC ≥ 0.9 (highlighted by purple solid circles); group 2 with 0.9 > PCC ≥ 0.8 for rutin, and 0.9 > PCC ≥ 0.85 for petunidin (highlighted by green solid circles); and group 3 with 0.8 > PCC value ≥ 0.6 for rutin, and 0.85 > PCC ≥ 0.8 for petunidin (highlighted by white solid circles). These ions were then identified by inspecting the raw MALDI imaging data and LC–MS/MS analysis of tomato homogenate. Each identified chemical species is represented by a unique color code. Note that the distance between bait ion and the colocalized ion is irrelevant to the PCC scores here.

Apart from the 6 rutin-derived ions shown in Figure 3 from dataset 1, 13 additional ions were found to colocalize with rutin, possessing PCC values ranging from 0.6 to 0.9 (Figure 5a). MALDI images confirmed that all 13 ions exhibit a similar spatial distribution to rutin (Figure S4). Of the 13 ions, 7 were identified as flavonols adducts or their respective ^13^C natural isotopes, including potassium adduct of rutin (C_27_H_30_O_16_, [M + K]^+^, *m*/*z* 649.115), sodium and potassium adducts of rutin-pentoside (C_32_H_38_O_20_, [M + Na]^+^, *m*/*z* 765.182; [M + K]^+^, *m*/*z* 781.157), and potassium adduct of kaempferol 3-rutinoside-7-glucoside (C_33_H_40_O_20_, [M + K]^+^, *m*/*z* 795.175). The availability of rutin and rutin-pentoside standards allowed us to confidently identify these two metabolites by comparing the retention time and MS/MS mass spectrum obtained from tomato skin to those generated by authentic standards analyzed using the same LC–MS/MS method (level-1 annotation). Interestingly, all three identified co-occurred ions share a common fragment ion *m*/*z* 611.161 (Figure S5), indicating that structurally similar molecules (e.g., derived from the same precursor) may have similar spatial distribution. As such, PICA can be also applied to cluster structurally similar metabolites and identify them according to known ones.

To further test the power of metabolite identification by clustering structurally similar metabolites, we applied PICA to anthocyanins, a class of pigments that are prone to ISF in MALDI.^43^ This dataset was obtained from a previous study, in which anthocyanin-rich tomato fruit generated by ectopic expression of the snapdragon *ROSEA1* (*ROS1*, a MYB-type) and *DELILA* (*DEL*, a bHLH-type) transcription factors were subjected to high-mass-resolution MALDI imaging analysis.^26,44^ Anthocyanin production in the ROS1/DEL tomato fruit was locally reduced by using the virus-induced gene-silencing technique, resulting in irregular accumulation of anthocyanins in fruit at the red, ripe stage. In this earlier study, seven anthocyanins were identified using MALDI imaging and on-tissue MALDI-MS/MS, corresponding to three sugar-free anthocyanins (i.e., anthocyanidins), petunidin (*m*/*z* 317.066, [M]^+^), malvidin (*m*/*z* 331.081, [M]^+^), and delphinidin (*m*/*z* 303.050, [M]^+^), with their glycosyl and acyl moieties. Here, these three anthocyanidins were used as baits for anthocyanin identification. In the case of petunidin, 43 distinct ions were found colocalized with it with PCC values ≥0.8 (Figure S6), of which 16 were with PCC values between 0.8 and 0.85, 12 between 0.85 and 0.9, and 15 between 0.9 and 1 (Figure 5b). MALDI images confirmed that all the 43 ions have a spatial distribution similar to petunidin. In the previous study, three petunidin-type anthocyanins were identified with MALDI, including petunidin 3-(*p*-coumaroyl)-rutinoside-5-glucoside (C_43_H_49_O_23_^+^, [M]^+^, *m*/*z* 933.266), petunidin 3-(caffeoyl)-rutinoside-5-glucoside (C_43_H_49_O_24_^+^, [M]^+^, *m*/*z* 949.262), and petunidin 3-(feruloyl)-rutinoside-5-glucoside (C_44_H_51_O_24_^+^, [M]^+^, *m*/*z* 963.278). Here, apart from petunidin 3-(caffeoyl)-rutinoside-5-glucoside, the other two petunidin-type anthocyanins were found moderately colocalized with the petunidin ion, with PCC values >0.80 (Figures 5b, S7, and S8). The PCC value of petunidin 3-(caffeoyl)-rutinoside-5-glucoside was 0.67, which is lower than that of the other two petunidin-type anthocyanins. One possible reason is the presence of a delphinidin-type anthocyanin isomer, delphinidin 3-(feruloyl)-rutinoside-5-glucoside (C_43_H_49_O_24_^+^, [M]^+^, *m*/*z* 949.262). This metabolite has the same elemental composition as petunidin 3-(caffeoyl)-rutinoside-5-glucoside, while its ion intensity was not highly spatially correlated with the petunidin ion, resulting in reduced PCC value for petunidin 3-(caffeoyl)-rutinoside-5-glucoside. In addition, one non-petunidin-type anthocyanins, delphinidin 3-(*p*-coumaroyl)-rutinoside-5-glucoside (C_42_H_47_O_23_^+^, [M]^+^, *m*/*z* 919.250), was also found as colocalized with the petunidin ion. The colocalization of this non-petunidin-type anthocyanin with the petunidin ion might be due to the fact that different anthocyanins have a similar spatial distribution in the transgenic tomato fruit. Interestingly, one unknown ion *m*/*z* 1069.283 was found highly colocalized with the petunidin ion with PCC value being 0.93. Unfortunately, this ion was not detected by LC–MS/MS analysis of the tomato fruit homogenate. Due to its low ion intensity, we were unable to perform on-tissue MALDI MS/MS of this ion. The elemental composition of this ion was calculated by its accurate mass using a 2-ppm mass window. This chemical formula was confirmed by comparing the isotopic distribution of the ion *m*/*z* 1969.283 and the simulated one using the formula given in Figure S9. Database searching in SciFinder (https://scifinder.cas.org) with this ion elemental composition (i.e., C_50_H_53_O_26_) revealed five candidates; all of them were stereoisomers of peonidin 3-(caffeoyl-*p*-hydroxybenzoyl sophoroside)-5-glucoside. This anthocyanin was detected previously in purple sweet potato,^45,46^ and peonidin-type anthocyanins have never been reported in ROS1/DEL tomato fruit. Nevertheless, the persistent presence of the ion *m*/*z* 1069.283 in different biological replicates and its high co-localization with the petunidin ion have led us to tentatively assign this ion as an anthocyanin-related ion.

In addition to petunidin, PICA was also applied to another two anthocyanidins: malvidin (*m*/*z* 331.081, [M]^+^) and delphinidin (*m*/*z* 303.050, [M]^+^). The previous study identified malvidin 3-(*p*-coumaroyl)-rutinoside-5-glucoside using MALDI imaging and on-tissue MALDI-MS/MS. Here, we detected this metabolite with PCC value as 0.77 (Figure S10). Compared to petunidin and malvidin, only one out of three previously detected delphinidin-type anthocyanins was found with the PCC value >0.65 (Figure S11). Apart from the above-discussed reason for isomer disturbance, another explanation is the presence of a major in-source fragment of rutin, *m*/*z* 303.050, which has the same accurate *m*/*z* value as the delphinidin radical ion (C_15_H_11_O_7_, M^+^). Therefore, PICA was in fact performed for both the rutin and delphinidin fragments. As this rutin fragment was distributed differently from delphinidin (Figure 3b), the power of extracting delphinidin-type anthocyanins was reduced. Indeed, the detection of colocalized flavonol-related ions confirmed that this rutin fragment negatively affected the PICA of the petunidin ion (Figure S11).

### PICA Limitations and Possible Solutions

One major limitation of PICA, particularly for high-mass-resolution and high-spatial-resolution MSI is that data analysis is often time-consuming. For instance, PICA takes ca. 9.5 min for rutin (Figure 3) using a computer with 16 GB memory and a 3.1 GHz Intel Core i7 processor. The computation time is proportional to the number of mass features and the number of pixels (i.e., time ∼ no. mass features × no. pixels). As such, we propose three solutions to reduce the total data analysis time. The first involves reducing the number of mass features. Small metabolites are typically singly charged in MALDI imaging;^47^ therefore, instead of using a complete mass range for PICA (i.e., 150–3000 Da for tomato fruit datasets), a mass range up to *m*/*z* value of the investigated precursor ion would be sufficient to detect its fragments. The second approach is to reduce the number of pixels. Metabolites are generally not localized in only a few pixels; therefore, every *n*th pixels could be used for colocalization analysis, which will reduce “*n*” times the total data analysis time. The third approach is to use regions of interest (ROI) rather than the whole measured sample region for PICA. The latter approach is particularly valuable for metabolites distributed in limited tissue areas.

**Figure 6:**
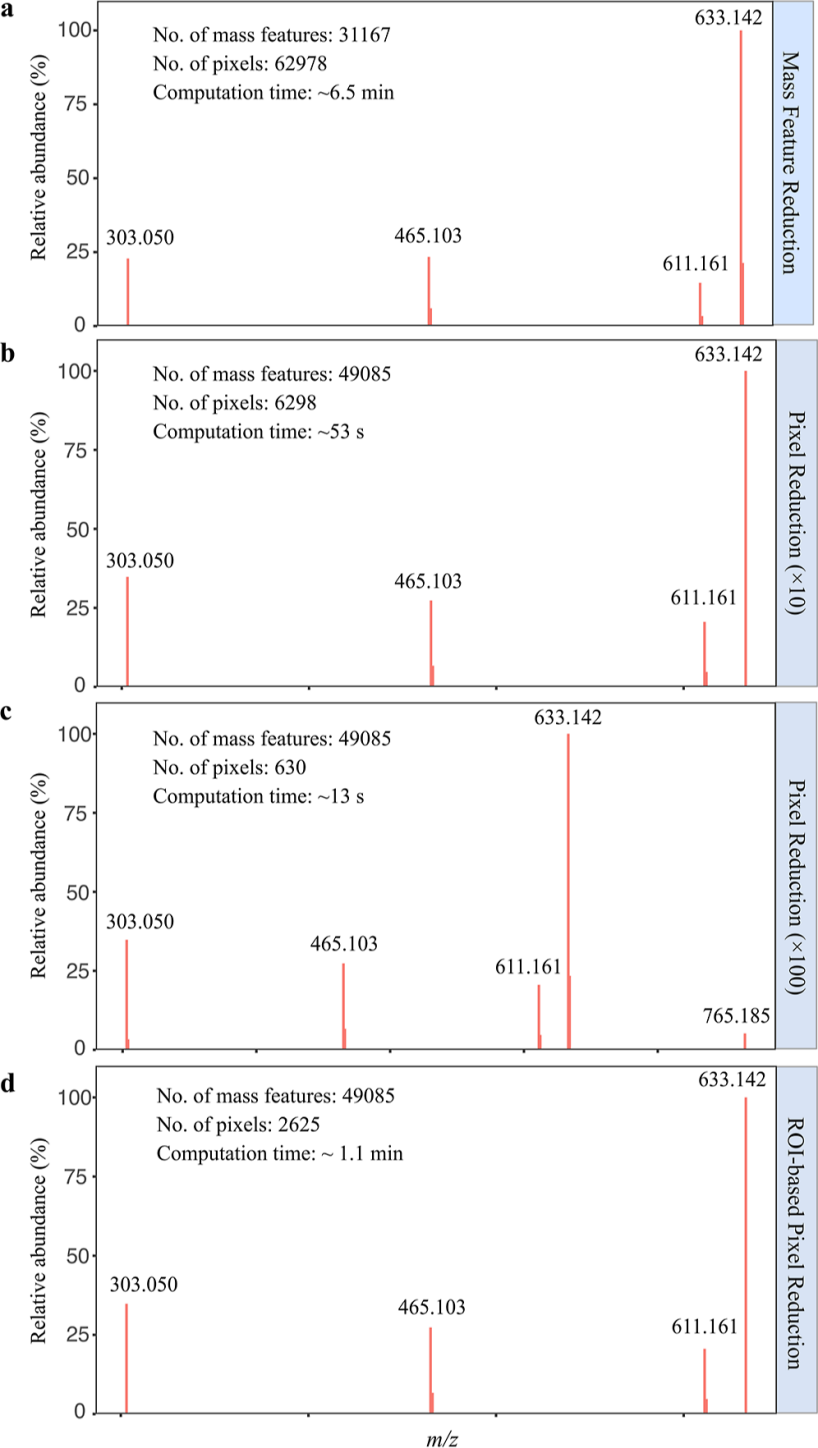
Evaluation of different strategies for reducing computation time when using PICA. (a) “Pseudo-rutin MS/MS spectrum” generated using the mass feature reduction approach, and the computation time was ca. 6.5 min. (b,c) “Pseudo-rutin MS/MS spectra” generated using every 10th and 100th pixel reduction approach, and the computation time was 53 and 13 s, respectively. (d) “Pseudo-rutin MS/MS spectrum” generated using a ROI-based pixel reduction approach, and the computation time was about 1.1 min.

To evaluate the effectiveness of the three approaches, PICA was performed for rutin ([M + H]^+^, *m*/*z* 611.161) with each one of the three strategies using the same dataset shown in Figure 3. The resulting “pseudo rutin MS/MS spectra” were then compared to the one produced using complete mass features and pixels as shown in Figure 3a. As for the mass feature reduction approach, a mass range of up to 650 Da was used, and the corresponding number of mass features was reduced from 49,580 to 31,167. The computation time for this method was reduced from 9.5 to 6.5 min. The “pseudo MS/MS spectrum” was found identical to the one shown in Figure 3a (Figure 6a). Every 10th and 100th pixels were used to evaluate the pixel reduction method. The number of pixels for colocalization analysis was reduced to 6298 and 630; consequently, the total data analysis time was reduced to ca. 53 and 13 s, respectively. The “pseudo rutin MS/MS spectrum” of every 10th pixel method is similar to the original one except that the ^13^C natural isotope peak of the rutin sodium adduct peak is missing (Figure 6b). As for the every 100th pixel method, one additional ion, *m*/*z* 765.182 (identified as sodium adduct of rutin-pentoside, C_32_H_38_O_20_, [M + Na]^+^), was included in the “pseudo rutin MS/MS spectrum” (Figure 6c). The total number of pixels was reduced from 62,978 to 2625 for the ROI-based pixel reduction method, and the data analysis time was ca. 1.1 min. The resulting “pseudo MS/MS spectrum” is identical to the one obtained using every 10th pixel reduction method (Figure 6d). Nevertheless, both two major rutin fragments, *m*/*z* 465.103 and *m*/*z* 303.050, were detected using all three proposed approaches. The computation time was reduced from 30% for the mass feature reduction method to 2.2% for every 100th pixel method. It is worth noting that each of the proposed approaches has its own limitations. For instance, the mass feature reduction approach is not effective for metabolites with higher *m*/*z* values; the pixel reduction method may not work well for metabolites with nonuniform distributions; and the ROI-based pixel reduction method becomes less efficient for metabolites widely distributed over the entire sample. Therefore, these methods should be applied according to the *m*/*z* values and their spatial distribution for a particular experiment. In addition, the three approaches can be combined during colocalization analysis to significantly reduce computation time.

As not all molecules have in-source fragments during MALDI imaging analysis, PICA is only limited to molecules with good ISF yields. Approaches to improve ISF in MALDI, such as developing new MALDI matrices and modifying instrument configuration (e.g., increasing laser power and introducing broadband collision-induced dissociation or in-source decay in MALDI imaging method), will allow for the detection of in-source fragments from a broader range of metabolites. In addition, high PCC values (e.g., PCC ≥ 0.9) may not be expected between a precursor and its fragments when the fragments are shared by other metabolites, particularly in the case of metabolites possessing different spatial distributions. Similarly, different precursor-derived fragments with the same *m*/*z* values may also disturb colocalization analysis as exemplified here for delphinidin (Figure S6).

It is important to note that the use of PCC ≥ 0.9 as the threshold for selecting correlated ions is based on subjective experience in this study. Unfortunately, we cannot suggest a universal and perfect PCC threshold value. As discussed above, ions originating from the same metabolite are in theory perfectly colocalized (PCC = 1); the use of PCC ≥ 0.9 is, therefore, a suitable threshold value for selecting the correlated ions. Indeed, many factors, particularly the presence of isomers and different fragment ions with the same *m*/*z* values, could disrupt the calculation of PCC value, leading to a reduced number of extracted ions. On the other hand, although it may fail to extract all ions derived from the same metabolite, the use of a higher PCC threshold value would allow for the selection of more specific ions of that metabolite, thus allowing for metabolite identification with high confidence. Our results show that moderately colocalized ions (PCC < 0.9) may be structurally related to the given ion, which can be also used to identify unknown metabolites through the known ones.

## Conclusions

Regardless of its “soft ionization” nature, ISF widely exists in MALDI. Due to the lack of chromatographic separation in MSI, in-source fragments are generally indistinguishable from intact endogenous molecules. As such, ISF can cause difficulties in metabolite identification, particularly when it generates undesirable fragments of identical *m*/*z* values to common metabolites in complex biological samples. This could lead to mis-annotation, false identification, and wrong interpretation of the biological data. In this study, we took advantage of ISF and used the PICA approach for metabolite identification in MALDI imaging. Our results showed that the highly colocalized ions with PCC ≥ 0.9 for a given ion are mainly its in-source fragments, natural isotopes, adduct ions, or multimers. A “pseudo MS/MS” spectrum can be produced with these extracted ions, which is then used for metabolite identification. In addition, we have observed that structurally related metabolites tend to co-occur, which provides the possibility of identifying unknowns through the known ones. Finally, we have proposed three approaches to reduce computation time for PICA. To conclude, PICA allows extracting and grouping ions stemming from the same metabolites in MALDI imaging, thus offering valuable chemical information for metabolite identification. PICA is also likely to be valuable in studies employing other MSI modalities such as DESI and SIMS imaging in which a significant proportion of in-source fragments are observed. Future studies will attempt to enhance ISF in MSI and apply PICA for on-tissue protein identification in MSI.

## Supporting Information Available

*m*/*z* values, PCC scores, annotation, and mass accuracies of colocalized ions; MS images of colocalized ions; LC-MS/MS of rutin, rutin-pentoside, and kaempferol 3-rutinoside-7-glucoside; PICA-assisted petunidin 3-(p-coumaroyl)-rutinoside-5-glucoside identification; and colocalization networks (PDF)
Reproducible example of colocalization analysis (PDF)
