# Supplementary material for: PICA: Pixel Intensity Correlation Analysis for Deconvolution and Metabolite Identification in Mass Spectrometry Imaging
Source: Anal Chem. 2023 Jan 3;95(2):1652–62. doi: 10.1021/acs.analchem.2c04778 (PMC9850408; doi:10.1021/acs.analchem.2c04778)
Supplement: Supplementary file 1 — ac2c04778_si_001.pdf [file ac2c04778_si_001.pdf]

<u>Supporting Information for the manuscript entitled:</u>

Pixel Intensity Correlation Analysis for Deconvolution and Metabolite Identification in Mass Spectrometry Imaging

Yonghui Dong[1,2], Nir Shachaf[1], Liron Feldberg[3], Ilana Rogachev[1], Uwe Heinig[1,2], Asaph Aharoni[1*]

[1]Department of Plant Sciences, Weizmann Institute of Science, Rehovot, 7610001, Israel

[2]Department of Life Sciences Core Facilities, Weizmann Institute of Science, Rehovot 7610001, Israel

[3]Department of Analytical Chemistry, Israel Institute for Biological Research, Ness Ziona, 7410001, Israel

[*]Corresponding author. Tel.: +972 544 784259

E-mail: asaph.aharoni@weizmann.ac.il

Competing interests

The authors declare no competing financial interests.

Content

Table S1 The *m/z* values, PCC scores, annotation, and mass accuracies of the 26 ions colocalizedwith DHB peak *m/z* 409.055.

Figure S1 MS images of ions highly colocalized (PCC score >= 0.9) with DHB peak *m/z* 409.055.

Table S2 The *m/z* values, PCC scores, annotation, and mass accuracies of the 6 ions colocalized with rutin peak *m/z* 611.161.

Figure S2 MS images of ions highly colocalized (PCC score >= 0.9) with rutin peak *m/z* 611.161.

Figure S3 MALDI imaging of mouse cerebellum.

Figure S4 MS images of ions moderately colocalized (0.9> PCC score >= 0.6) with rutin peak *m/z* 611.161.

Figure S5 LC-MS/MS of rutin, rutin-pentoside, and kaempferol 3-rutinoside-7-glucoside.

Figure S6 MS images of ions highly and moderately colocalized (PCC score >= 0.8) with petunidin peak *m/z* 317.066.

Figure S7 PICA-assisted petunidin 3-(p-coumaroyl)-rutinoside-5-glucoside identification.

Figure S8 MS images of ions highly and moderately colocalized (PCC score >= 0.8) with petunidin 3-(p-coumaroyl)-rutinoside-5-glucoside peak *m/z* 933.266.

Figure S9 Comparison of ion peak *m/z* 1069.28289 and its ^{13}C isotope peaks with the simulatedpeaks calculated from the elemental composition $C_{50}H_{53}O_{26}$ ($[M^{+\cdot}]$) at positive ion mode.

Figure S10 Colocalization networks for anthocyanidin malvidin.

Figure S11 Colocalization networks for the anthocyanidin delphinidin.

Table S1 The *m/z* values, PCC scores, annotation, and mass accuracies of the 26 ions colocalized with DHB peak *m/z* 409.055.

**ID**	**Measured *m/z***	**Identity**	**PCC score**	**Exact *m/z***	**mass accuracy (ppm)**
1	409.0552	[3DHB-3$H_2$0+H]$^+$	1	409.0554	-0.5
2	273.0393	[2DHB-2$H_2$0+H]$^+$	0.99	273.0394	0
3	274.0428	13C isotope of 2	0.99	273.0428	0
4	291.0493	[2DHB-$H_2$0+H]$^+$	0.98	291.0499	-2.1
5	290.0433		0.98		
6	137.0232	[DHB-$H_2$0+H]$^+$	0.97	137.0233	-0.7
7	427.0624		0.97		
8	136.3534		0.95		
9	439.0662	Unknown DHB peak	0.95		
10	292.0535	13C isotope of 4	0.94	292.0533	0.68
11	512.0028		0.94		
12	136.5199		0.94		
13	440.0684	13C isotope of 9	0.94	\	\
14	438.0575		0.94		
15	361.1055	Unknown DHB peak	0.93		
16	146.3572		0.93		
17	275.0448	13C isotope of 2	0.92	275.0449	-0.36
18	296.2578		0.92		
19	575.0806		0.92		
20	280.2635		0.91		
21	246.0538		0.91		
22	519.0240		0.91		
23	435.2977		0.91		
24	317.0047	[2DHB-2$H_2$0-H+2Na]$^+$	0.91	317.0038	2.83
25	289.0357		0.91		
26	410.0583	13C isotope of 1	0.91	410.0588	-1.21
27	155.0339	[DHB+H]$^+$	0.91	155.0339	0

Figure S1 MS images of ions highly colocalized (PCC score >= 0.9) with DHB peak *m/z* 409.055.

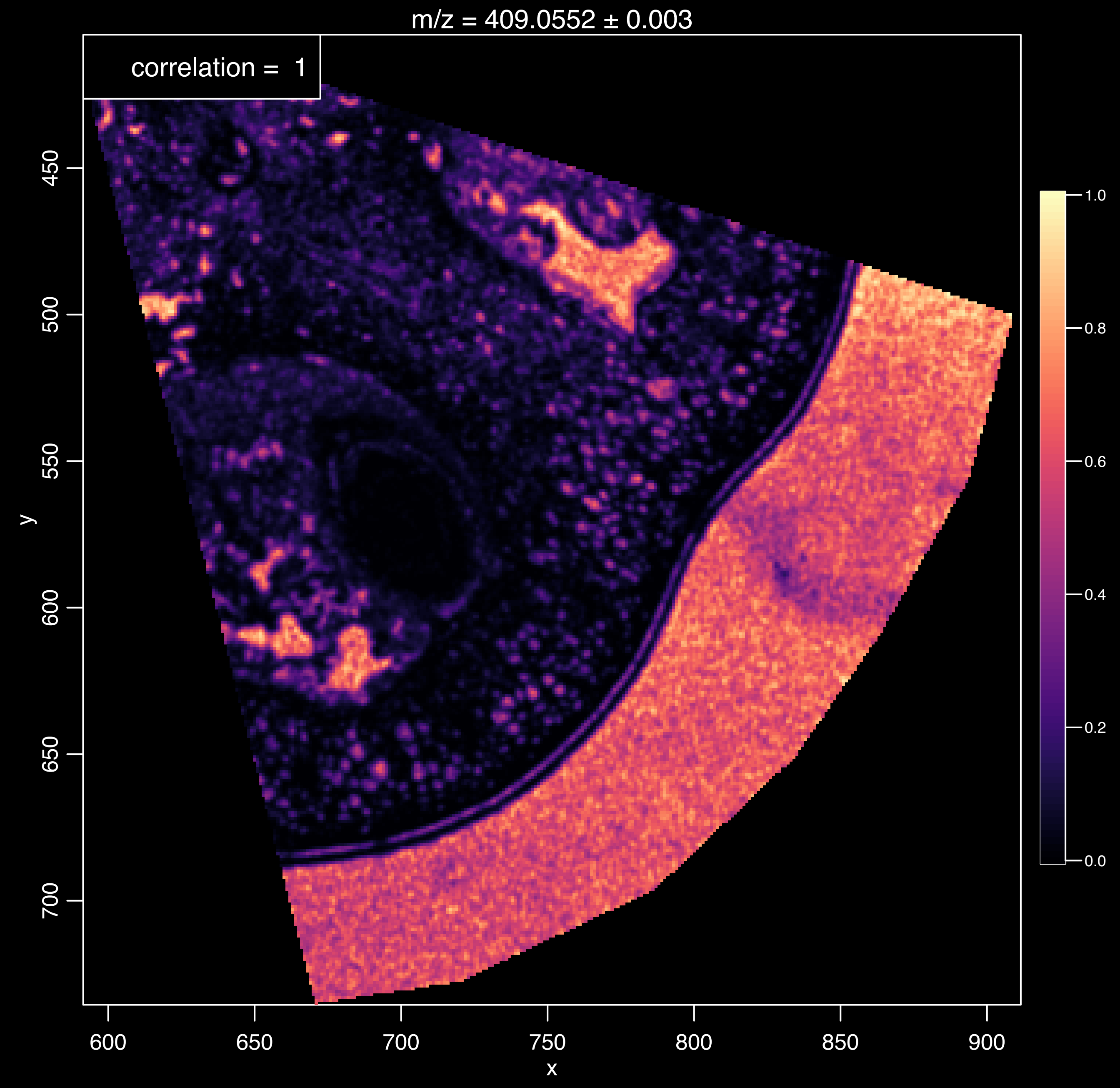
m/z = 409.0552 ± 0.003
correlation = 1
y
x
450
500
550
600
650
700
600
650
700
750
800
850
900
1.0
0.8
0.6
0.4
0.2
0.0

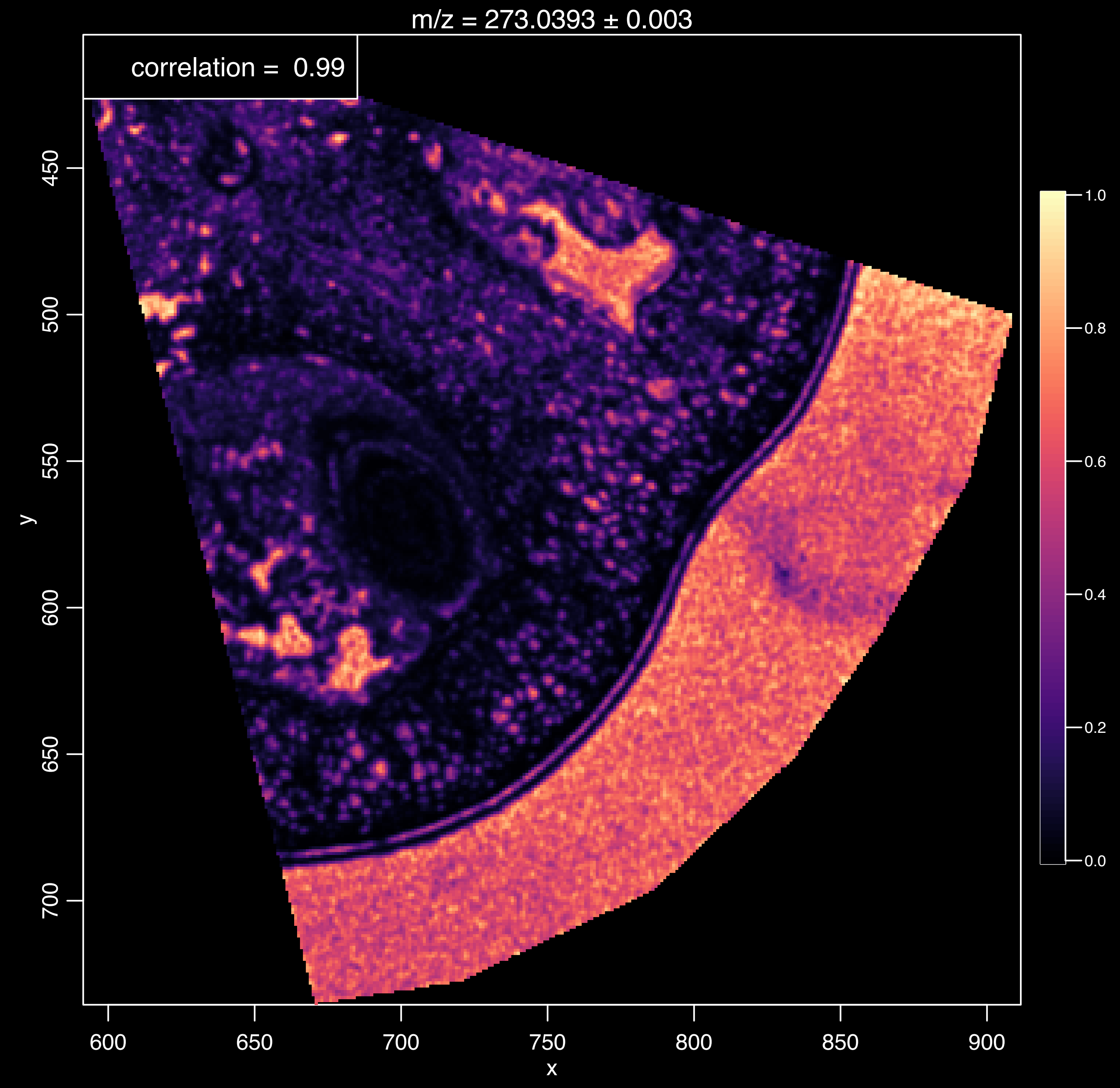

m/z = 273.0393 ± 0.003
correlation = 0.99
y
450
500
550
600
650
700
x
600
650
700
750
800
850
900
1.0
0.8
0.6
0.4
0.2
0.0

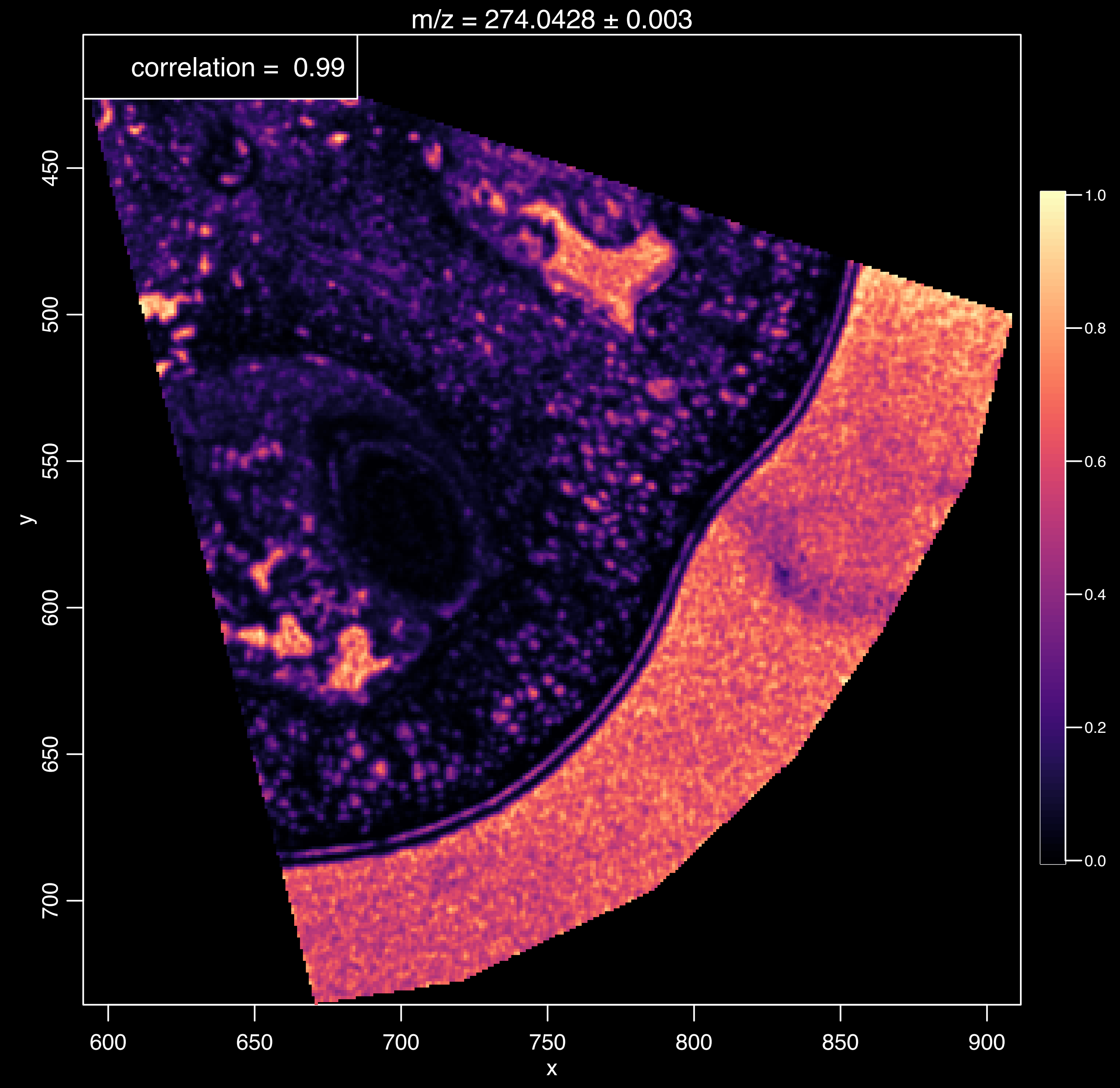

m/z = 274.0428 ± 0.003
correlation = 0.99
450
500
550
600
650
700
y
600
650
700
750
800
850
900
x
1.0
0.8
0.6
0.4
0.2
0.0

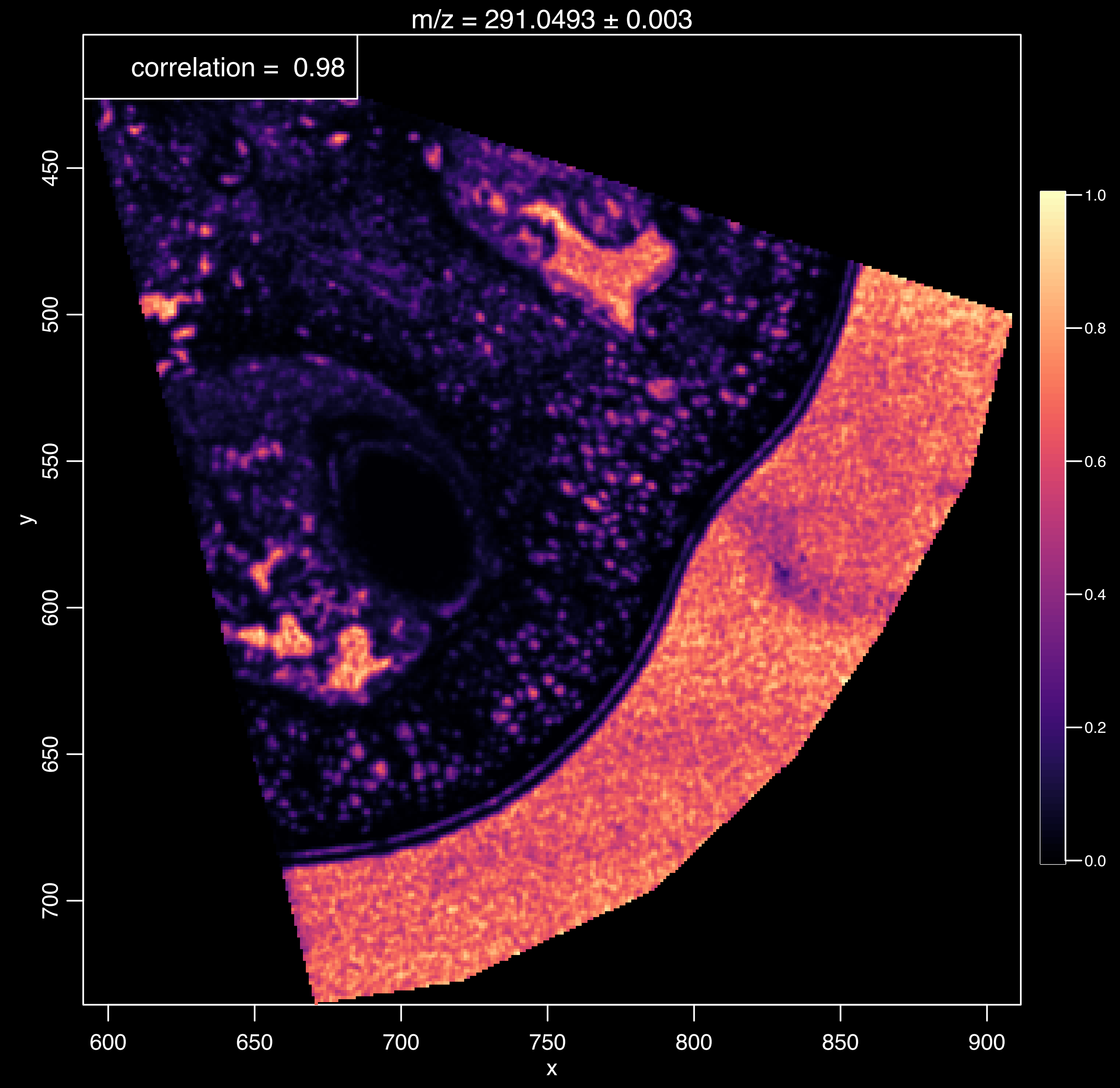

m/z = 291.0493 ± 0.003
correlation = 0.98
450
500
550
600
650
700
y
600
650
700
750
800
850
900
x
1.0
0.8
0.6
0.4
0.2
0.0

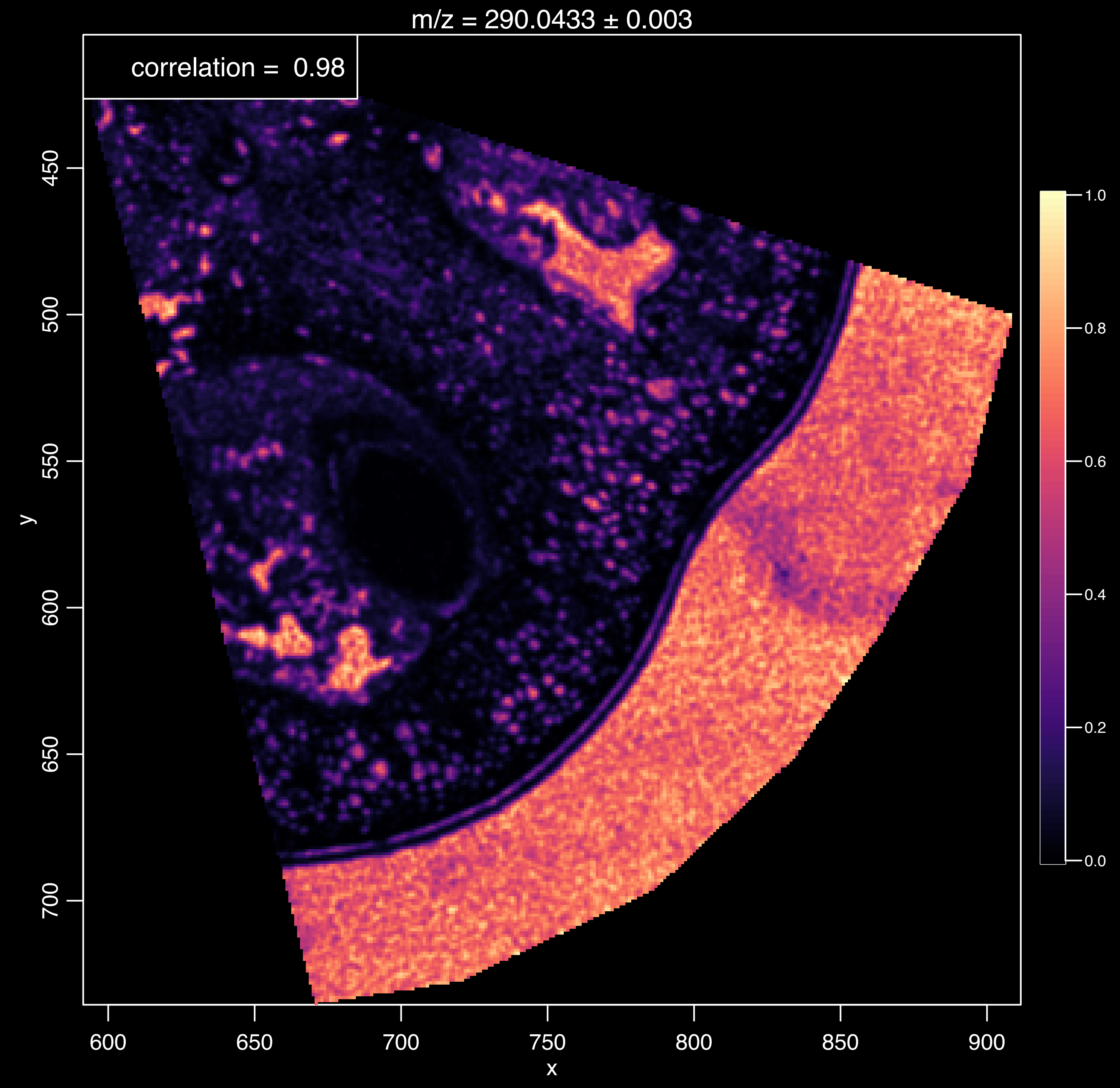

m/z = 290.0433 ± 0.003
correlation = 0.98
y
x
450
500
550
600
650
700
600
650
700
750
800
850
900
1.0
0.8
0.6
0.4
0.2
0.0

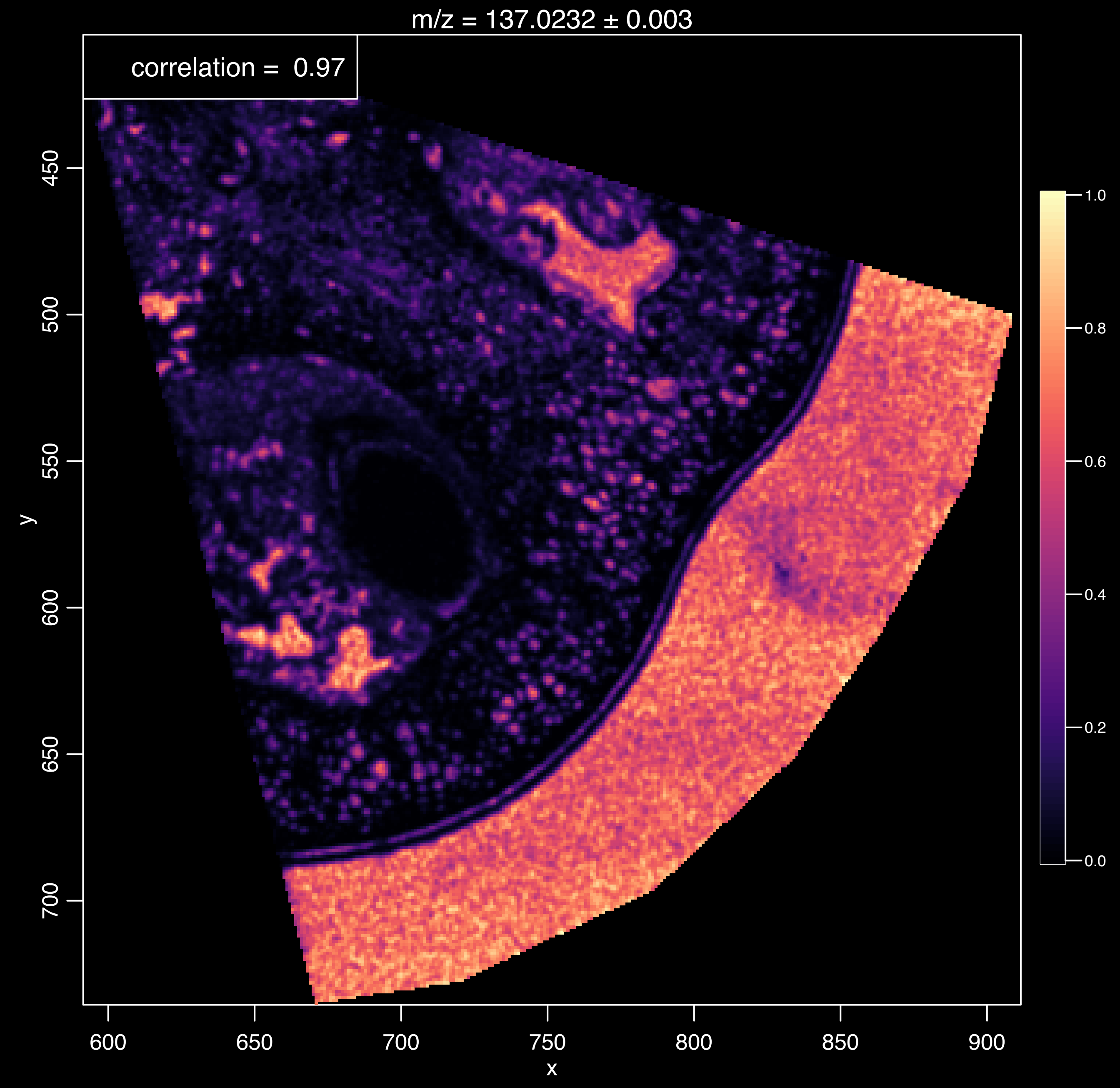

m/z = 137.0232 ± 0.003
correlation = 0.97
y
450
500
550
600
650
700
x
600
650
700
750
800
850
900
1.0
0.8
0.6
0.4
0.2
0.0

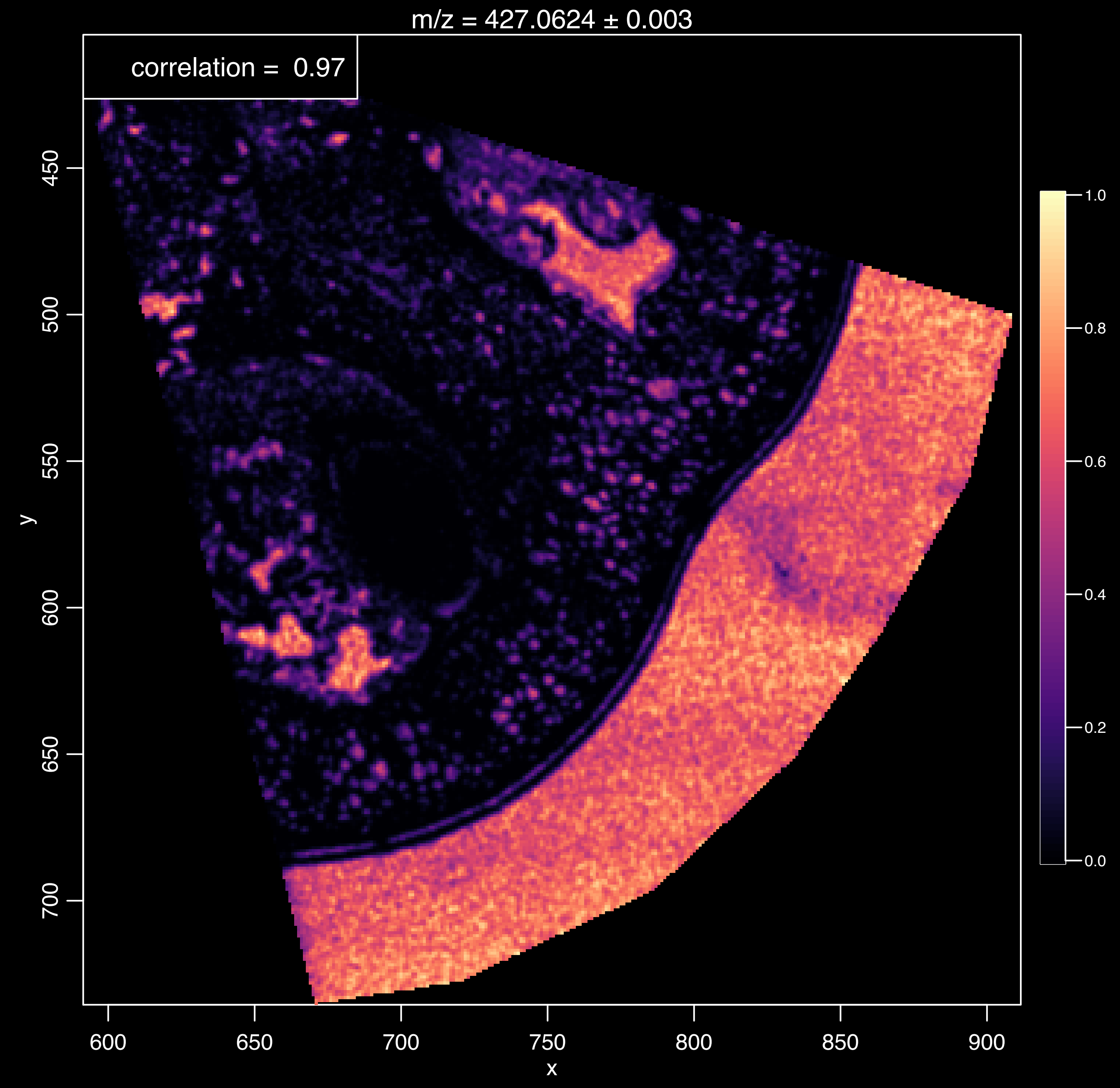

m/z = 427.0624 ± 0.003
correlation = 0.97
y
450
500
550
600
650
700
x
600
650
700
750
800
850
900
1.0
0.8
0.6
0.4
0.2
0.0

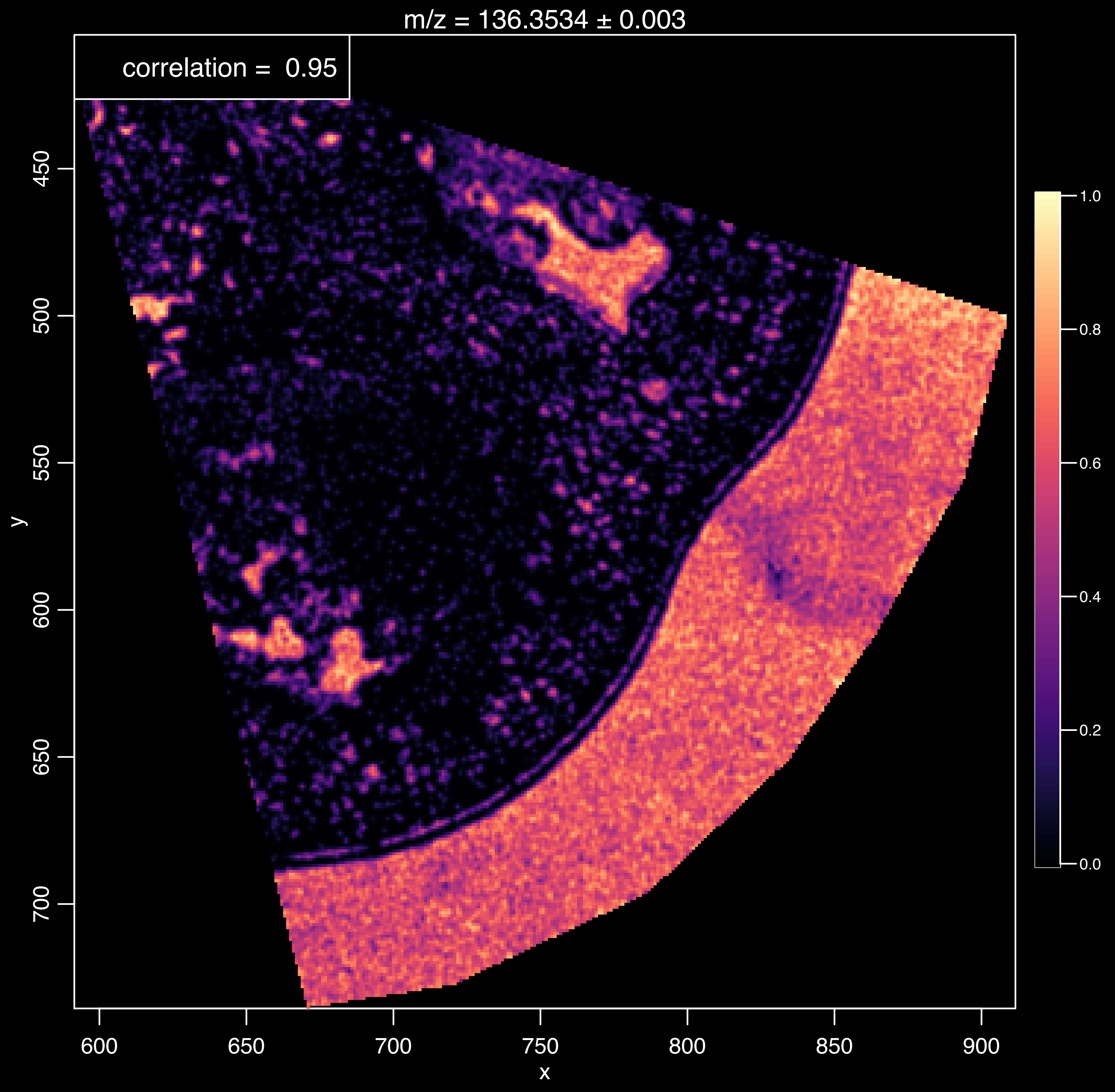

m/z = 136.3534 ± 0.003
correlation = 0.95
450
500
550
600
650
700
y
600
650
700
750
800
850
900
x
1.0
0.8
0.6
0.4
0.2
0.0

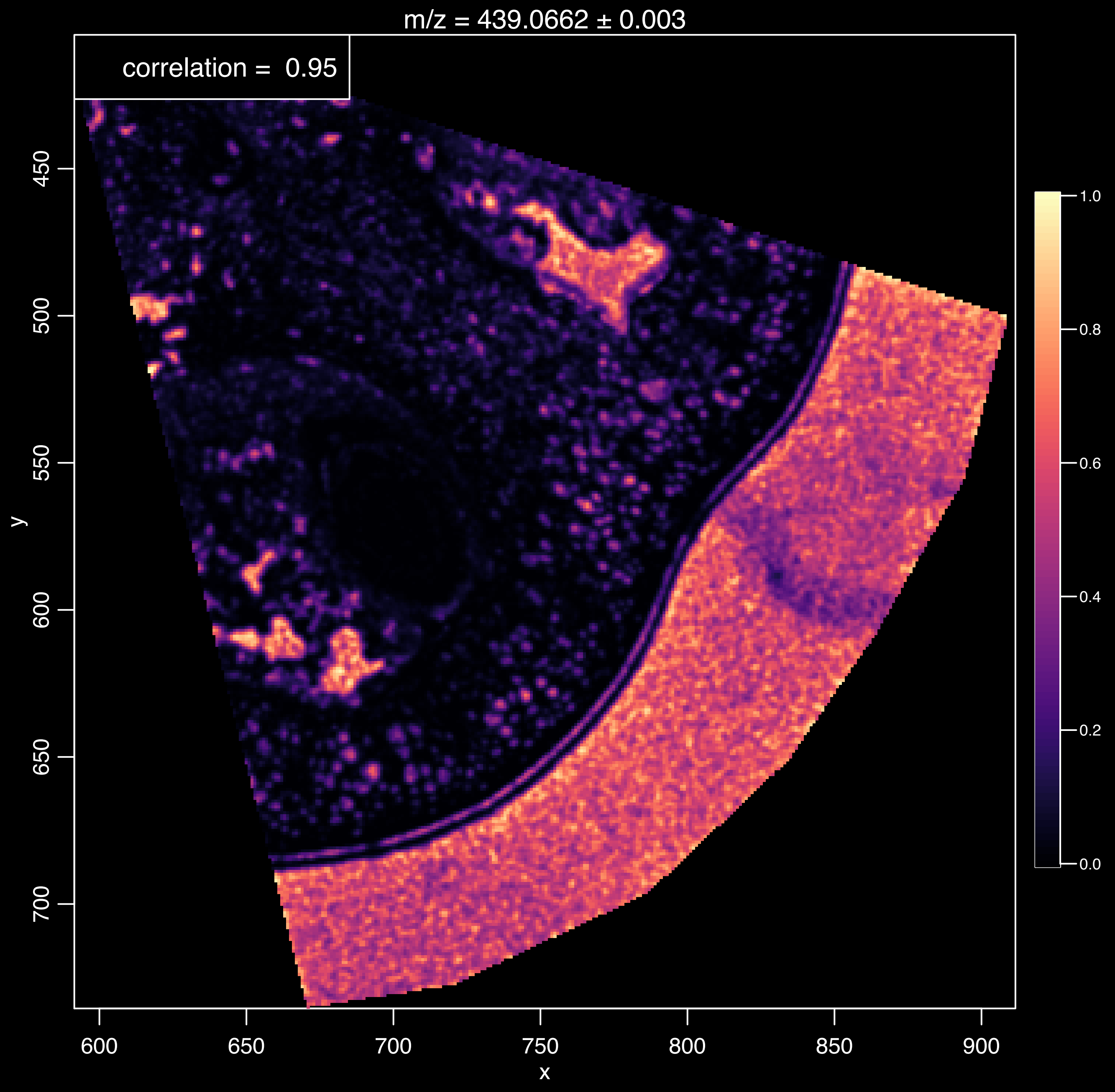

m/z = 439.0662 ± 0.003
correlation = 0.95
450
500
550
600
650
700
y
600
650
700
750
800
850
900
x
1.0
0.8
0.6
0.4
0.2
0.0

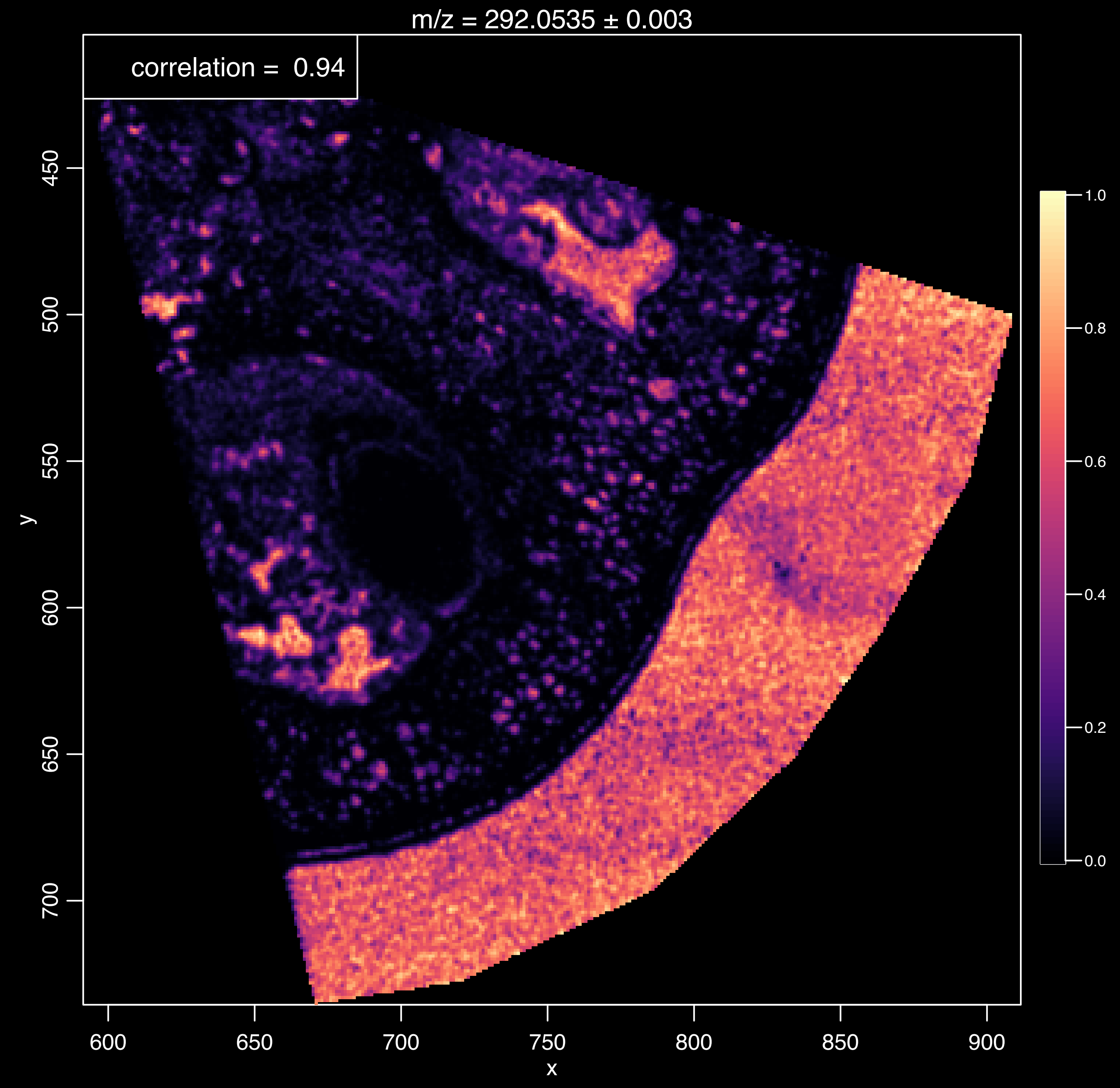

m/z = 292.0535 ± 0.003
correlation = 0.94
450
500
550
600
650
700
y
600
650
700
750
800
850
900
x
1.0
0.8
0.6
0.4
0.2
0.0

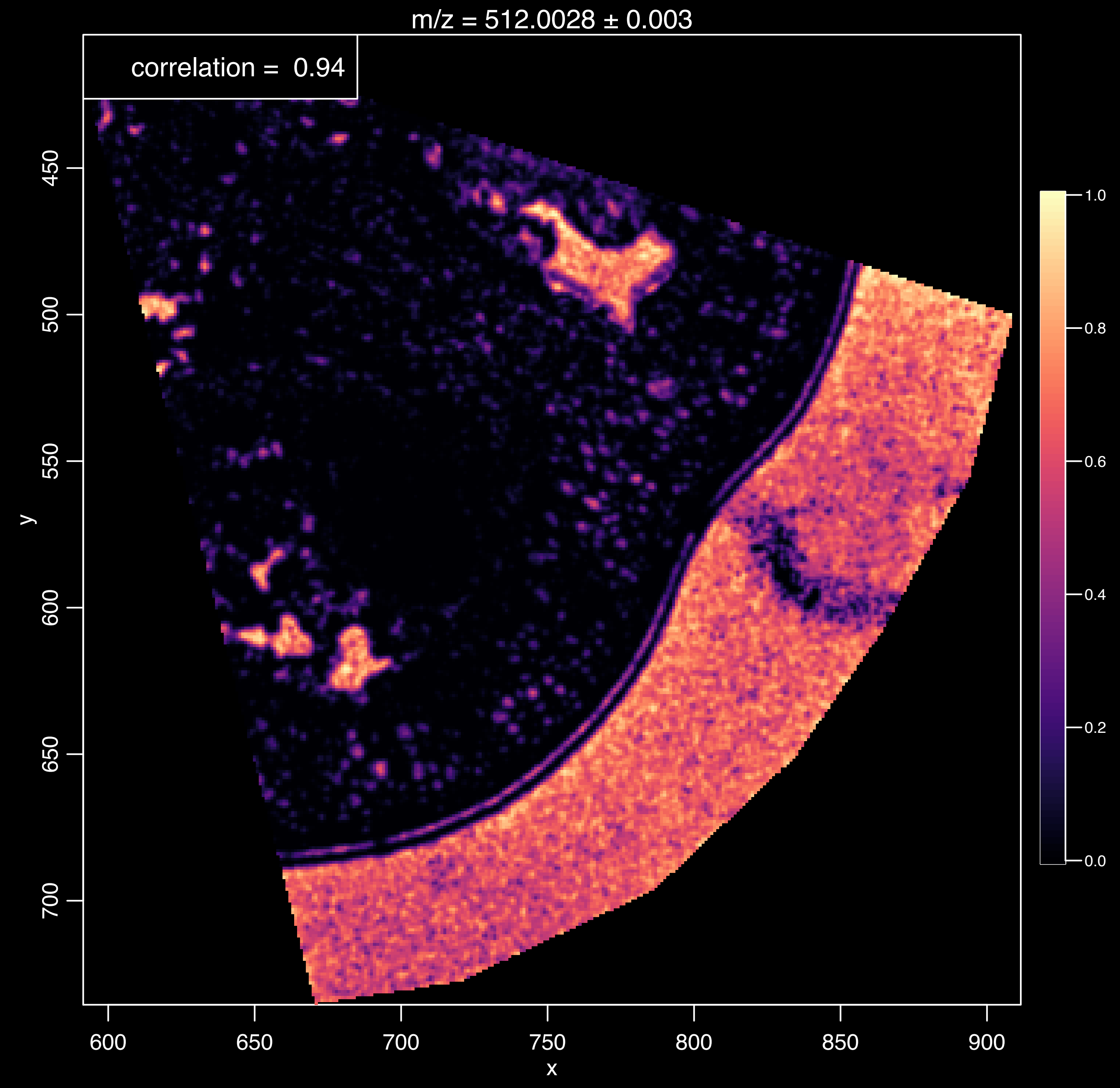

m/z = 512.0028 ± 0.003
correlation = 0.94
450
500
550
600
650
700
y
600
650
700
750
800
850
900
x
1.0
0.8
0.6
0.4
0.2
0.0

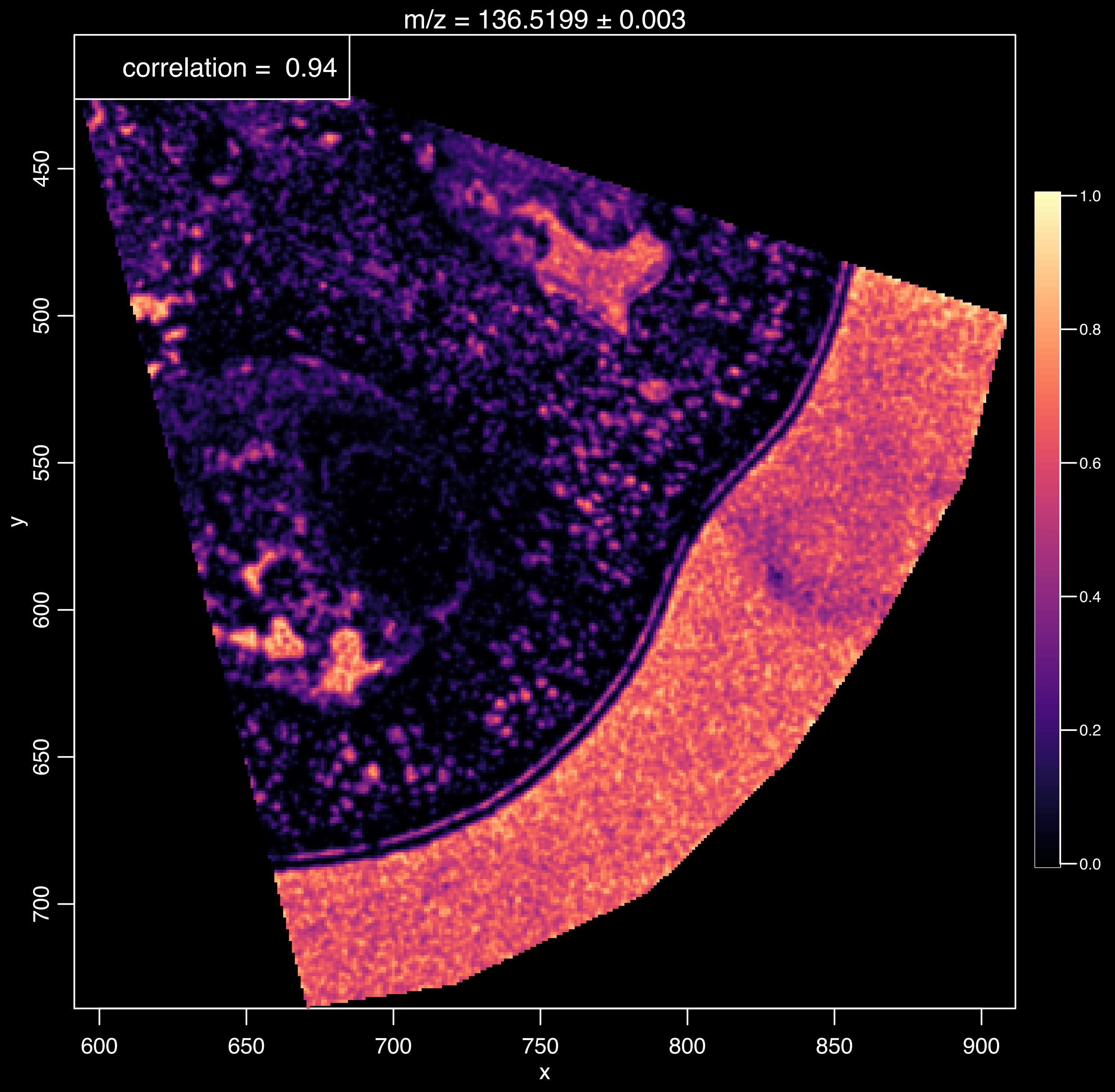

m/z = 136.5199 ± 0.003
correlation = 0.94
y
450
500
550
600
650
700
x
600
650
700
750
800
850
900
1.0
0.8
0.6
0.4
0.2
0.0

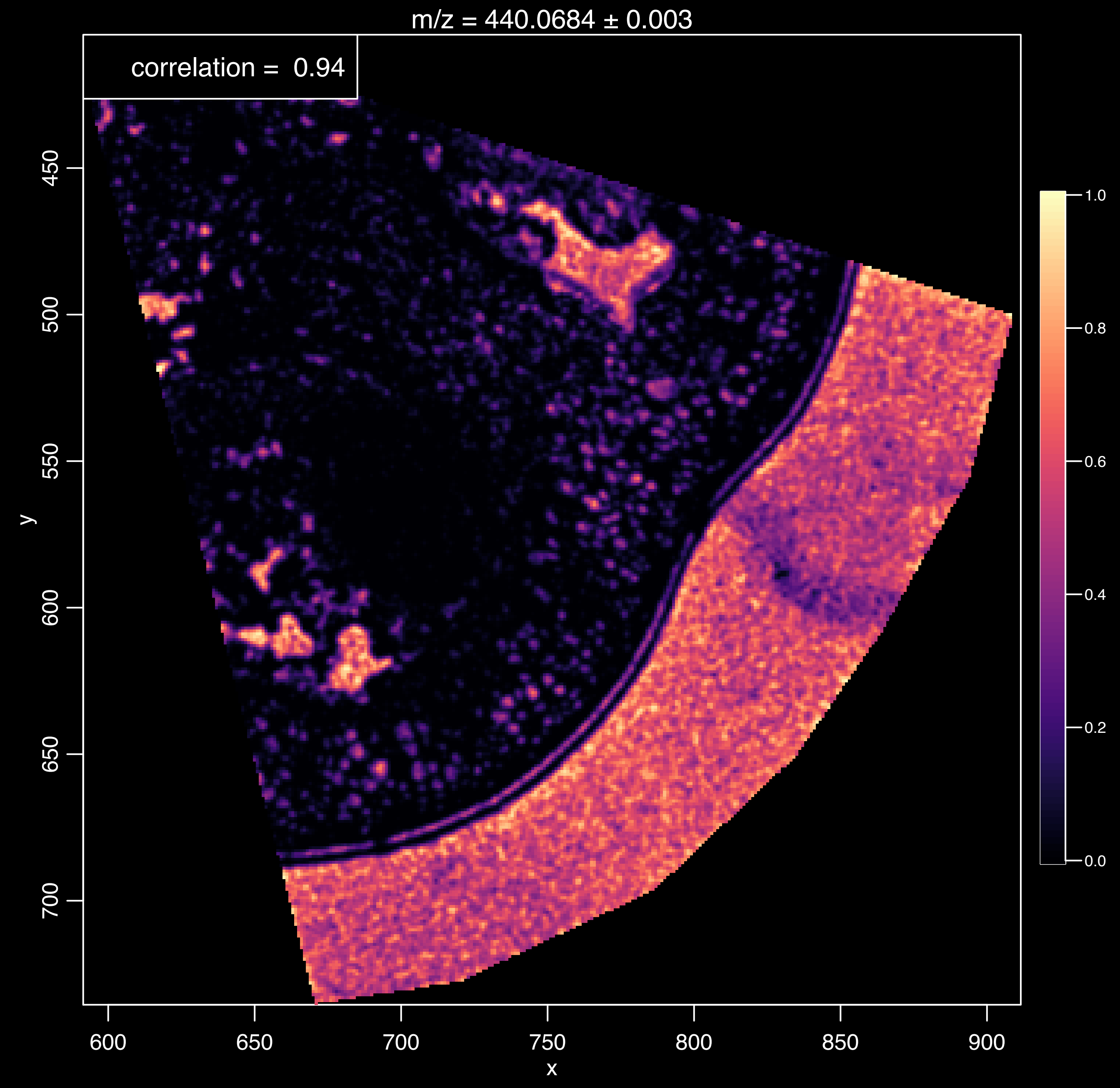

m/z = 440.0684 ± 0.003
correlation = 0.94
450
500
550
600
650
700
y
600
650
700
750
800
850
900
x
1.0
0.8
0.6
0.4
0.2
0.0

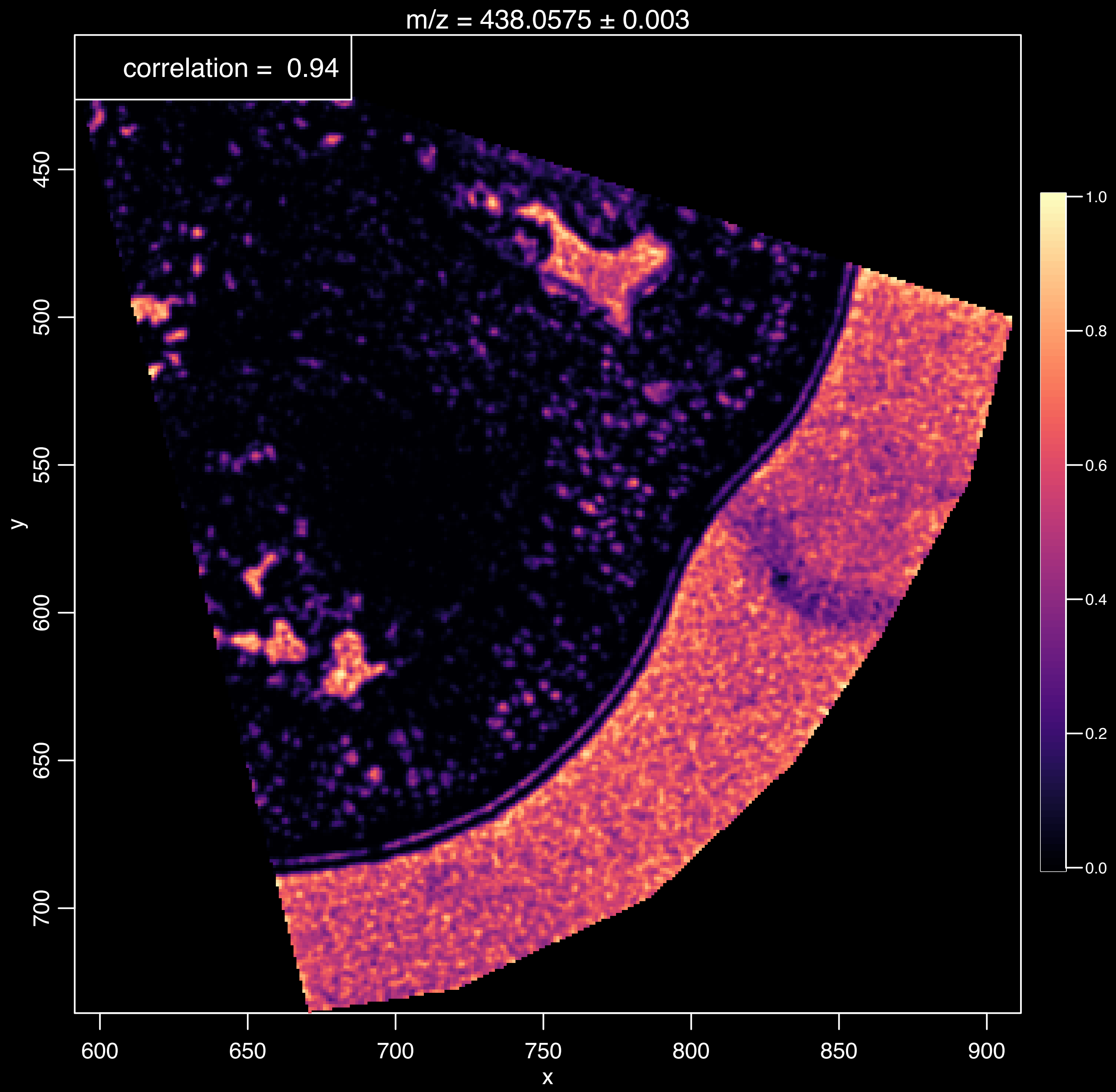

m/z = 438.0575 ± 0.003
correlation = 0.94
450
500
550
600
650
700
y
600
650
700
750
800
850
900
x
1.0
0.8
0.6
0.4
0.2
0.0

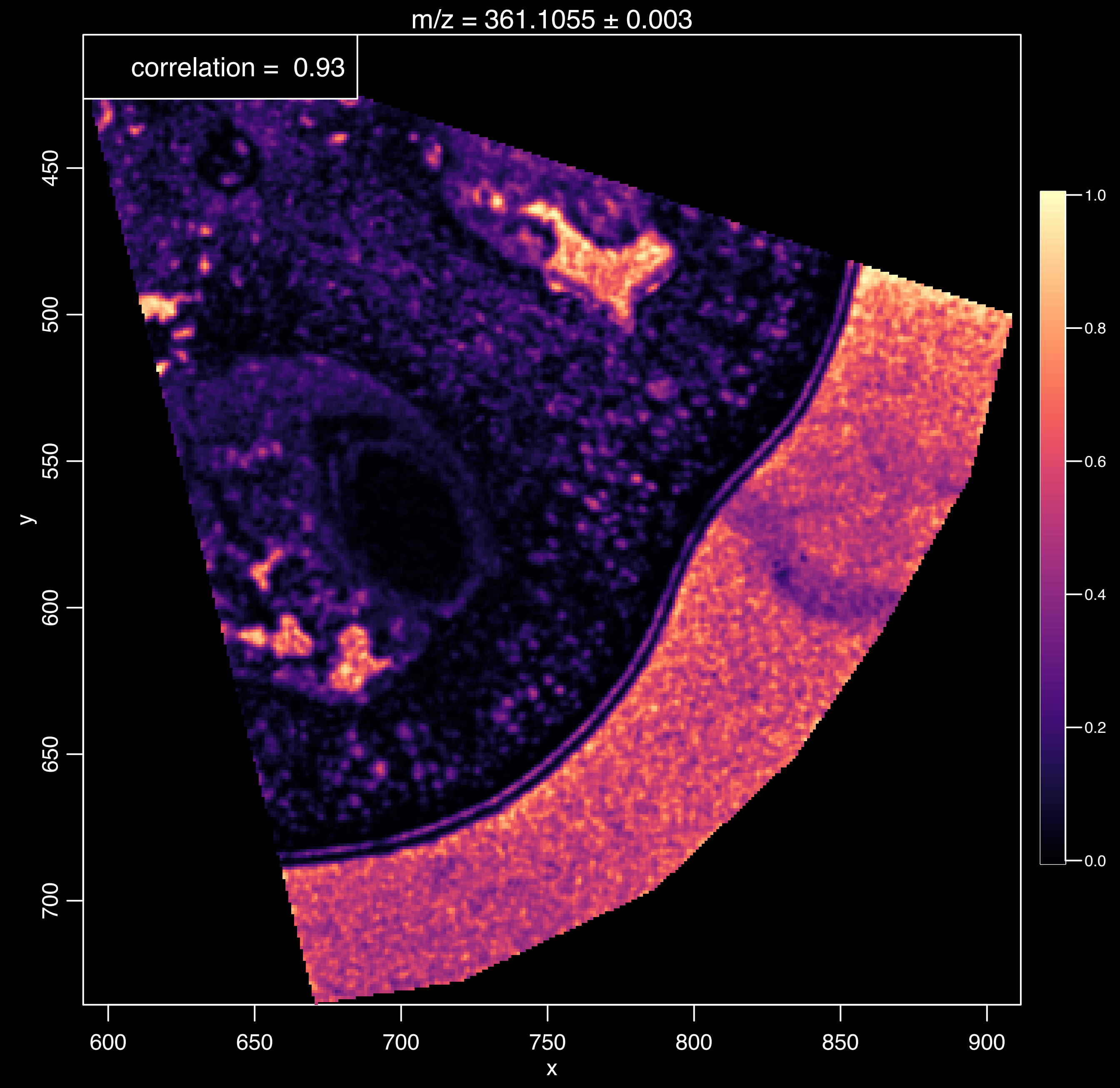

m/z = 361.1055 ± 0.003
correlation = 0.93
y
450
500
550
600
650
700
x
600
650
700
750
800
850
900
1.0
0.8
0.6
0.4
0.2
0.0

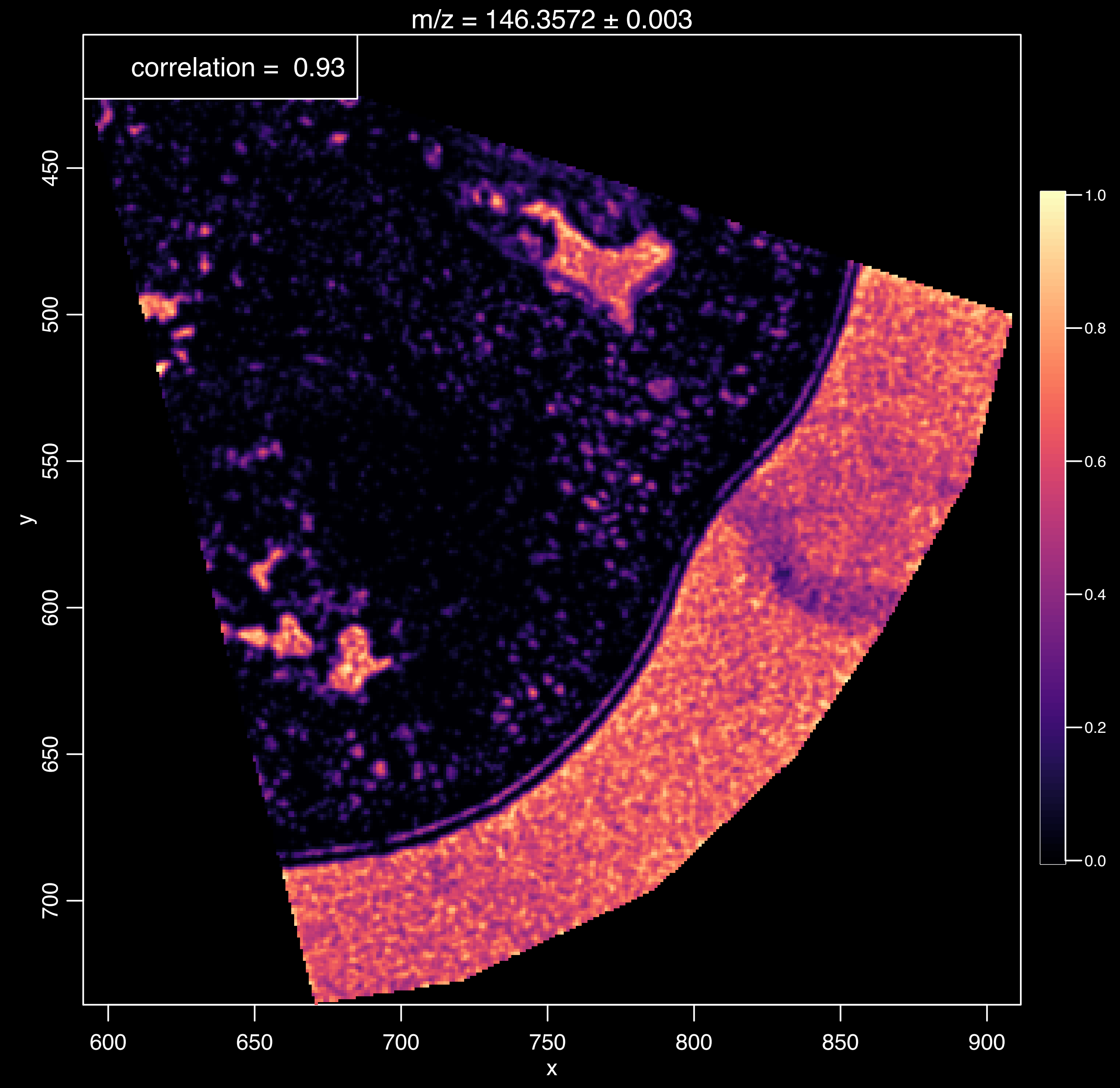

m/z = 146.3572 ± 0.003
correlation = 0.93
450
500
550
600
650
700
y
600
650
700
750
800
850
900
x
1.0
0.8
0.6
0.4
0.2
0.0

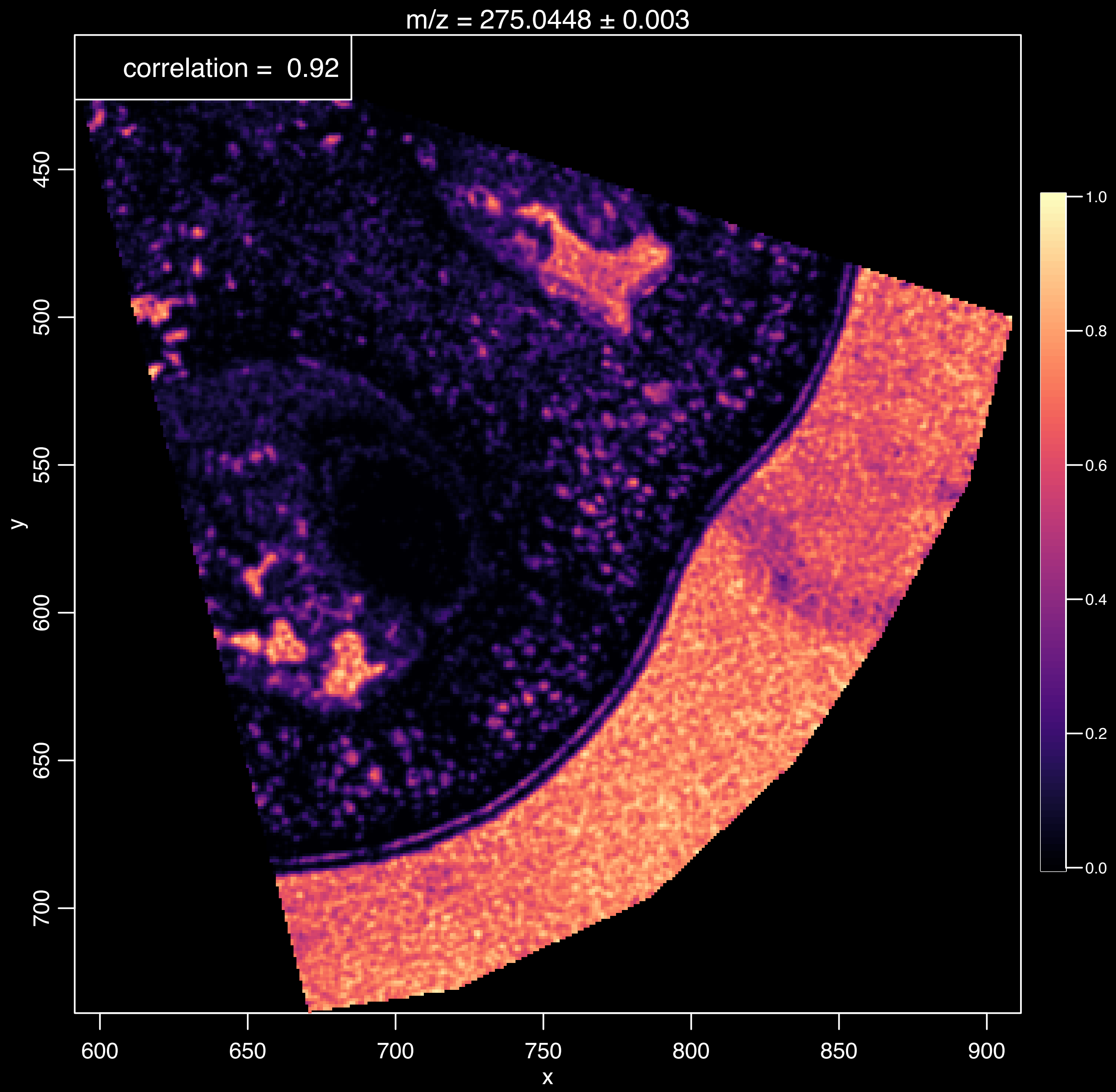

m/z = 275.0448 ± 0.003
correlation = 0.92
450
500
550
600
650
700
y
600
650
700
750
800
850
900
x
1.0
0.8
0.6
0.4
0.2
0.0

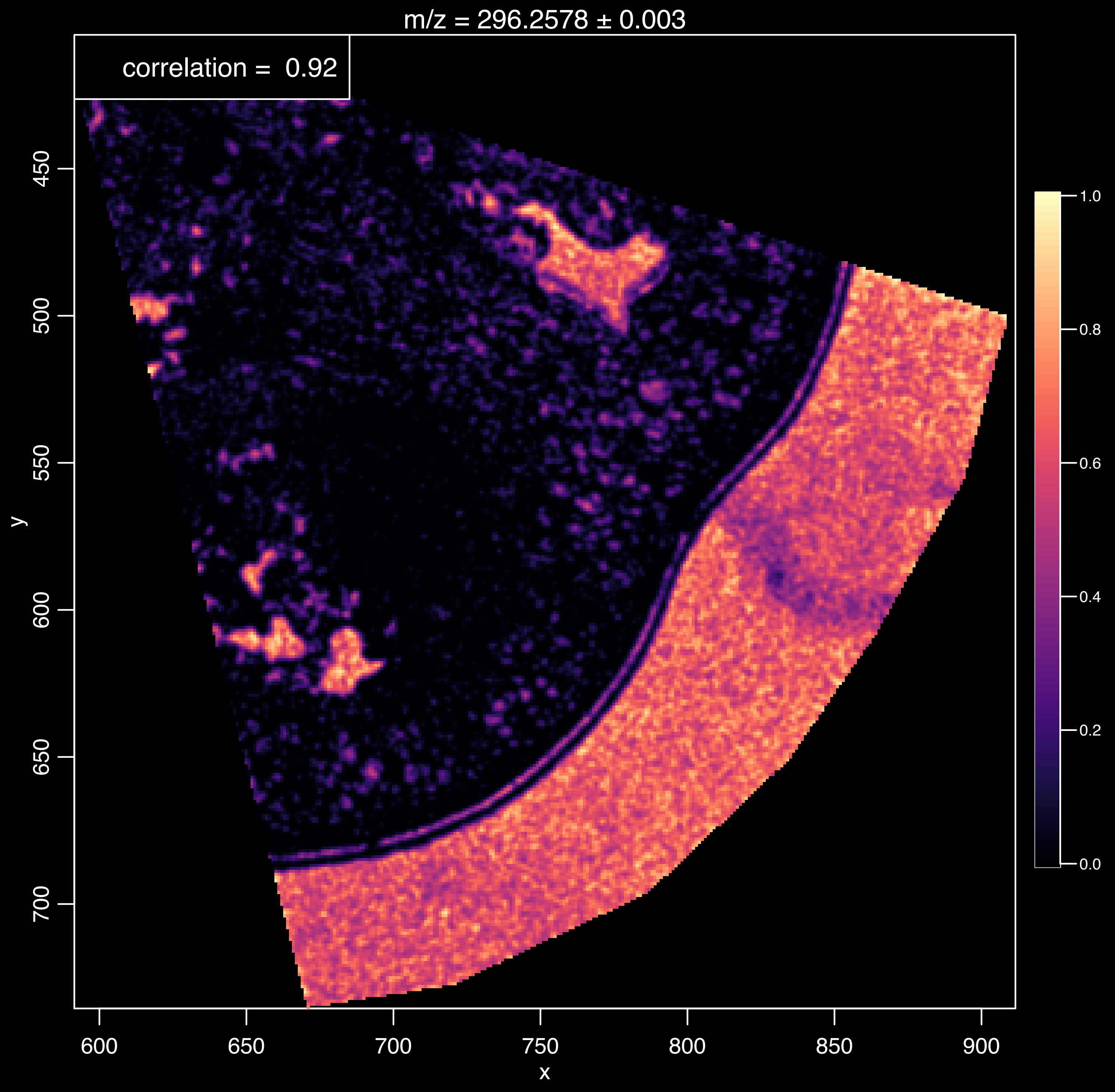

m/z = 296.2578 ± 0.003
correlation = 0.92
y
x
450
500
550
600
650
700
600
650
700
750
800
850
900
1.0
0.8
0.6
0.4
0.2
0.0

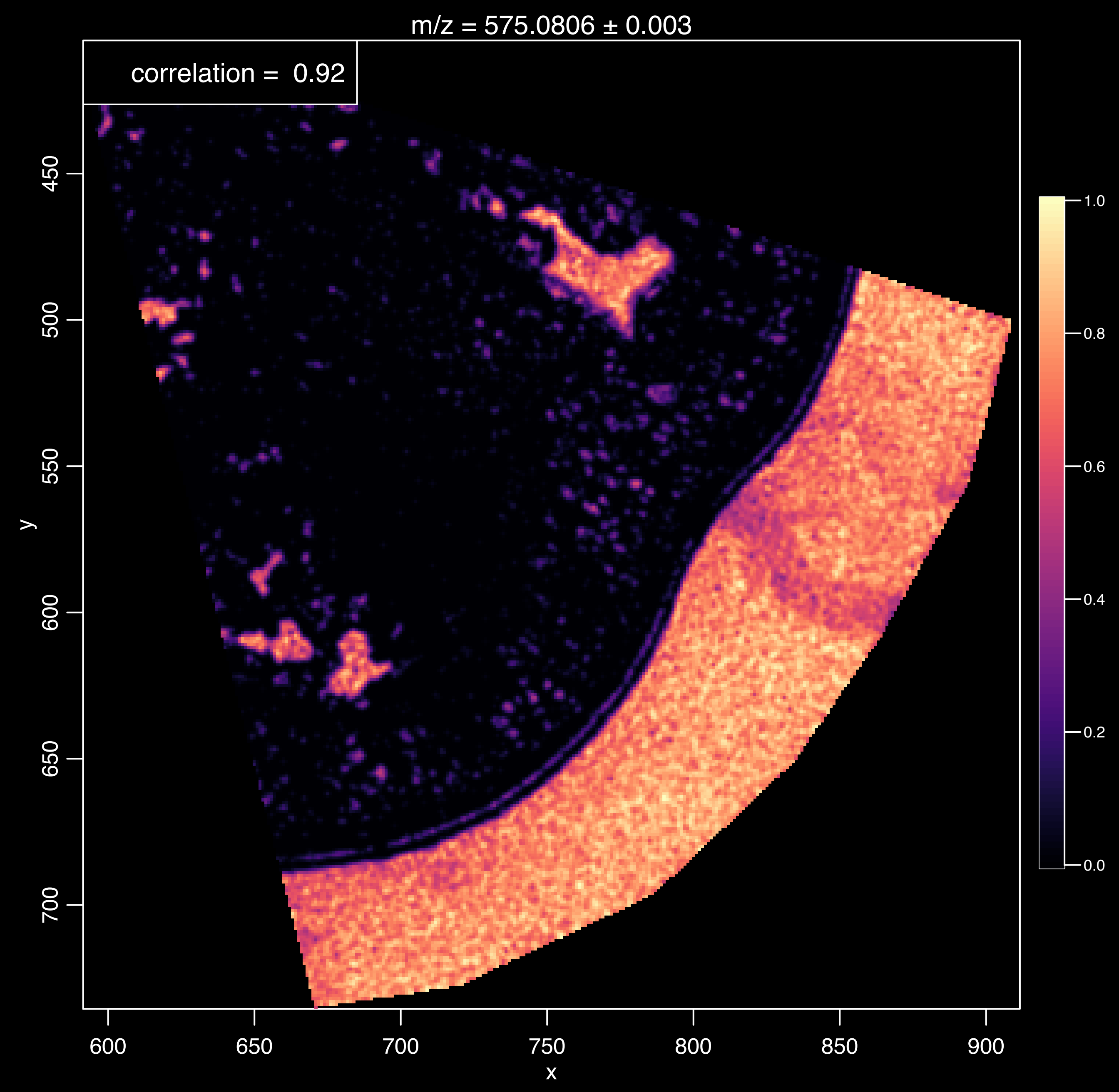

m/z = 575.0806 ± 0.003
correlation = 0.92
450
500
550
600
650
700
y
600
650
700
750
800
850
900
x
1.0
0.8
0.6
0.4
0.2
0.0

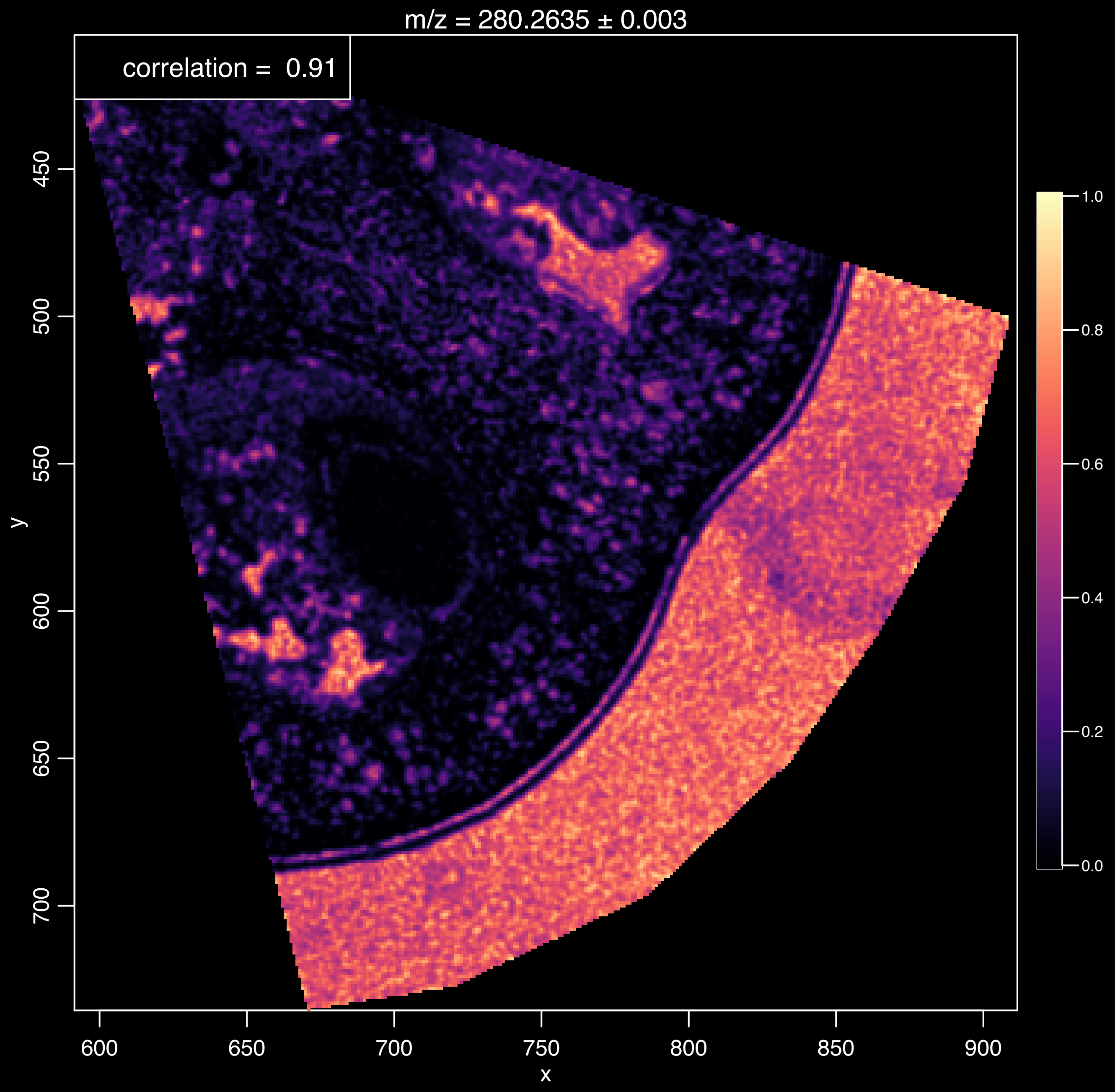

m/z = 280.2635 ± 0.003
correlation = 0.91
450
500
550
600
650
700
y
600
650
700
750
800
850
900
x
1.0
0.8
0.6
0.4
0.2
0.0

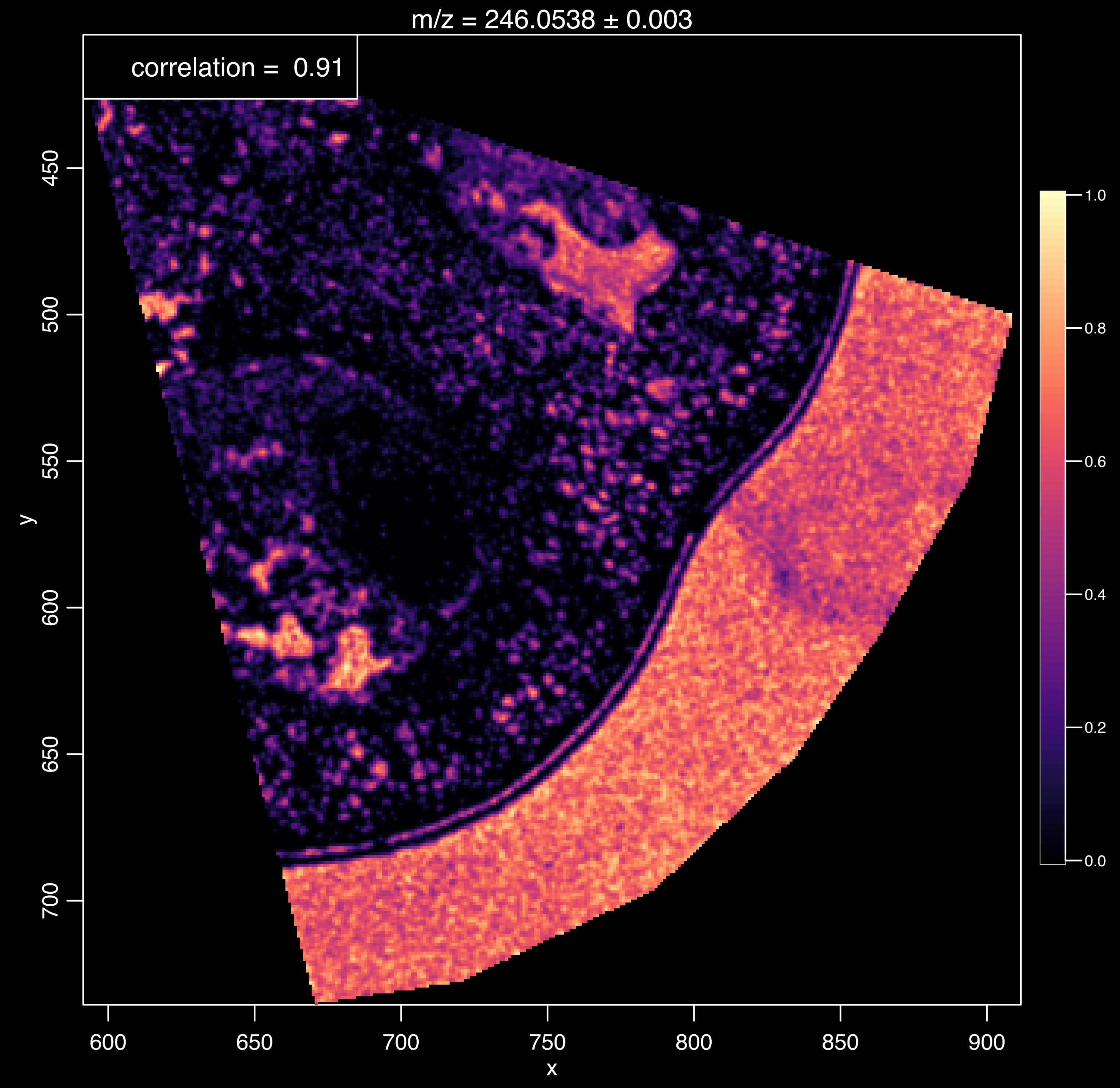

m/z = 246.0538 ± 0.003
correlation = 0.91
450
500
550
600
650
700
y
600
650
700
750
800
850
900
x
1.0
0.8
0.6
0.4
0.2
0.0

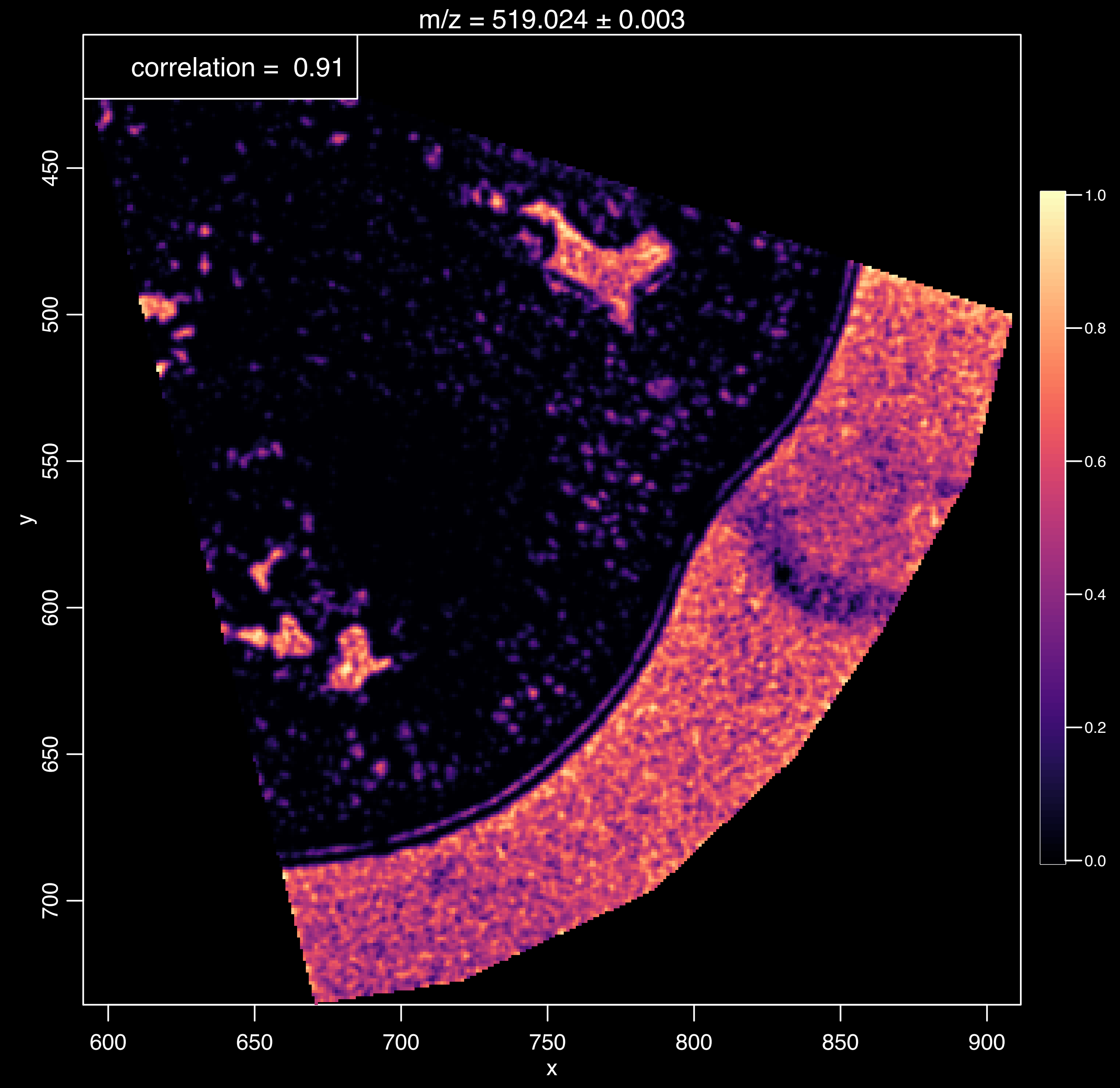
m/z = 519.024 ± 0.003
correlation = 0.91
450
500
550
600
650
700
y
600
650
700
750
800
850
900
x
1.0
0.8
0.6
0.4
0.2
0.0

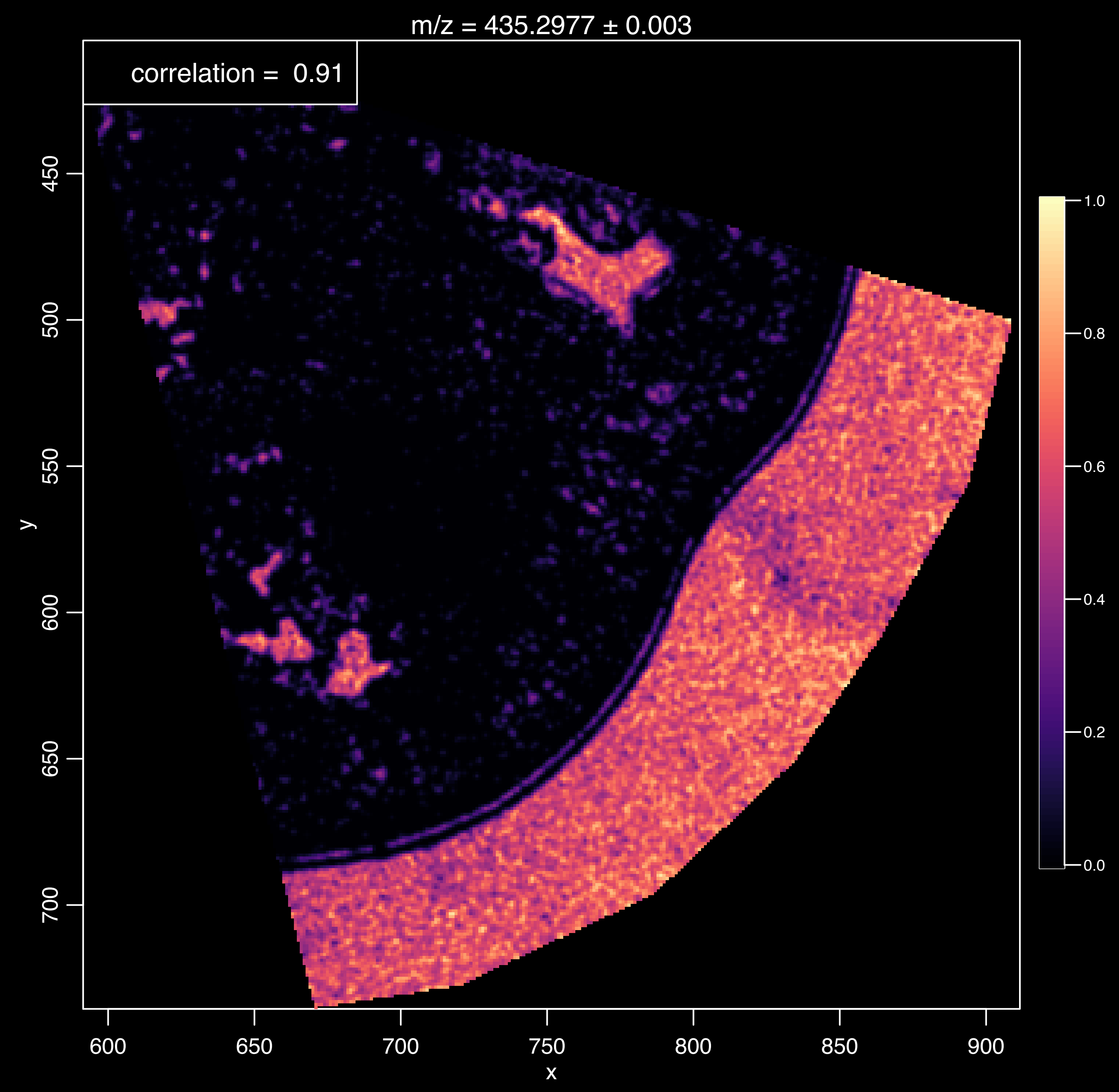

m/z = 435.2977 ± 0.003
correlation = 0.91
450
500
550
600
650
700
y
600
650
700
750
800
850
900
x
1.0
0.8
0.6
0.4
0.2
0.0

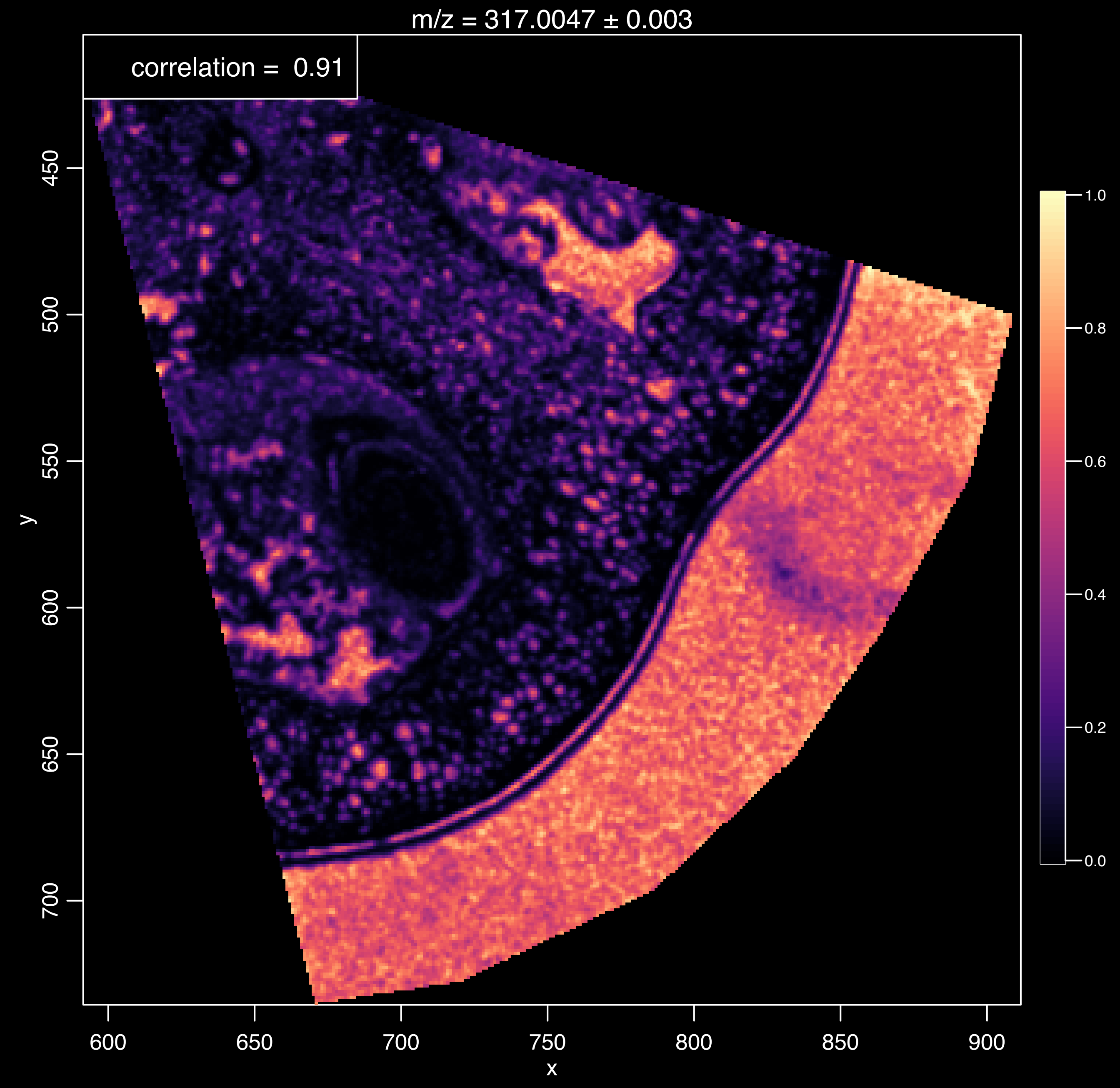

m/z = 317.0047 ± 0.003
correlation = 0.91
450
500
550
600
650
700
y
600
650
700
750
800
850
900
x
1.0
0.8
0.6
0.4
0.2
0.0

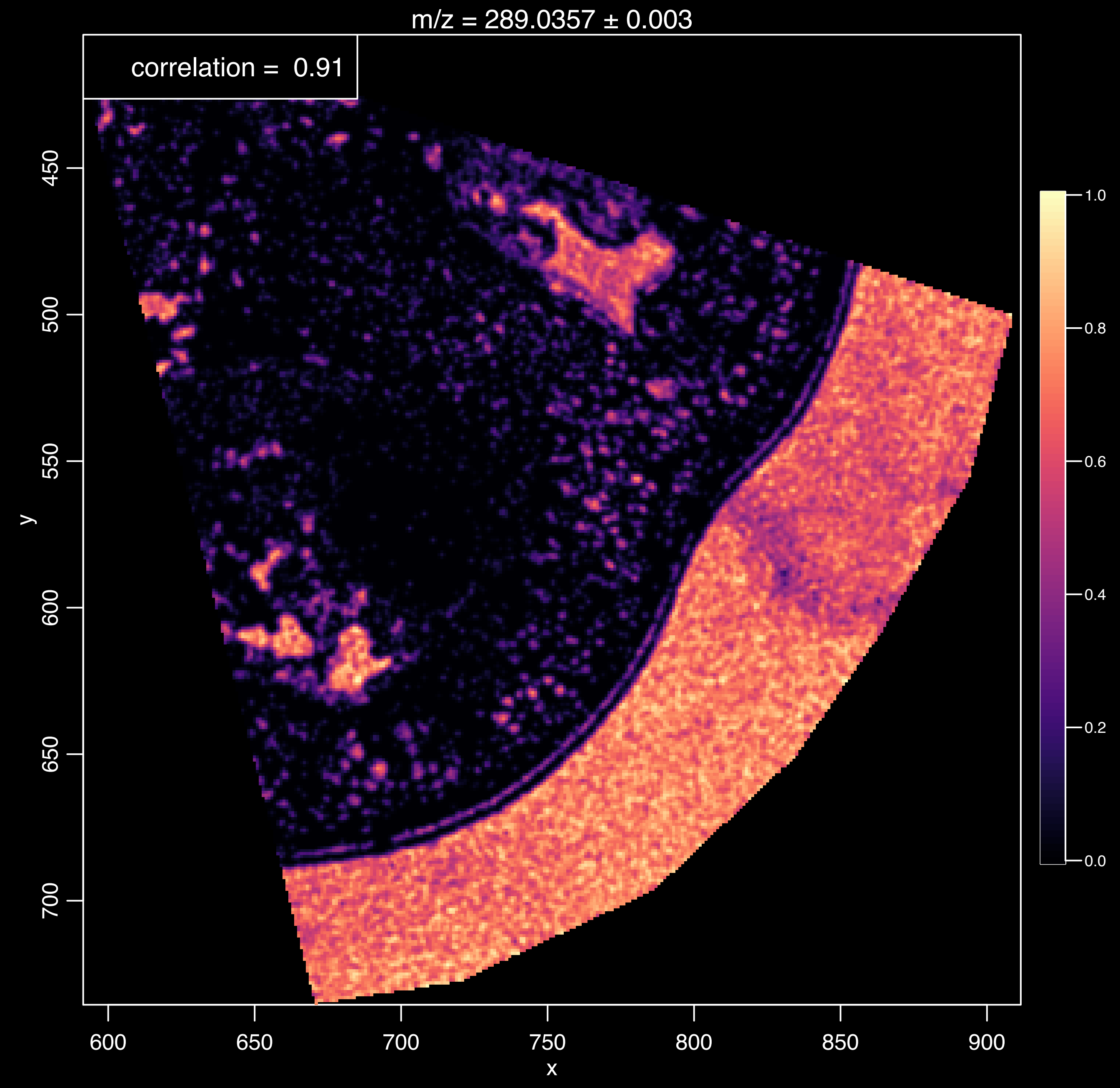

m/z = 289.0357 ± 0.003
correlation = 0.91
450
500
550
600
650
700
y
600
650
700
750
800
850
900
x
1.0
0.8
0.6
0.4
0.2
0.0

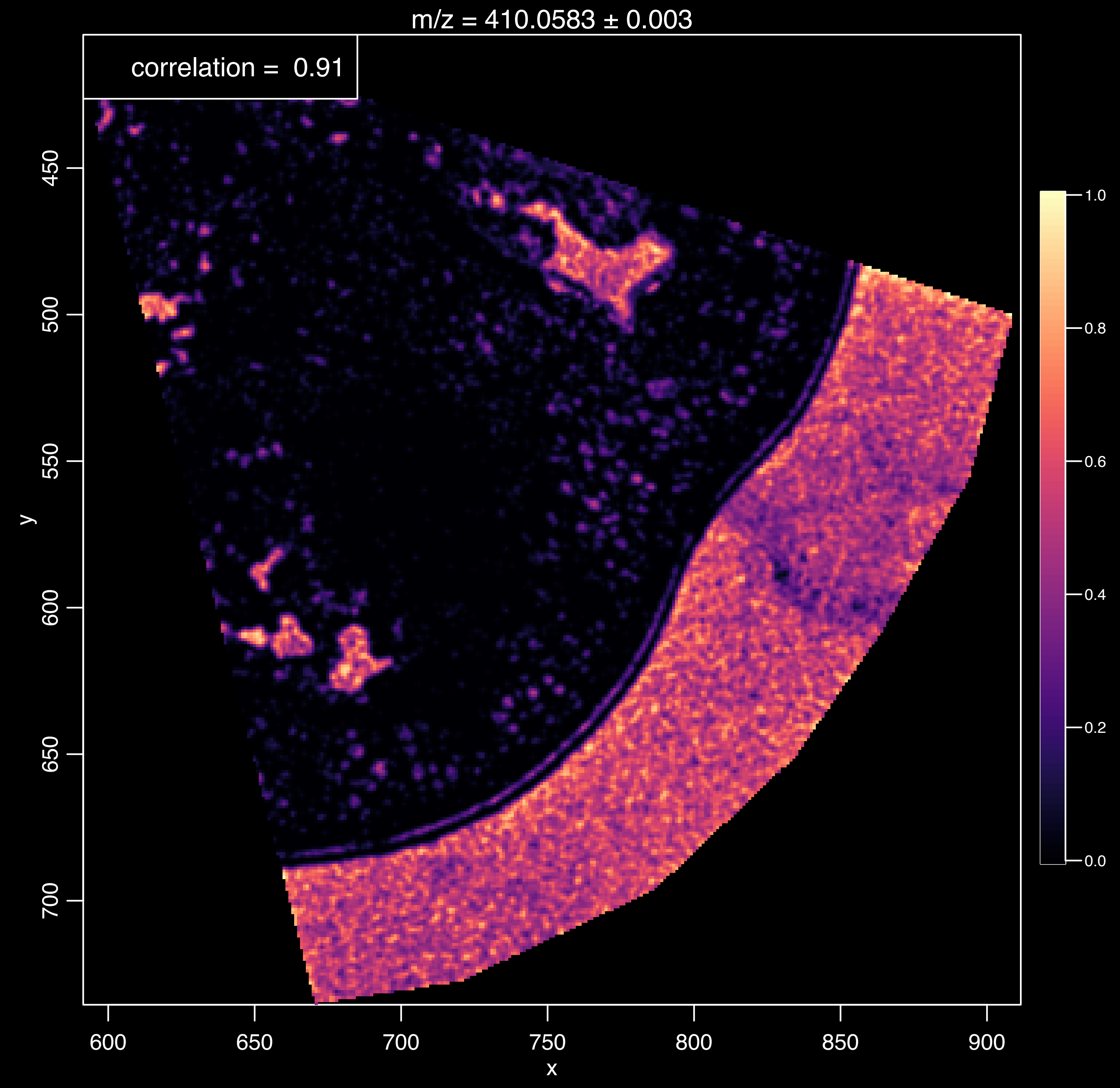
m/z = 410.0583 ± 0.003
correlation = 0.91
y
450
500
550
600
650
700
x
600
650
700
750
800
850
900
1.0
0.8
0.6
0.4
0.2
0.0

Table S2 The *m/z* values, PCC scores, annotation, and mass accuracies of the 6 ions colocalized with rutin peak *m/z* 611.161.

ID	Measured *m/z*	Identity	PCC score	Exact *m/z*	mass accuracy (ppm)
1	611.1611	$[M+H]^+$	1	611.1607	0.65
2	465.1029	Fragment: $[M+H\text{-}rhamnosyl]^+$	0.98		-0.31
3	303.0502	Fragment: $[M+H\text{-}glucosyl]^+$	0.95	303.0499	0.99
4	466.1056	13C isotope of 2	0.93	466.1062	-1.29
5	612.1645	13C isotope of 1	0.9	612.1641	0.65
6	633.1427	$[M+Na]^+$	0.9	633.1426	0.16
7	634.1475	13C isotope of 6	0.9	634.1460	2.37

Figure S2 MS images of ions highly colocalized (PCC score >= 0.9) with rutin peak *m/z* 611.161.

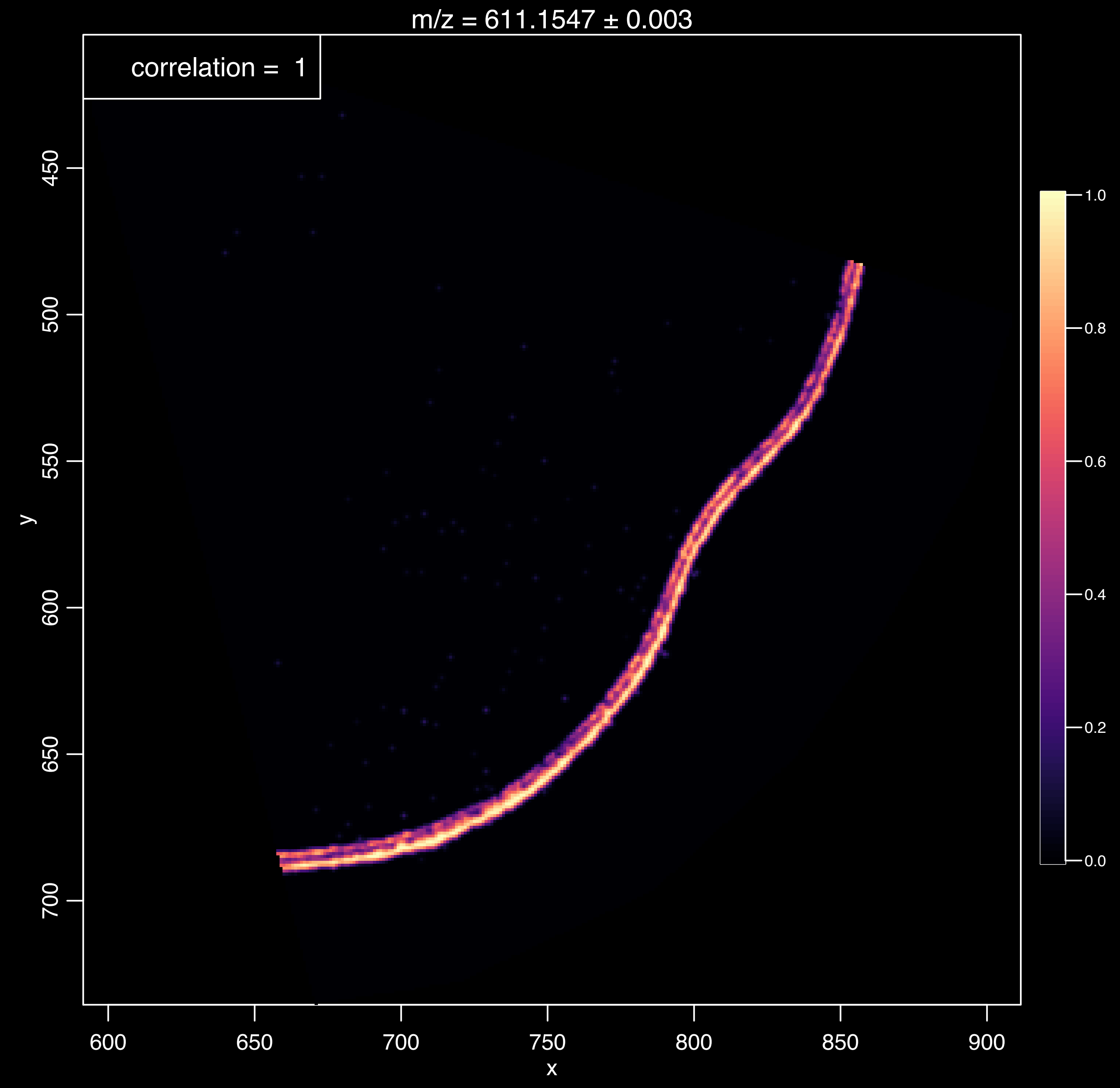

m/z = 611.1547 ± 0.003
correlation = 1
450
500
550
600
650
700
y
600
650
700
750
800
850
900
x
1.0
0.8
0.6
0.4
0.2
0.0

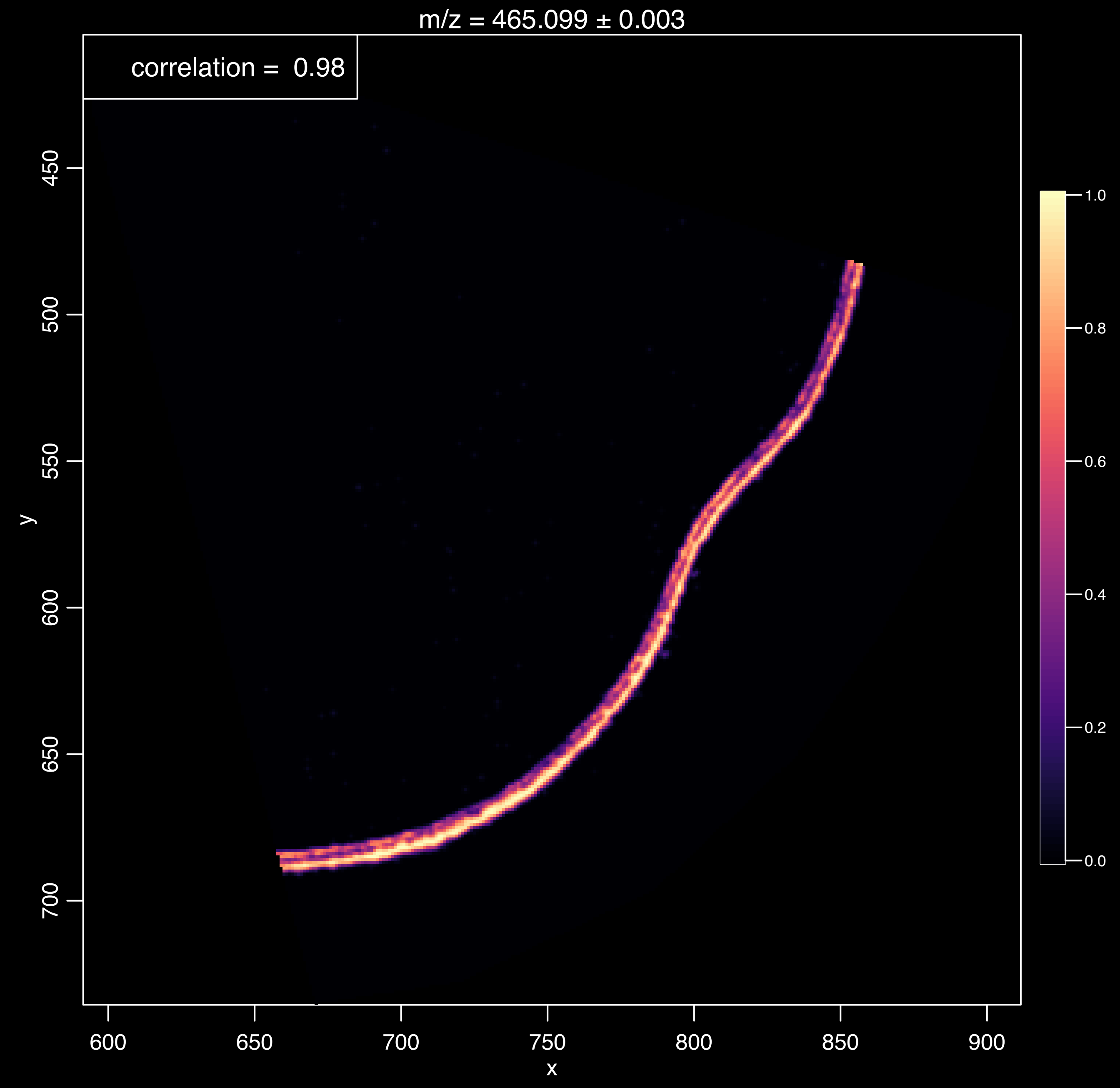

m/z = 465.099 ± 0.003
correlation = 0.98
450
500
550
600
650
700
y
600
650
700
750
800
850
900
x
1.0
0.8
0.6
0.4
0.2
0.0

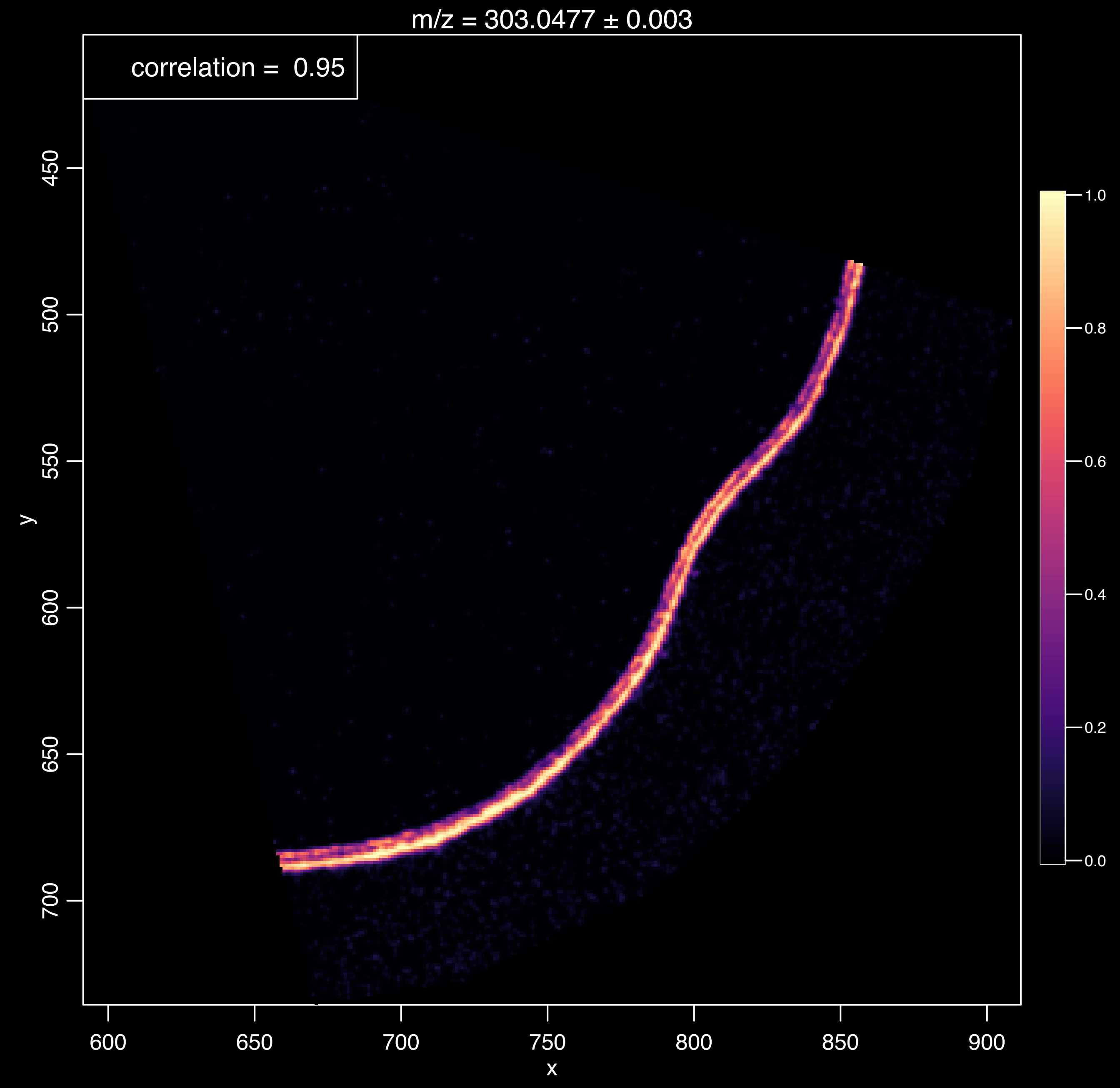

m/z = 303.0477 ± 0.003
correlation = 0.95
450
500
550
600
650
700
y
600
650
700
750
800
850
900
x
1.0
0.8
0.6
0.4
0.2
0.0

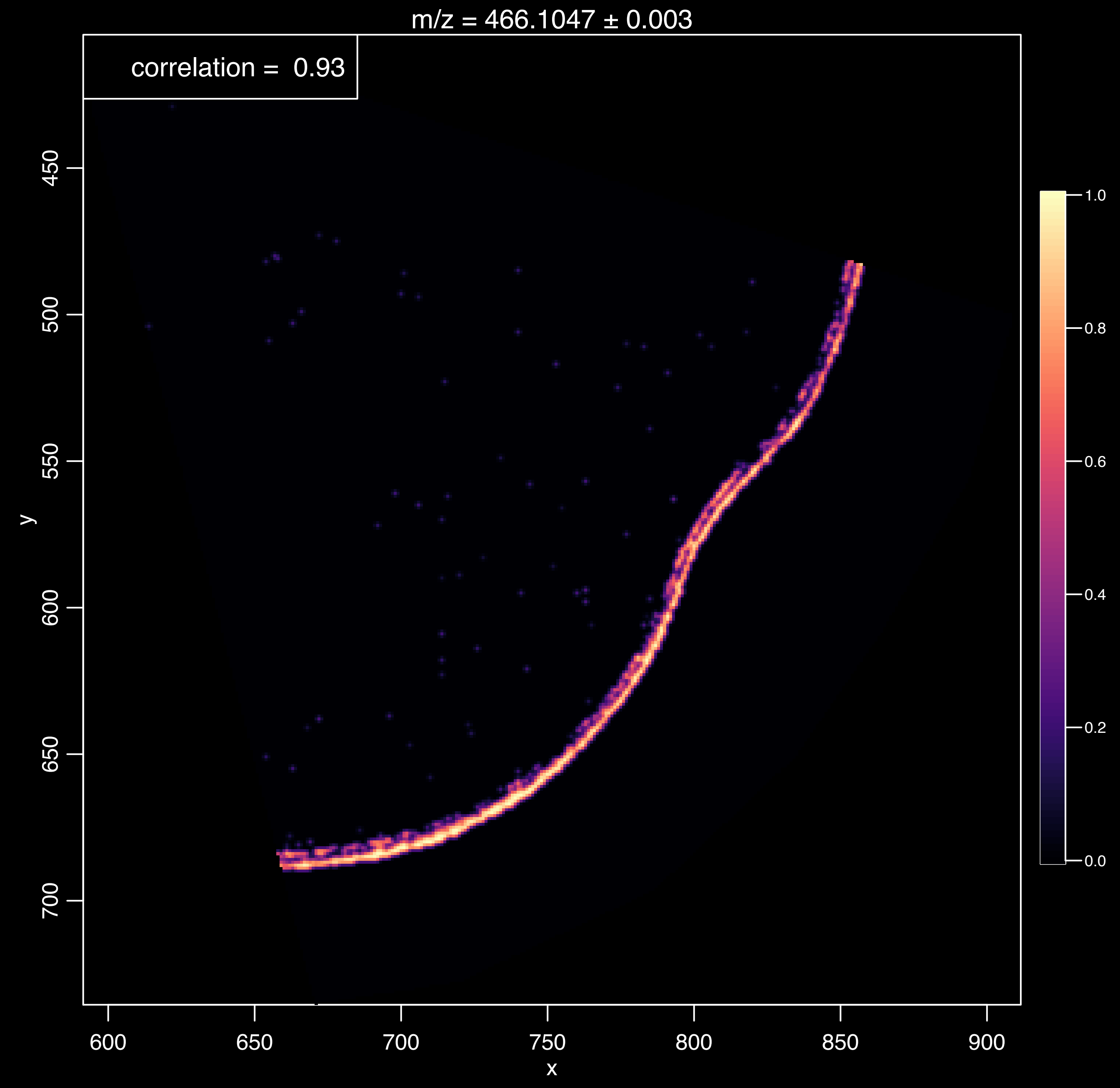

m/z = 466.1047 ± 0.003
correlation = 0.93
450
500
550
600
650
700
y
600
650
700
750
800
850
900
x
1.0
0.8
0.6
0.4
0.2
0.0

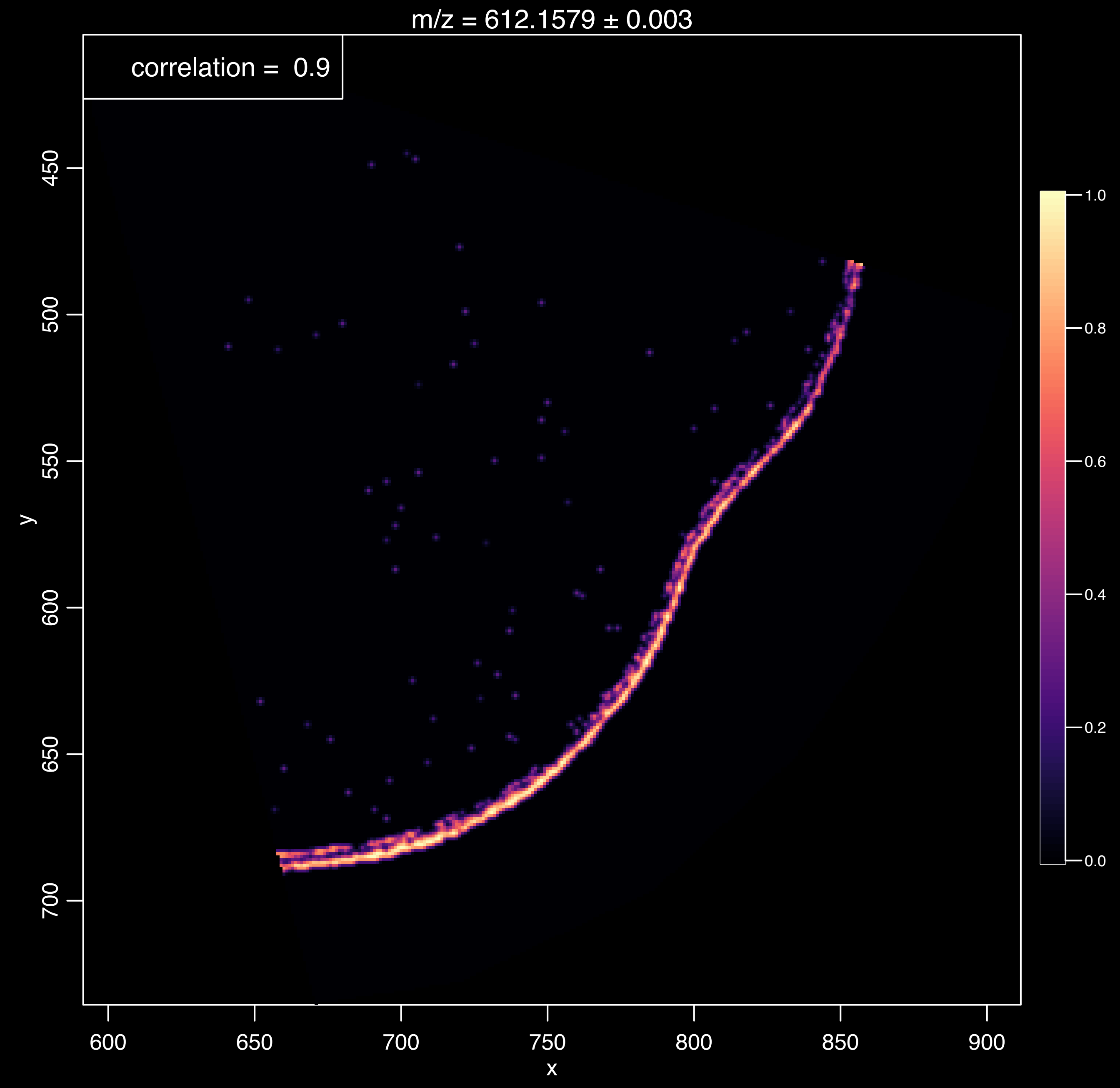

m/z = 612.1579 ± 0.003
correlation = 0.9
450
500
550
600
650
700
y
600
650
700
750
800
850
900
x
1.0
0.8
0.6
0.4
0.2
0.0

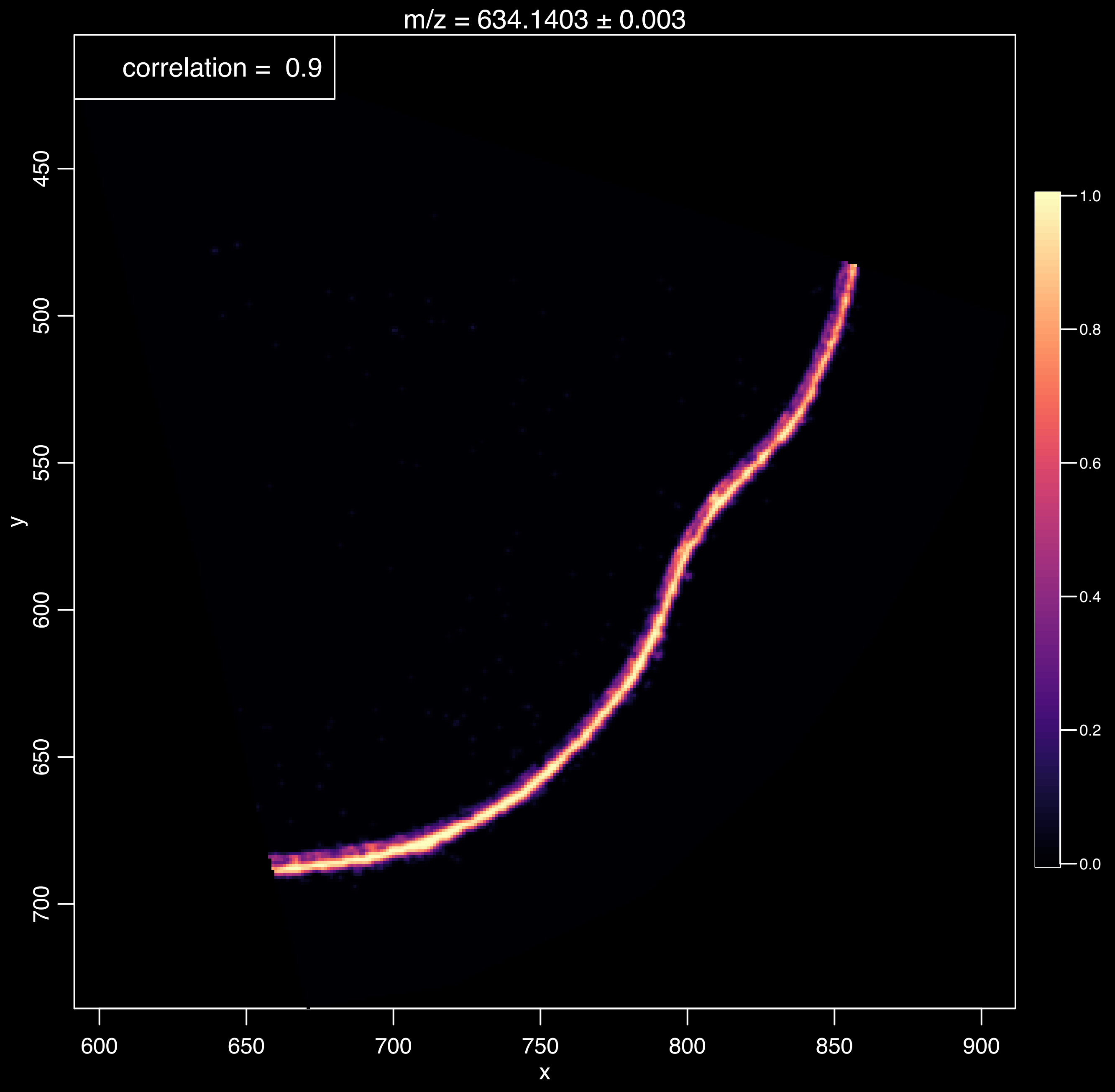

m/z = 634.1403 ± 0.003
correlation = 0.9
y
450
500
550
600
650
700
x
600
650
700
750
800
850
900
1.0
0.8
0.6
0.4
0.2
0.0

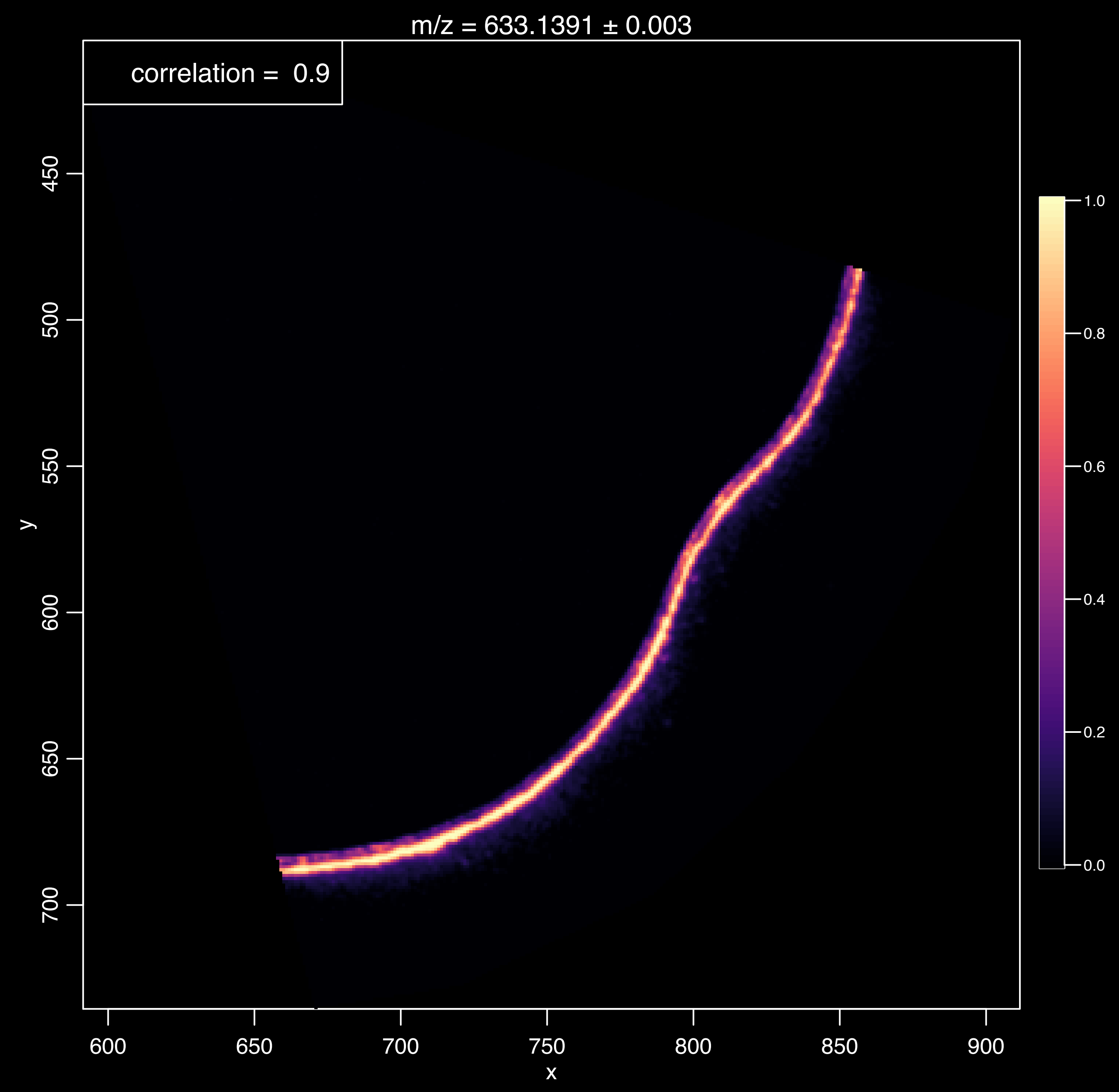

m/z = 633.1391 ± 0.003
correlation = 0.9
y
450
500
550
600
650
700
x
600
650
700
750
800
850
900
1.0
0.8
0.6
0.4
0.2
0.0

Figure S3

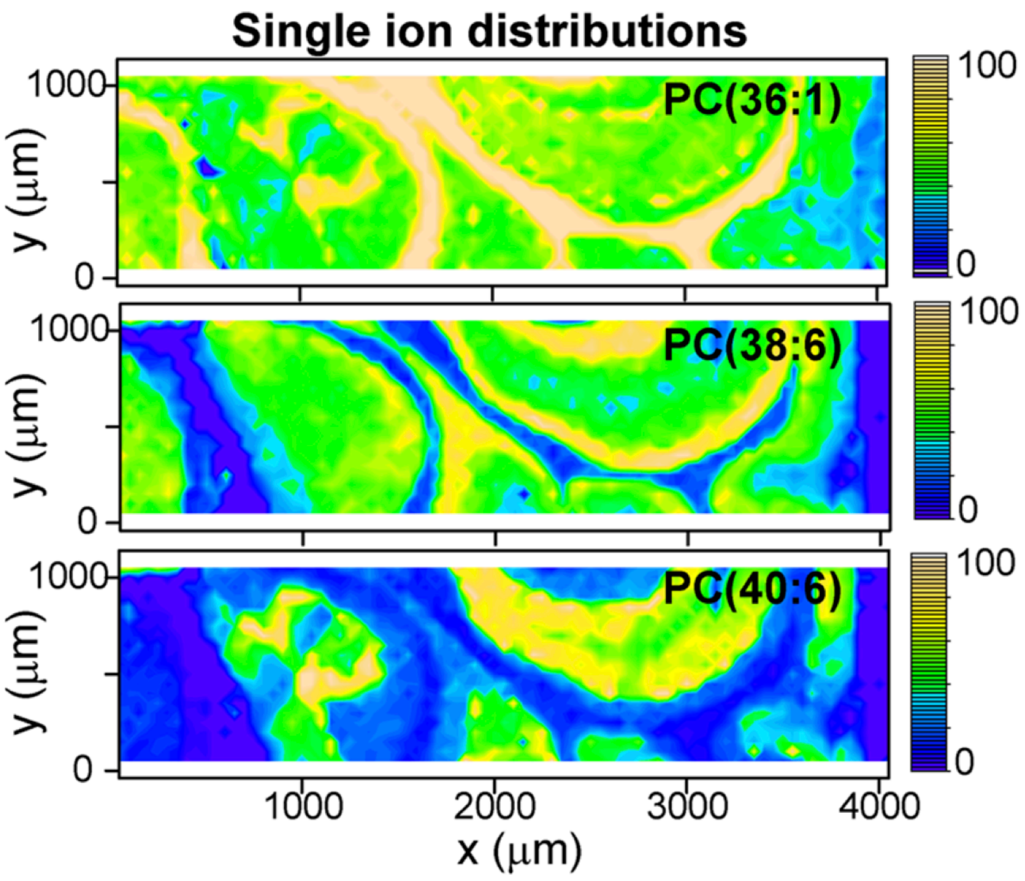

Figure S3 MALDI imaging of mouse cerebellum. MALDI images for $[PC(36:1)+K]^+$, $[PC(38:6)+K]^+$, and $[PC(40:6)+K]^+$ show that they are predominantly located in white matter, granular layer, and the molecular layer, respectively. Reprinted (adapted) from Bond et al. (2019) [1]with permission.

Figure S4 MS images of ions moderately colocalized (0.9> PCC score >= 0.6) with rutin peak *m/z* 611.161.

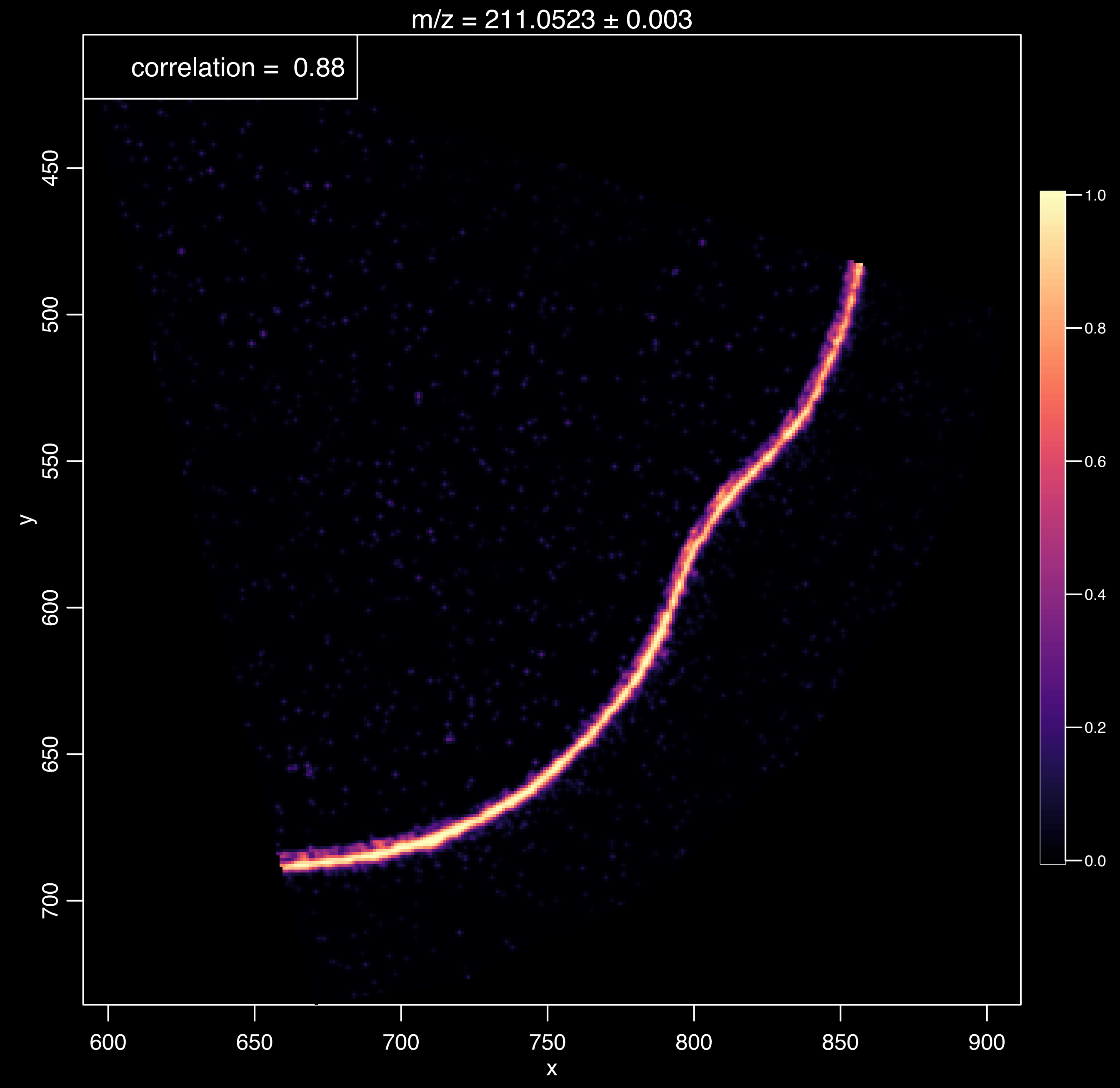
m/z = 211.0523 ± 0.003
correlation = 0.88
450
500
550
600
650
700
y
600
650
700
750
800
850
900
x
1.0
0.8
0.6
0.4
0.2
0.0

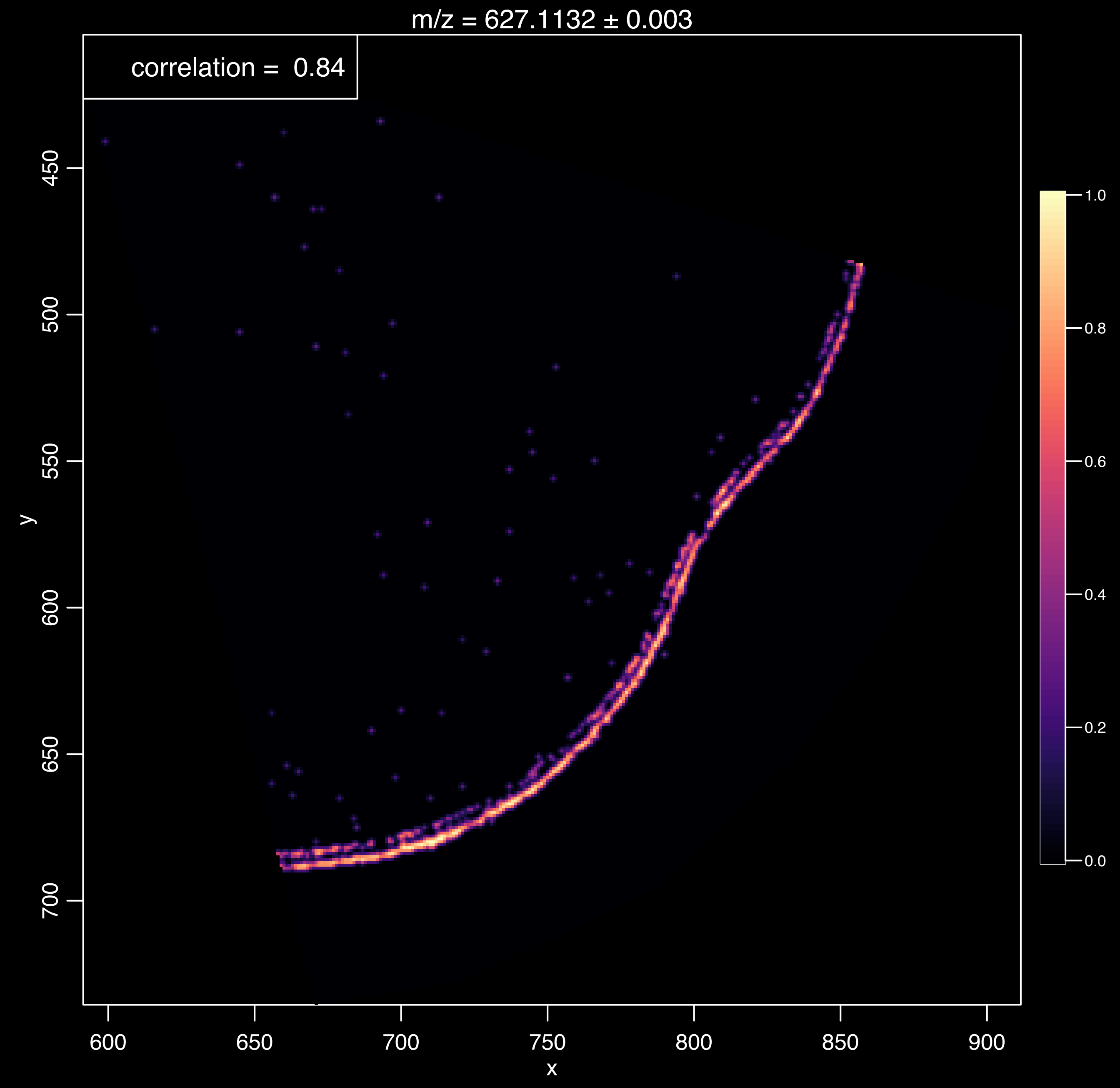

m/z = 627.1132 ± 0.003
correlation = 0.84
y
450
500
550
600
650
700
x
600
650
700
750
800
850
900
1.0
0.8
0.6
0.4
0.2
0.0

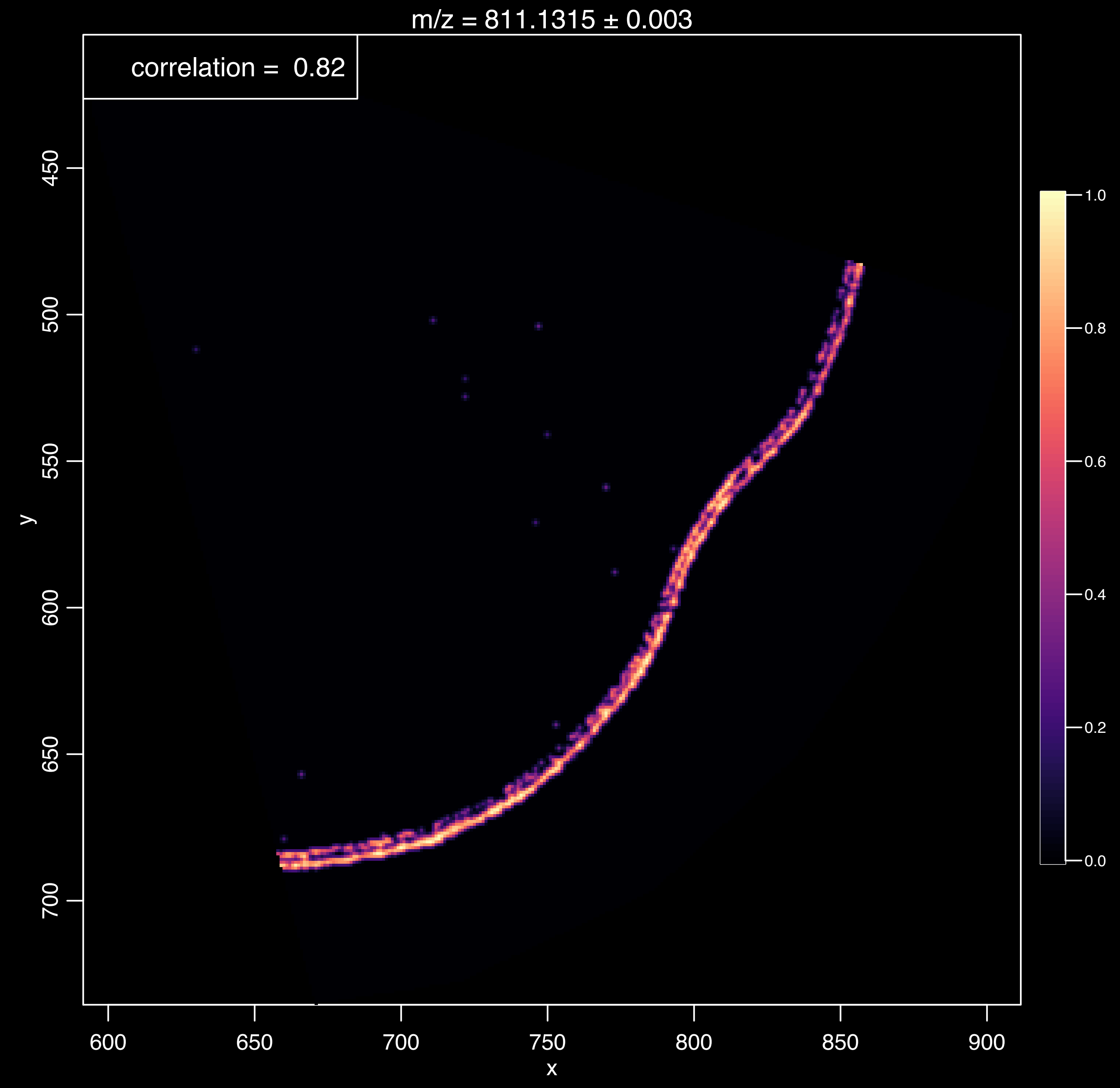

m/z = 811.1315 ± 0.003
correlation = 0.82
450
500
550
600
650
700
y
600
650
700
750
800
850
900
x
1.0
0.8
0.6
0.4
0.2
0.0

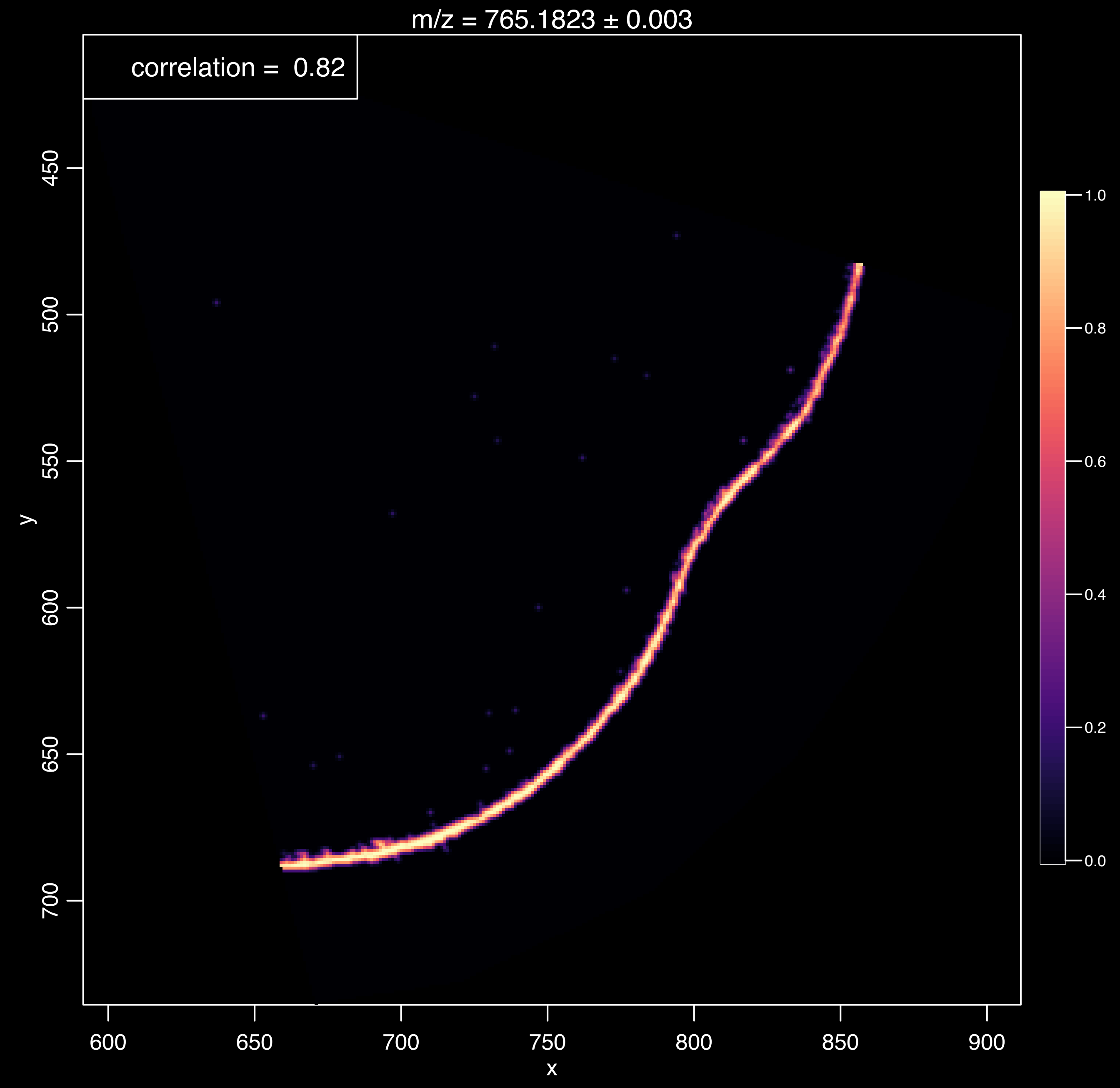

m/z = 765.1823 ± 0.003
correlation = 0.82
y
450
500
550
600
650
700
x
600
650
700
750
800
850
900
1.0
0.8
0.6
0.4
0.2
0.0

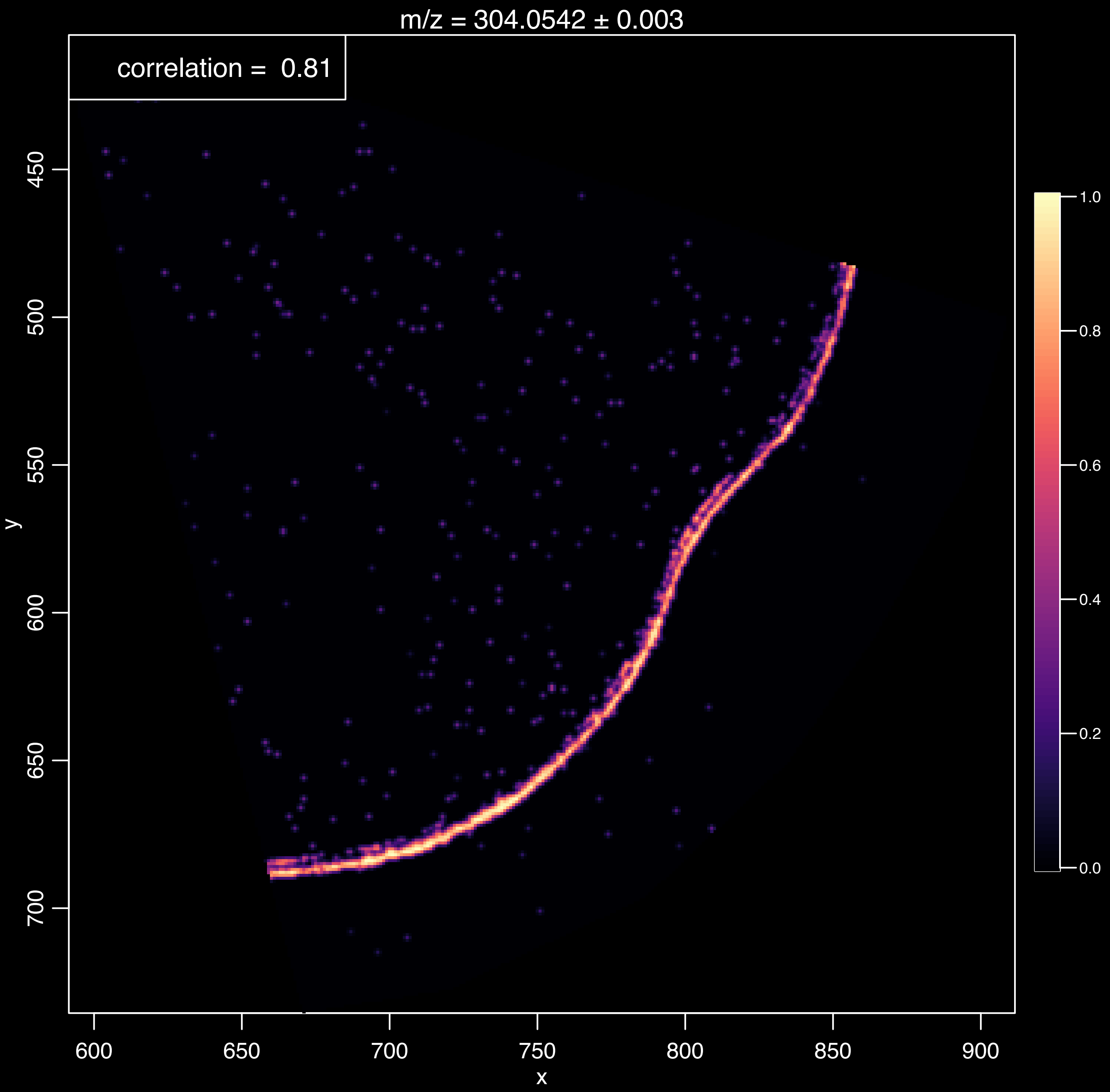
m/z = 304.0542 ± 0.003
correlation = 0.81
y
x
450
500
550
600
650
700
600
650
700
750
800
850
900
1.0
0.8
0.6
0.4
0.2
0.0

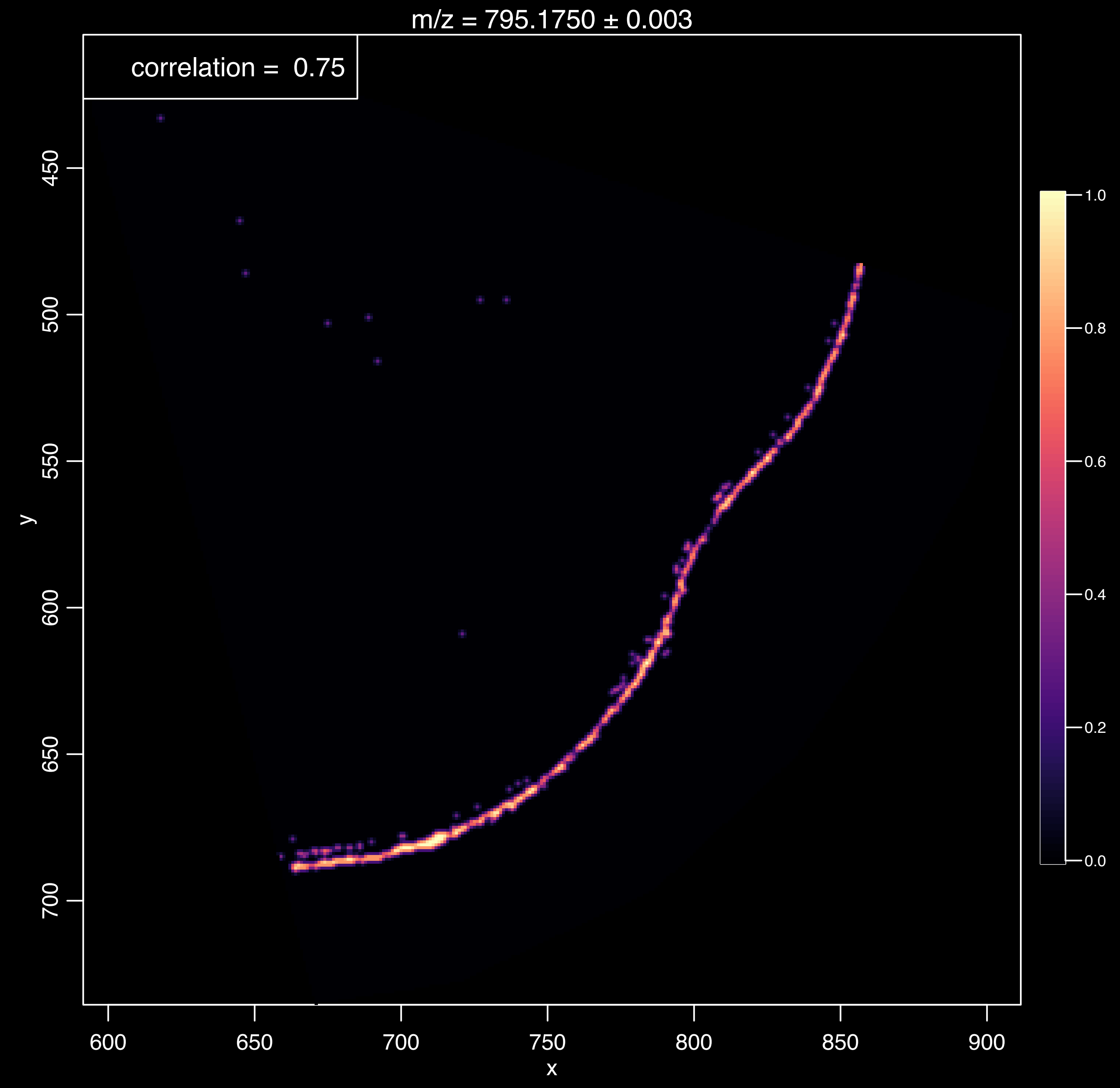

m/z = 795.1750 ± 0.003
correlation = 0.75
450
500
550
600
650
700
y
600
650
700
750
800
850
900
x
1.0
0.8
0.6
0.4
0.2
0.0

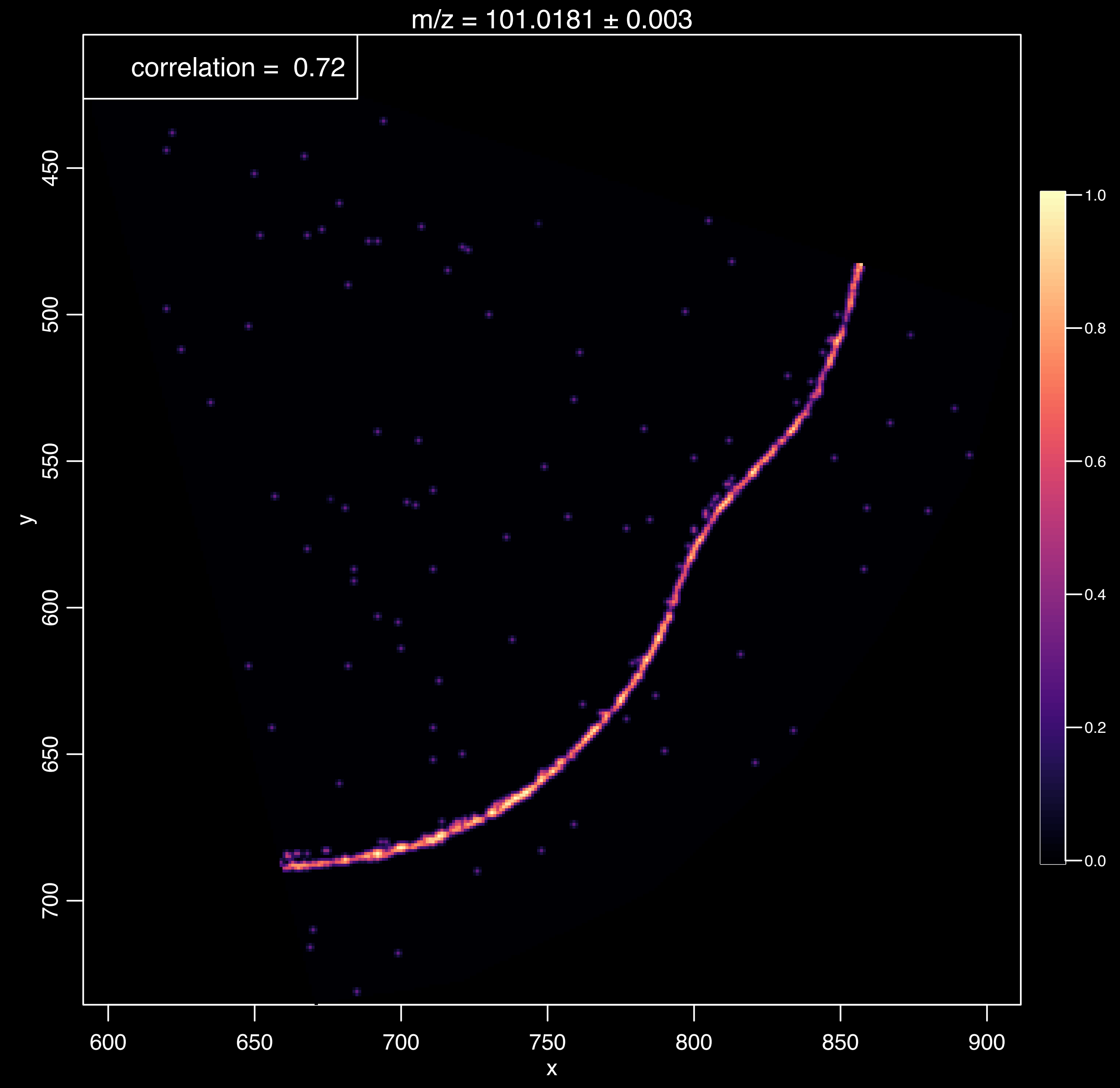

m/z = 101.0181 ± 0.003
correlation = 0.72
y
x
450
500
550
600
650
700
600
650
700
750
800
850
900
0.0
0.2
0.4
0.6
0.8
1.0

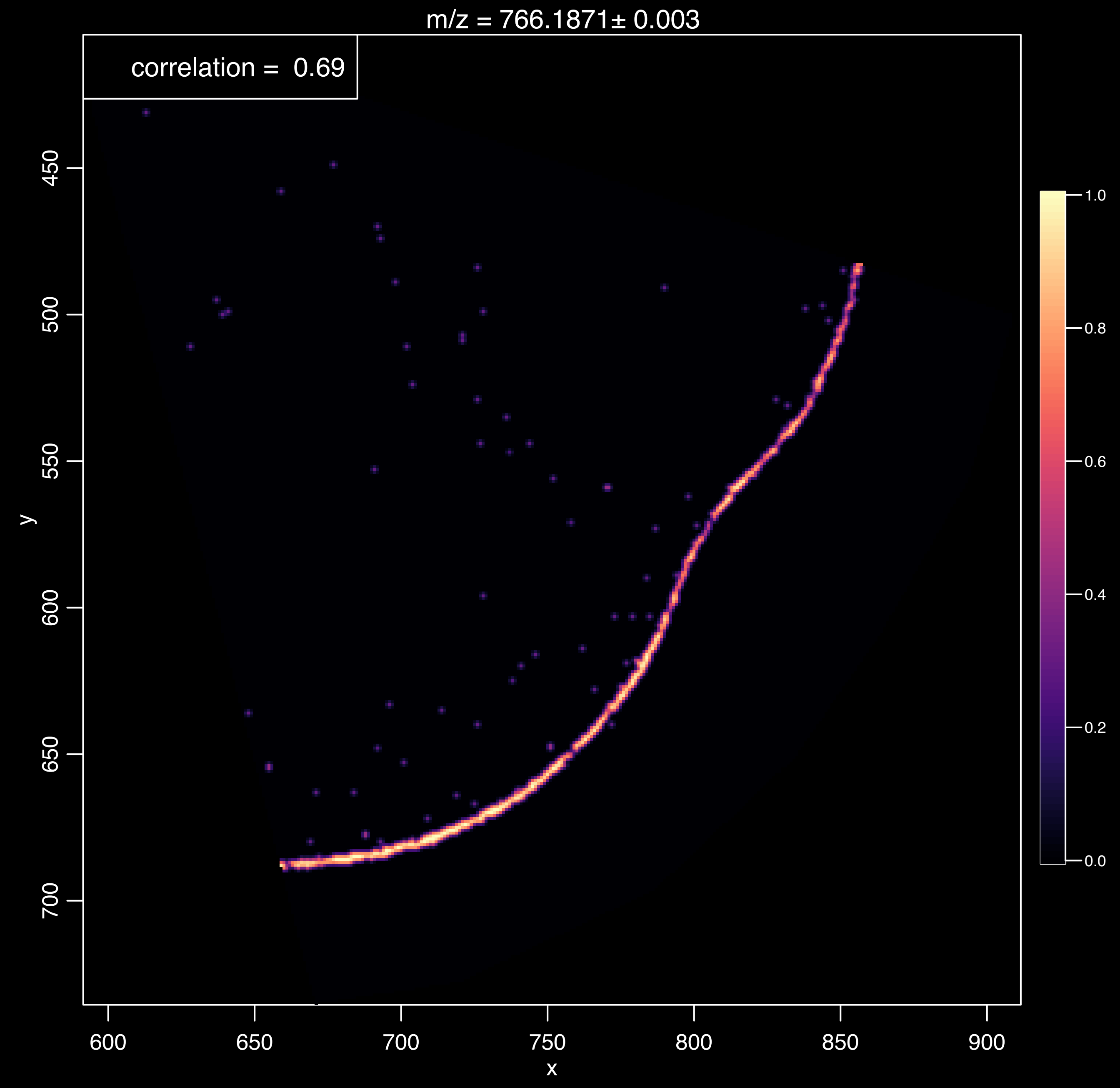

m/z = 766.1871± 0.003
correlation = 0.69
y
450
500
550
600
650
700
x
600
650
700
750
800
850
900
1.0
0.8
0.6
0.4
0.2
0.0

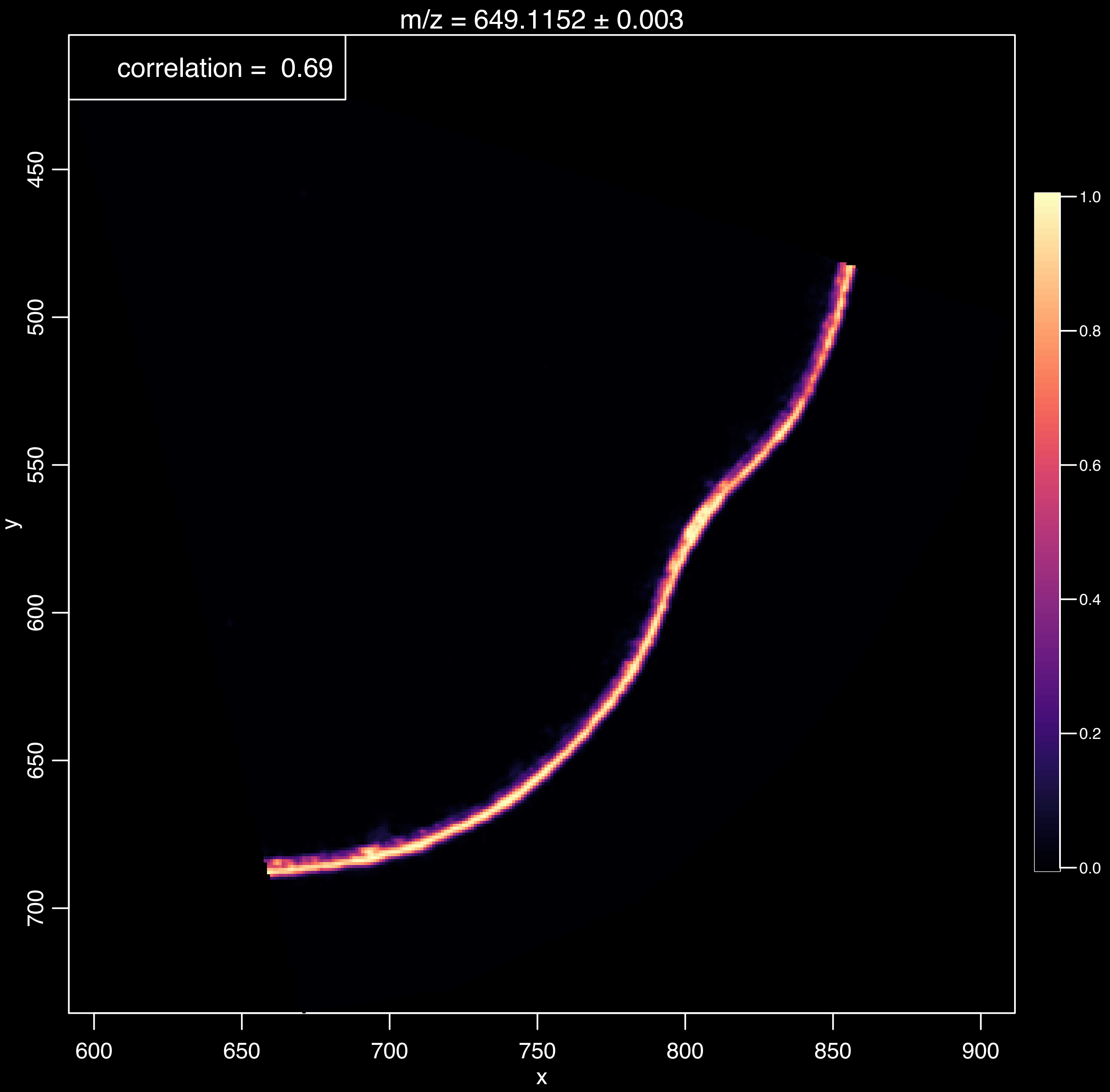
m/z = 649.1152 ± 0.003
correlation = 0.69
450
500
550
600
650
700
y
600
650
700
750
800
850
900
x
1.0
0.8
0.6
0.4
0.2
0.0

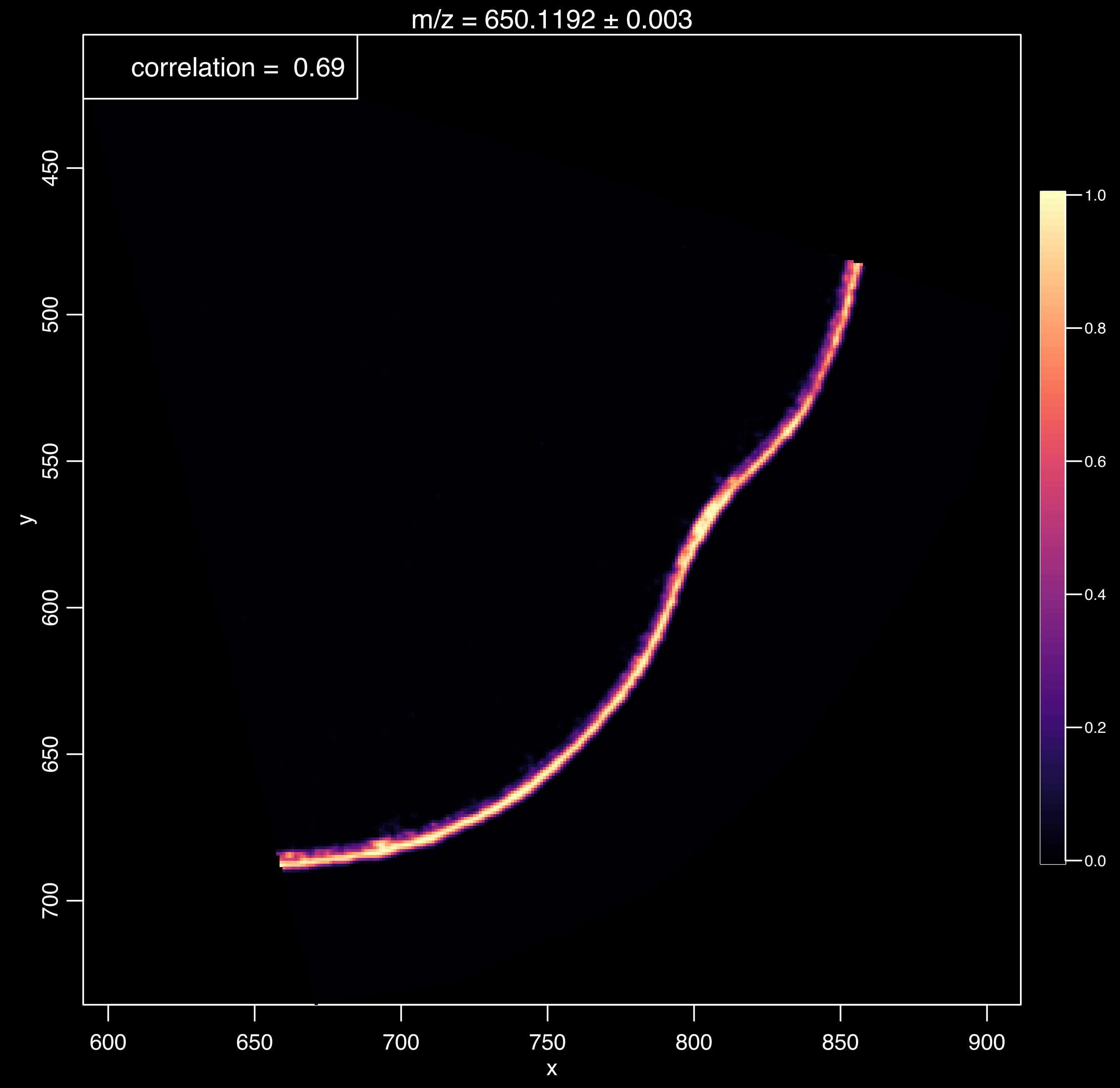

m/z = 650.1192 ± 0.003
correlation = 0.69
y
450
500
550
600
650
700
x
600
650
700
750
800
850
900
1.0
0.8
0.6
0.4
0.2
0.0

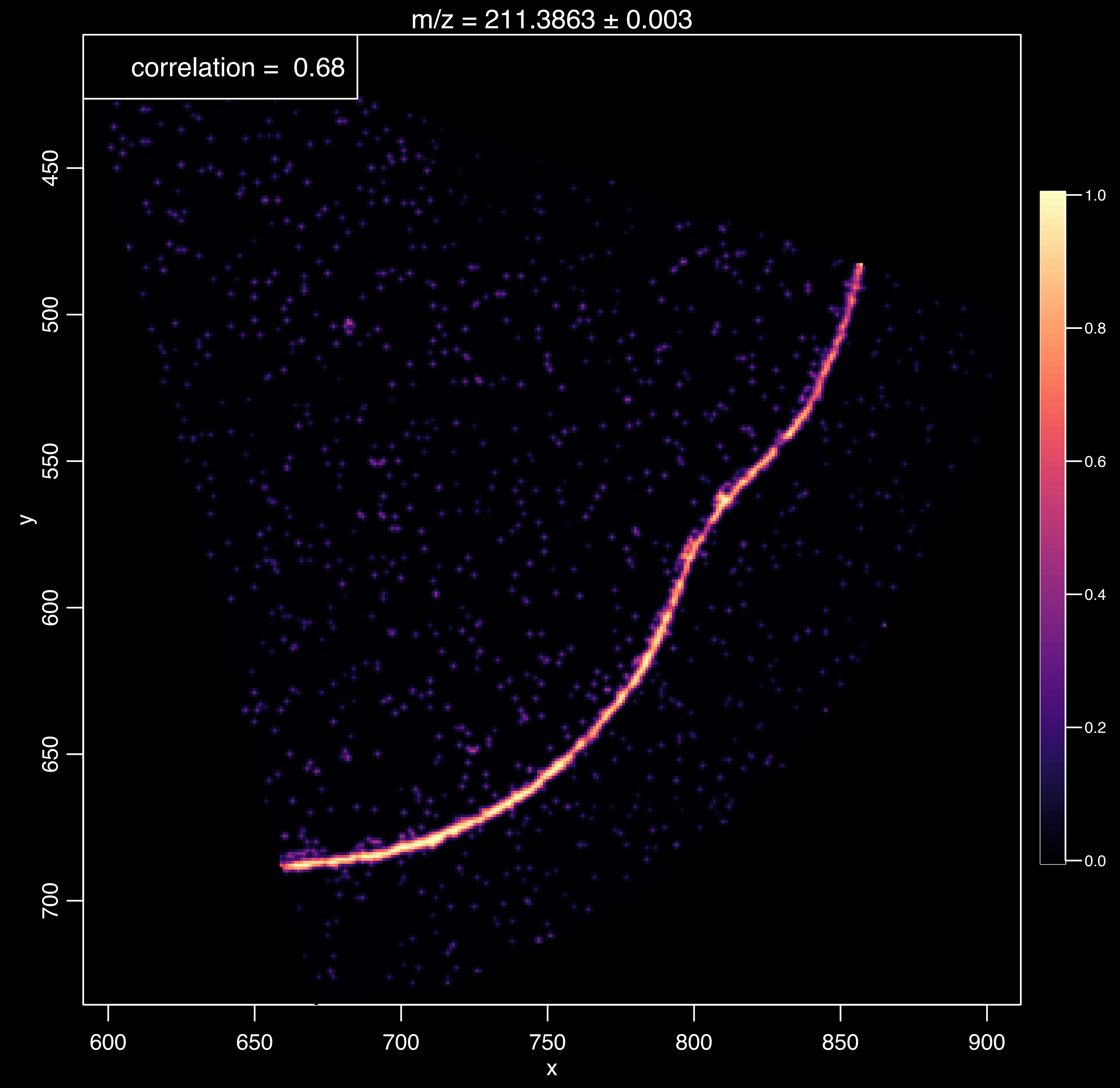

m/z = 211.3863 ± 0.003
correlation = 0.68
y
x
450
500
550
600
650
700
600
650
700
750
800
850
900
1.0
0.8
0.6
0.4
0.2
0.0

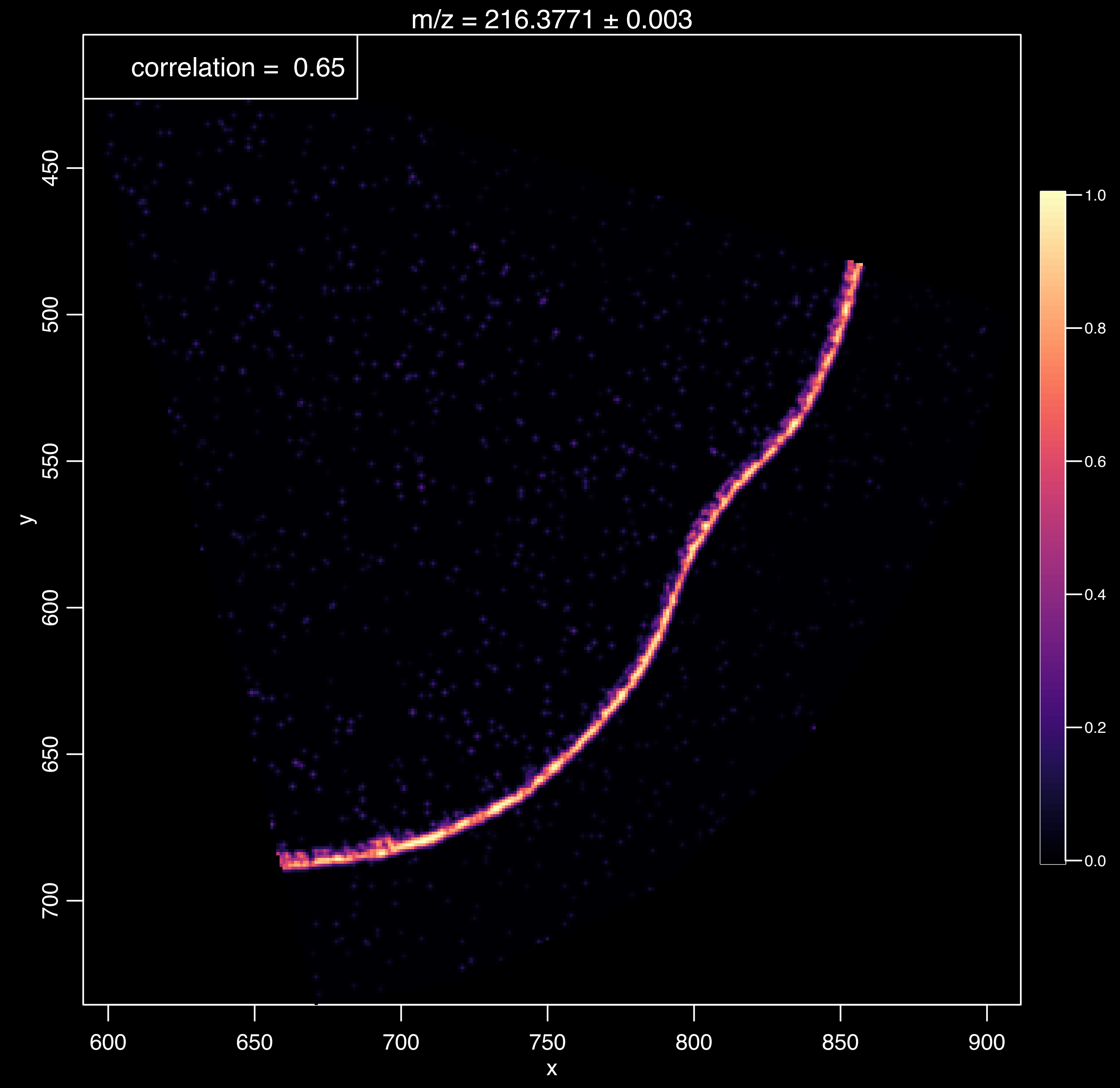

m/z = 216.3771 ± 0.003
correlation = 0.65
450
500
550
600
650
700
y
600
650
700
750
800
850
900
x
1.0
0.8
0.6
0.4
0.2
0.0

Figure S5

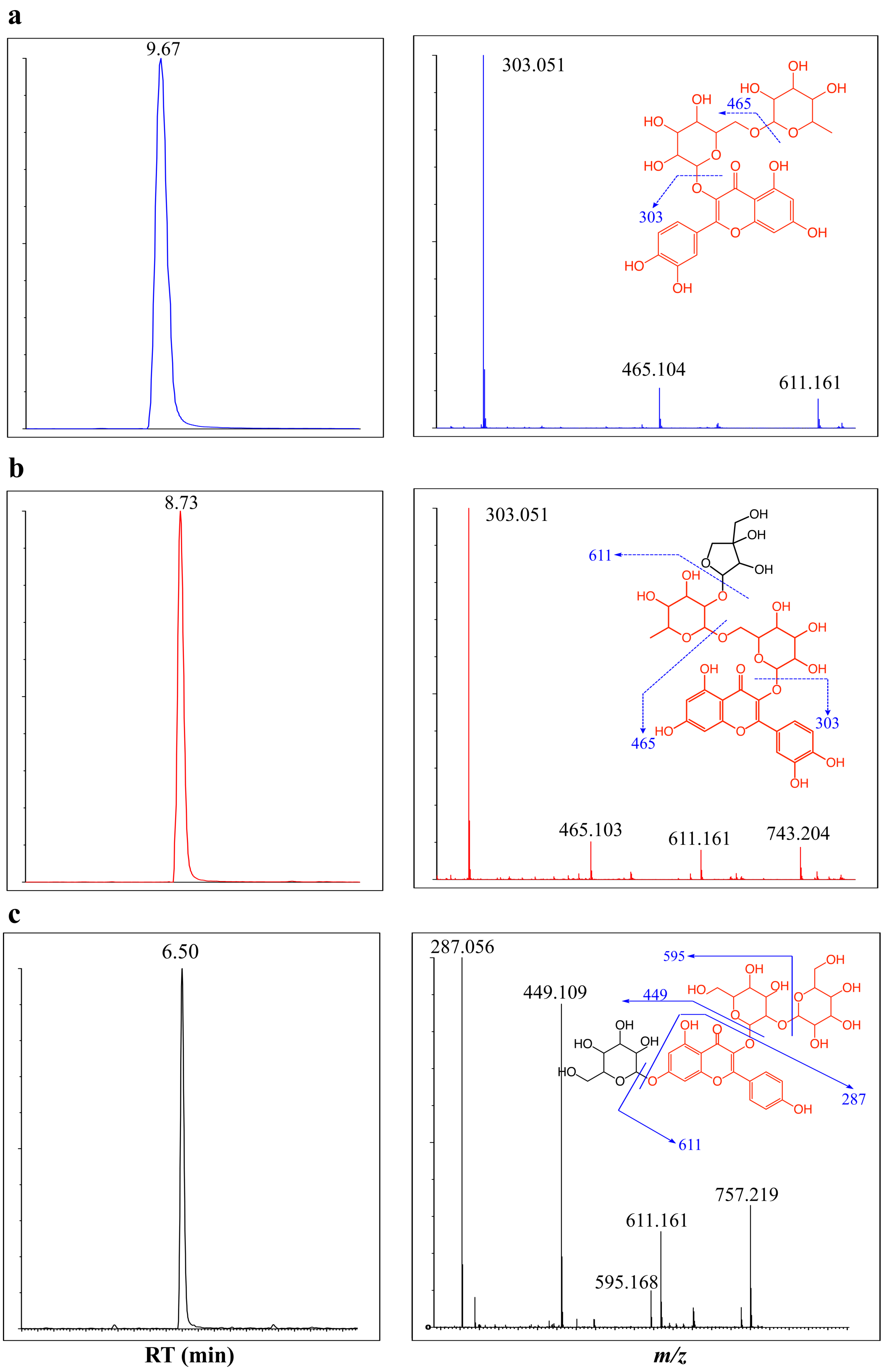

a
9.67
303.051
465
303
465.104
611.161
b
8.73
303.051
611
465
303
465.103
611.161
743.204
c
6.50
287.056
595
449
449.109
287
611
757.219
611.161
595.168
RT (min)
m/z

Figure S5 LC-MS/MS of rutin, rutin-pentoside, and kaempferol 3-rutinoside-7-glucoside. Left panel: LC chromatograms of rutin (a), rutin-pentoside (b), and kaempferol 3-rutinoside-7-glucoside (c), respectively. Right panel: MS/MS spectra and annotated fragments rutin (a), rutin-pentoside (b), and kaempferol 3-rutinoside-7-glucoside (c).

Figure S6 MS images of ions highly and moderately colocalized (PCC score >= 0.8) with petunidin peak *m/z* 317.066.

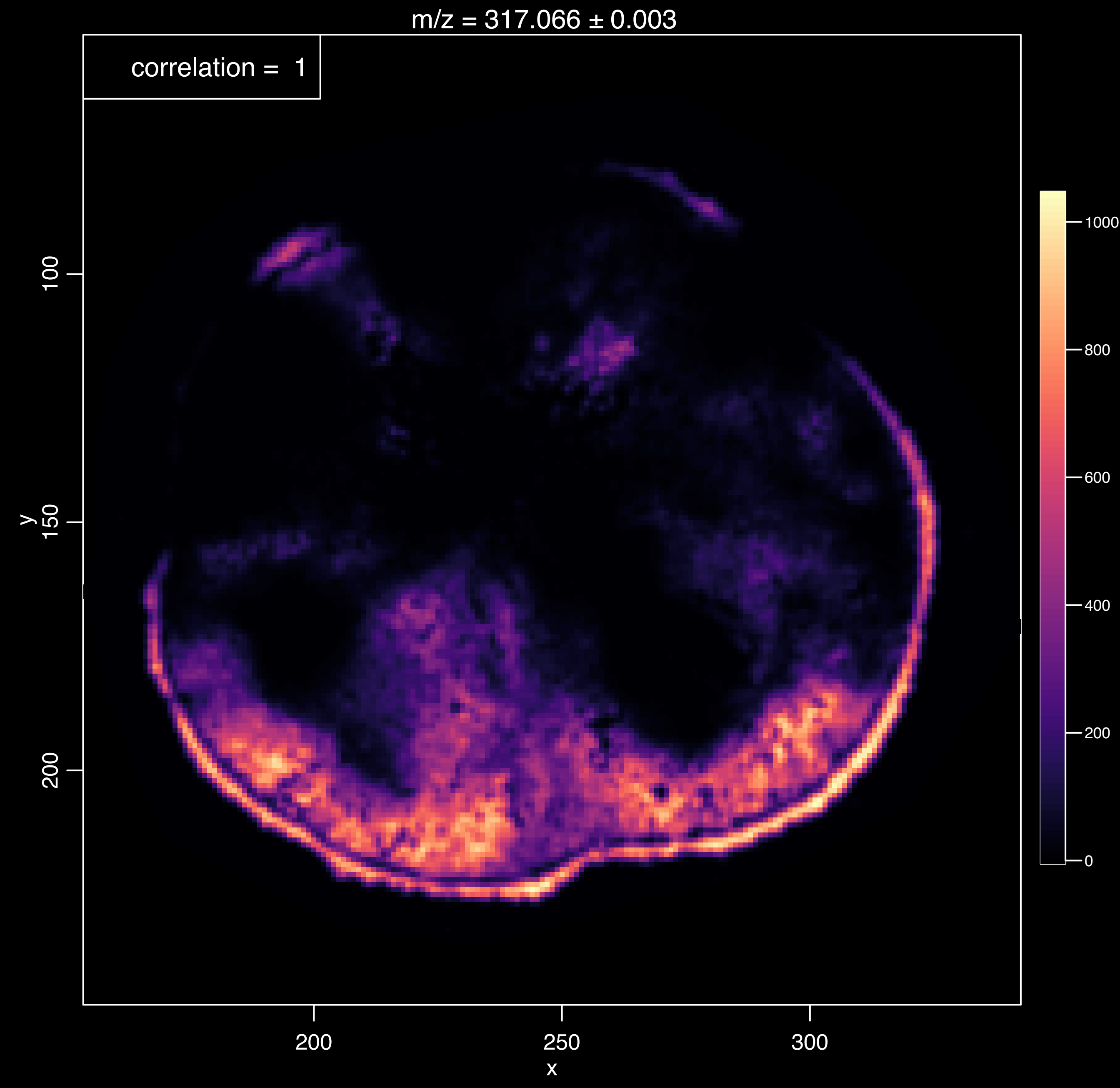

m/z = 317.066 ± 0.003
correlation = 1
100
150
200
y
200
250
300
x
1000
800
600
400
200
0

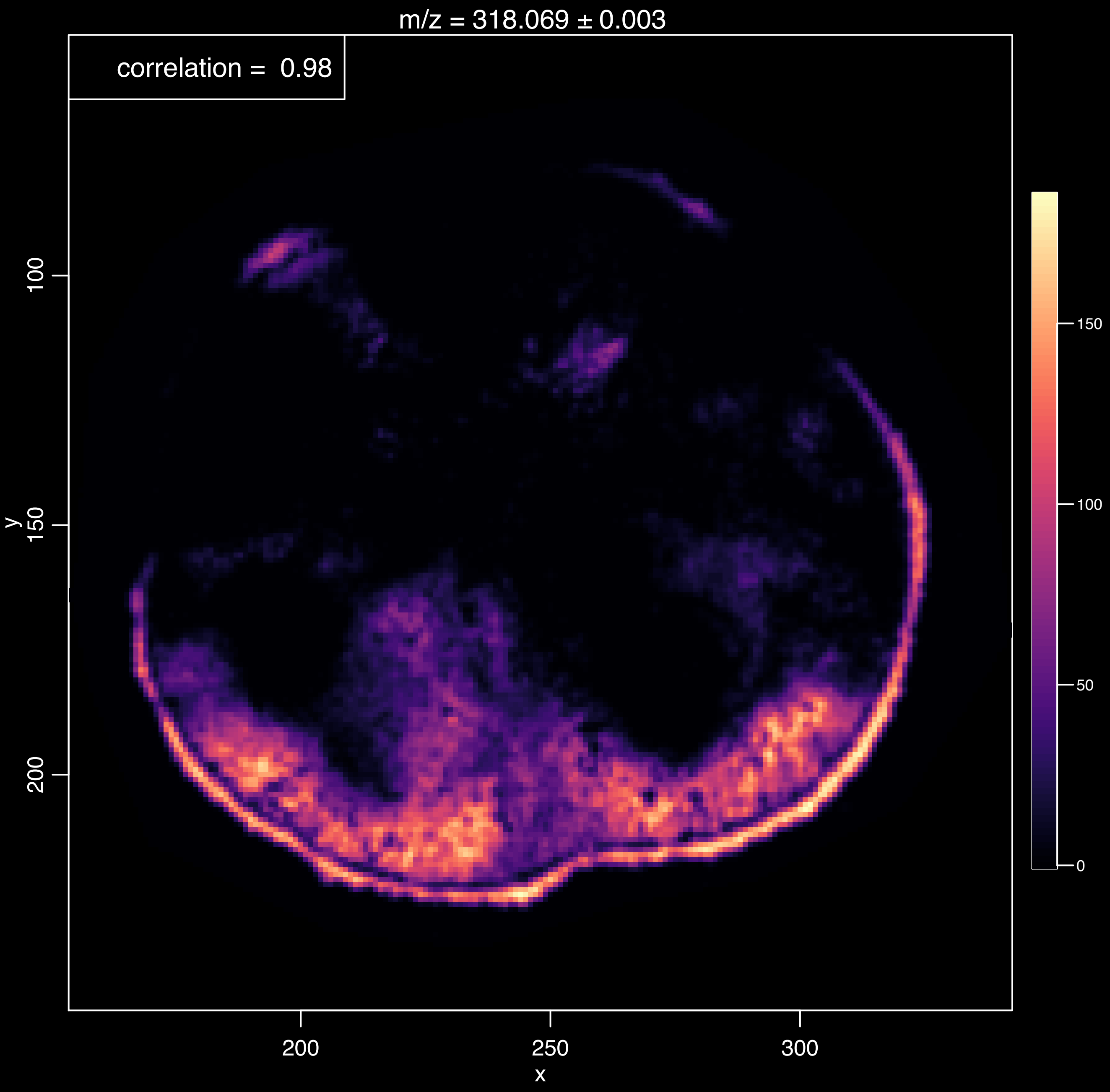

m/z = 318.069 ± 0.003
correlation = 0.98
100
150
200
y
200
250
300
x
150
100
50
0

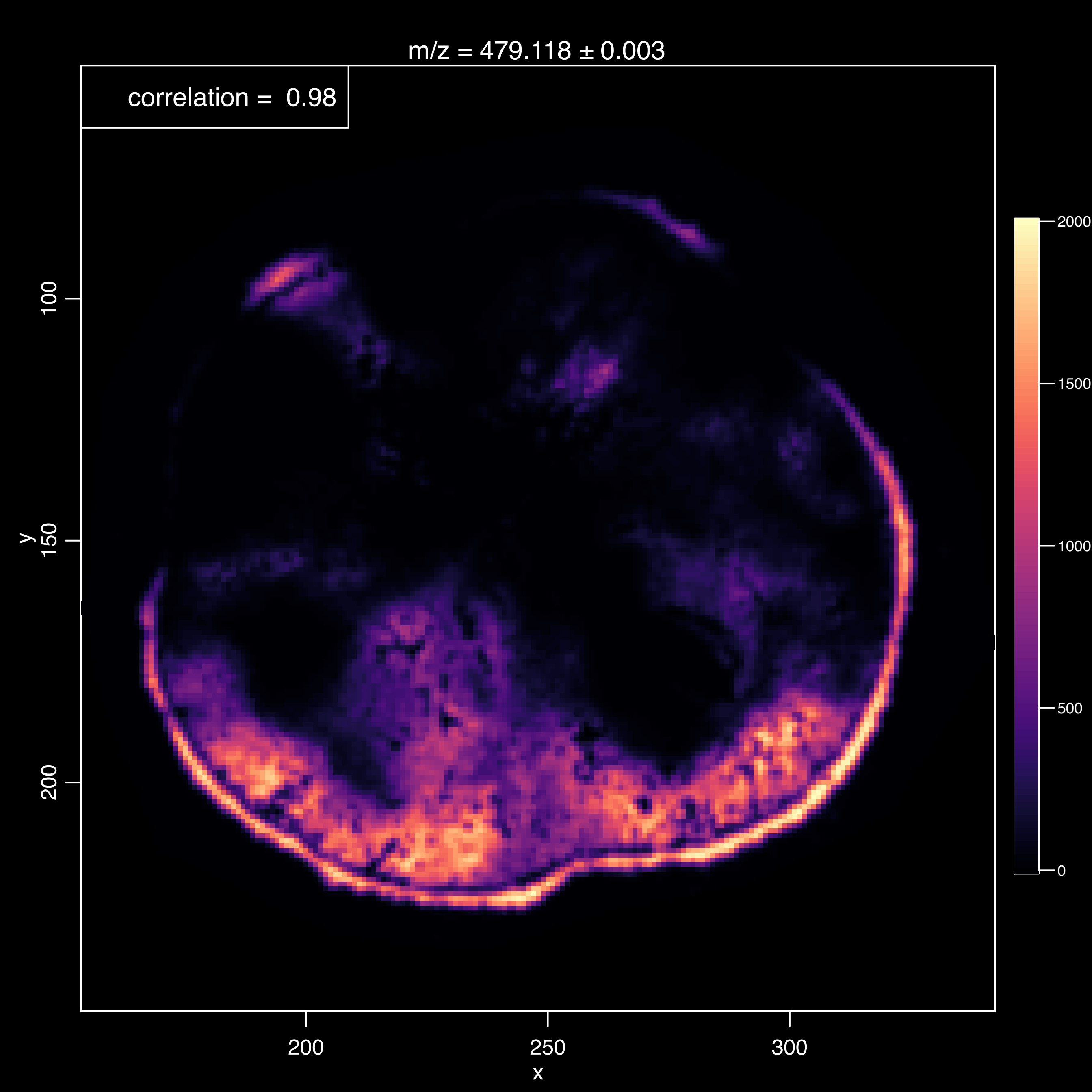

m/z = 479.118 ± 0.003
correlation = 0.98
y
100
150
200
x
200
250
300
2000
1500
1000
500
0

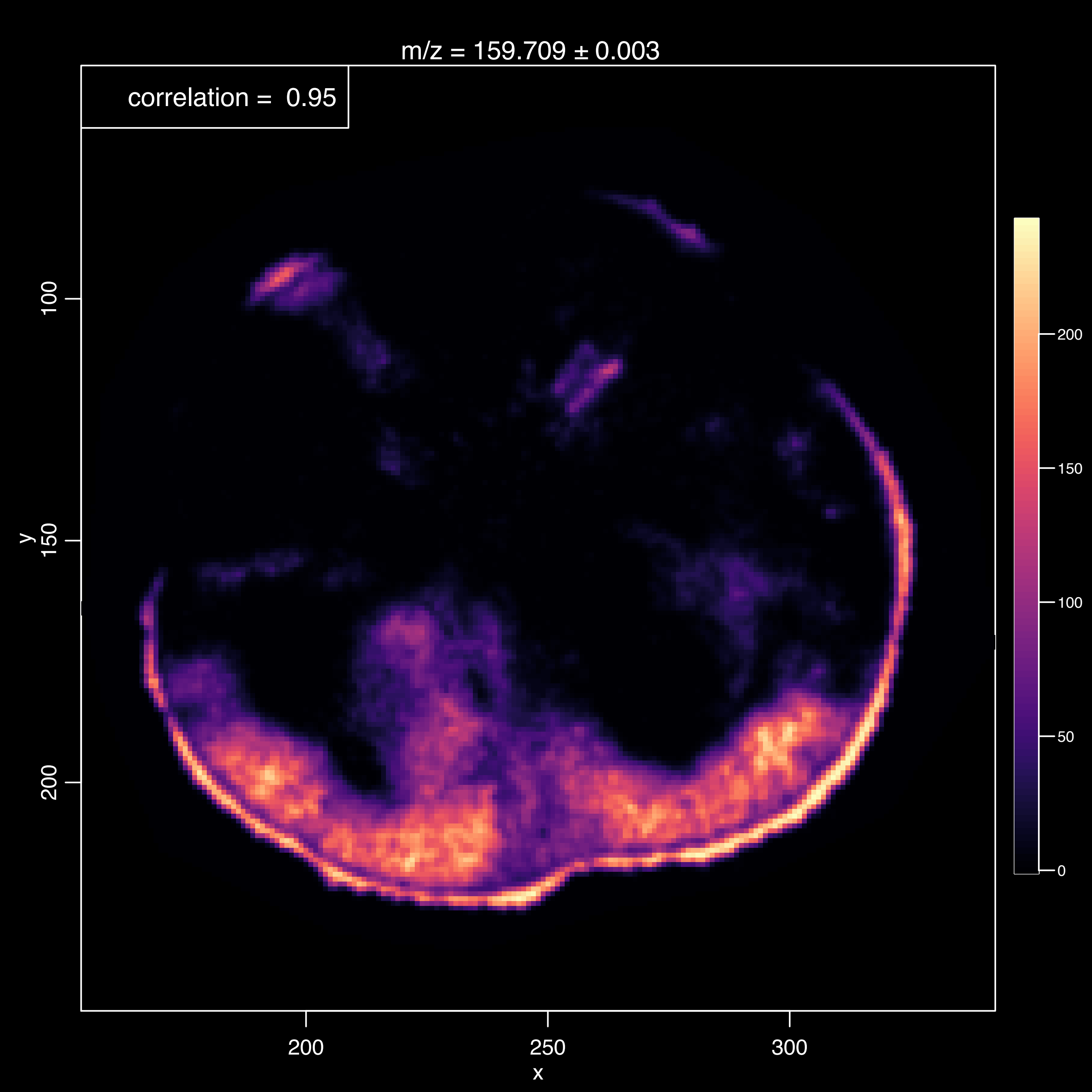

m/z = 159.709 ± 0.003
correlation = 0.95
100
150
200
y
200
250
300
x
0
50
100
150
200

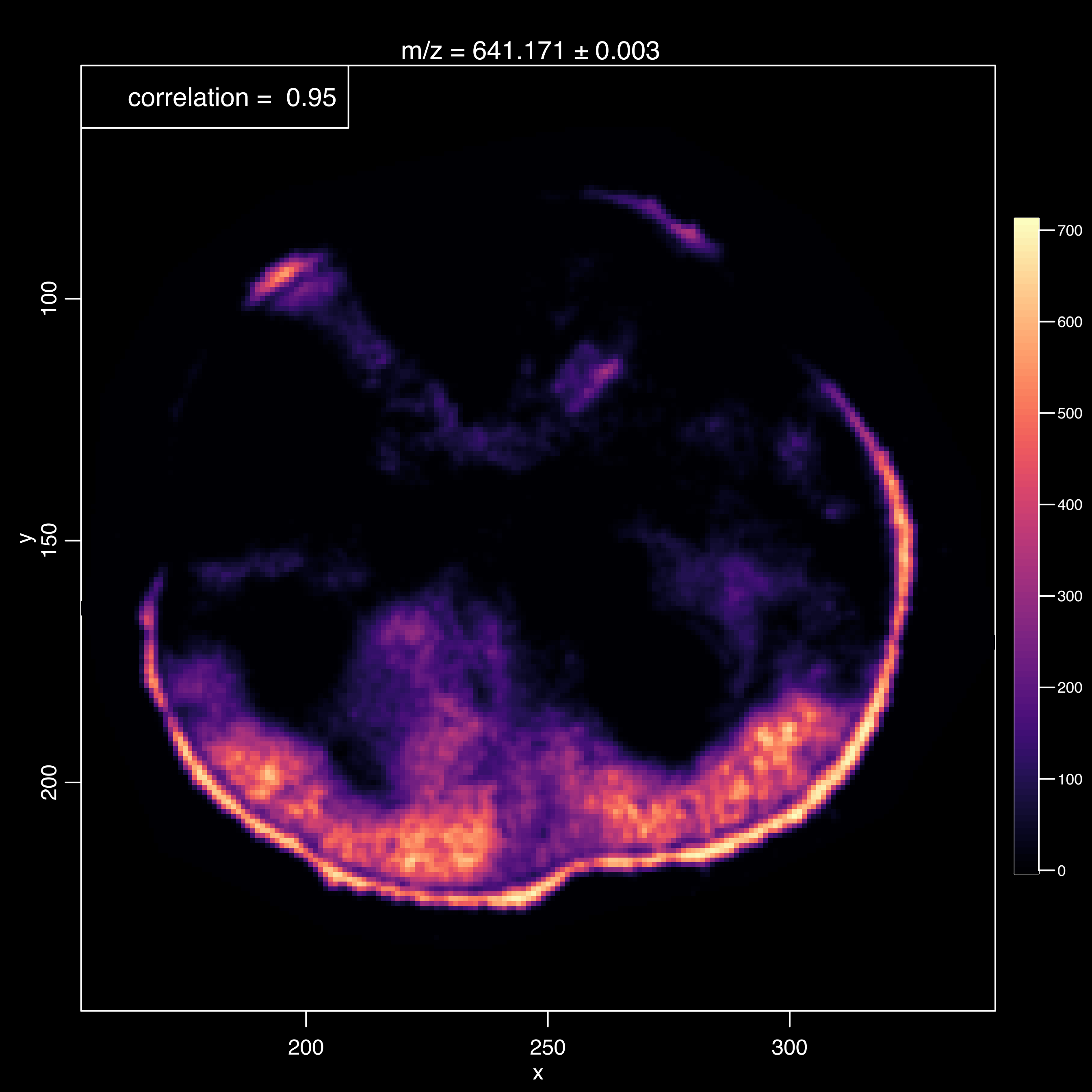
m/z = 641.171 ± 0.003
correlation = 0.95
y
100
150
200
x
200
250
300
700
600
500
400
300
200
100
0

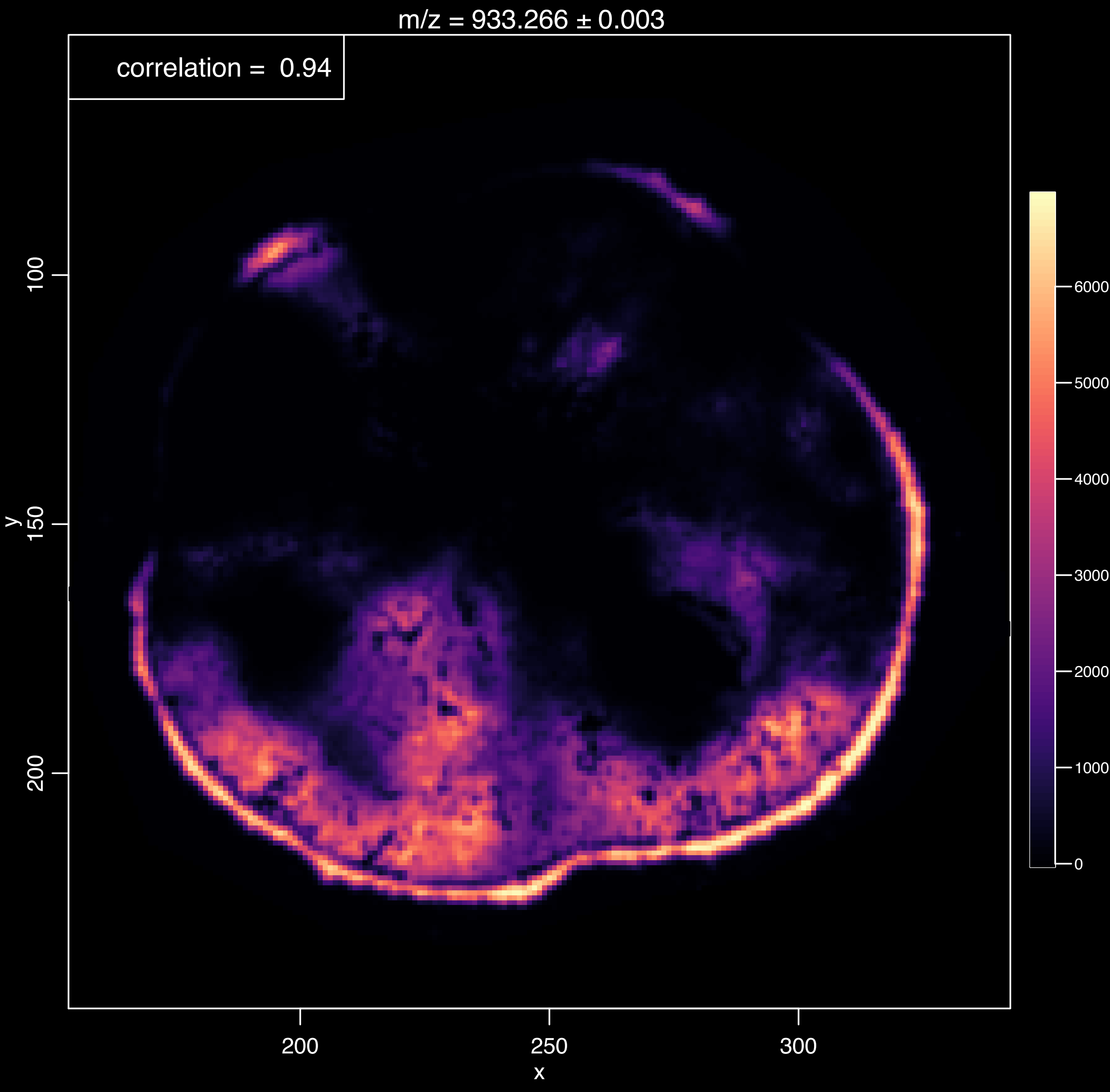

m/z = 933.266 ± 0.003
correlation = 0.94
100
150
200
y
200
250
300
x
6000
5000
4000
3000
2000
1000
0

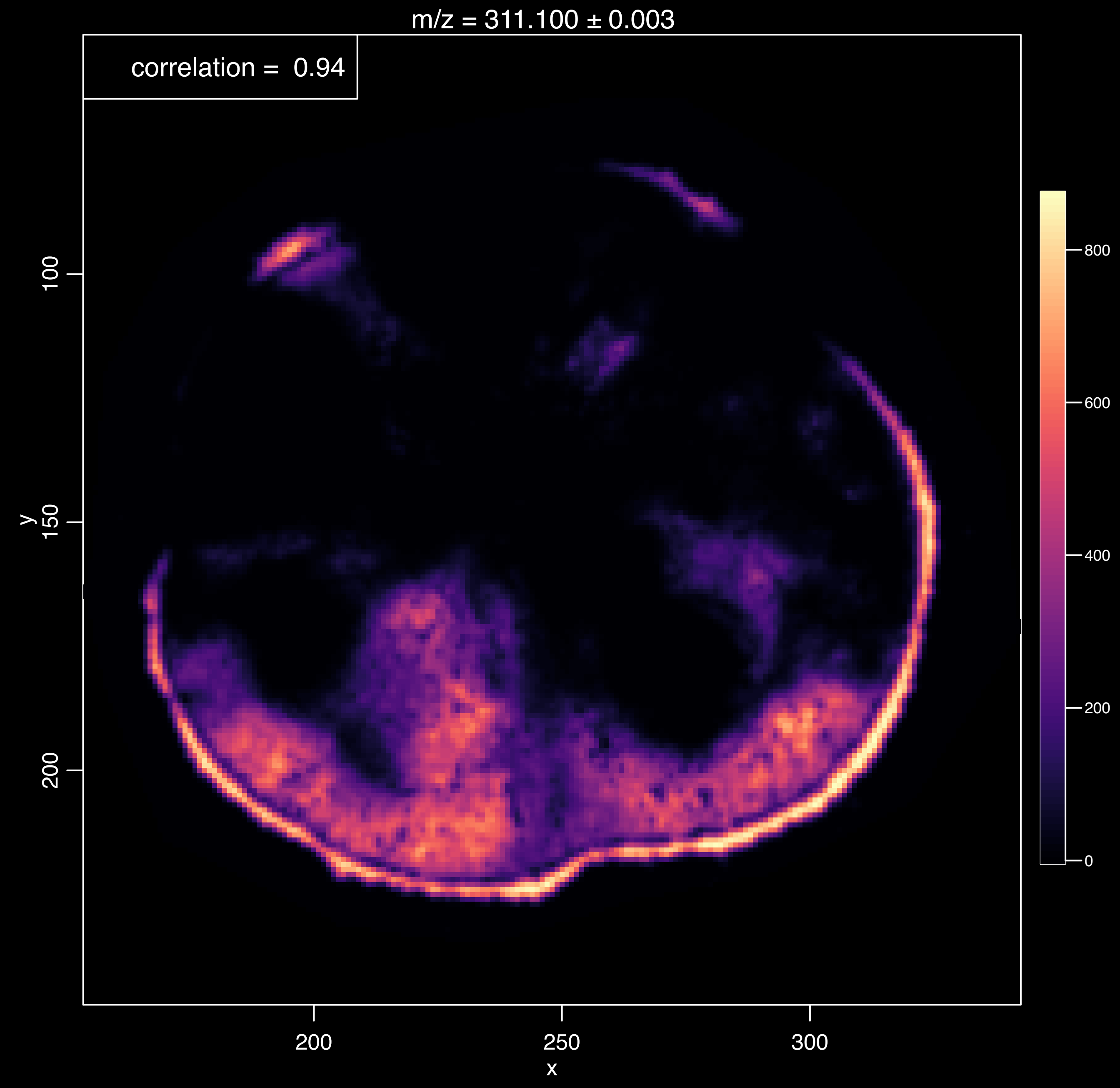
m/z = 311.100 ± 0.003
correlation = 0.94
100
150
200
y
200
250
300
x
800
600
400
200
0

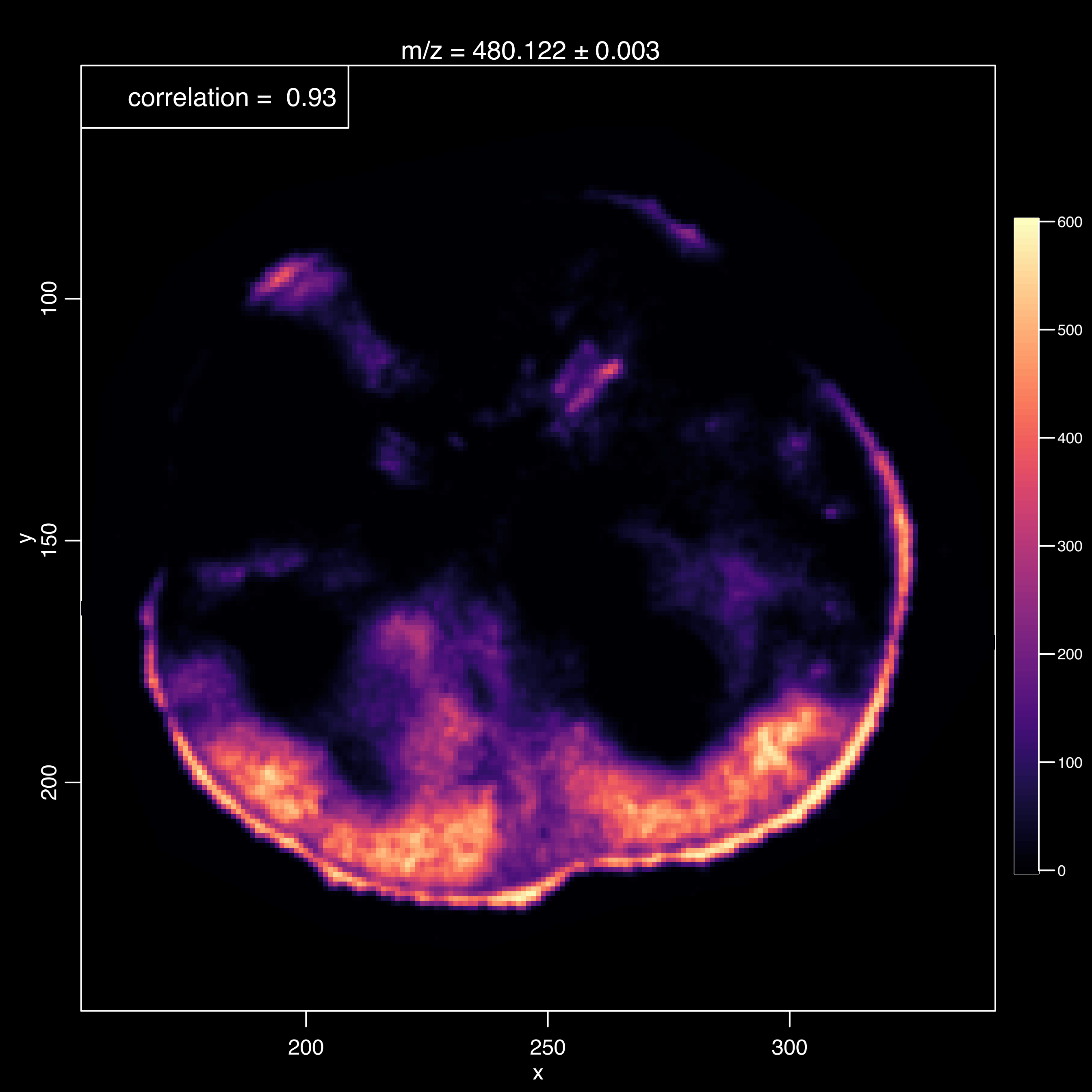
m/z = 480.122 ± 0.003
correlation = 0.93
y
100
150
200
x
200
250
300
600
500
400
300
200
100
0

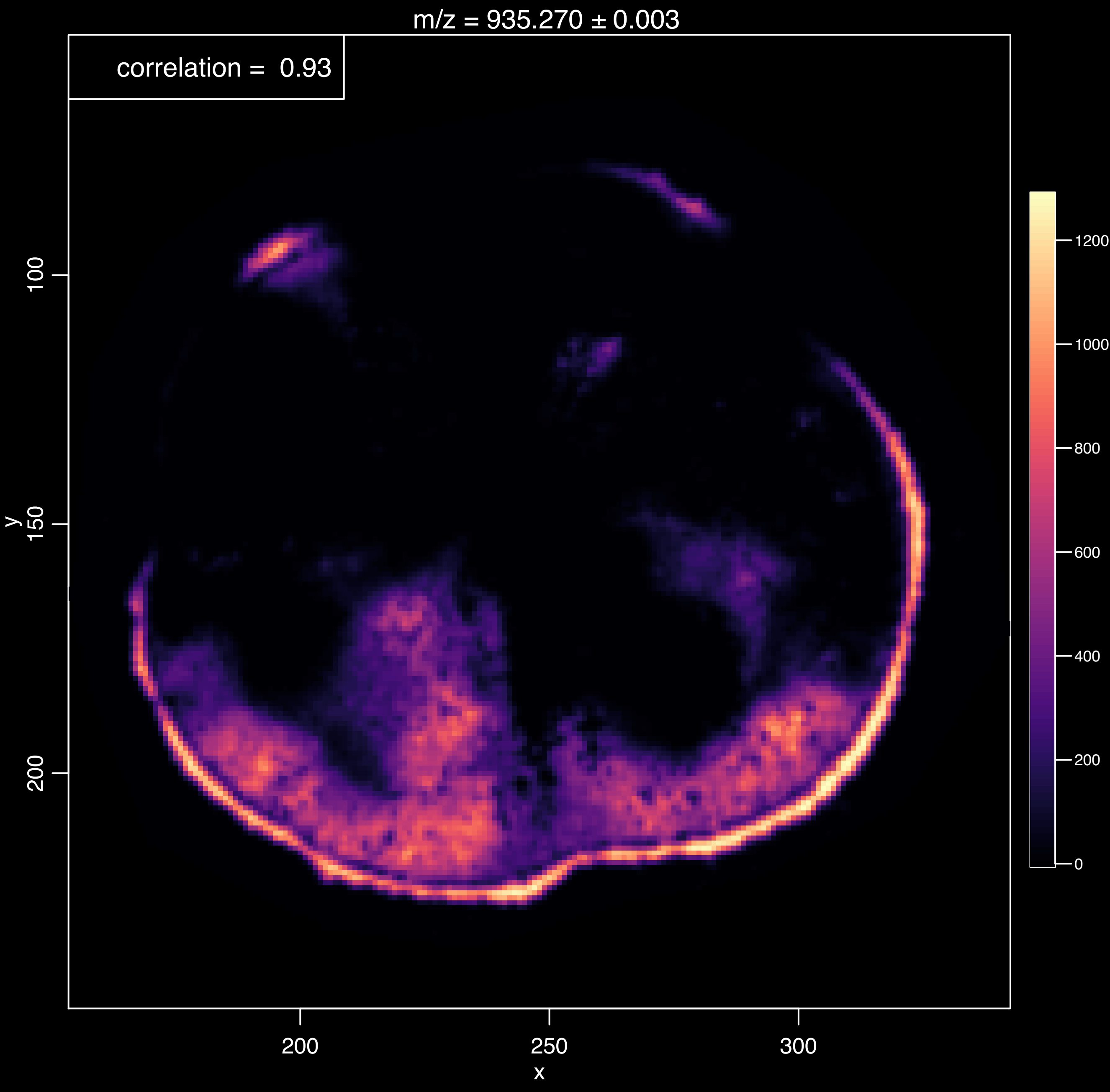

m/z = 935.270 ± 0.003
correlation = 0.93
y
100
150
200
x
200
250
300
0
200
400
600
800
1000
1200

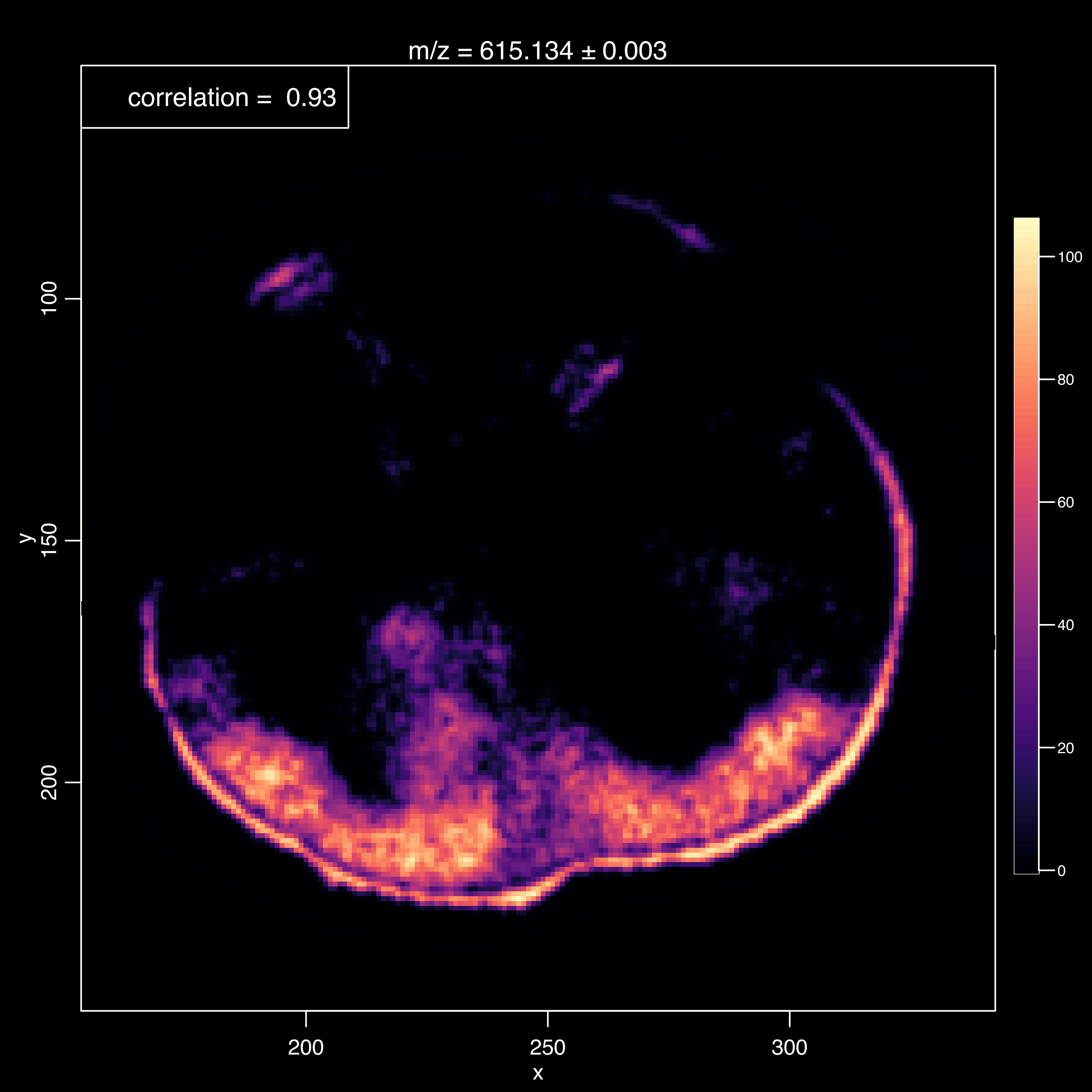
m/z = 615.134 ± 0.003
correlation = 0.93
y
100
150
200
x
200
250
300
100
80
60
40
20
0

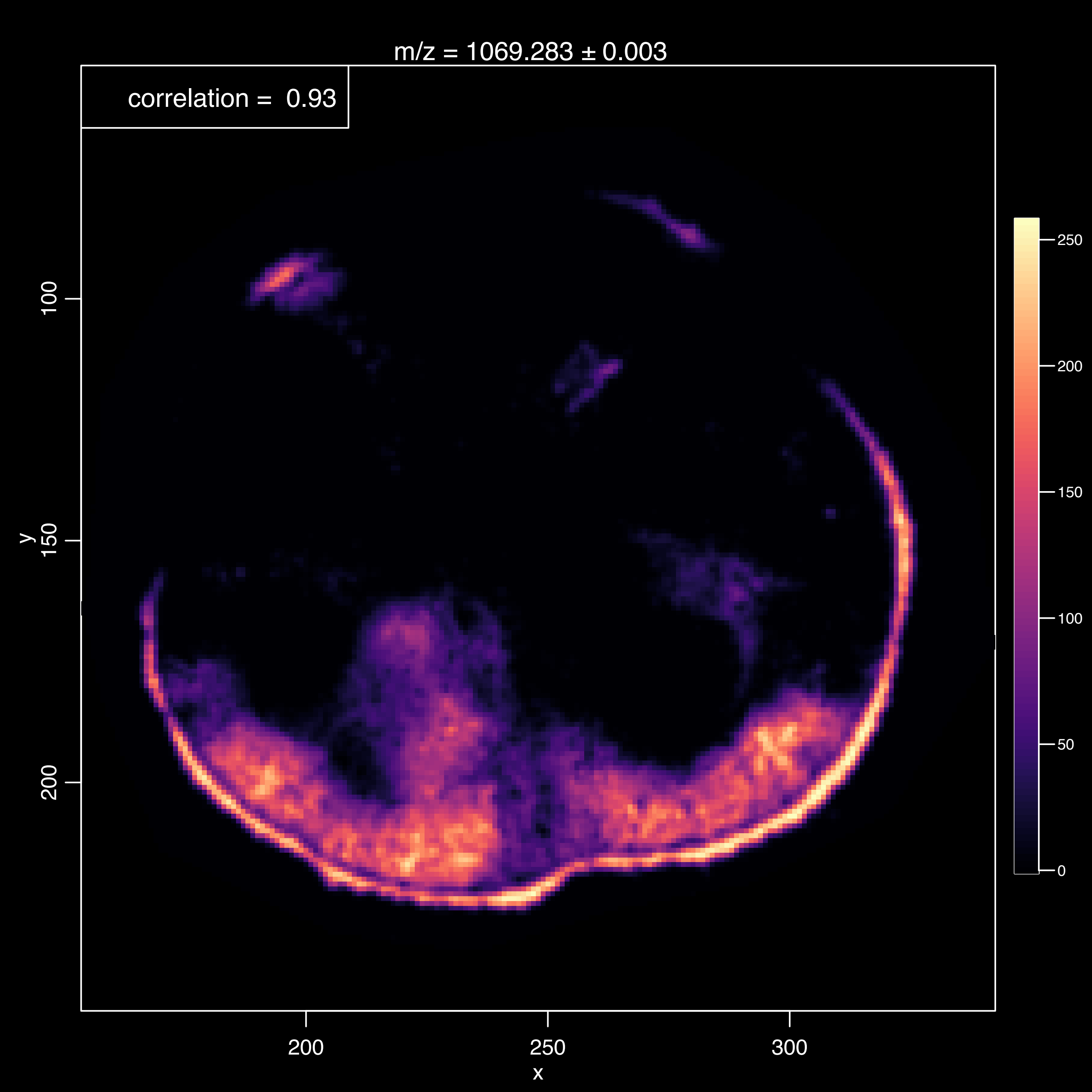

m/z = 1069.283 ± 0.003
correlation = 0.93
y
100
150
200
x
200
250
300
250
200
150
100
50
0

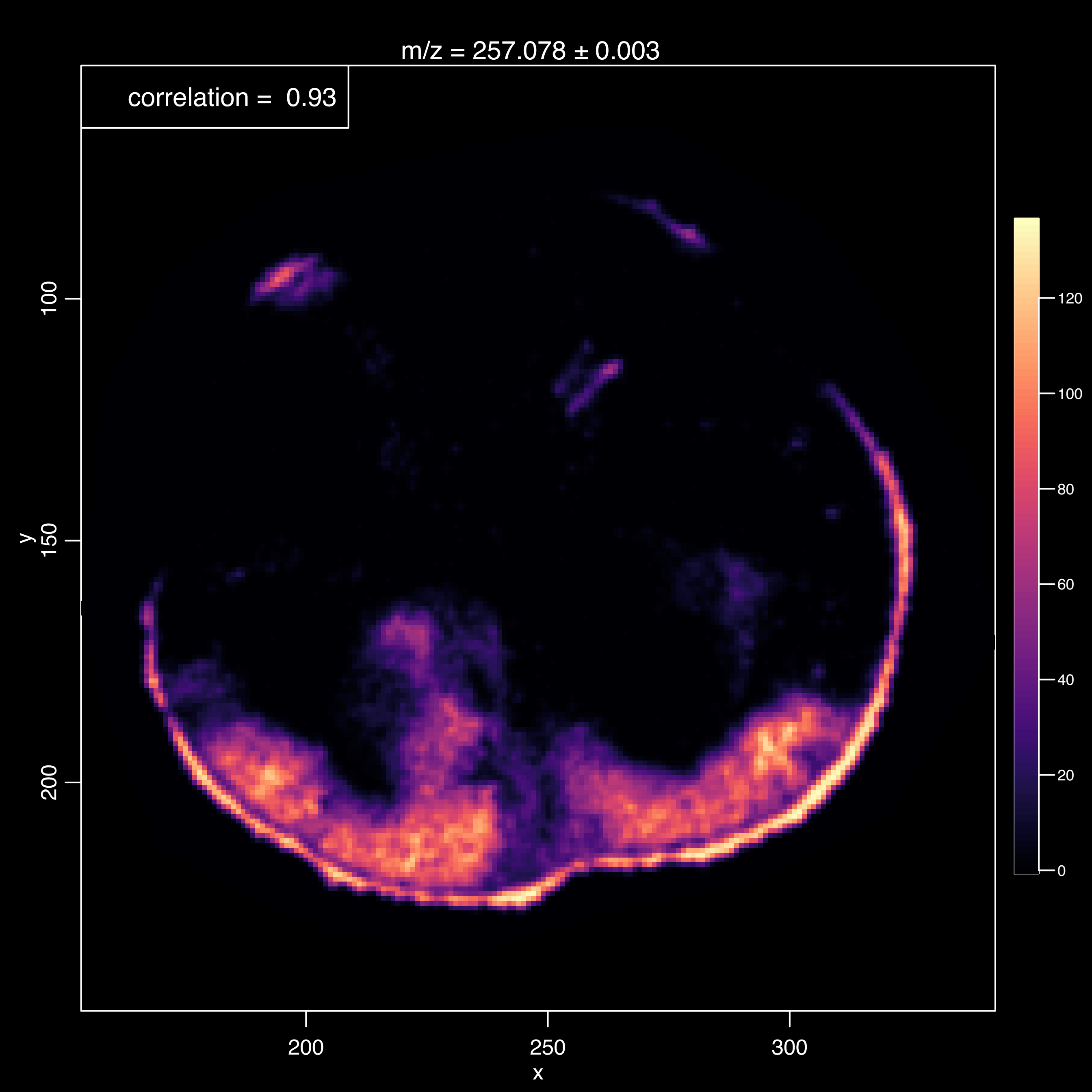

m/z = 257.078 ± 0.003
correlation = 0.93
y
100
150
200
x
200
250
300
120
100
80
60
40
20
0

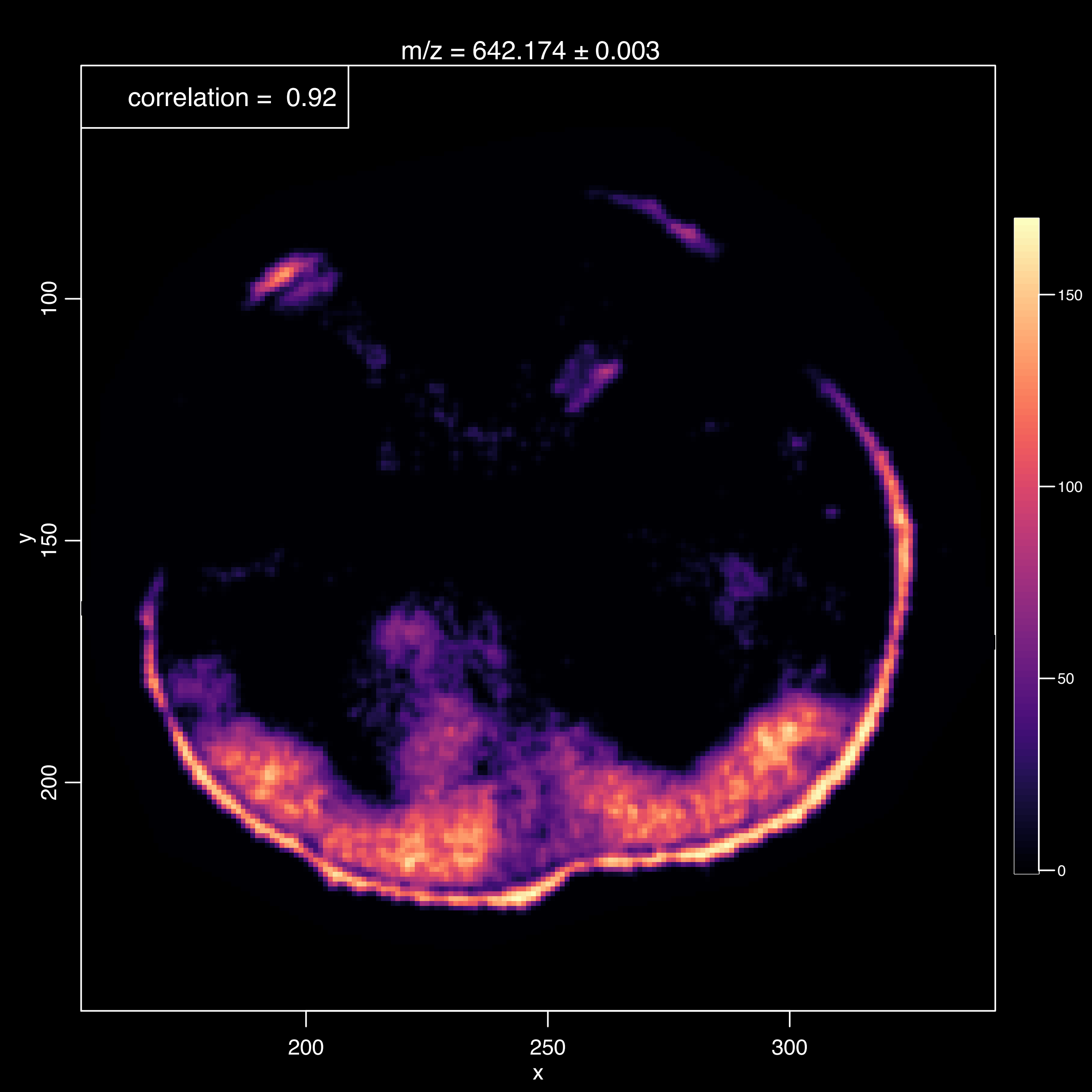
m/z = 642.174 ± 0.003
correlation = 0.92
100
150
200
y
200
250
300
x
150
100
50
0

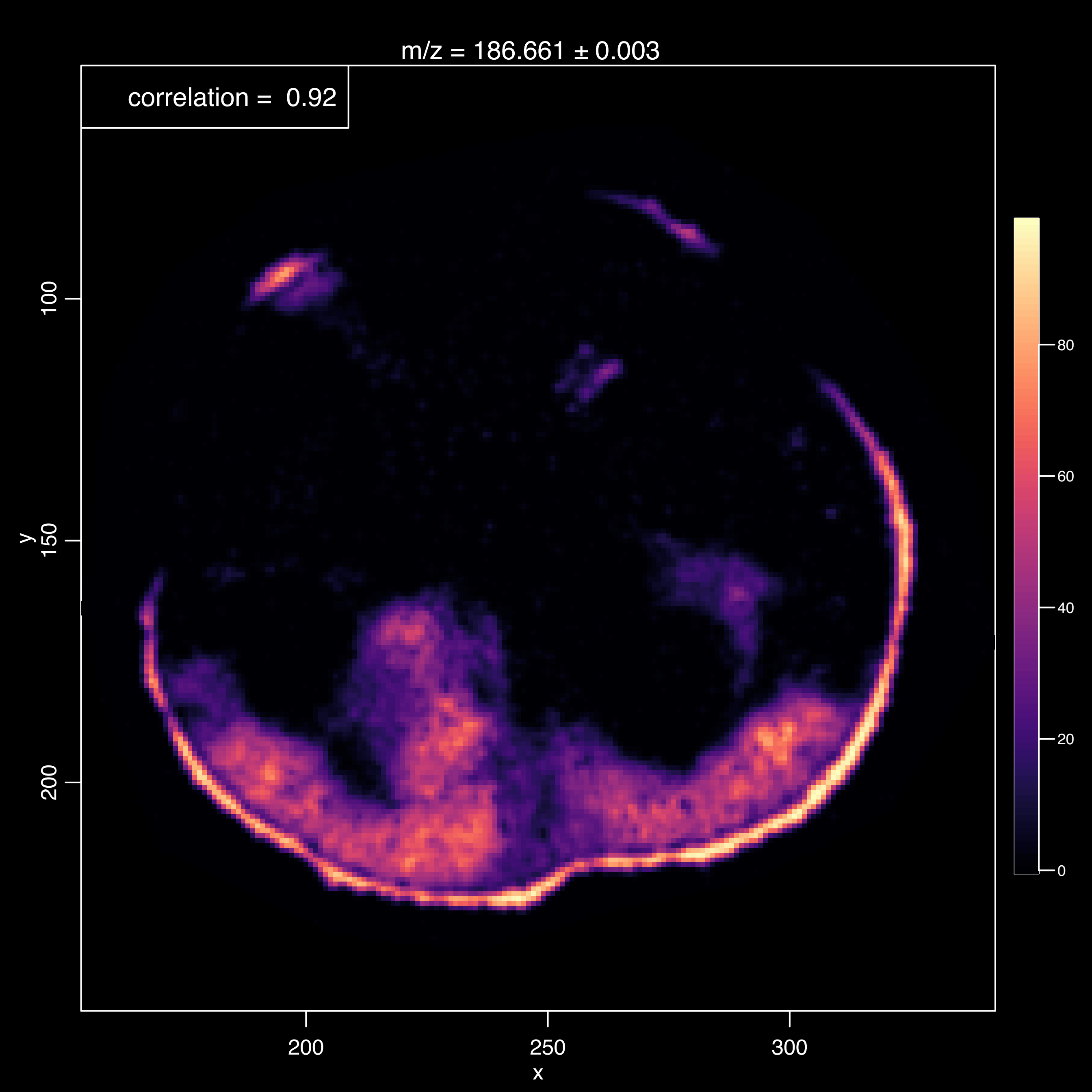
m/z = 186.661 ± 0.003
correlation = 0.92
100
150
200
y
200
250
300
x
80
60
40
20
0

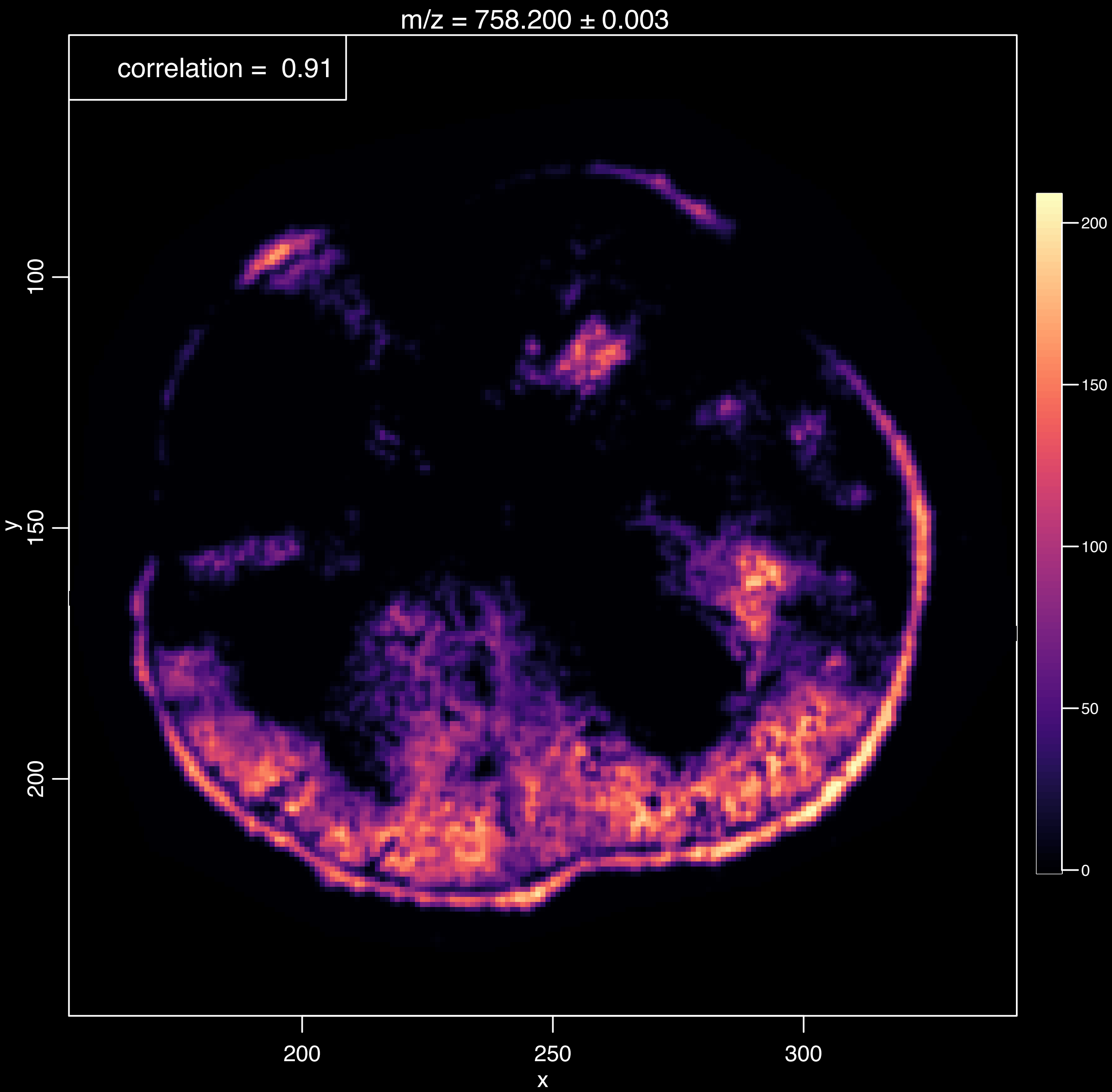
m/z = 758.200 ± 0.003
correlation = 0.91
100
150
200
y
200
250
300
x
0
50
100
150
200

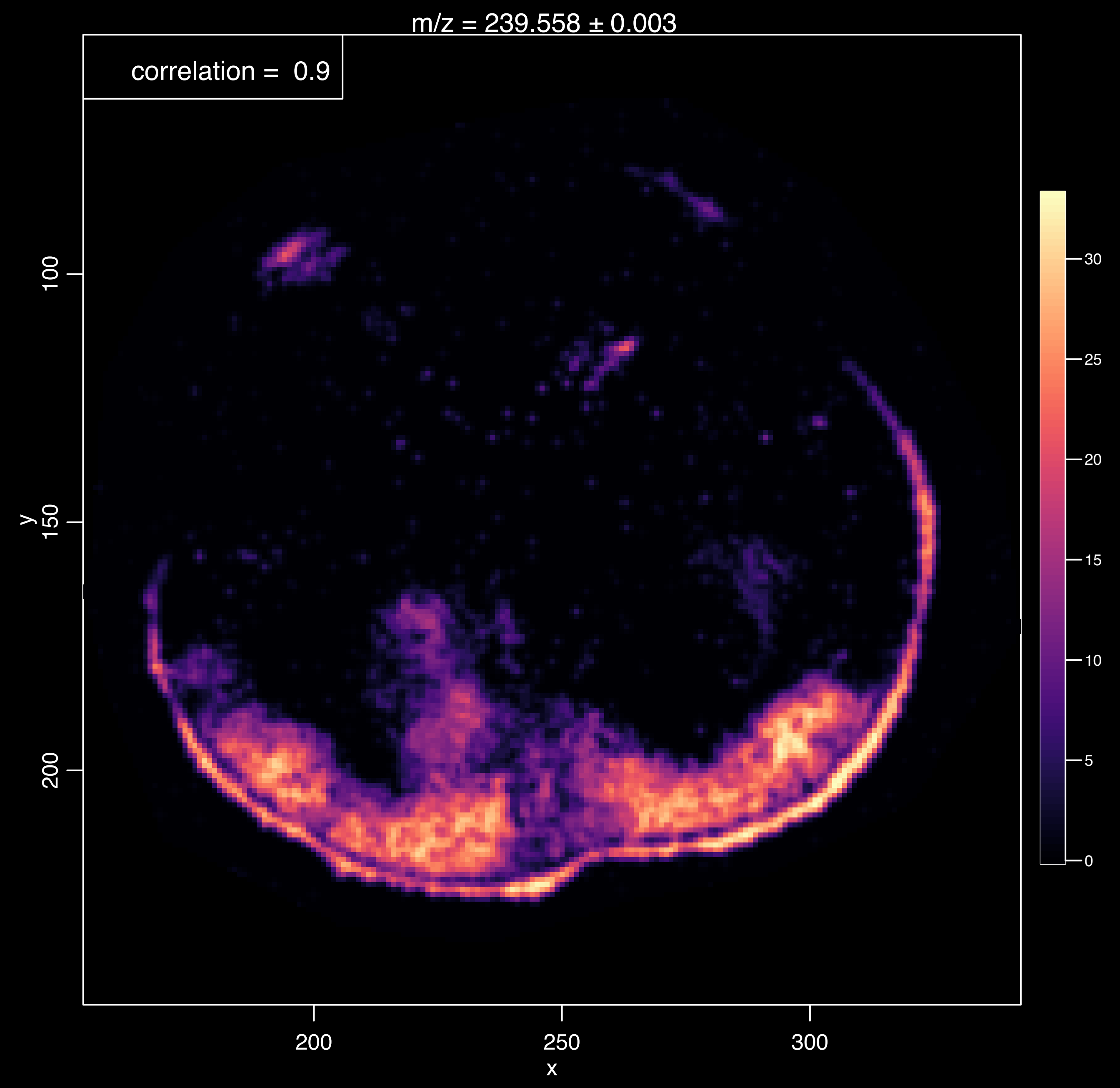

m/z = 239.558 ± 0.003
correlation = 0.9
y
100
150
200
x
200
250
300
0
5
10
15
20
25
30

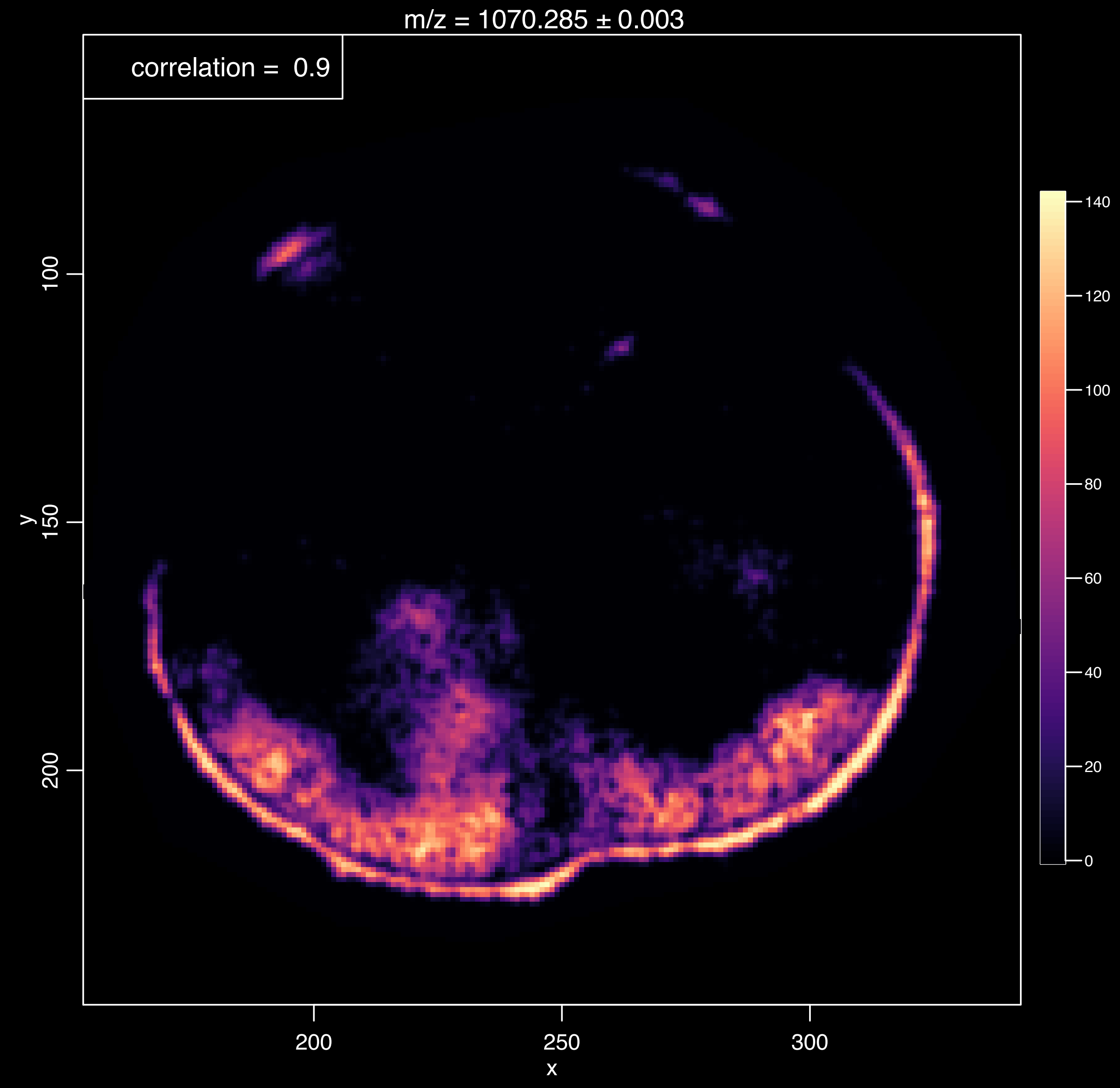

m/z = 1070.285 ± 0.003
correlation = 0.9
100
150
200
y
200
250
300
x
140
120
100
80
60
40
20
0

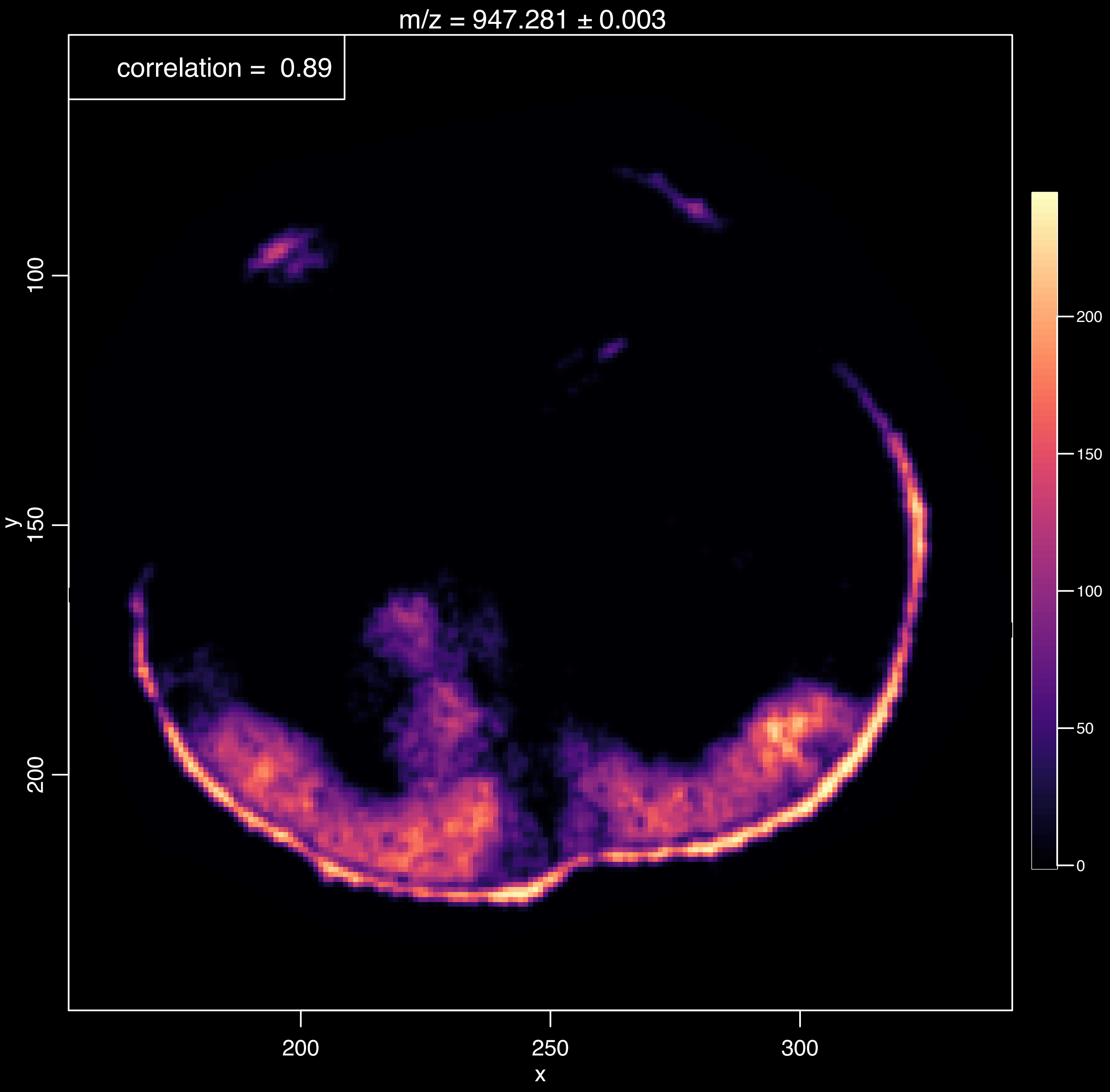
m/z = 947.281 ± 0.003
correlation = 0.89
y
100
150
200
x
200
250
300
0
50
100
150
200

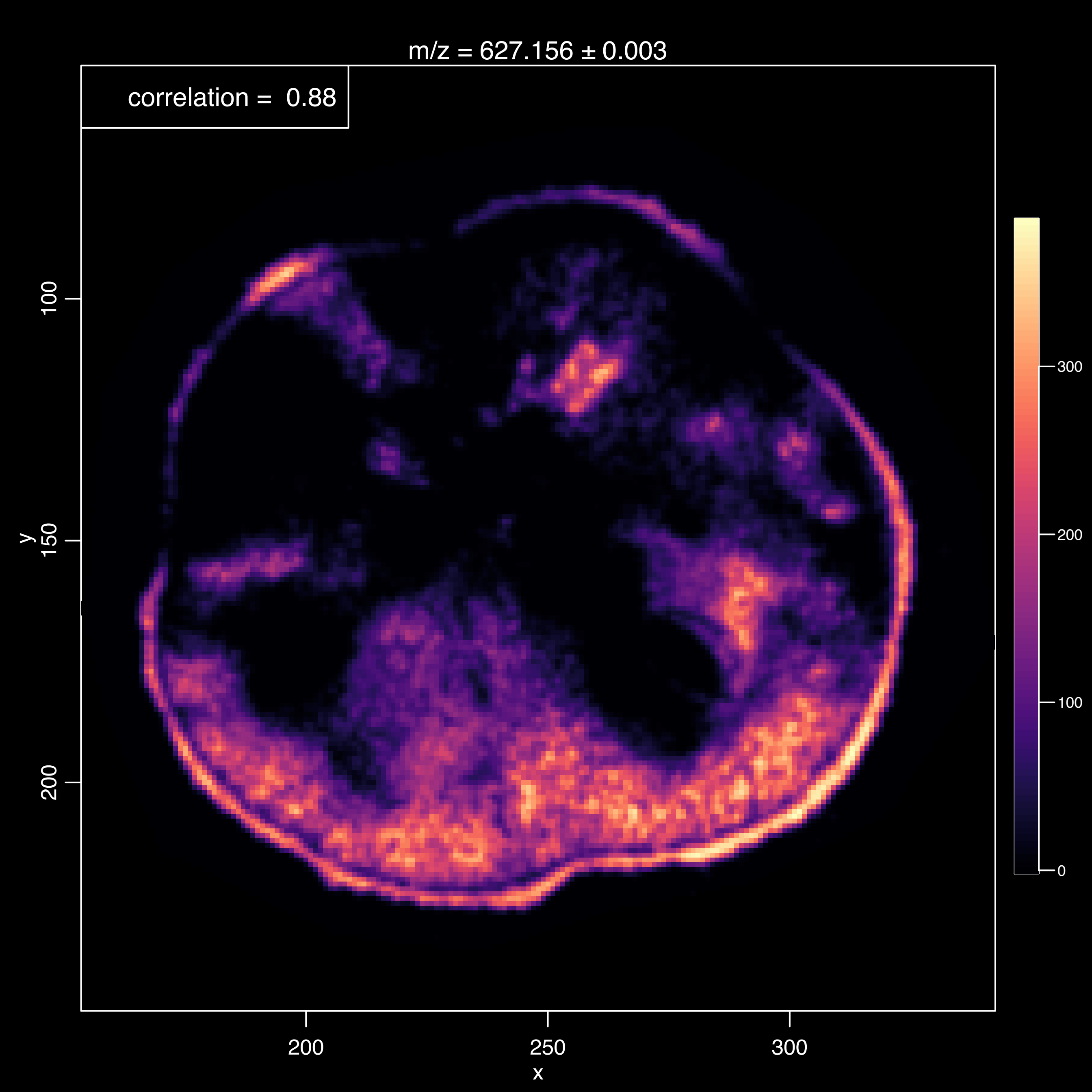
m/z = 627.156 ± 0.003
correlation = 0.88
100
150
200
y
200
250
300
x
300
200
100
0

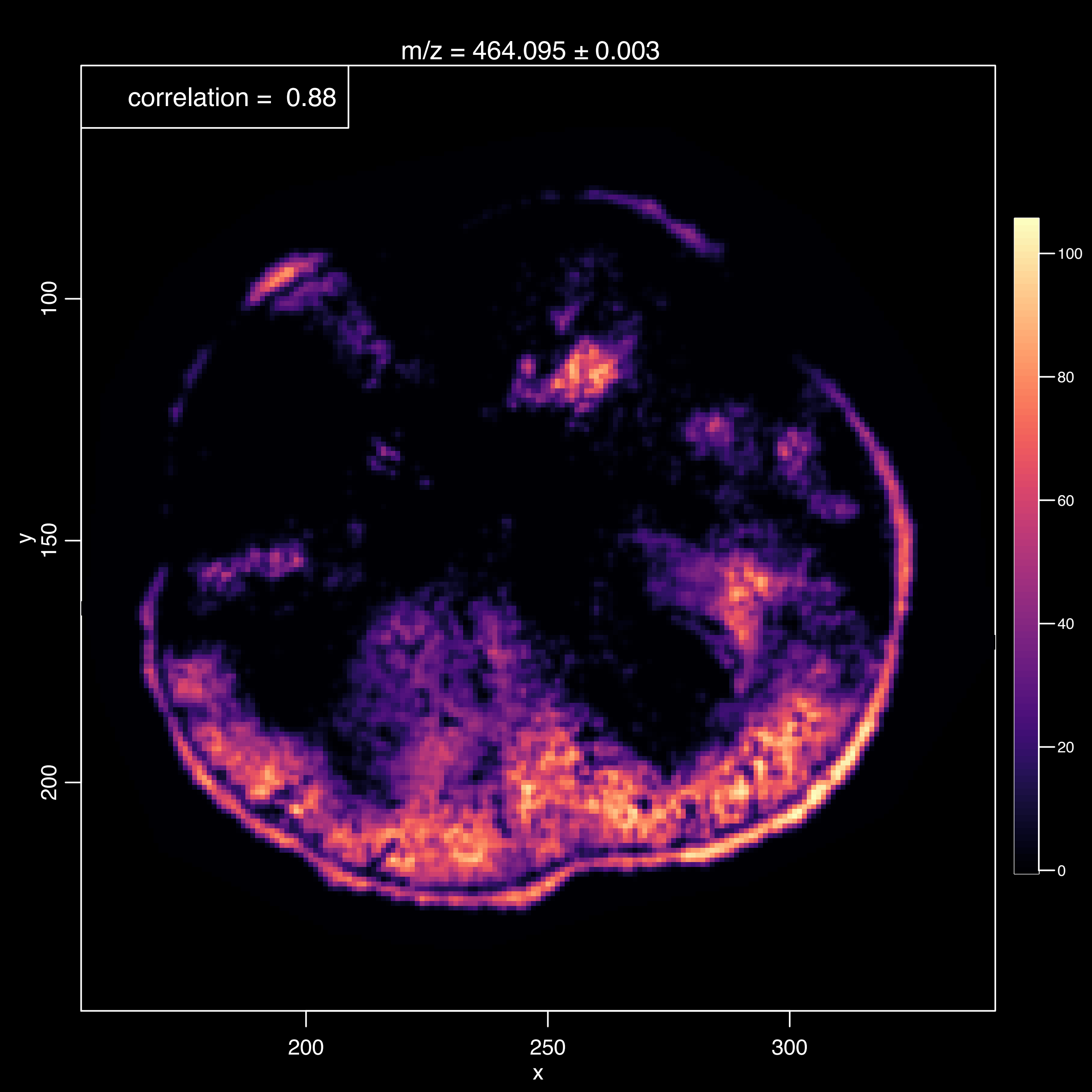

m/z = 464.095 ± 0.003
correlation = 0.88
y
100
150
200
x
200
250
300
0
20
40
60
80
100

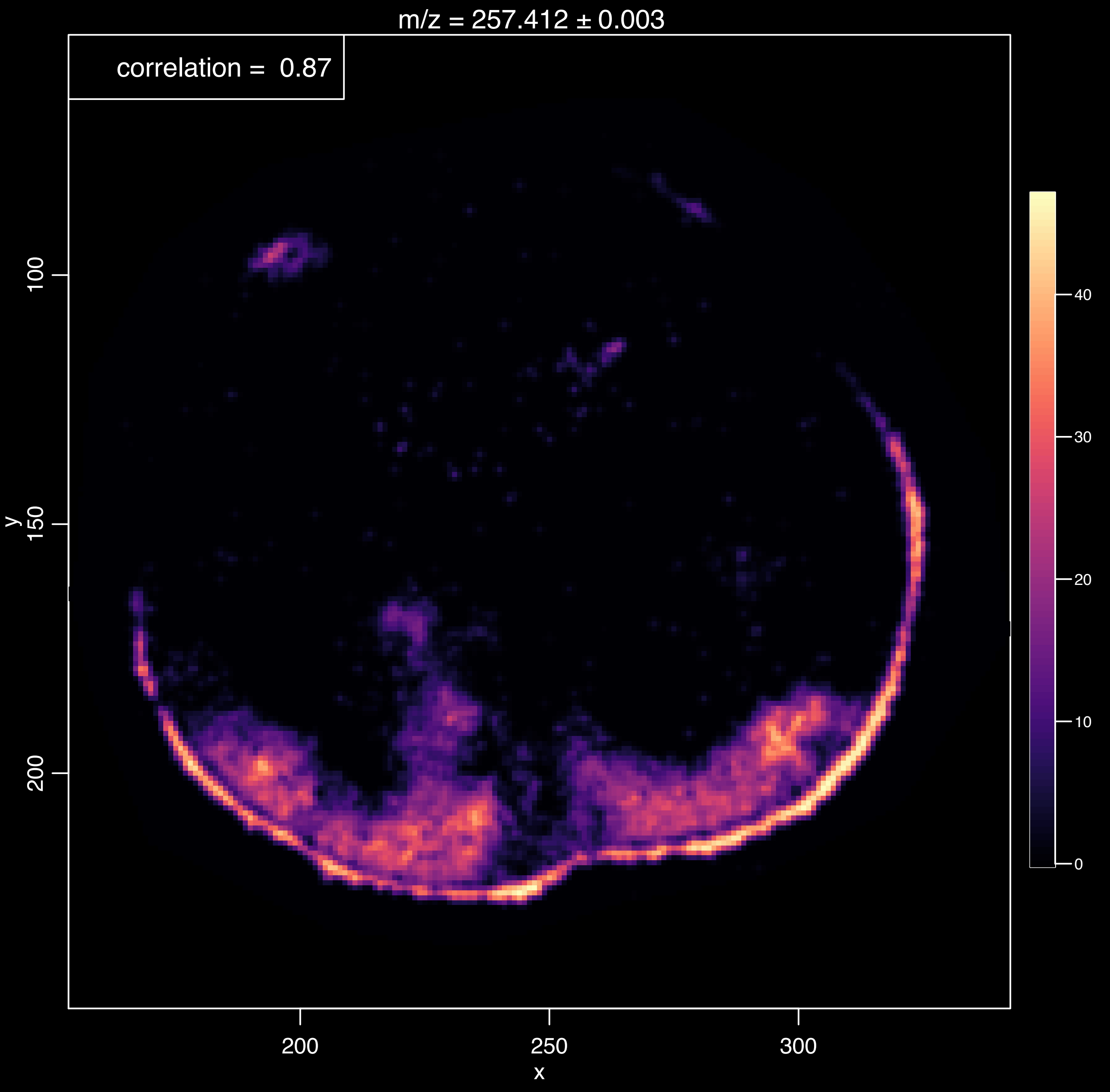
m/z = 257.412 ± 0.003
correlation = 0.87
y
100
150
200
x
200
250
300
0
10
20
30
40

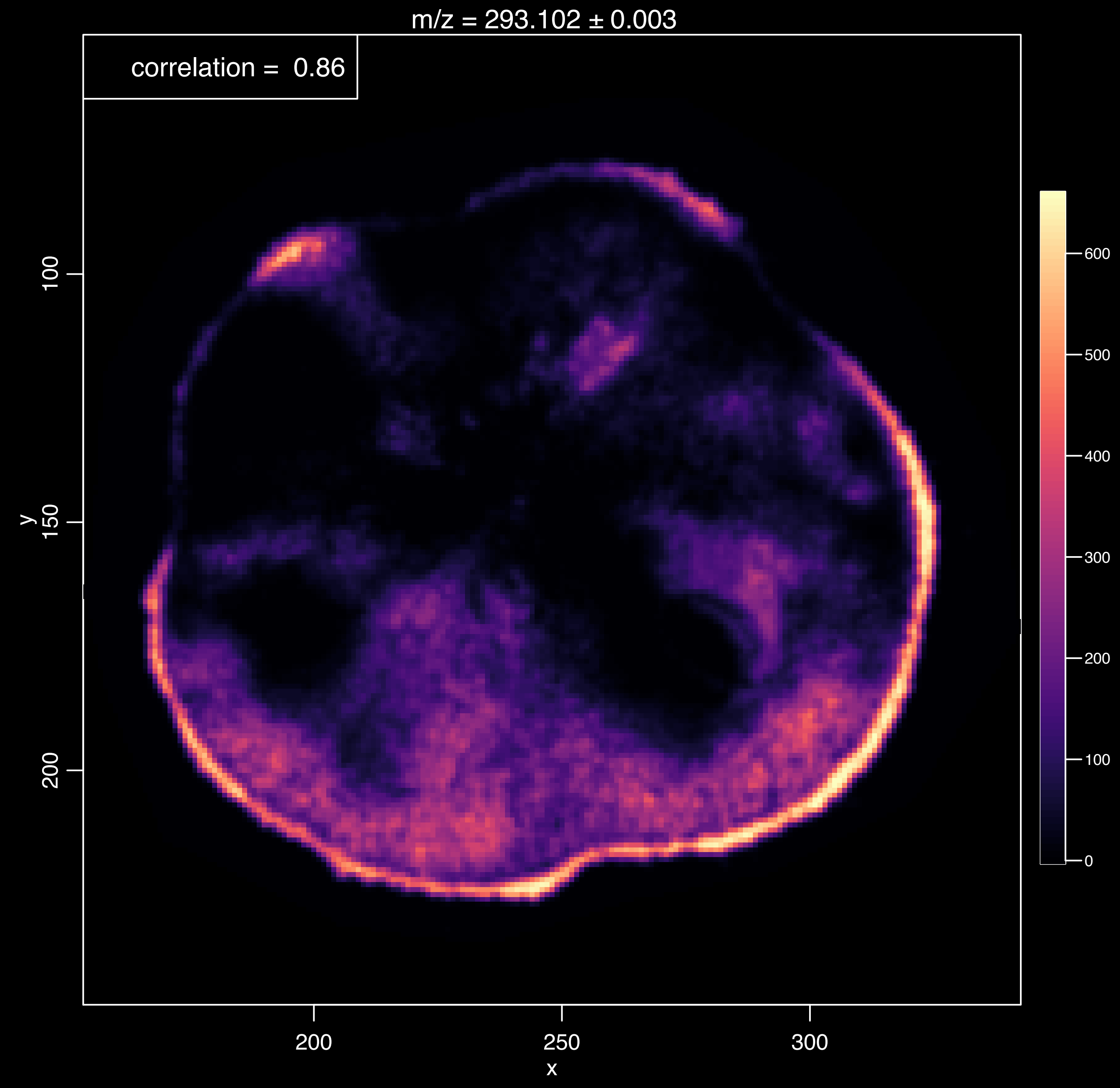

m/z = 293.102 ± 0.003
correlation = 0.86
100
150
200
y
200
250
300
x
600
500
400
300
200
100
0

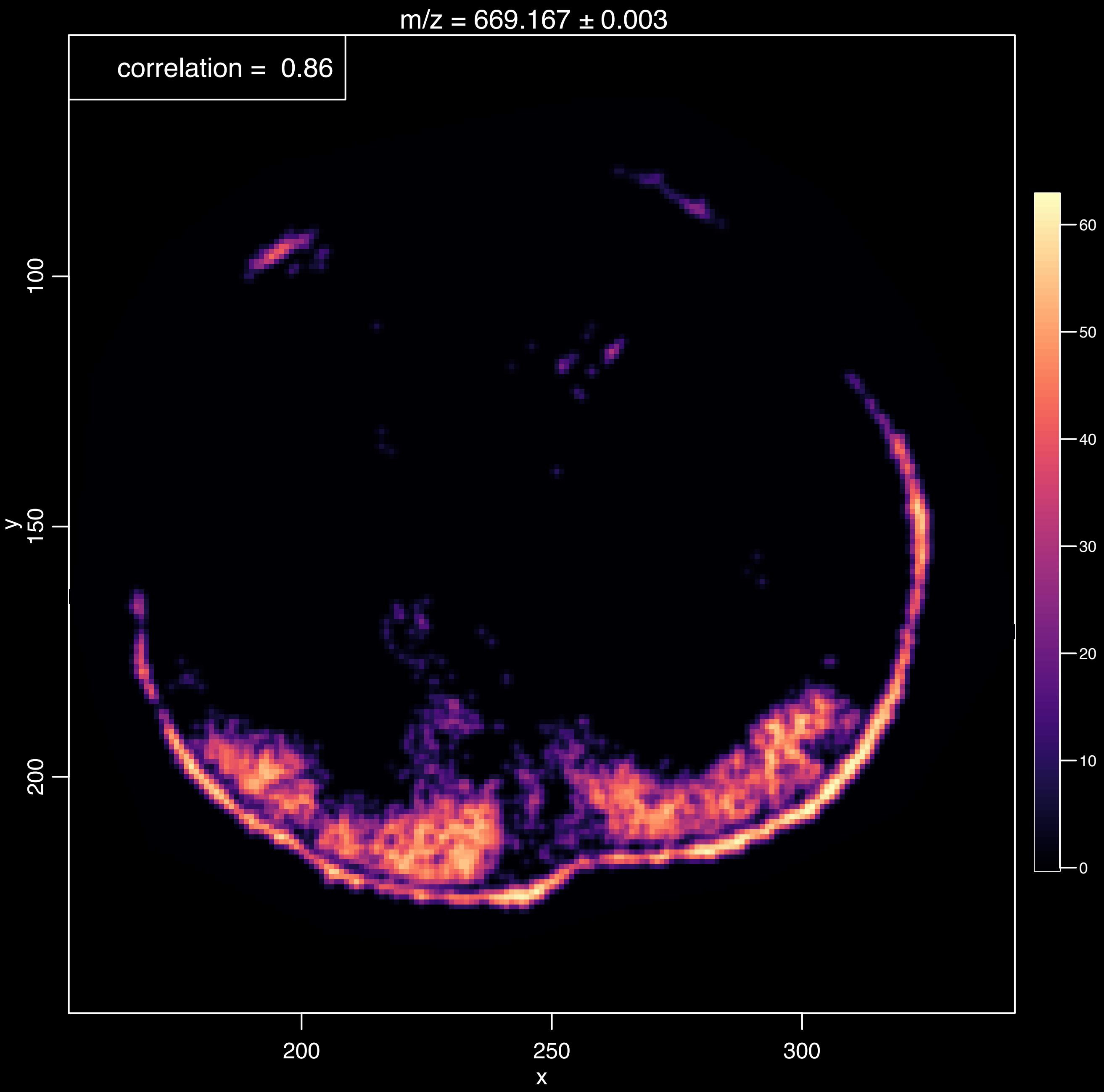

m/z = 669.167 ± 0.003
correlation = 0.86
y
100
150
200
x
200
250
300
0
10
20
30
40
50
60

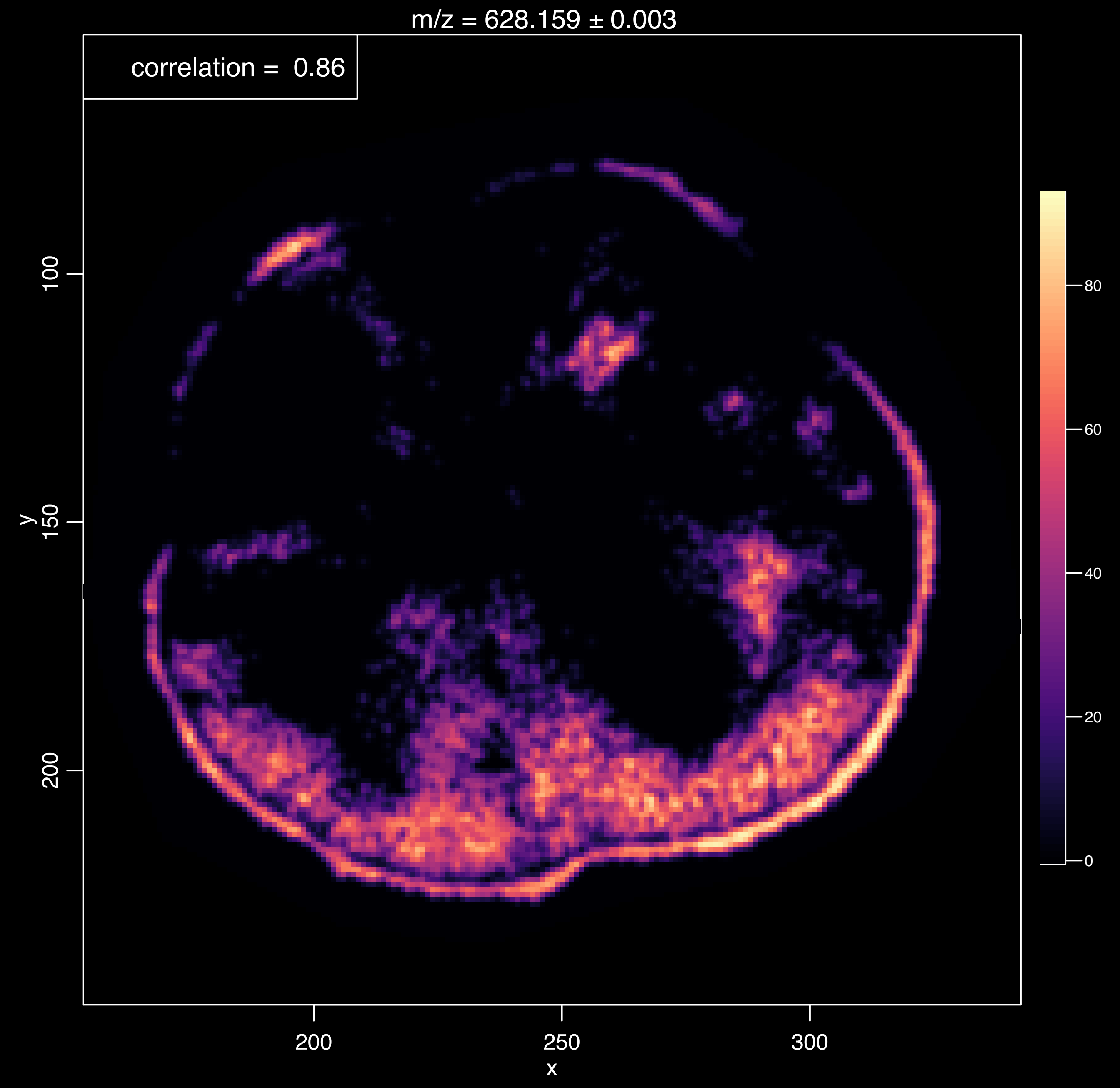

m/z = 628.159 ± 0.003
correlation = 0.86
100
150
200
y
200
250
300
x
80
60
40
20
0

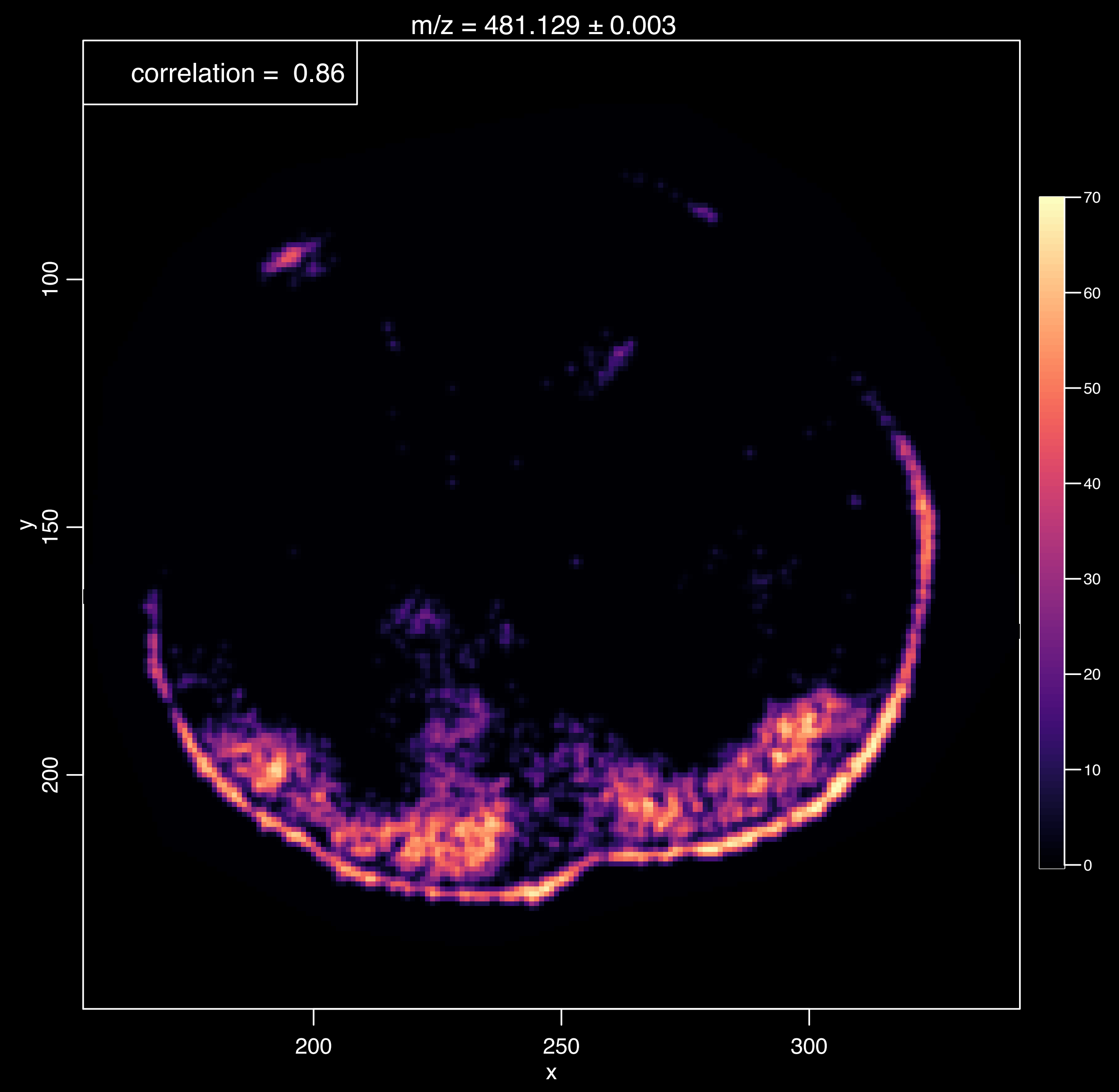

m/z = 481.129 ± 0.003
correlation = 0.86
y
100
150
200
x
200
250
300
70
60
50
40
30
20
10
0

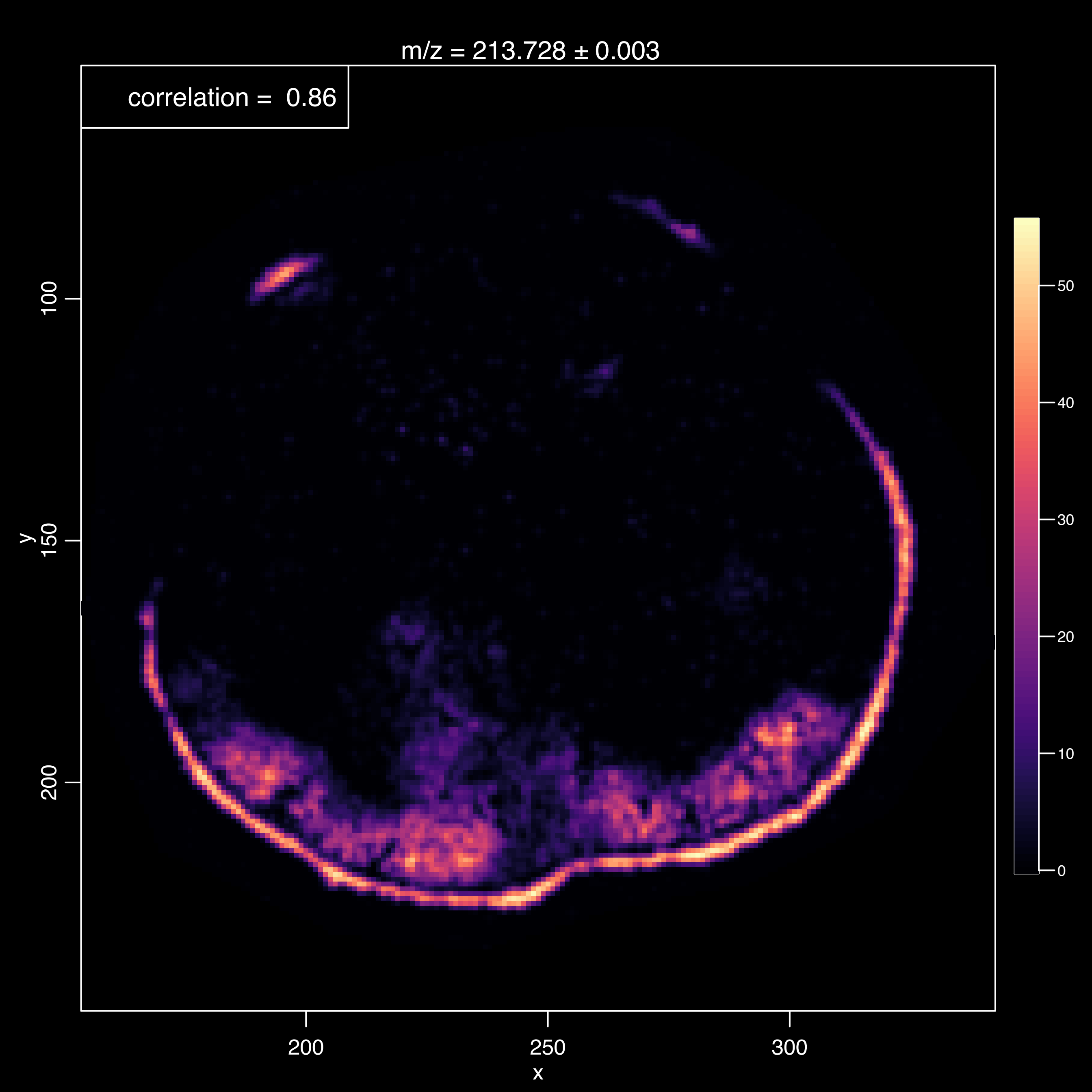

m/z = 213.728 ± 0.003
correlation = 0.86
100
150
200
y
200
250
300
x
50
40
30
20
10
0

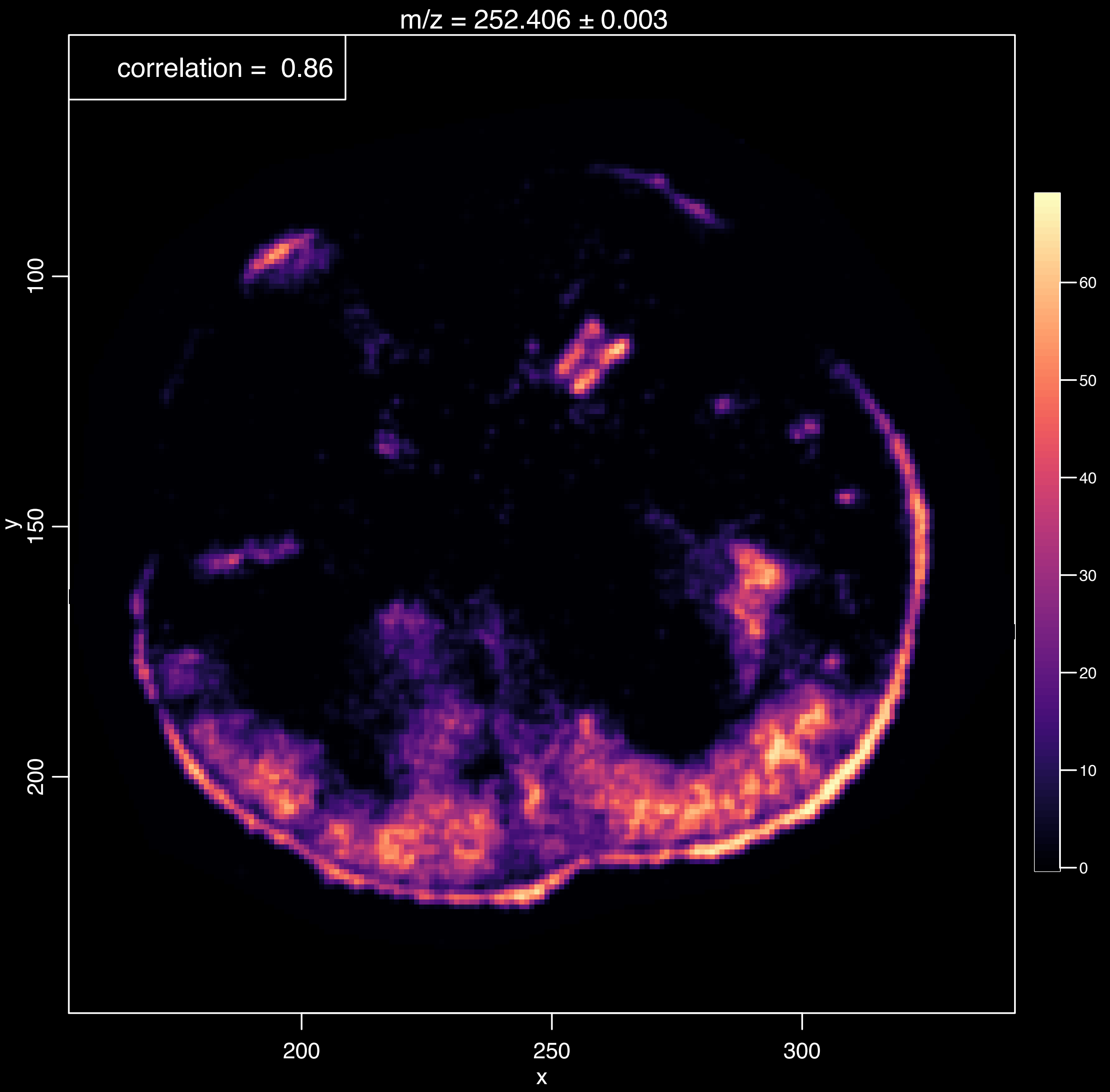
m/z = 252.406 ± 0.003
correlation = 0.86
100
150
200
y
200
250
300
x
60
50
40
30
20
10
0

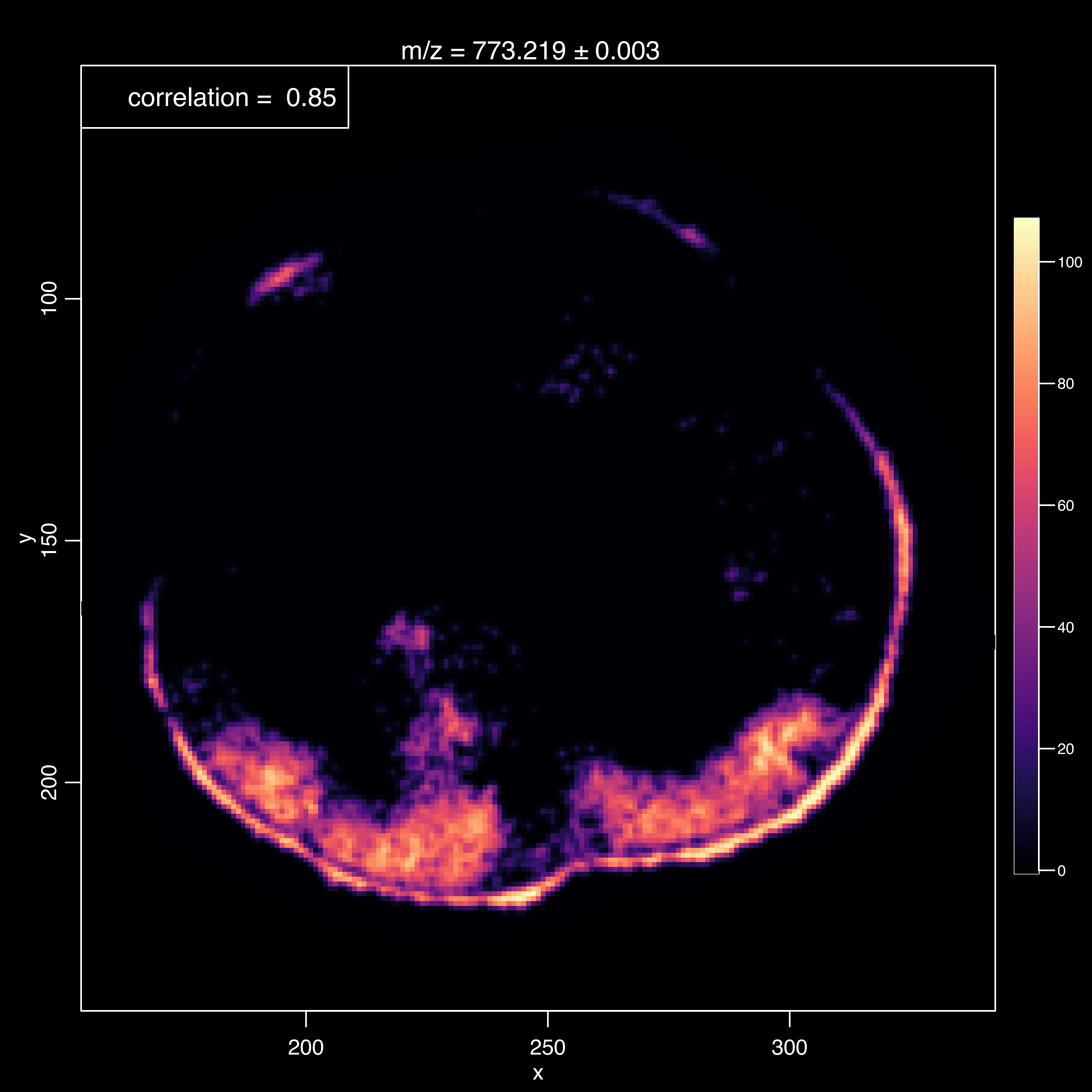

m/z = 773.219 ± 0.003
correlation = 0.85
100
150
200
y
200
250
300
x
100
80
60
40
20
0

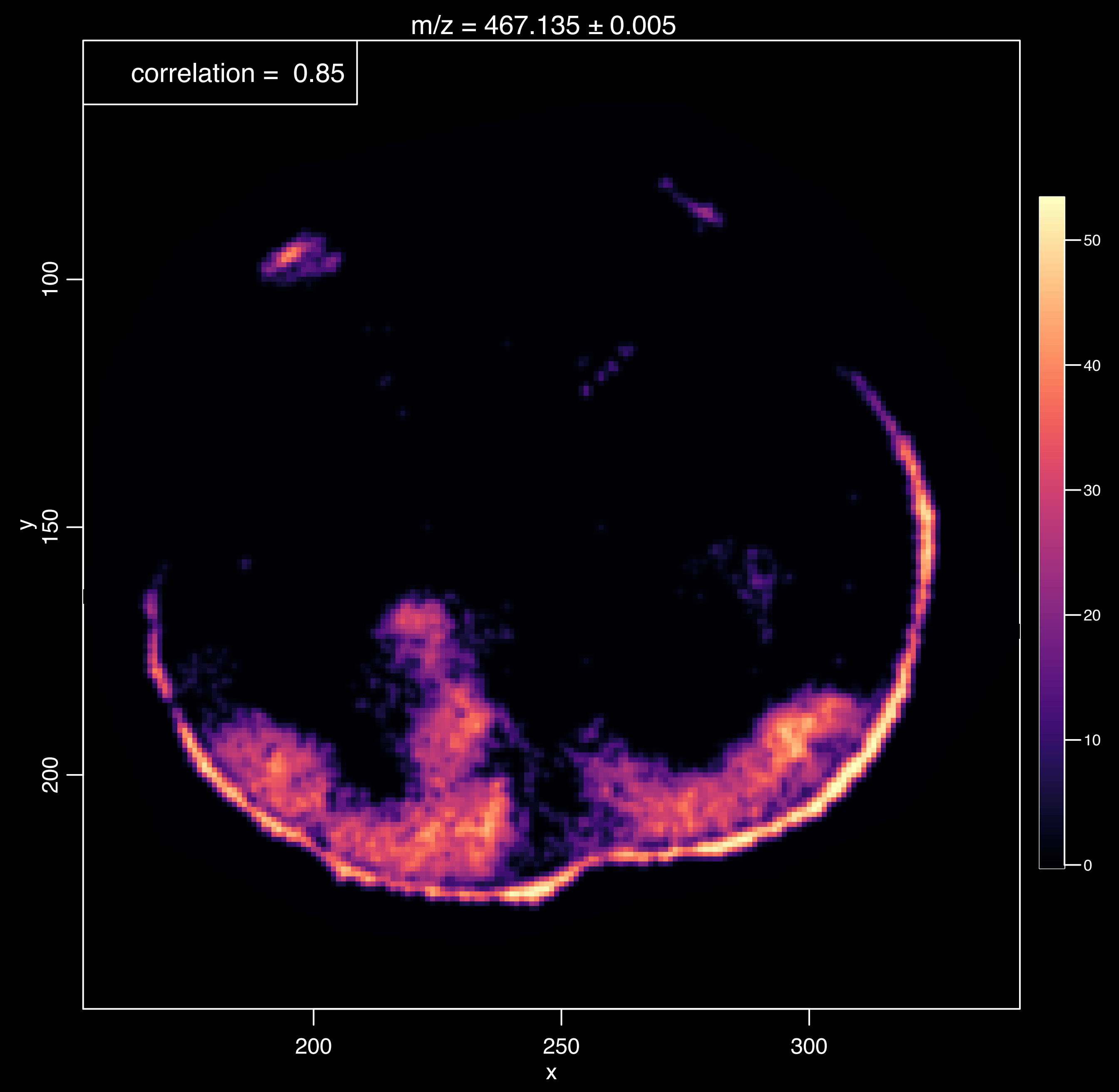
m/z = 467.135 ± 0.005
correlation = 0.85
100
150
200
y
200
250
300
x
0
10
20
30
40
50

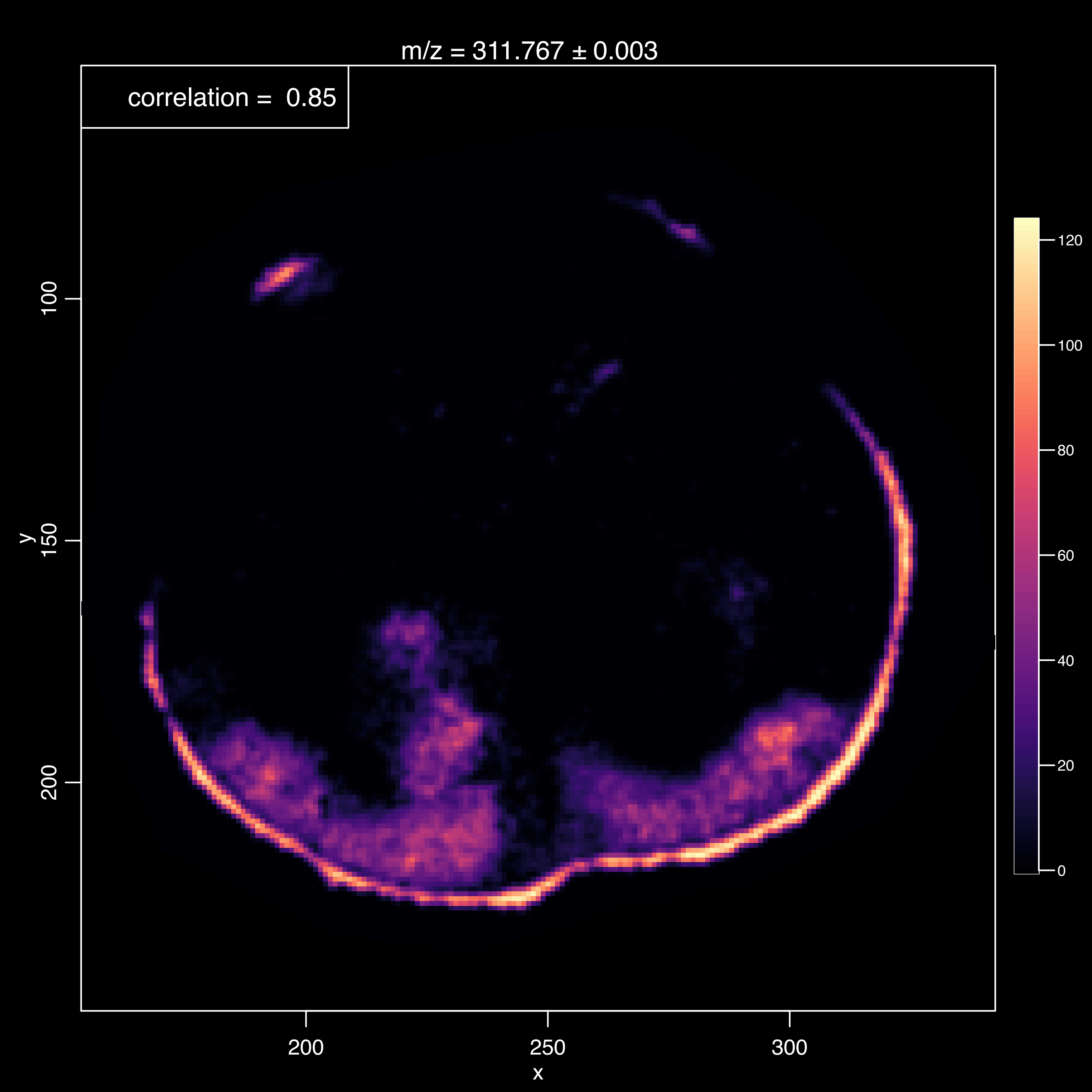
m/z = 311.767 ± 0.003
correlation = 0.85
y
100
150
200
x
200
250
300
0
20
40
60
80
100
120

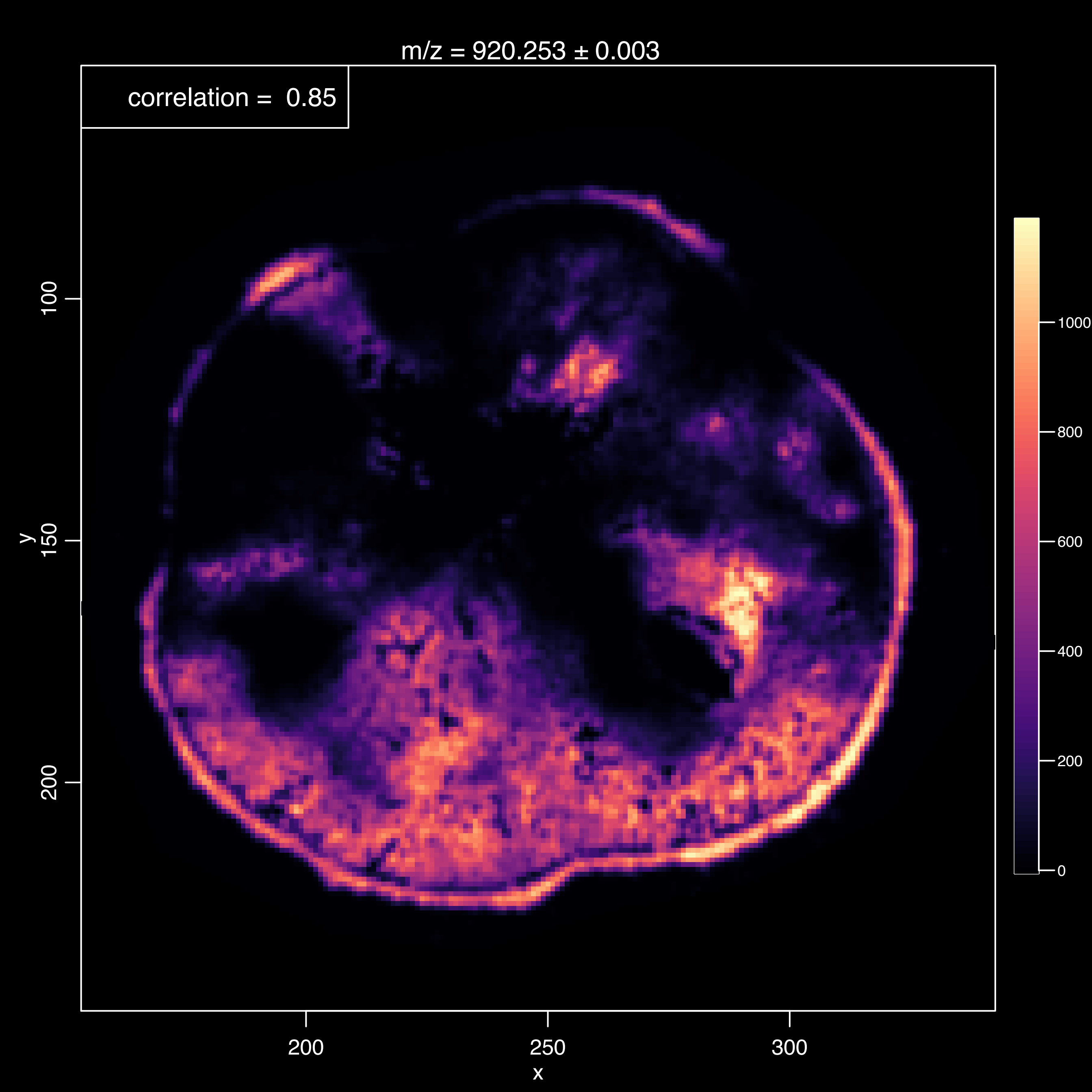
m/z = 920.253 ± 0.003
correlation = 0.85
100
150
200
y
200
250
300
x
1000
800
600
400
200
0

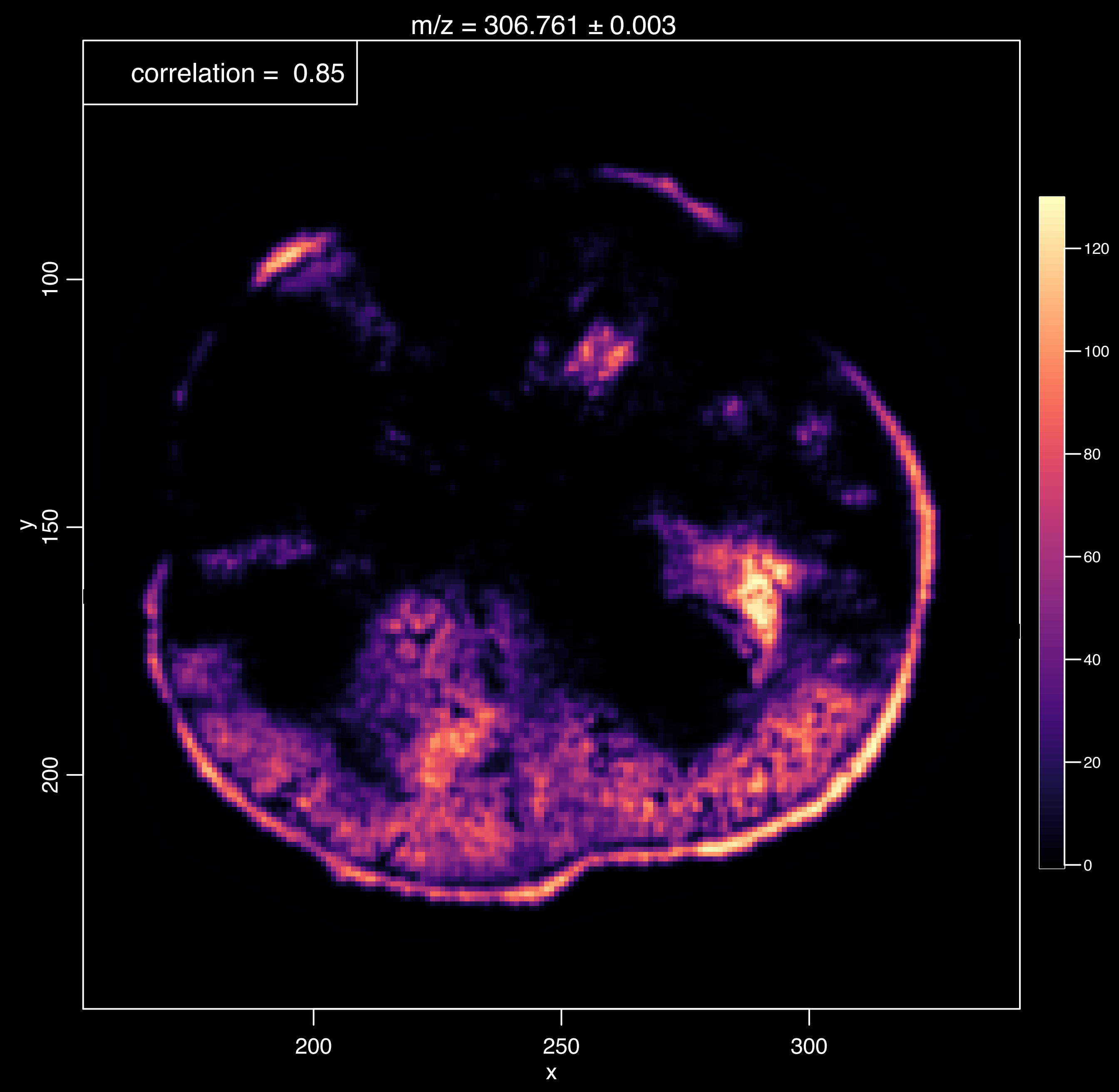
m/z = 306.761 ± 0.003
correlation = 0.85
y
100
150
200
x
200
250
300
0
20
40
60
80
100
120

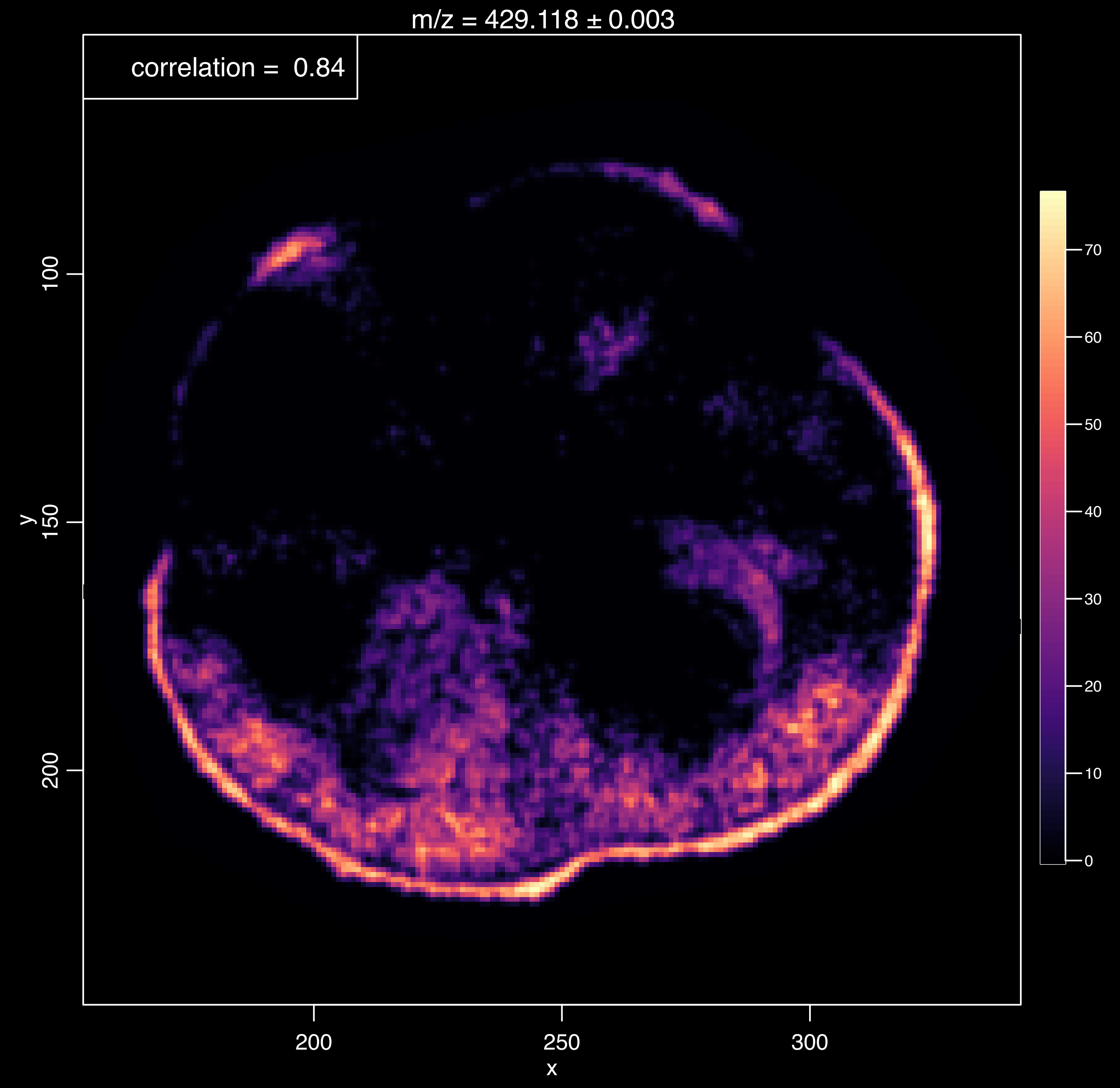

m/z = 429.118 ± 0.003
correlation = 0.84
y
100
150
200
x
200
250
300
0
10
20
30
40
50
60
70

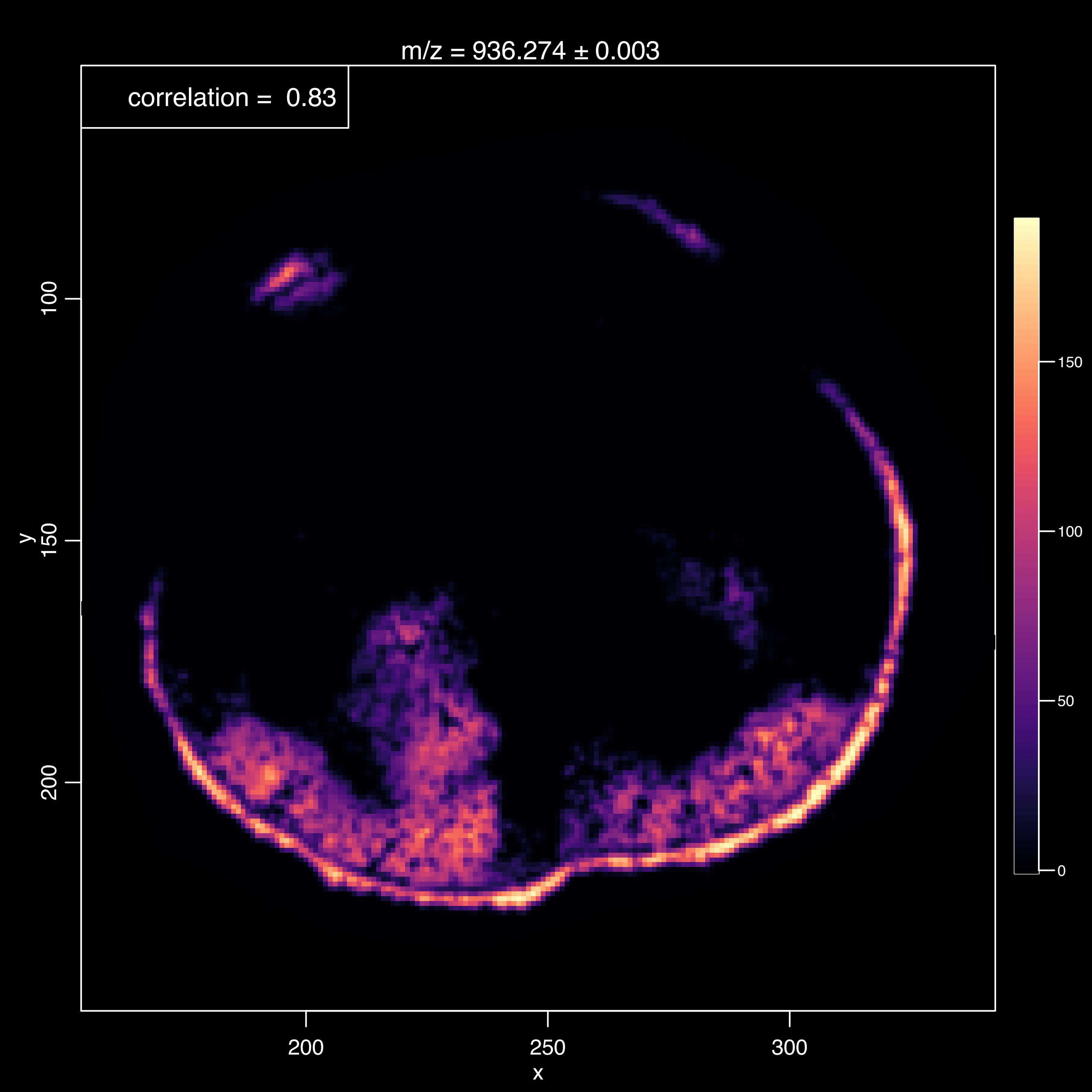

m/z = 936.274 ± 0.003
correlation = 0.83
100
150
200
y
200
250
300
x
0
50
100
150

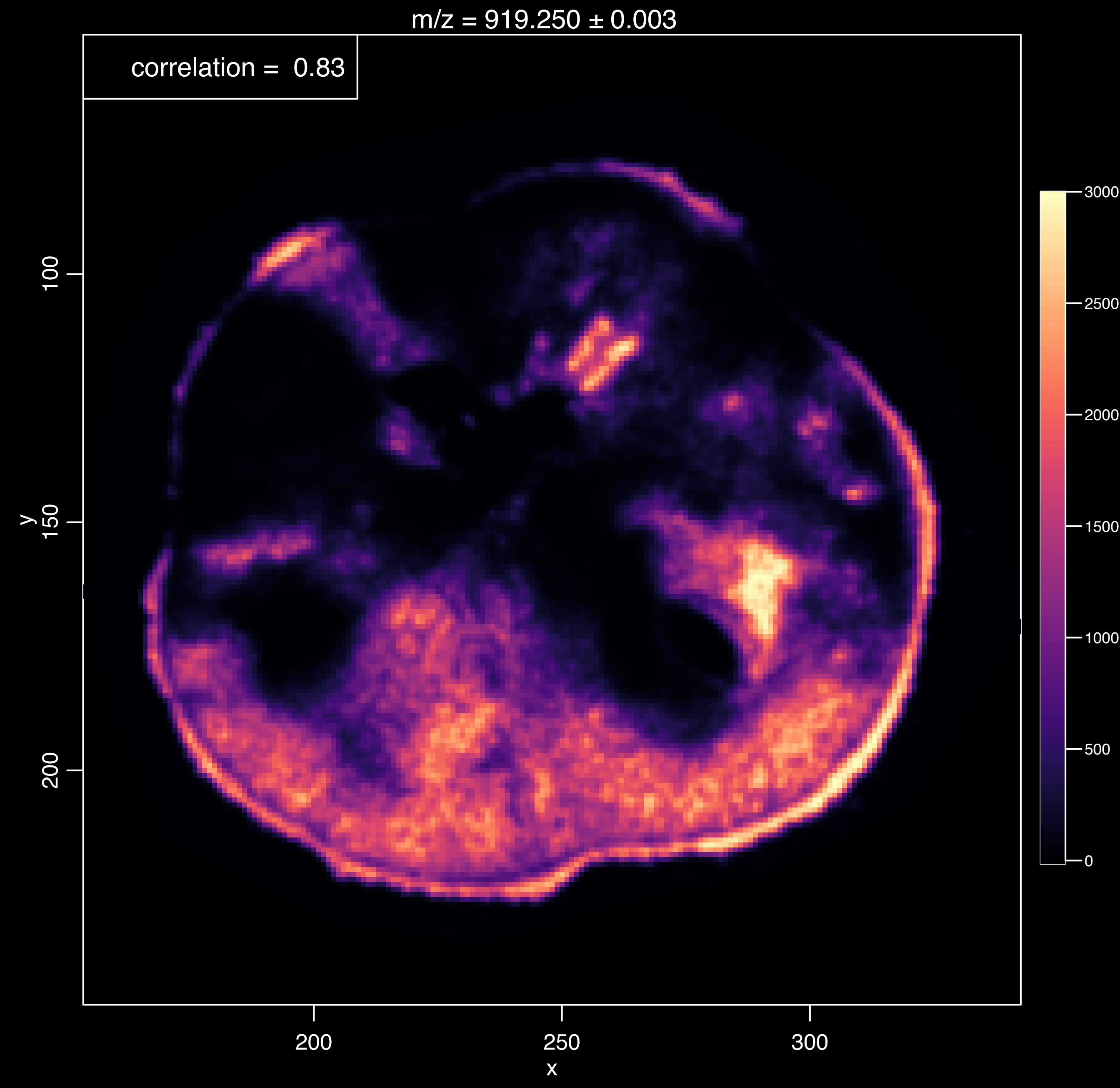

m/z = 919.250 ± 0.003
correlation = 0.83
100
150
200
y
200
250
300
x
3000
2500
2000
1500
1000
500
0

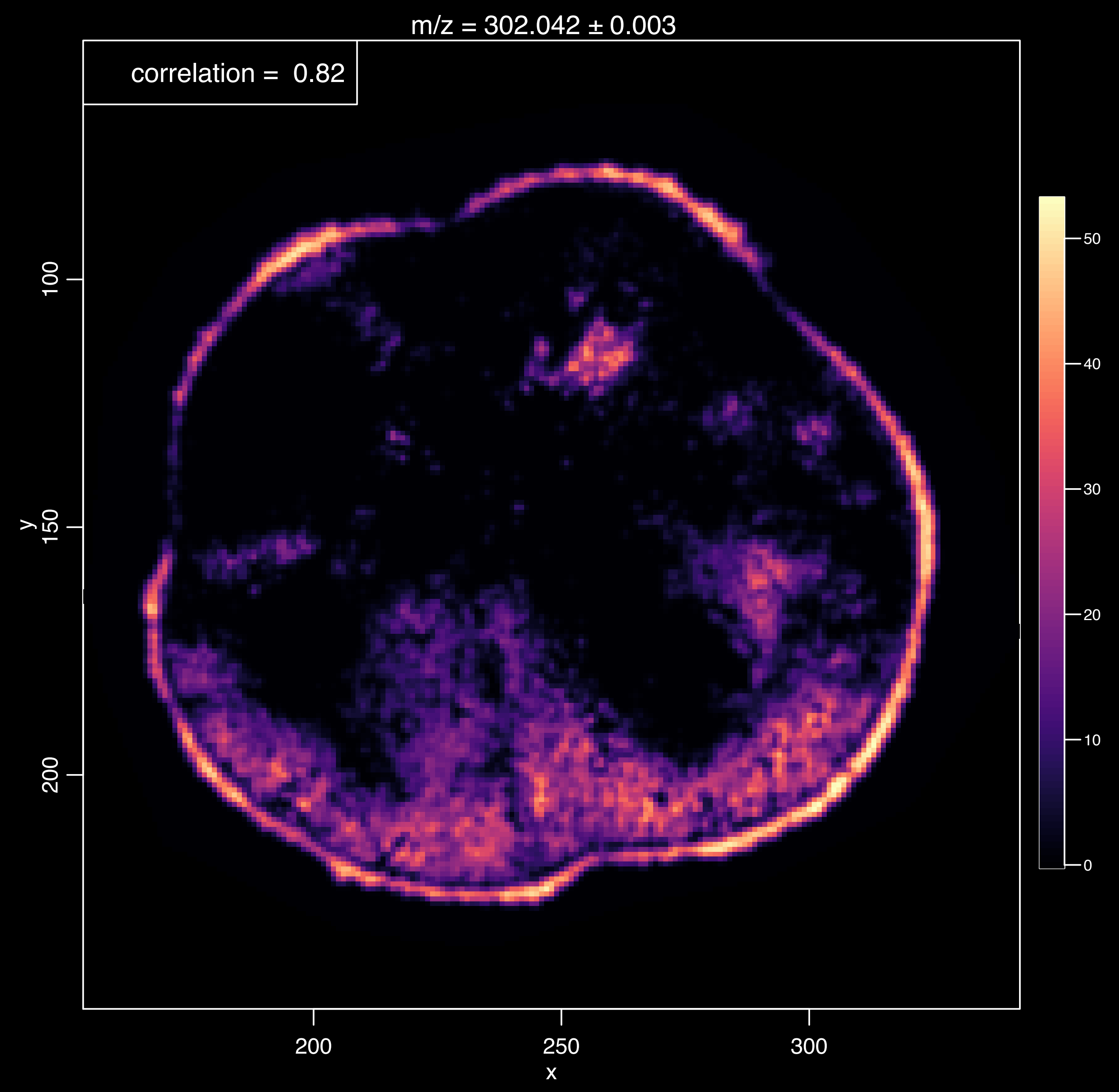

m/z = 302.042 ± 0.003
correlation = 0.82
y
100
150
200
x
200
250
300
50
40
30
20
10
0

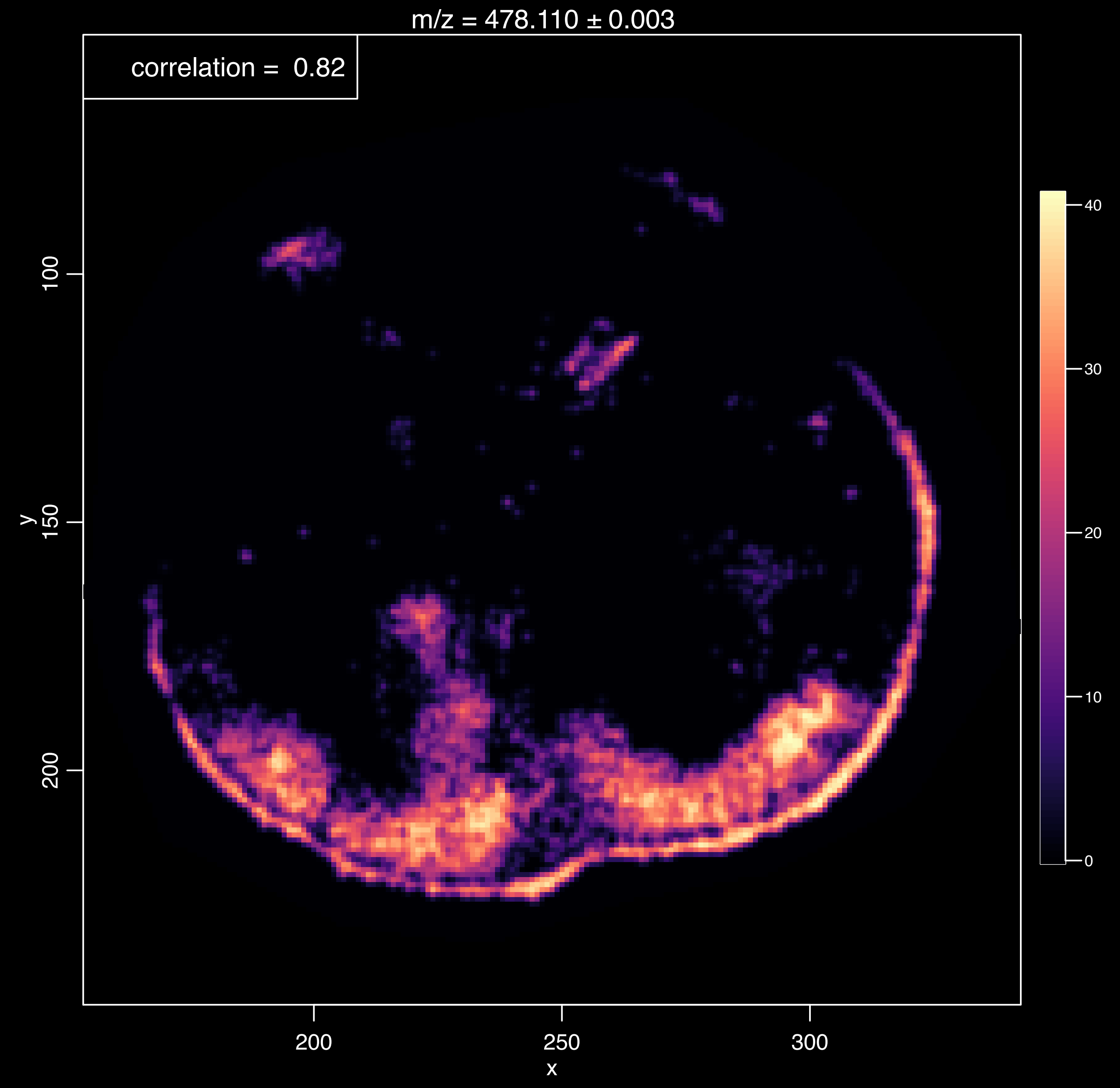

m/z = 478.110 ± 0.003
correlation = 0.82
100
150
200
y
200
250
300
x
40
30
20
10
0

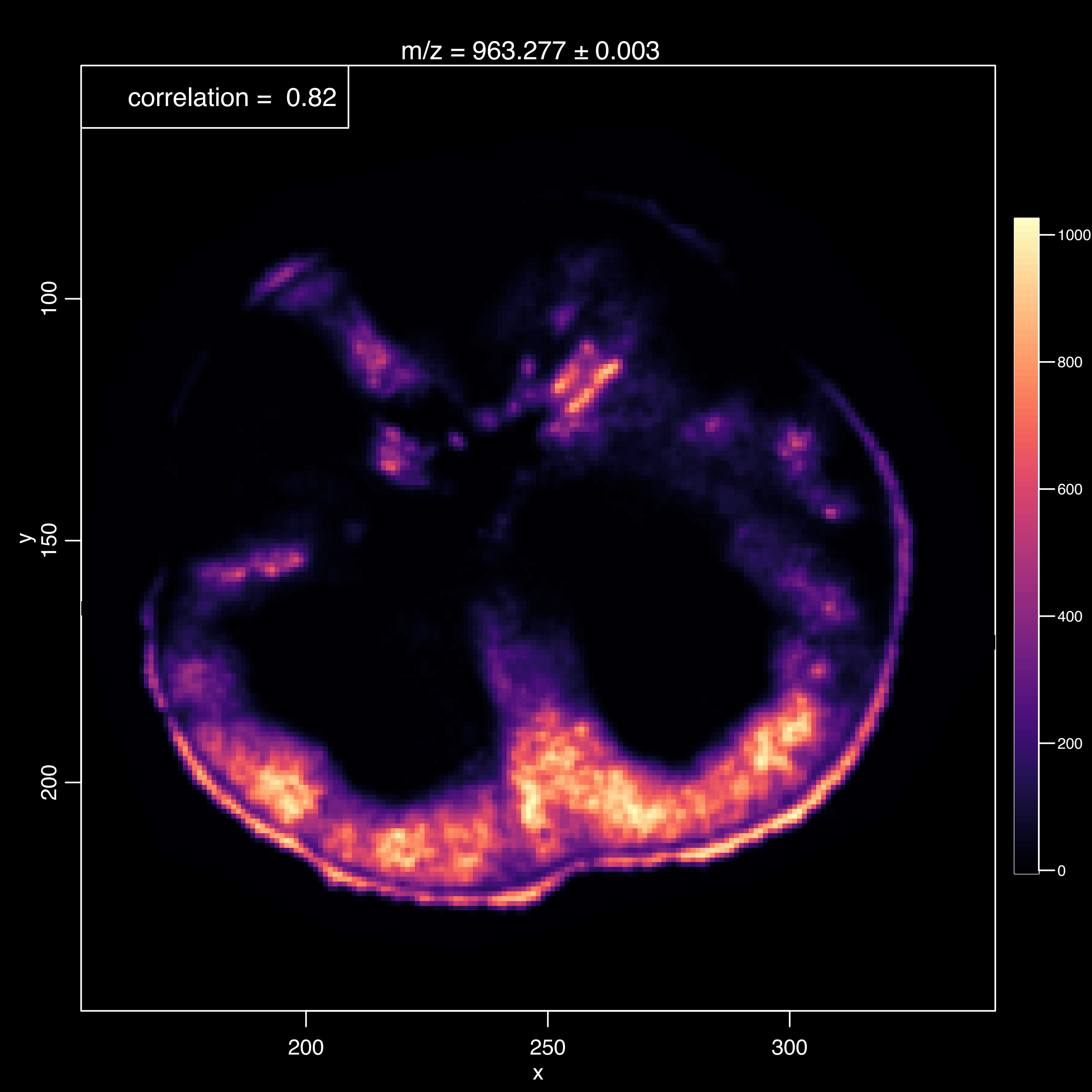

m/z = 963.277 ± 0.003
correlation = 0.82
y
100
150
200
x
200
250
300
1000
800
600
400
200
0

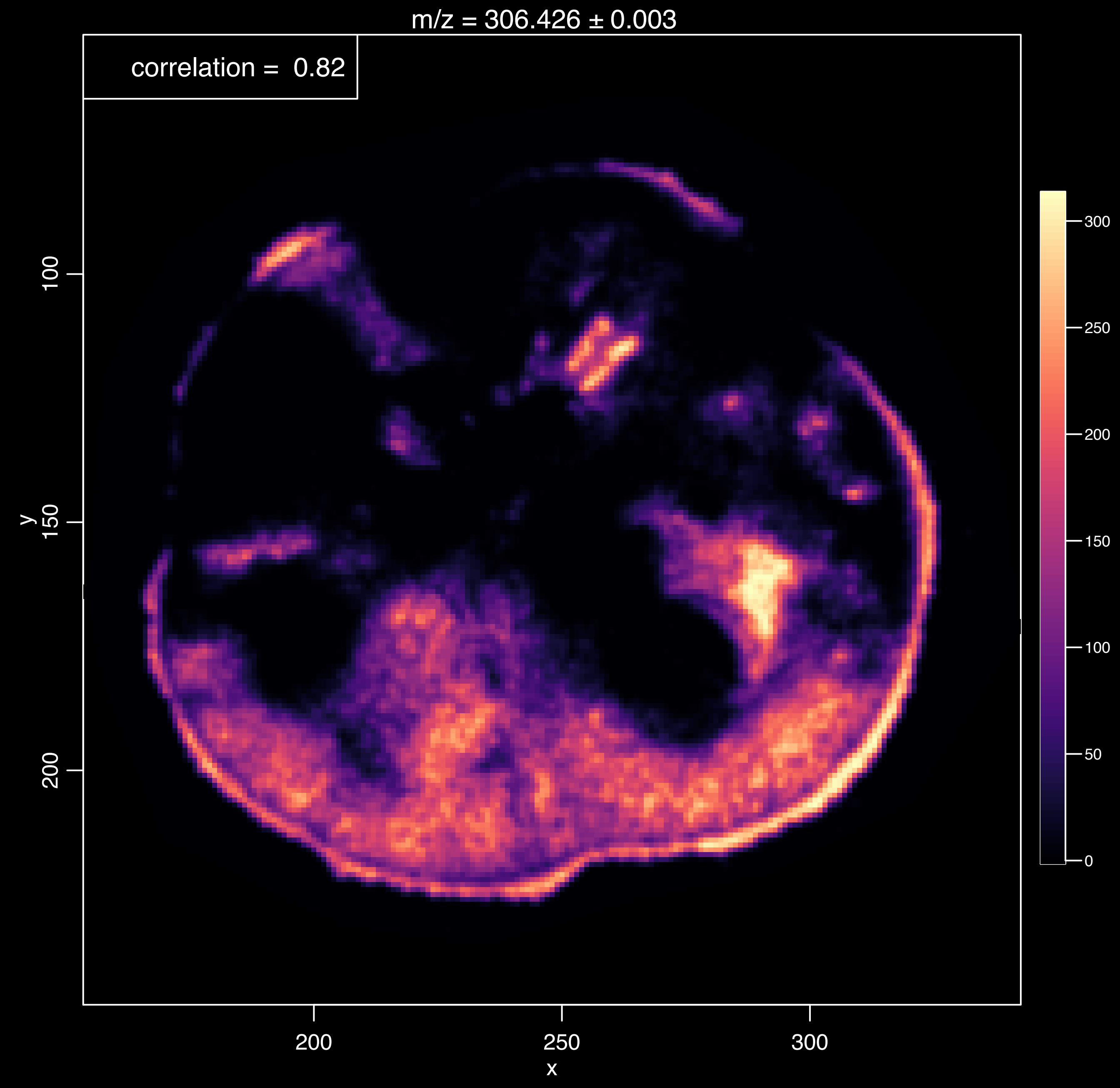

m/z = 306.426 ± 0.003
correlation = 0.82
100
150
200
y
200
250
300
x
300
250
200
150
100
50
0

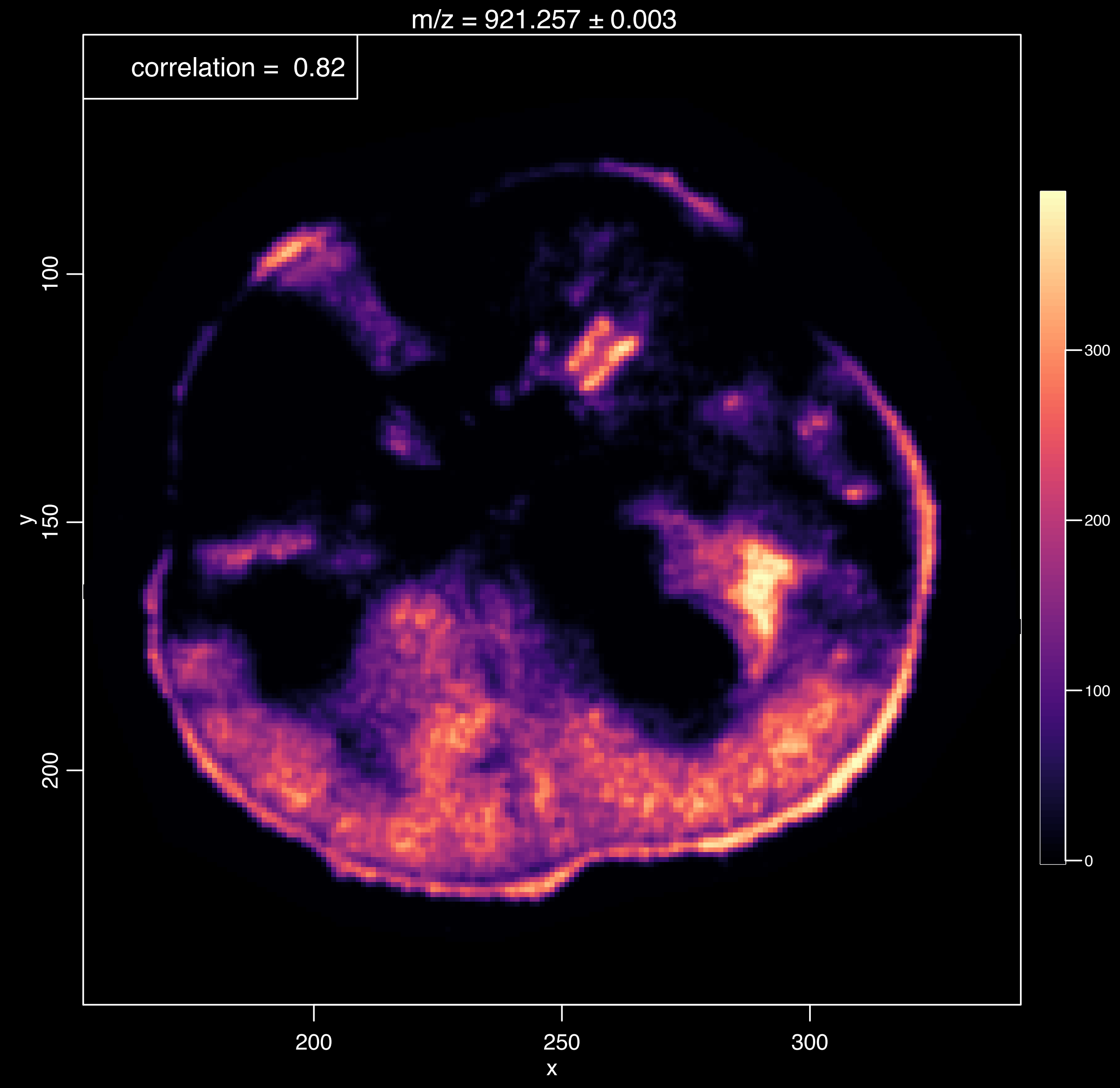

m/z = 921.257 ± 0.003
correlation = 0.82
y
100
150
200
x
200
250
300
0
100
200
300

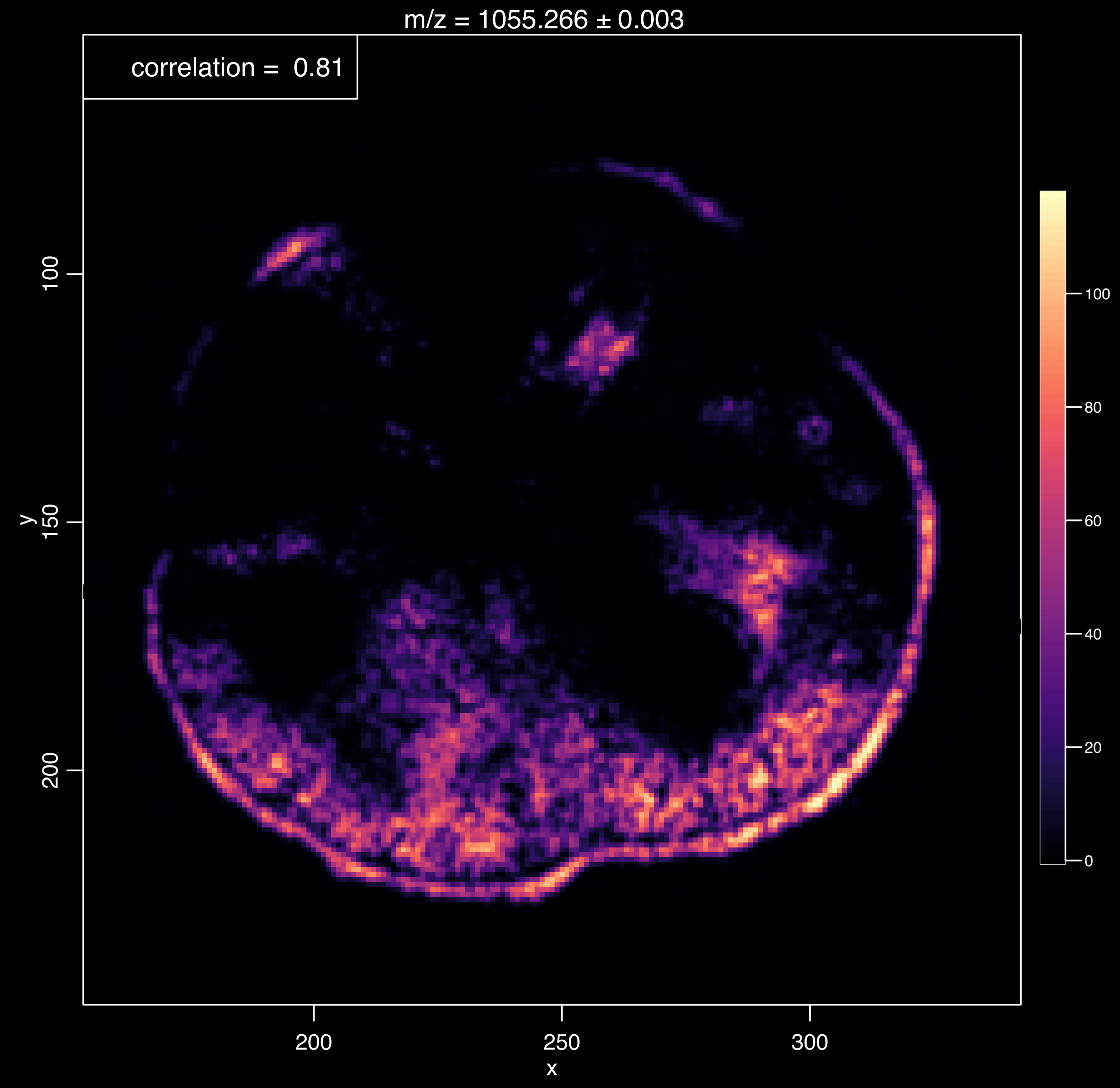

m/z = 1055.266 ± 0.003
correlation = 0.81
y
100
150
200
x
200
250
300
0
20
40
60
80
100

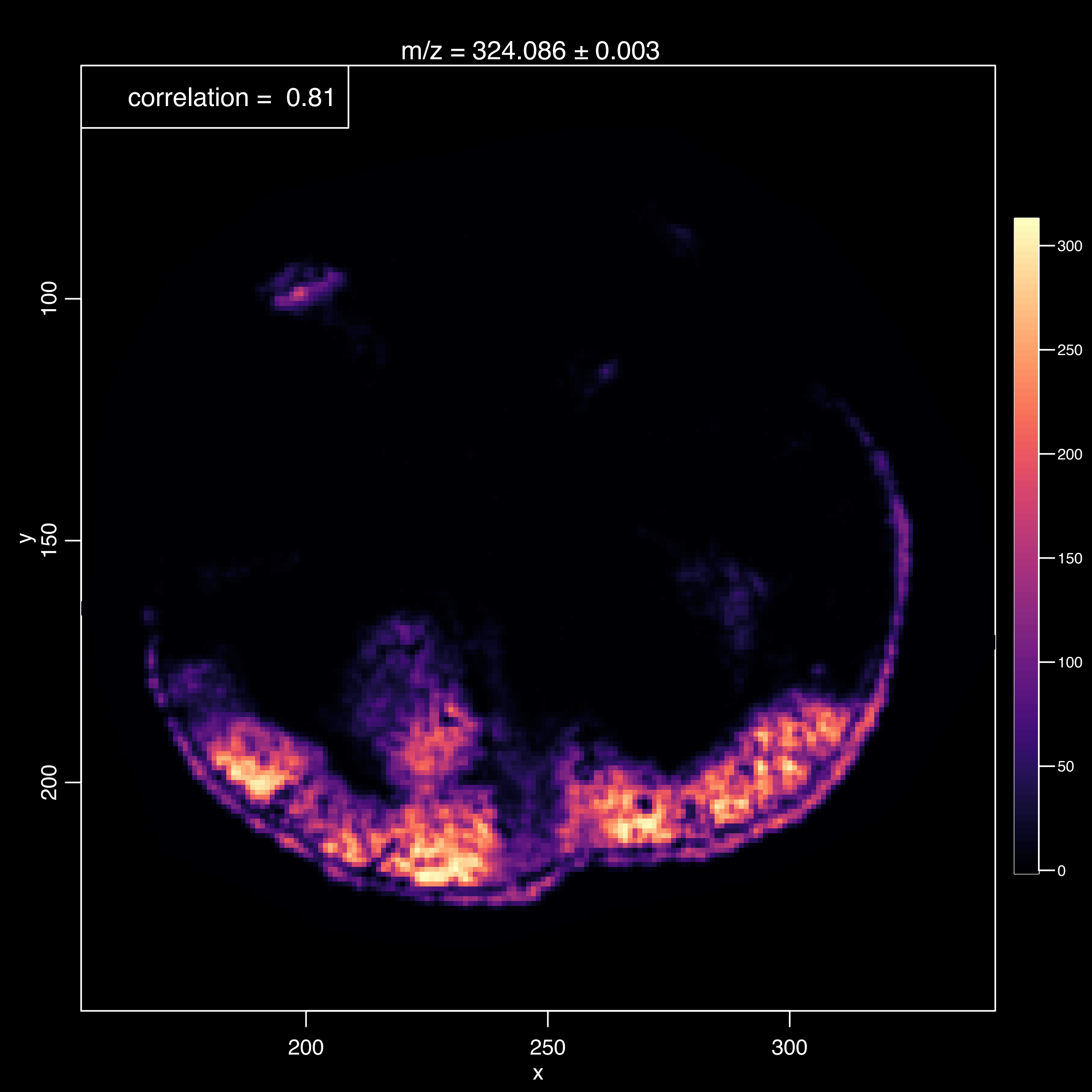
m/z = 324.086 ± 0.003
correlation = 0.81
y
100
150
200
x
200
250
300
0
50
100
150
200
250
300

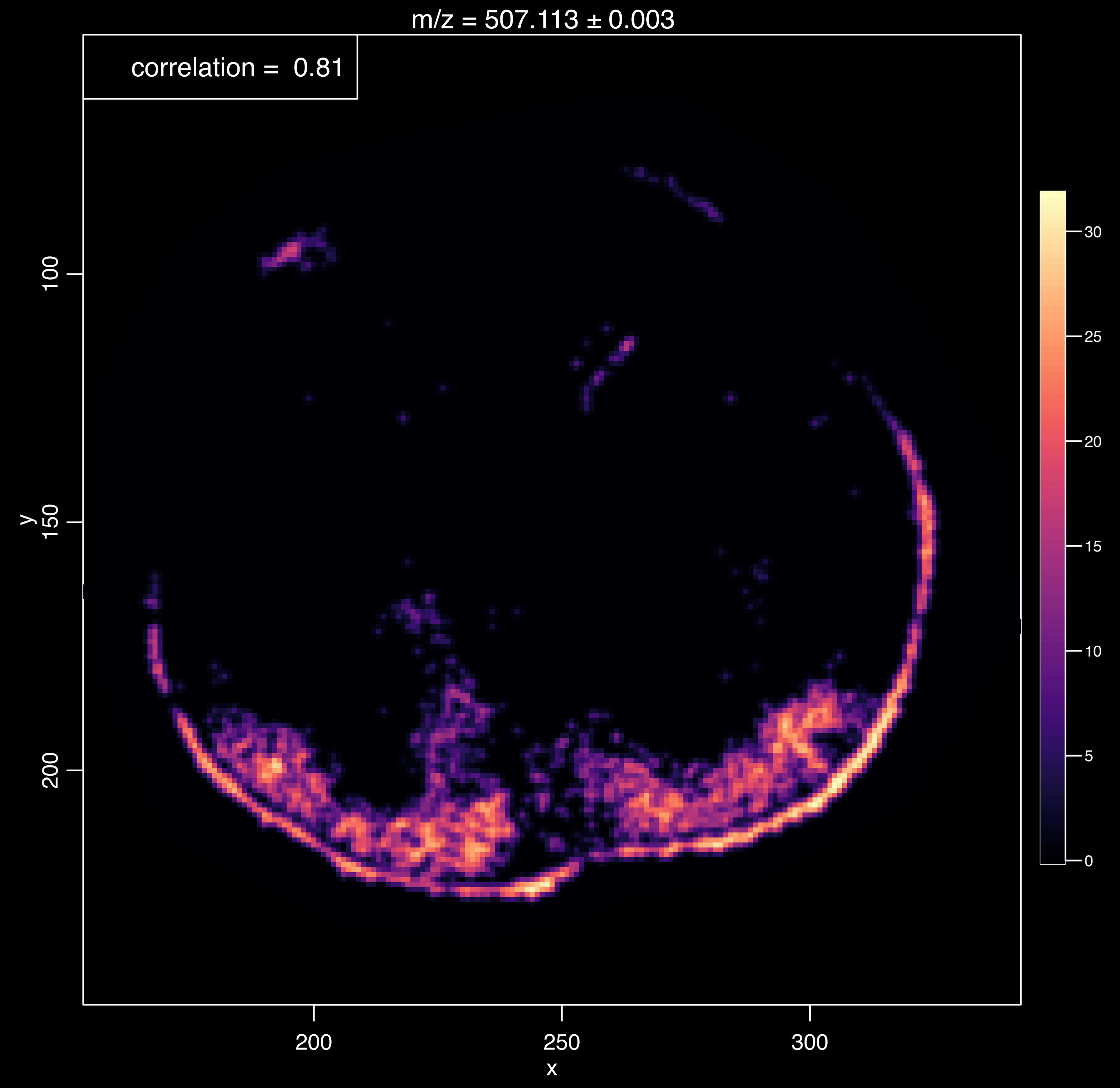

m/z = 507.113 ± 0.003
correlation = 0.81
y
100
150
200
x
200
250
300
0
5
10
15
20
25
30

Figure S7

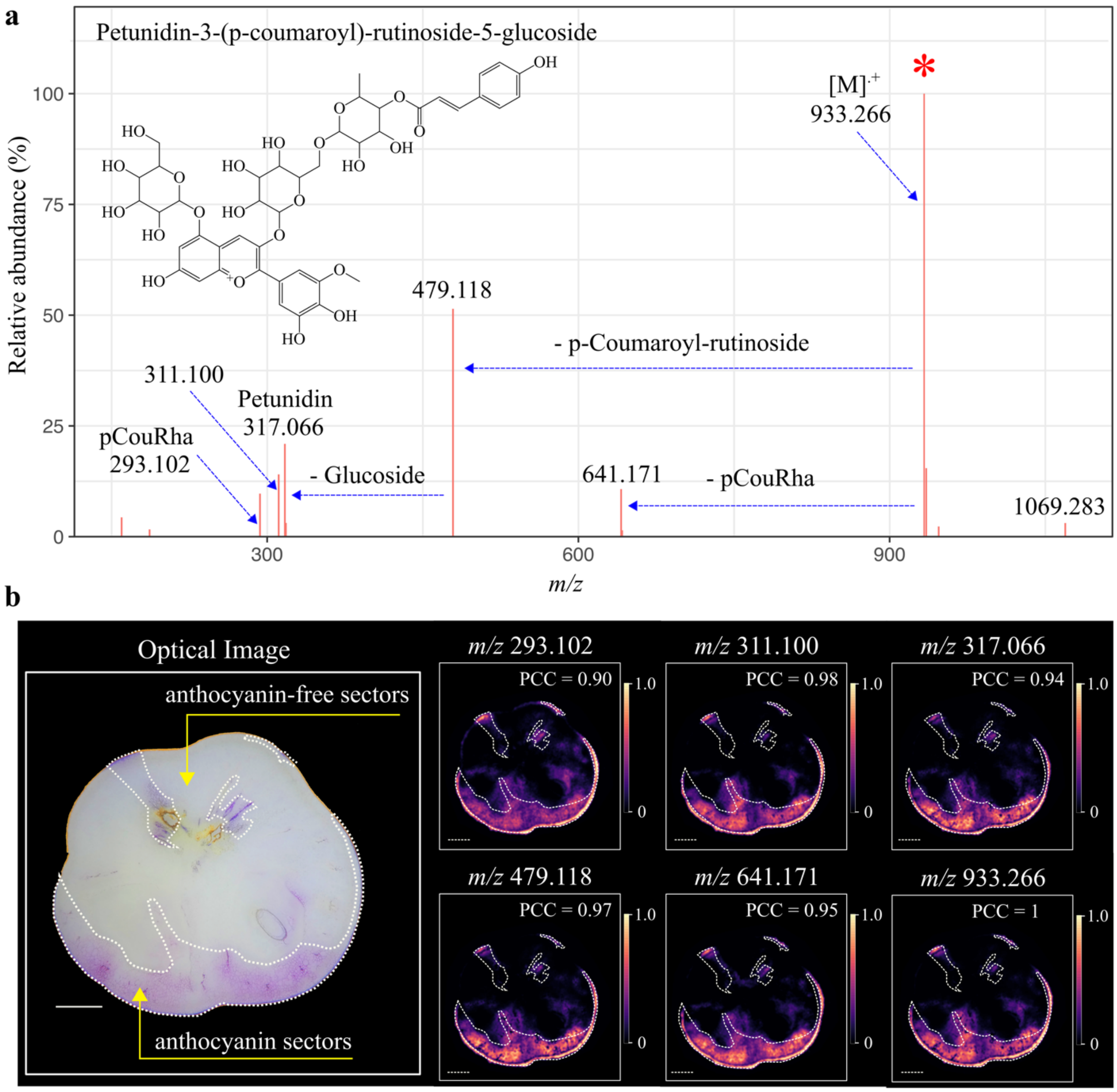

Figure S7 PICA-assisted petunidin 3-(p-coumaroyl)-rutinoside-5-glucoside identification. (a) "Pseudo MS/MS spectrum" of petunidin 3-(p-coumaroyl)-rutinoside-5-glucoside ($[M]^{+\cdot}$, *m/z* 933.266). (b) Optical image of the analyzed tomato fruit section, and MALDI images of six representative colocalized ions. MALDI images were generated using the exact *m/z* value with a

mass bin width of ± 0.003 Da and optimized with gaussian smoothing and contrast enhancement. The color scale indicates the range of total ion current normalized intensity. Scale bar, 2mm.

Figure S8 MS images of ions highly and moderately colocalized (PCC score >= 0.8) with petunidin 3-(p-coumaroyl)-rutinoside-5-glucoside peak *m/z* 933.266.

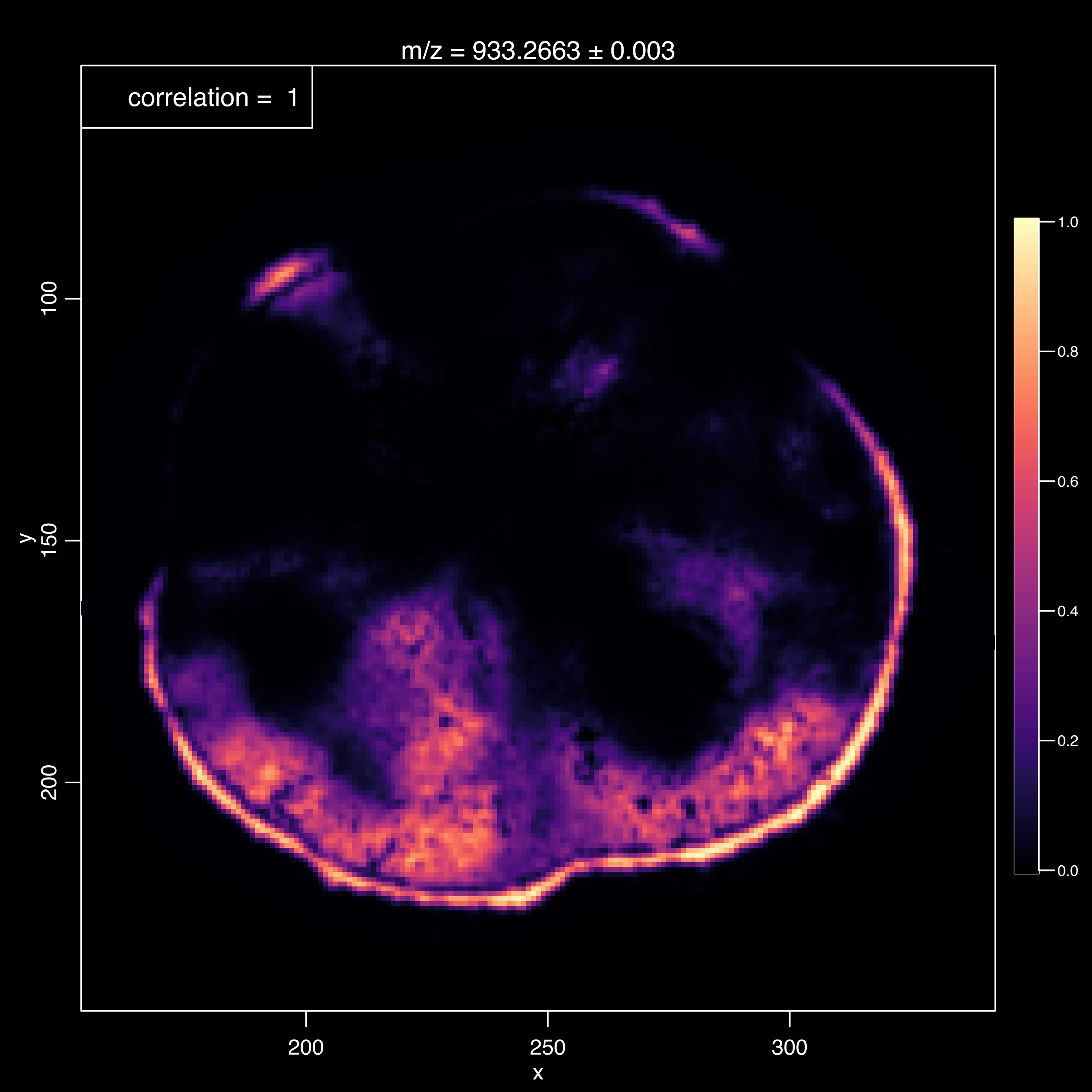

m/z = 933.2663 ± 0.003
correlation = 1
y
100
150
200
x
200
250
300
1.0
0.8
0.6
0.4
0.2
0.0

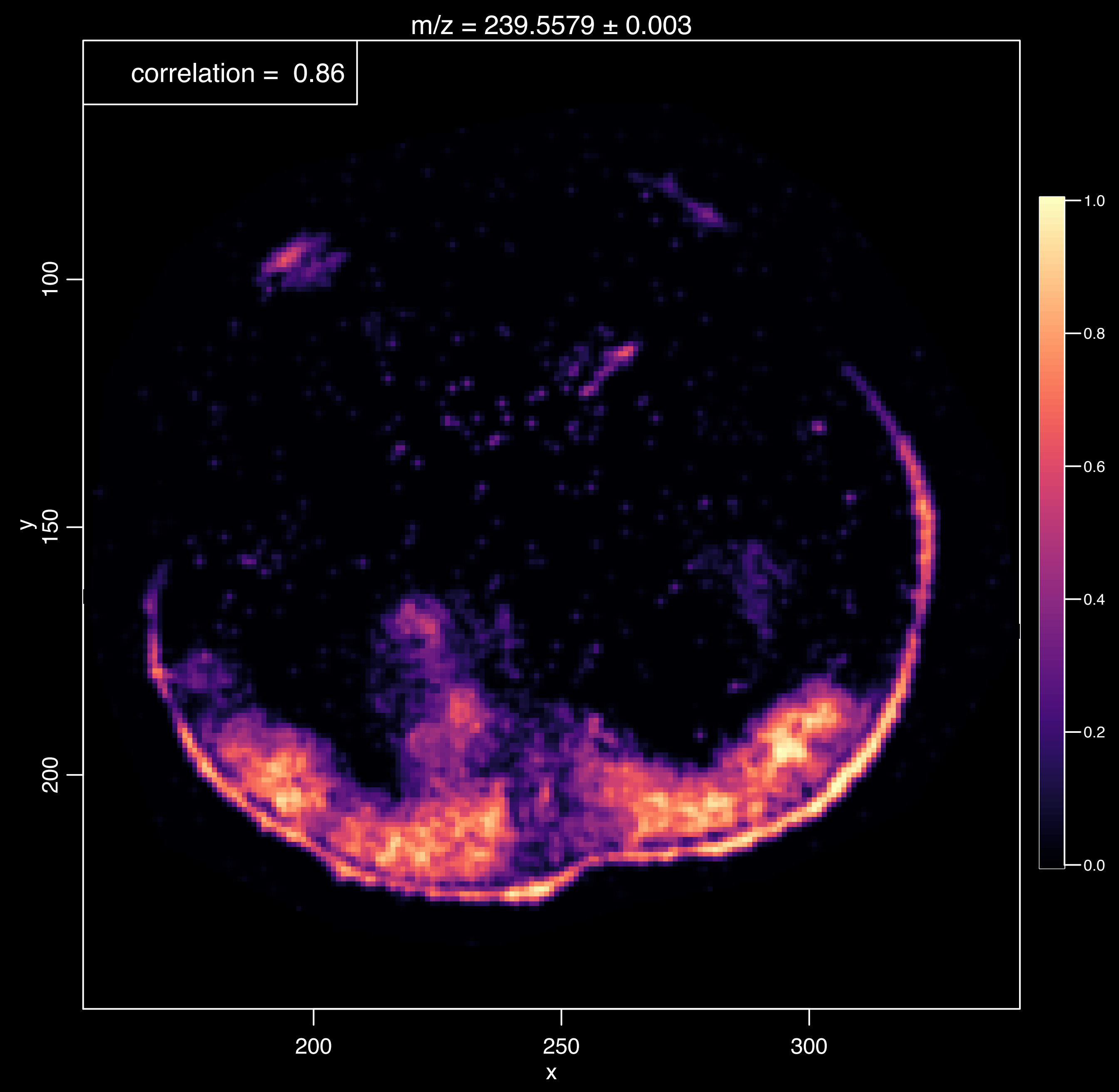
m/z = 239.5579 ± 0.003
correlation = 0.86
y
100
150
200
x
200
250
300
1.0
0.8
0.6
0.4
0.2
0.0

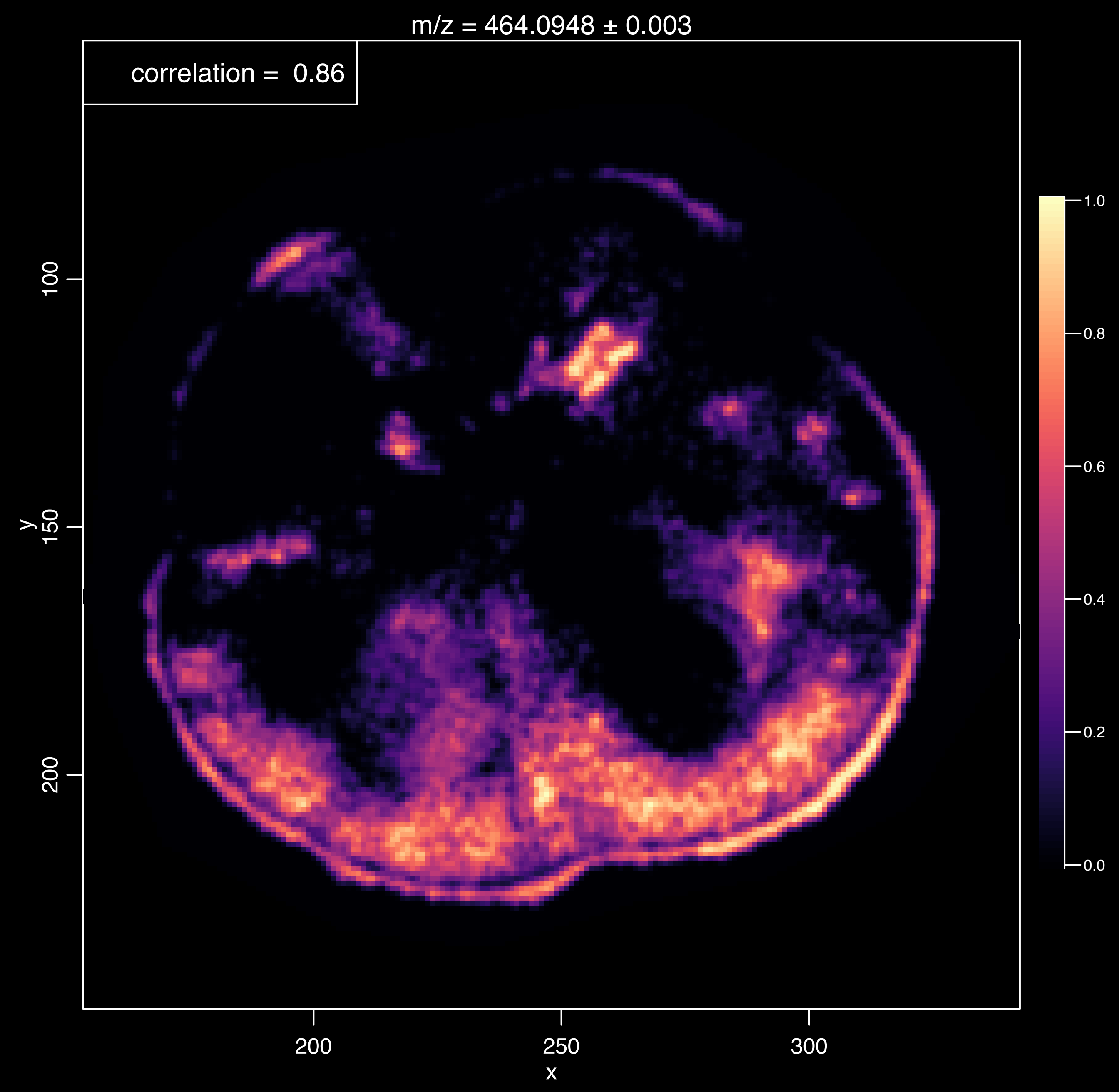

m/z = 464.0948 ± 0.003
correlation = 0.86
y
100
150
200
x
200
250
300
1.0
0.8
0.6
0.4
0.2
0.0

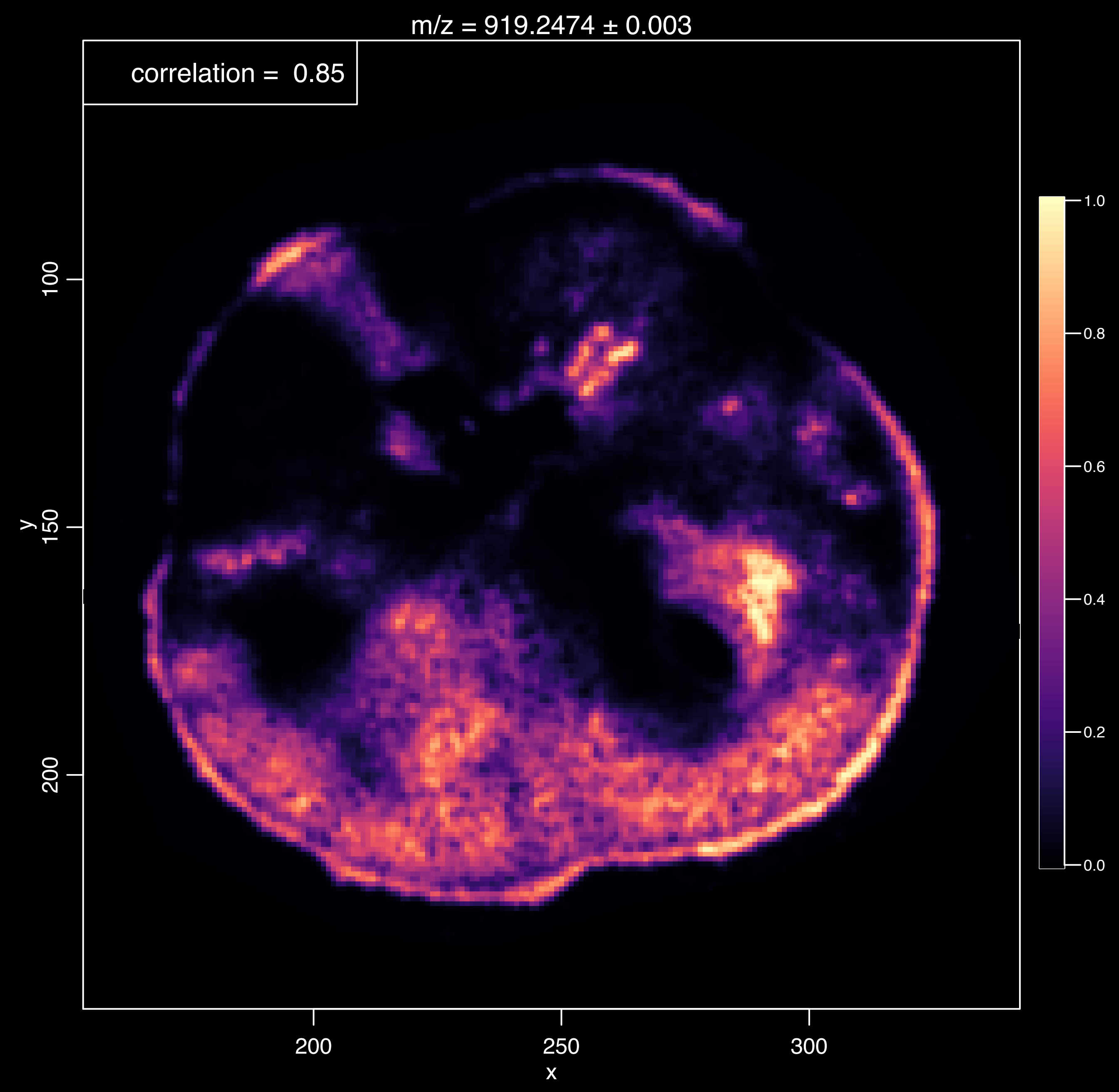

m/z = 919.2474 ± 0.003
correlation = 0.85
y
100
150
200
x
200
250
300
1.0
0.8
0.6
0.4
0.2
0.0

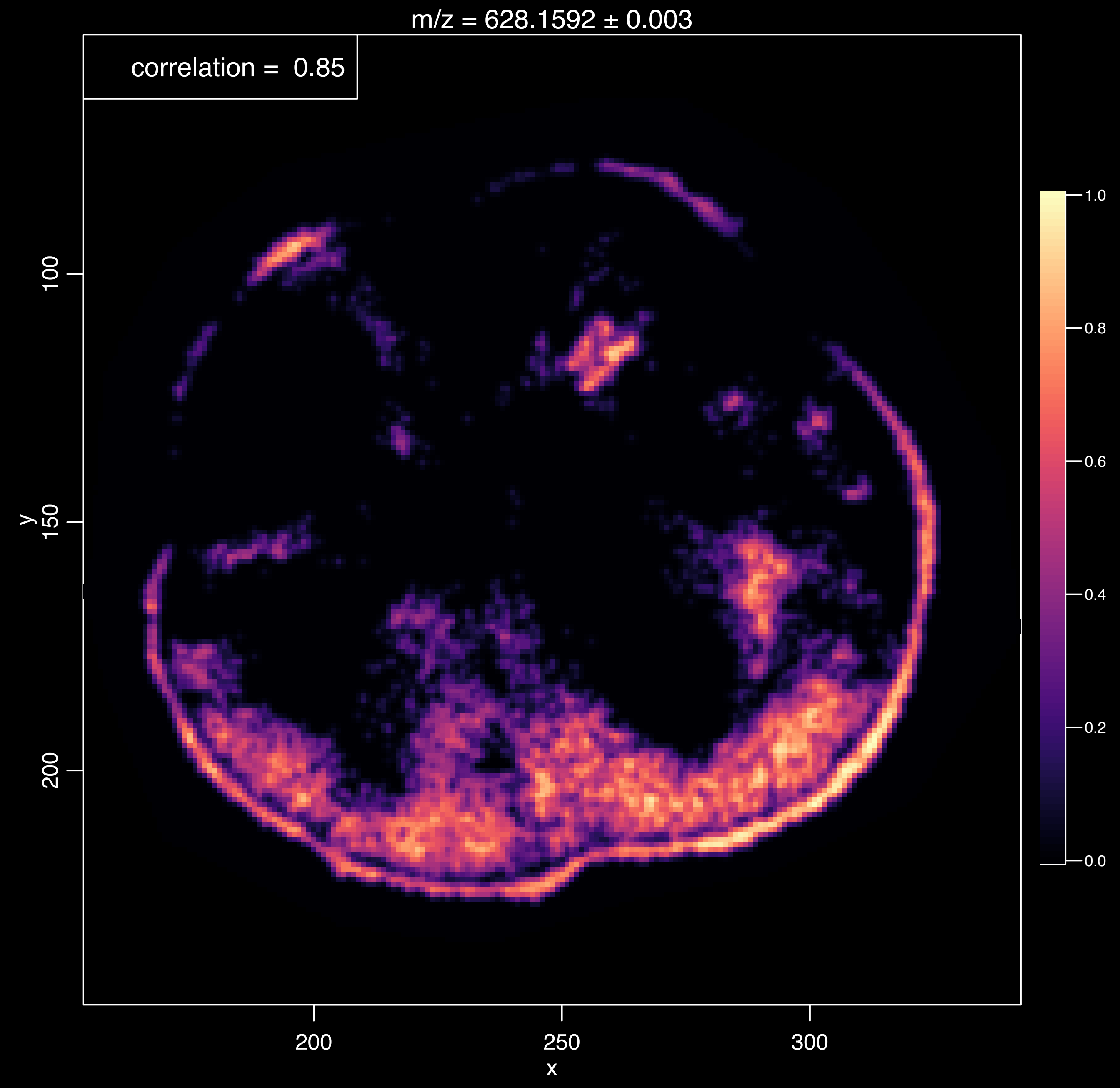

m/z = 628.1592 ± 0.003
correlation = 0.85
y
100
150
200
x
200
250
300
1.0
0.8
0.6
0.4
0.2
0.0

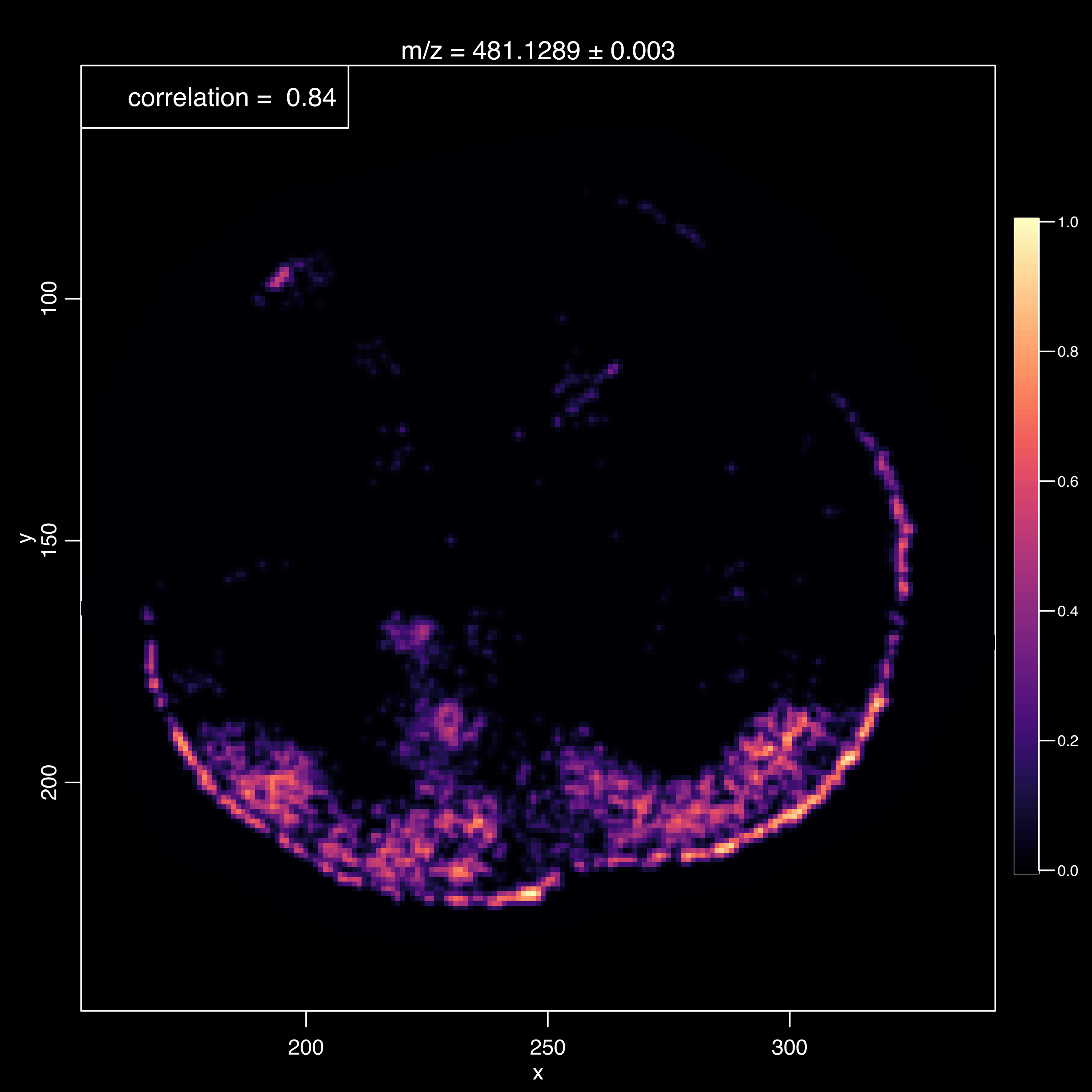
m/z = 481.1289 ± 0.003
correlation = 0.84
y
100
150
200
x
200
250
300
1.0
0.8
0.6
0.4
0.2
0.0

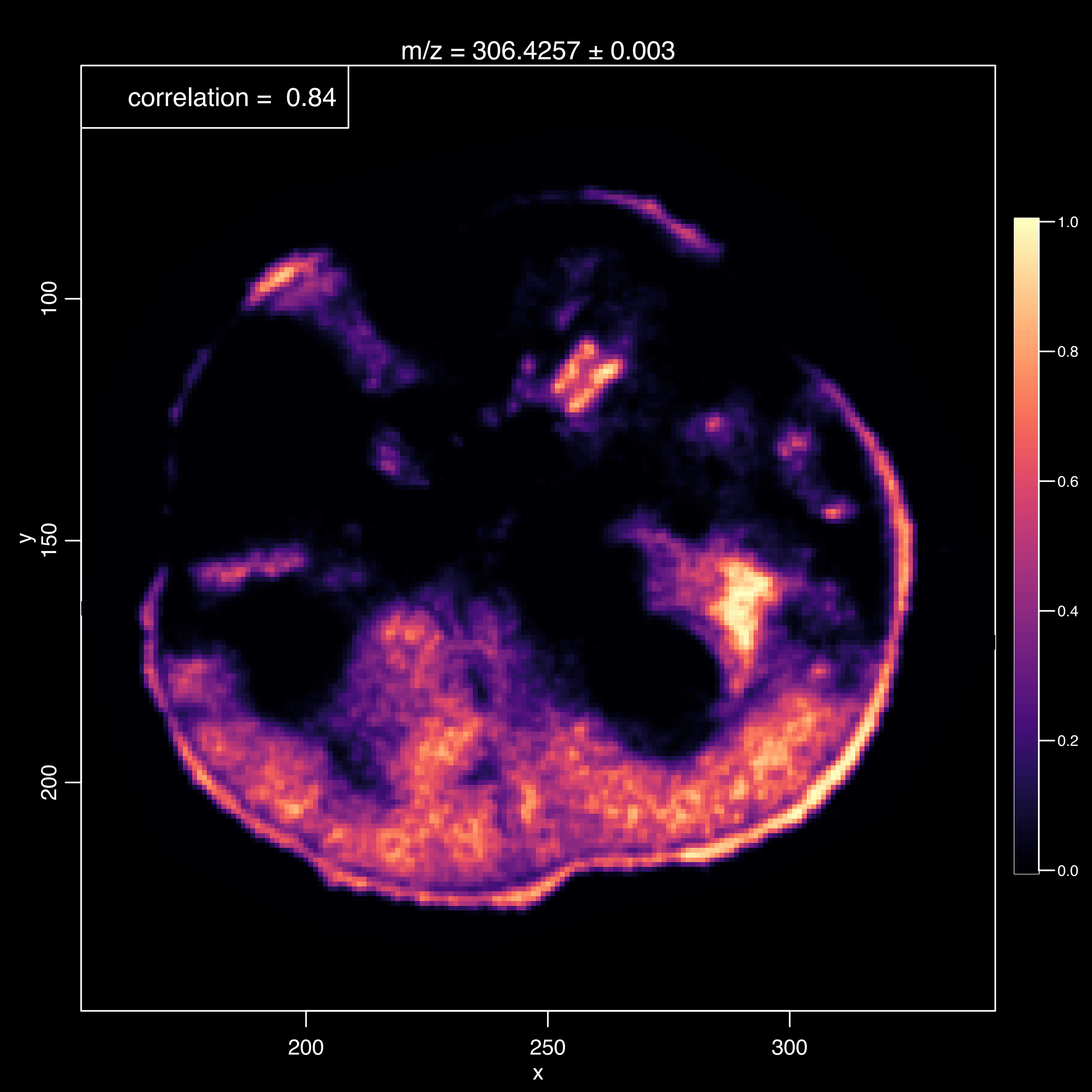
m/z = 306.4257 ± 0.003
correlation = 0.84
y
100
150
200
x
200
250
300
1.0
0.8
0.6
0.4
0.2
0.0

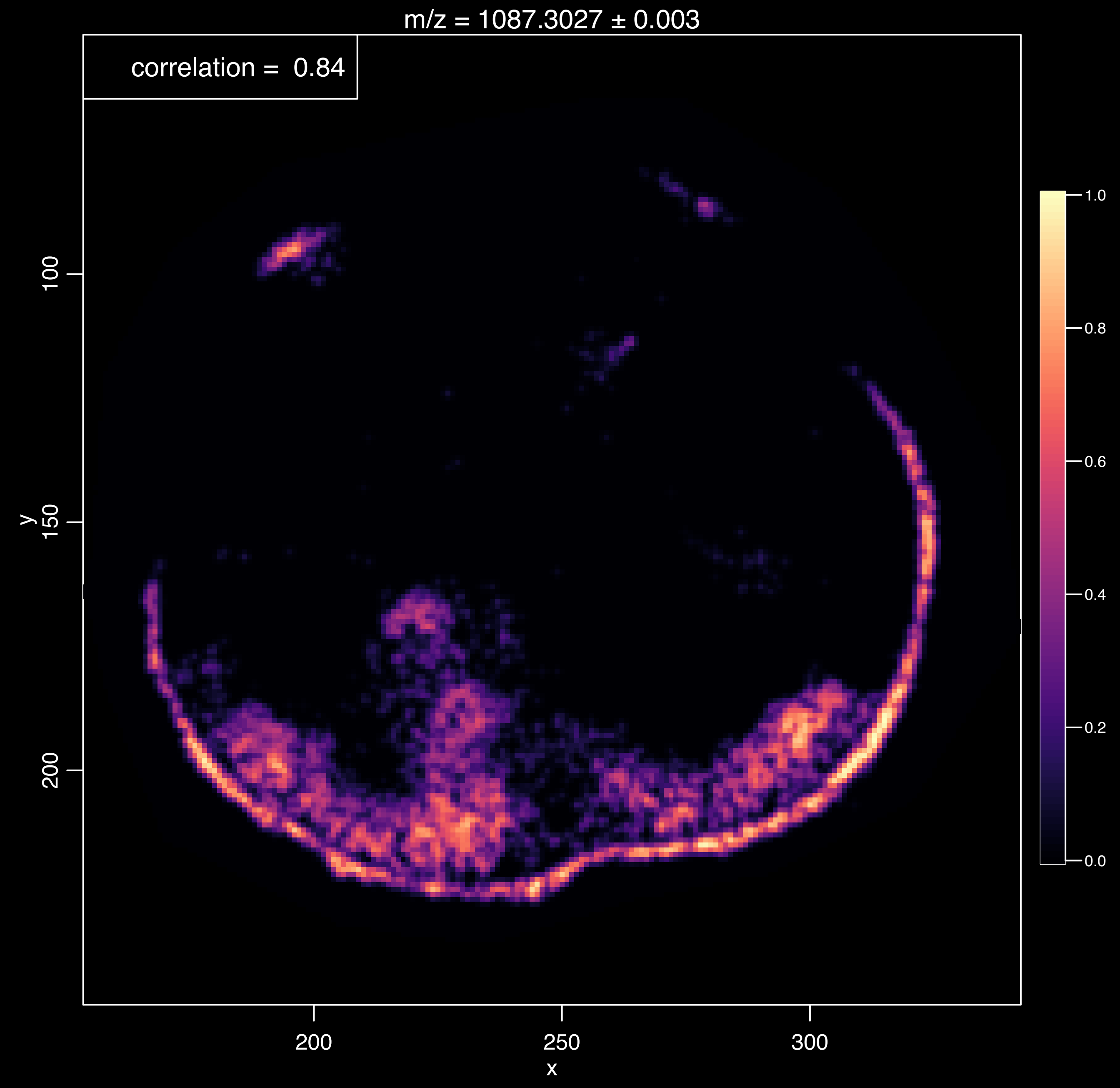
m/z = 1087.3027 ± 0.003
correlation = 0.84
100
150
200
y
200
250
300
x
1.0
0.8
0.6
0.4
0.2
0.0

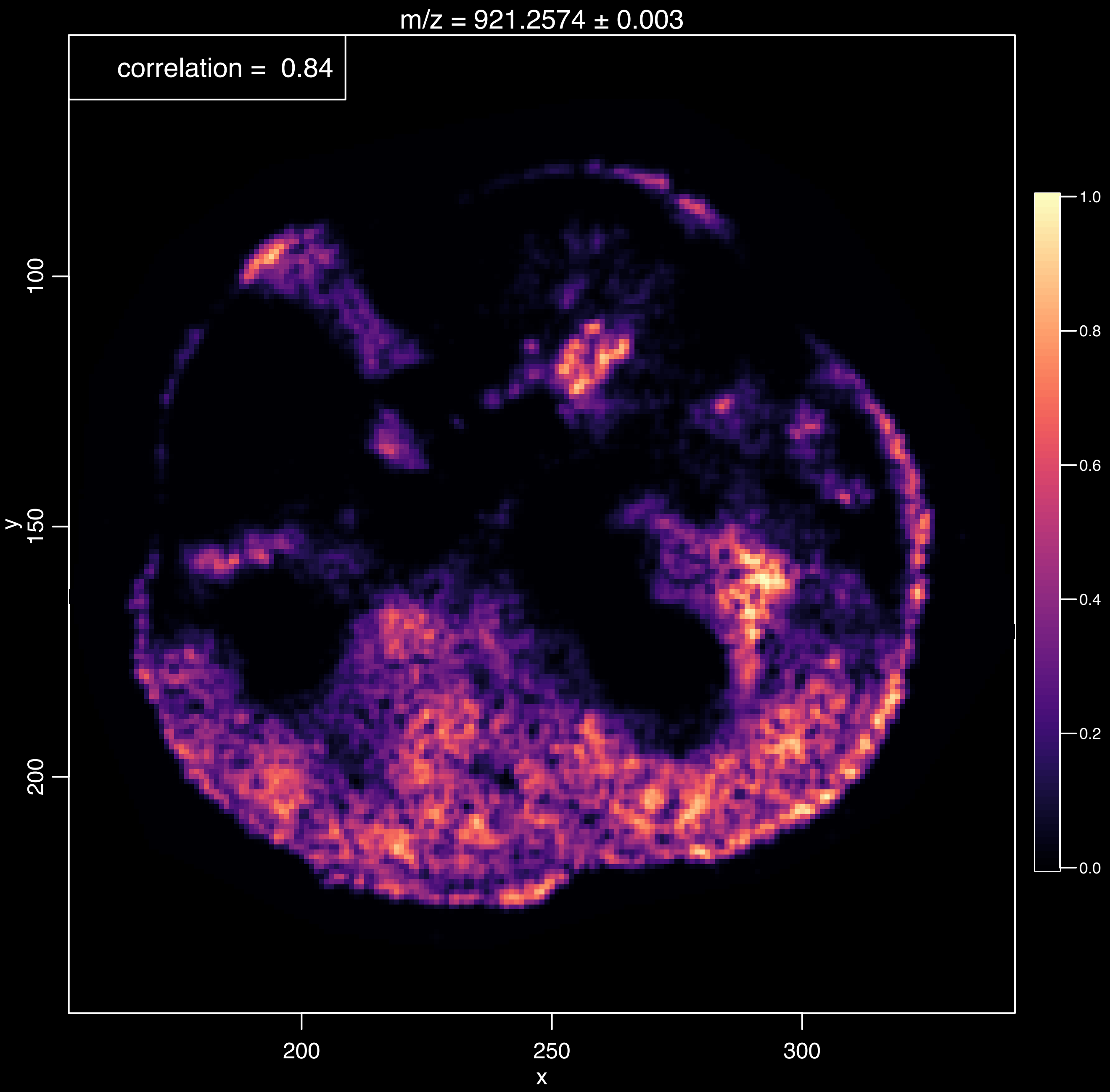
m/z = 921.2574 ± 0.003
correlation = 0.84
100
150
200
y
200
250
300
x
1.0
0.8
0.6
0.4
0.2
0.0

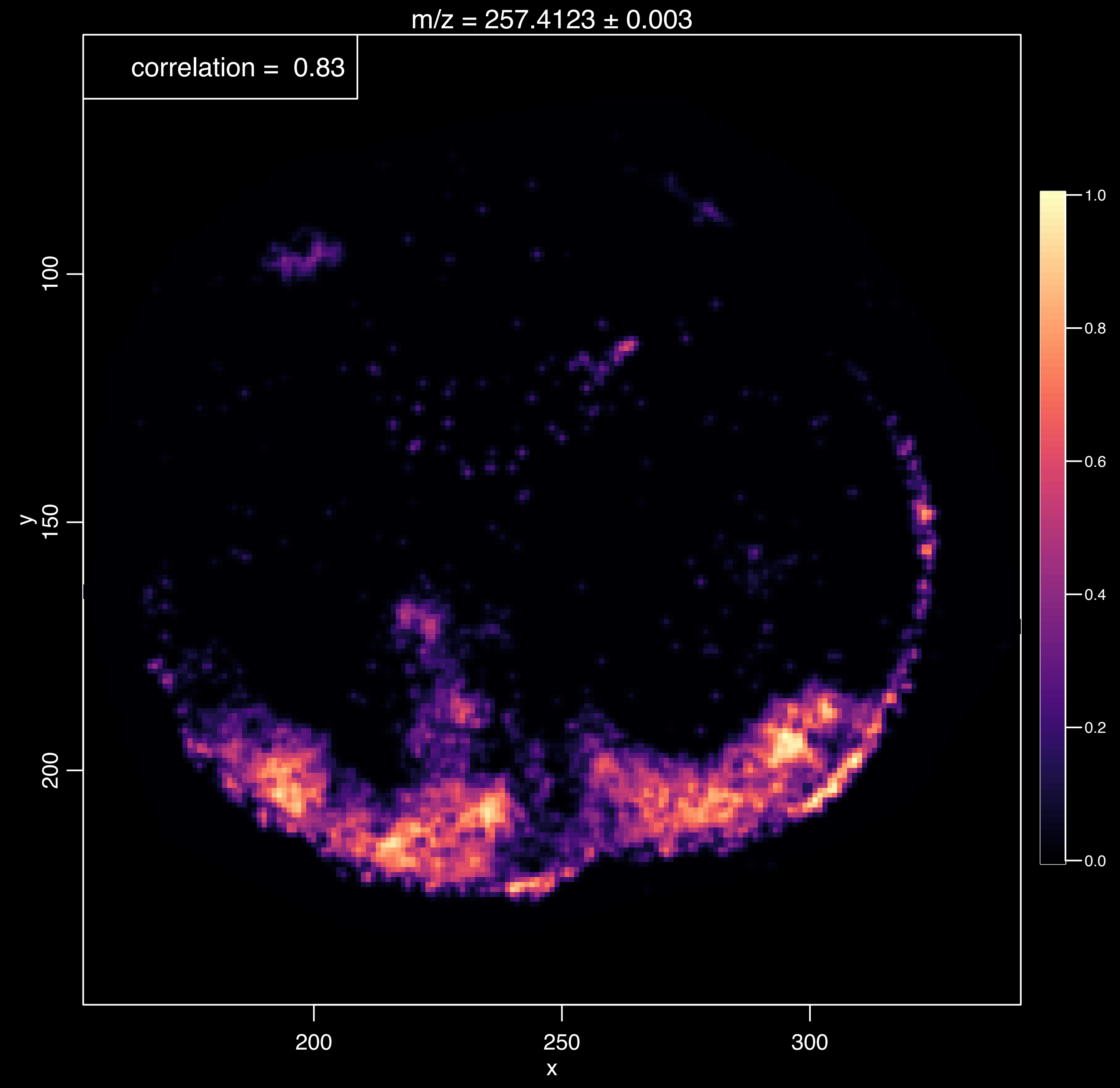

m/z = 257.4123 ± 0.003
correlation = 0.83
y
100
150
200
x
200
250
300
1.0
0.8
0.6
0.4
0.2
0.0

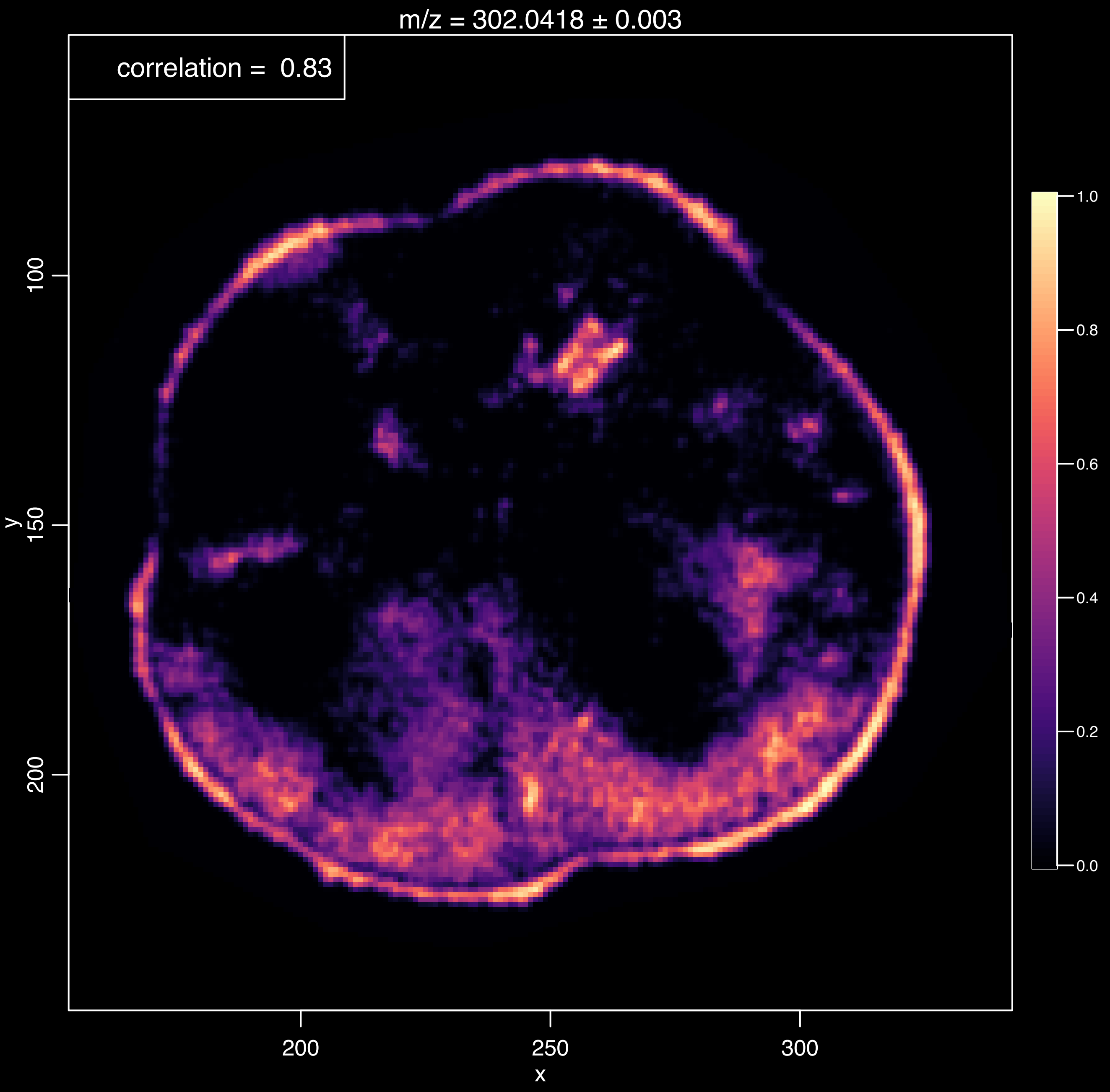

m/z = 302.0418 ± 0.003
correlation = 0.83
100
150
200
y
200
250
300
x
1.0
0.8
0.6
0.4
0.2
0.0

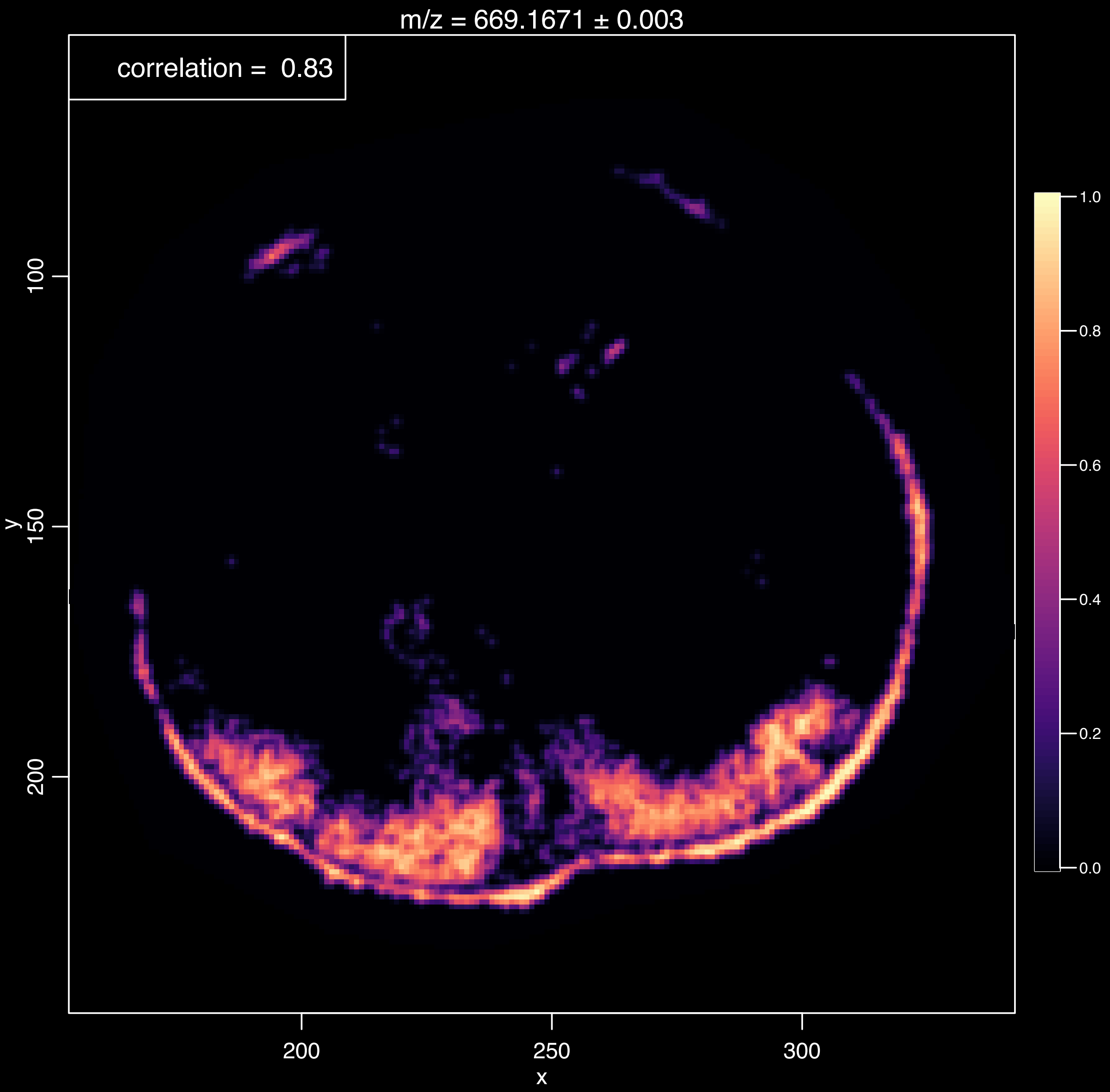
m/z = 669.1671 ± 0.003
correlation = 0.83
y
100
150
200
x
200
250
300
0.0
0.2
0.4
0.6
0.8
1.0

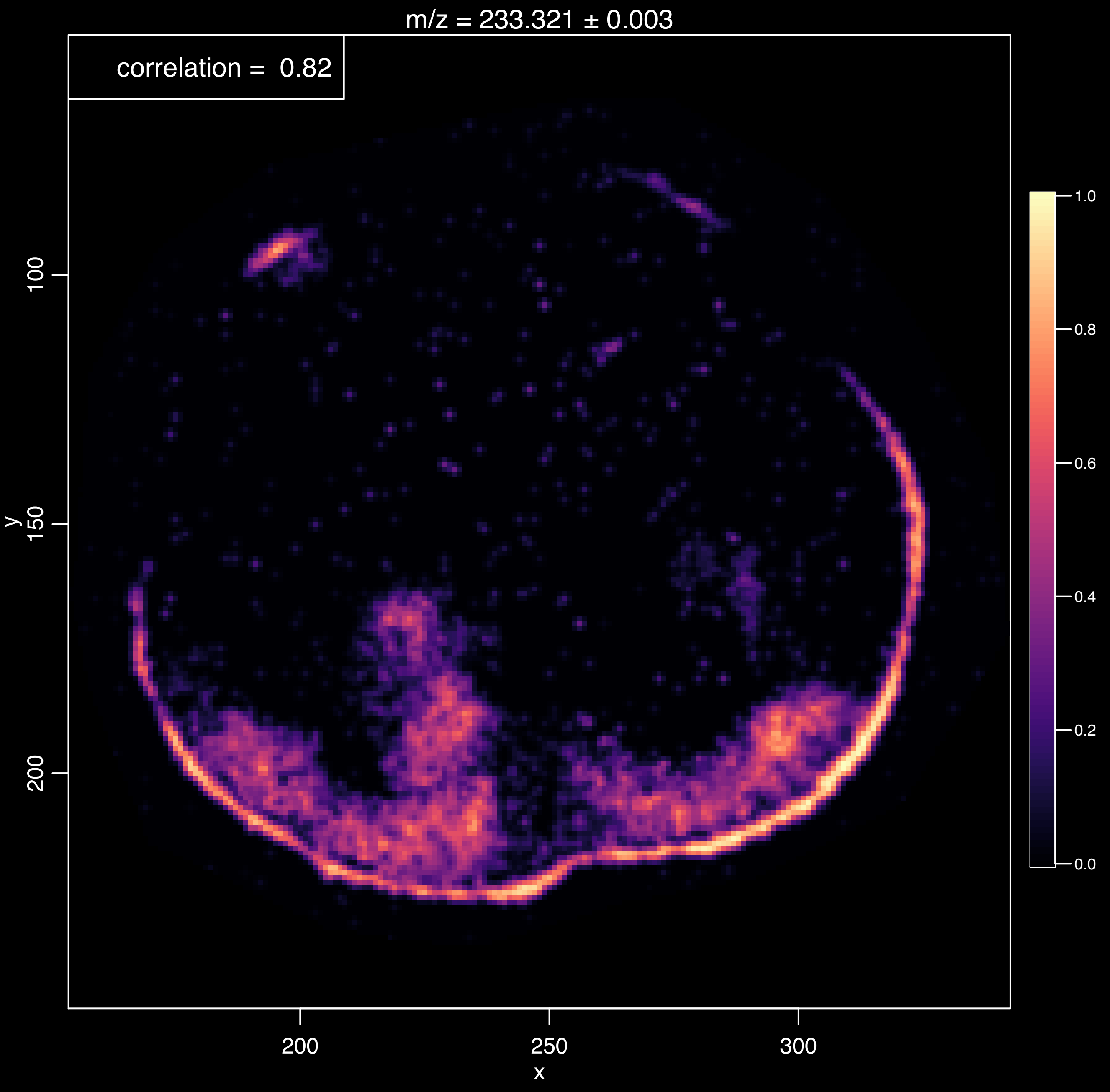

m/z = 233.321 ± 0.003
correlation = 0.82
y
100
150
200
x
200
250
300
1.0
0.8
0.6
0.4
0.2
0.0

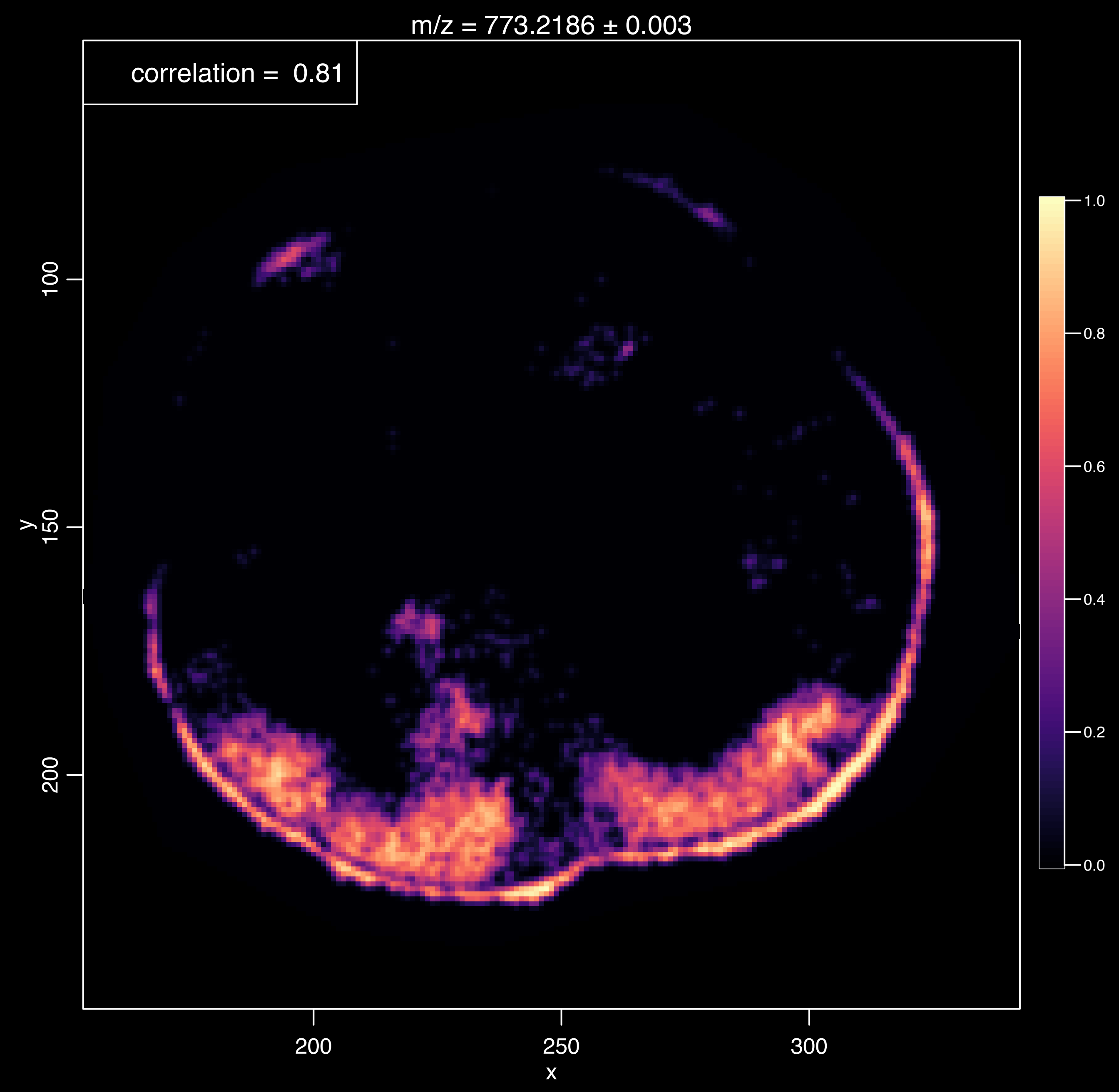
m/z = 773.2186 ± 0.003
correlation = 0.81
y
100
150
200
x
200
250
300
1.0
0.8
0.6
0.4
0.2
0.0

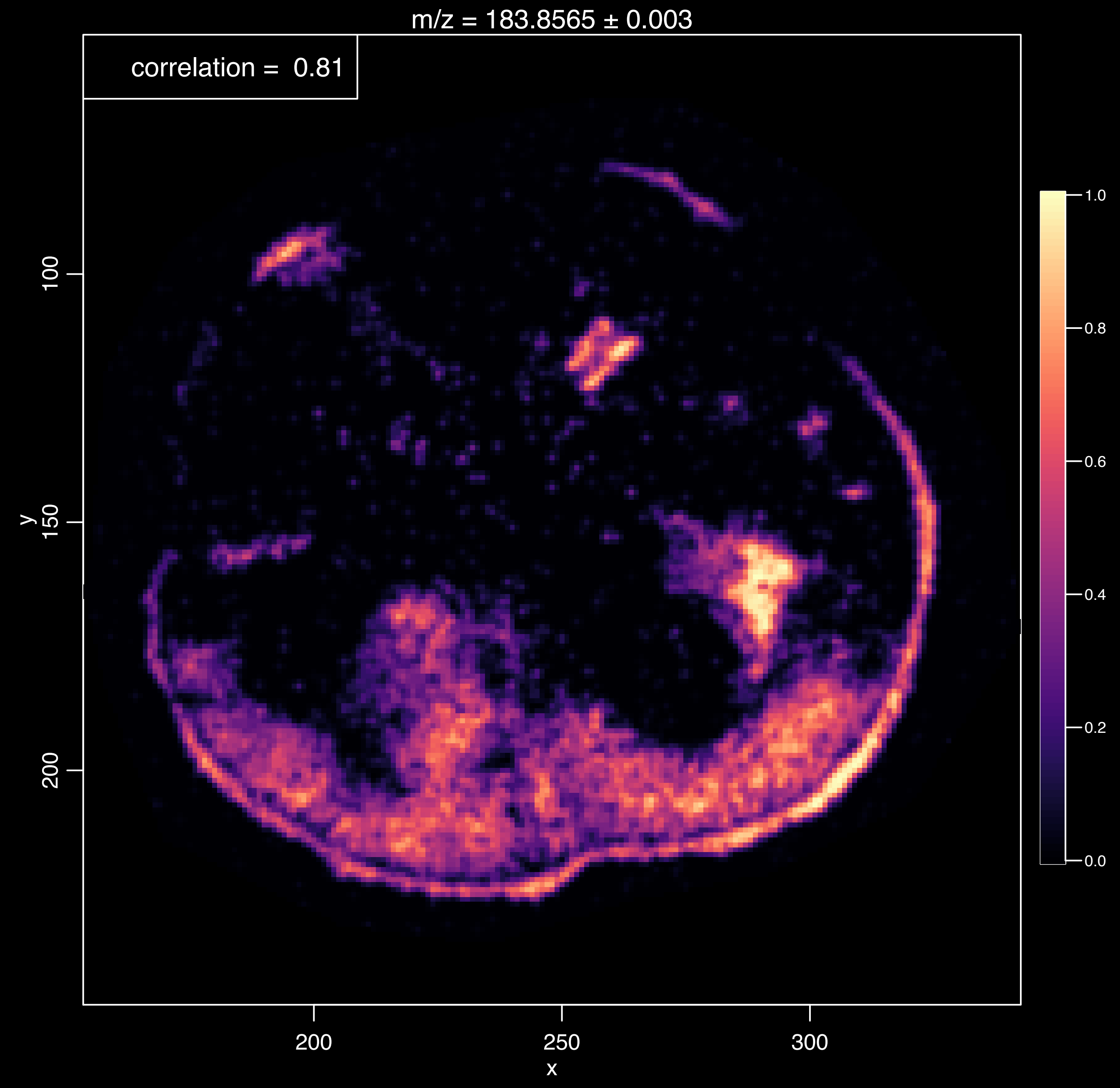

m/z = 183.8565 ± 0.003
correlation = 0.81
y
100
150
200
x
200
250
300
1.0
0.8
0.6
0.4
0.2
0.0

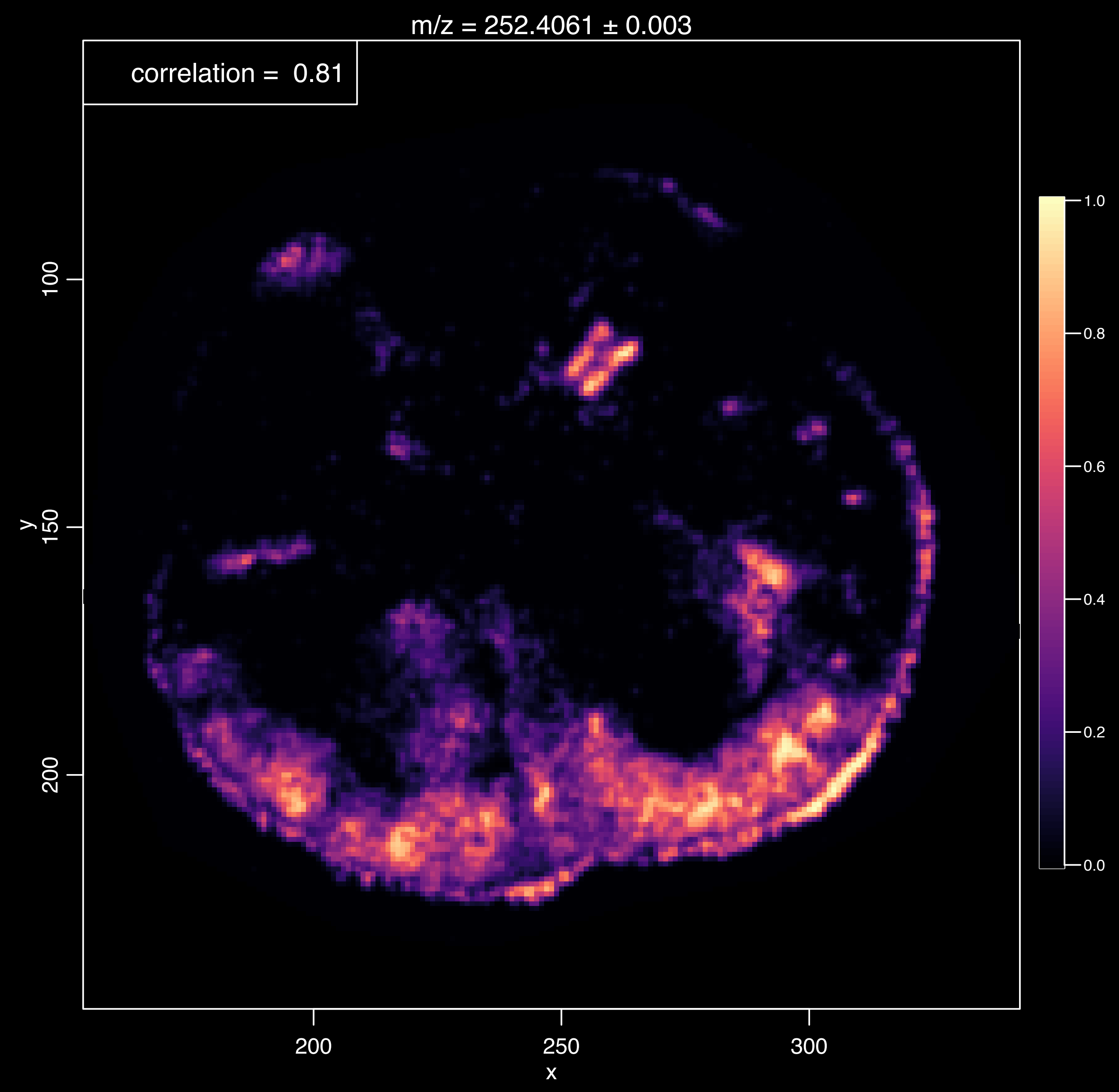

m/z = 252.4061 ± 0.003
correlation = 0.81
100
150
200
y
200
250
300
x
1.0
0.8
0.6
0.4
0.2
0.0

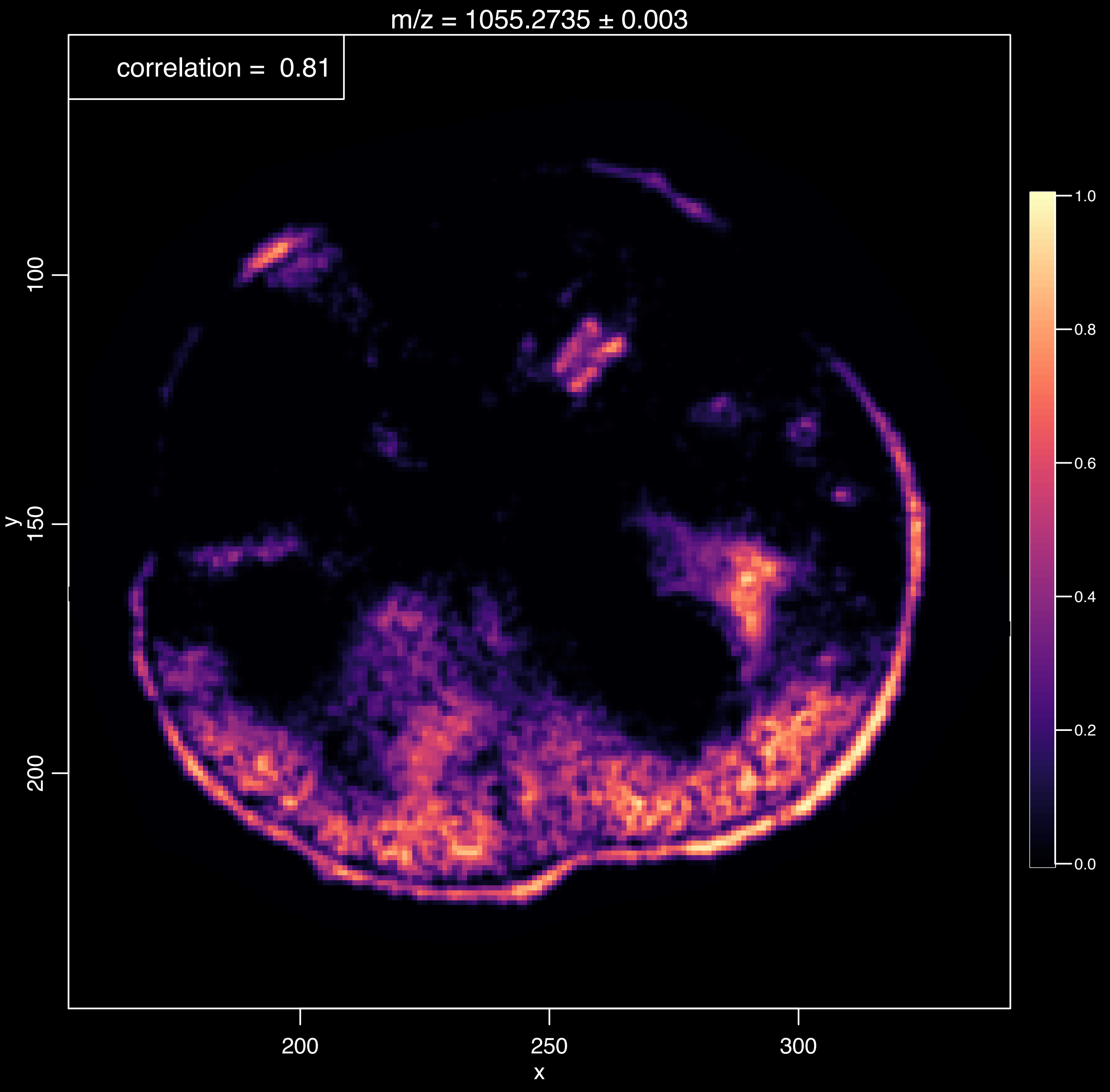

m/z = 1055.2735 ± 0.003
correlation = 0.81
y
100
150
200
x
200
250
300
1.0
0.8
0.6
0.4
0.2
0.0

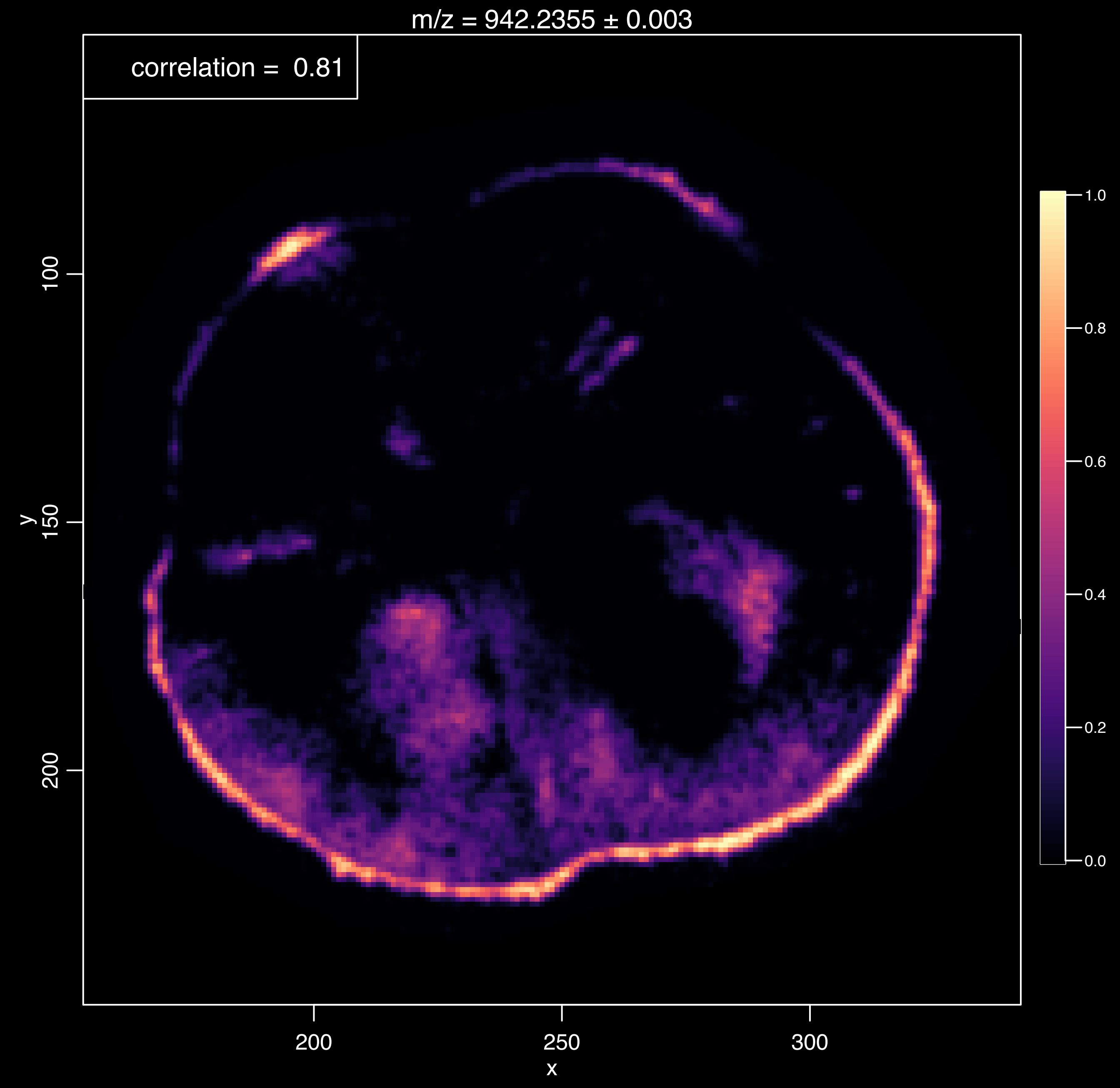
m/z = 942.2355 ± 0.003
correlation = 0.81
y
100
150
200
x
200
250
300
1.0
0.8
0.6
0.4
0.2
0.0

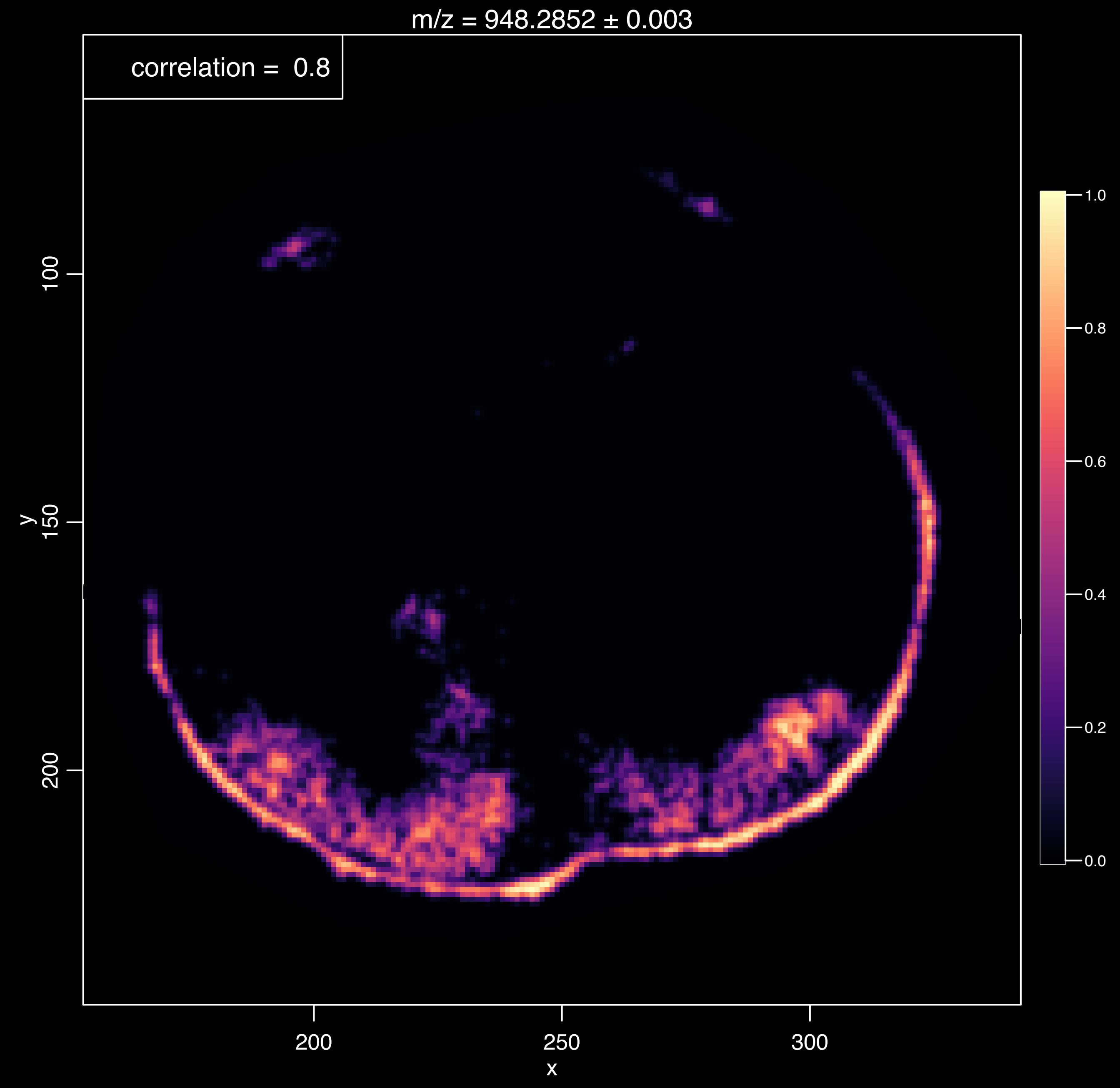
m/z = 948.2852 ± 0.003
correlation = 0.8
100
150
200
y
200
250
300
x
1.0
0.8
0.6
0.4
0.2
0.0

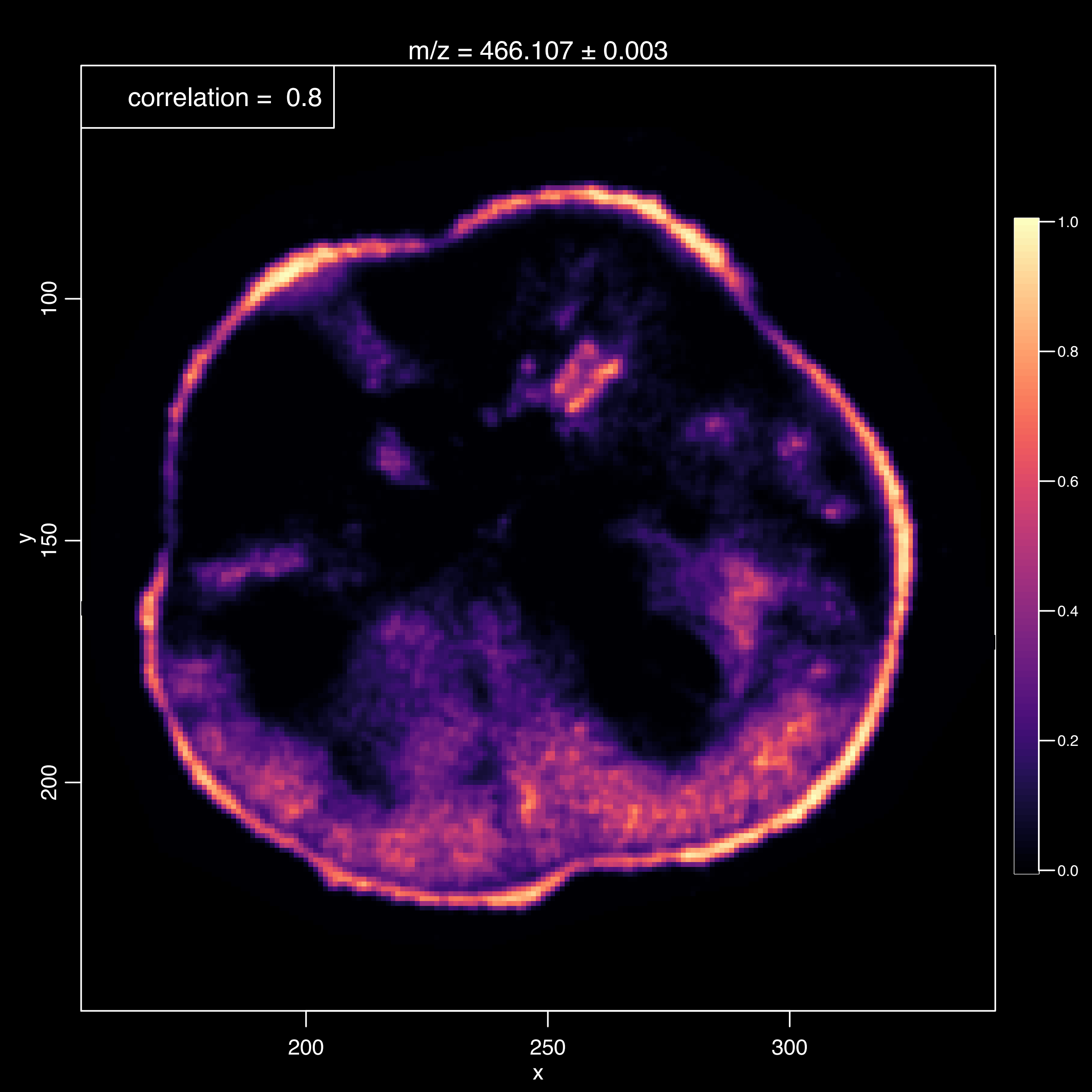
m/z = 466.107 ± 0.003
correlation = 0.8
y
100
150
200
x
200
250
300
1.0
0.8
0.6
0.4
0.2
0.0

Figure S9

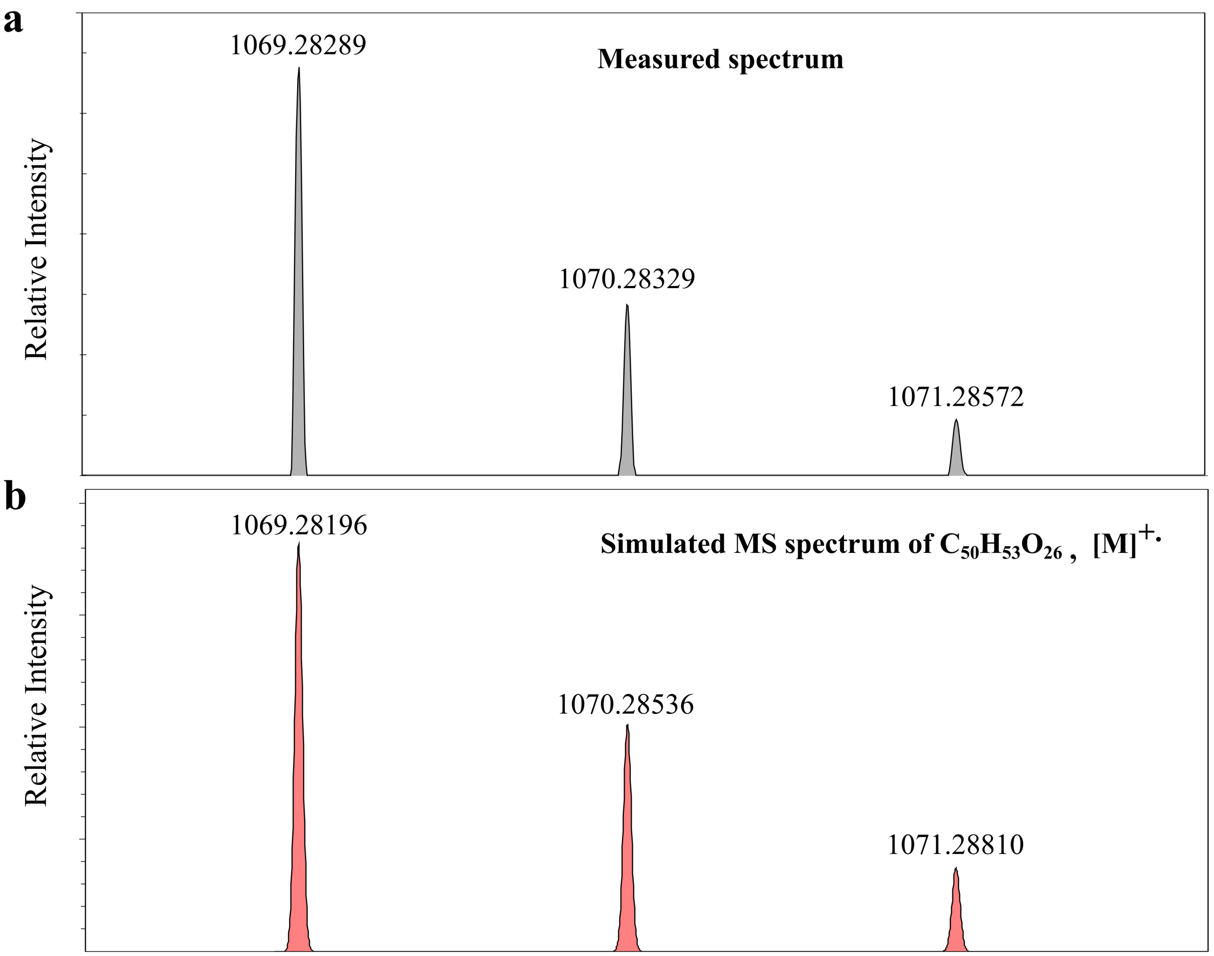

Figure S9 Comparison of ion peak *m/z* 1069.28289 and its ^{13}C isotope peaks with the simulated peaks calculated from the elemental composition $C_{50}H_{53}O_{26}$ ($[M^{+\cdot}]$) at positive ion mode.

Figure S10

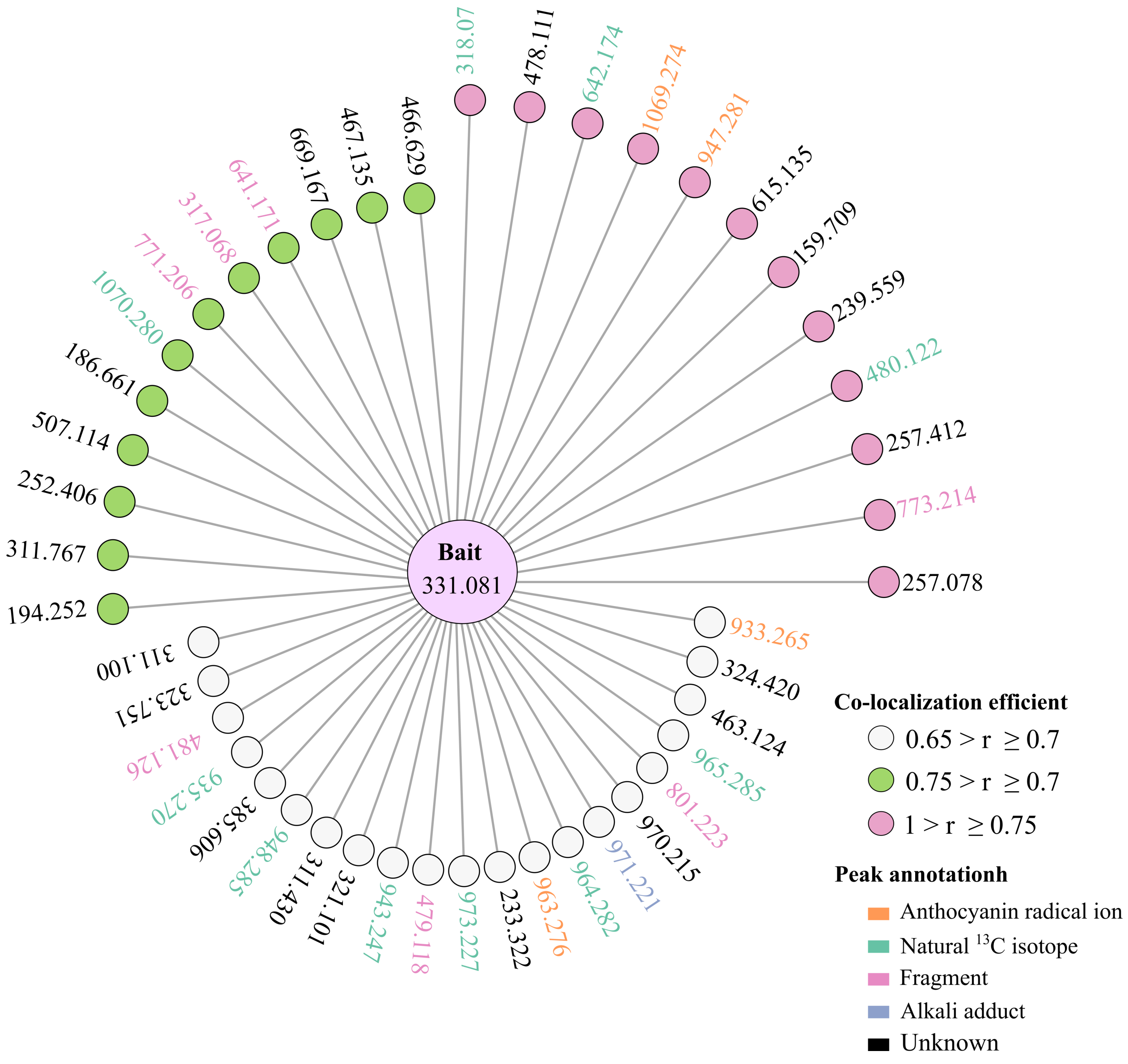

Figure S10 Colocalization networks for anthocyanidin malvidin. (a) Malvidin radical ion ($[M]^{+\cdot}$, *m/z* 331.081) was used as bait, and 45 ions were found colocalized with it. They were classified into 3 groups according to their PCC values, in which 12 ions were with PCC ≥ 0.75 (highlighted by purple solid circles), 12 with 0.75 > PCC ≥ 0.7 (highlighted by green solid circles), and 21 with 0.7 > PCC value ≥ 0.65 (highlighted by white solid circles). These ions were then identified by

inspecting the raw MALDI imaging data and in-parallel LC-MS/MS analysis of tomato skin homogenates. These ions were categorized into anthocyanin radical ions (indicated by orange color), natural ^{13}C isotopes (indicated by green color), fragments (indicated by purple color), alkali adduct (indicated by blue color), and unknown ions (indicated by black color).

Figure S11

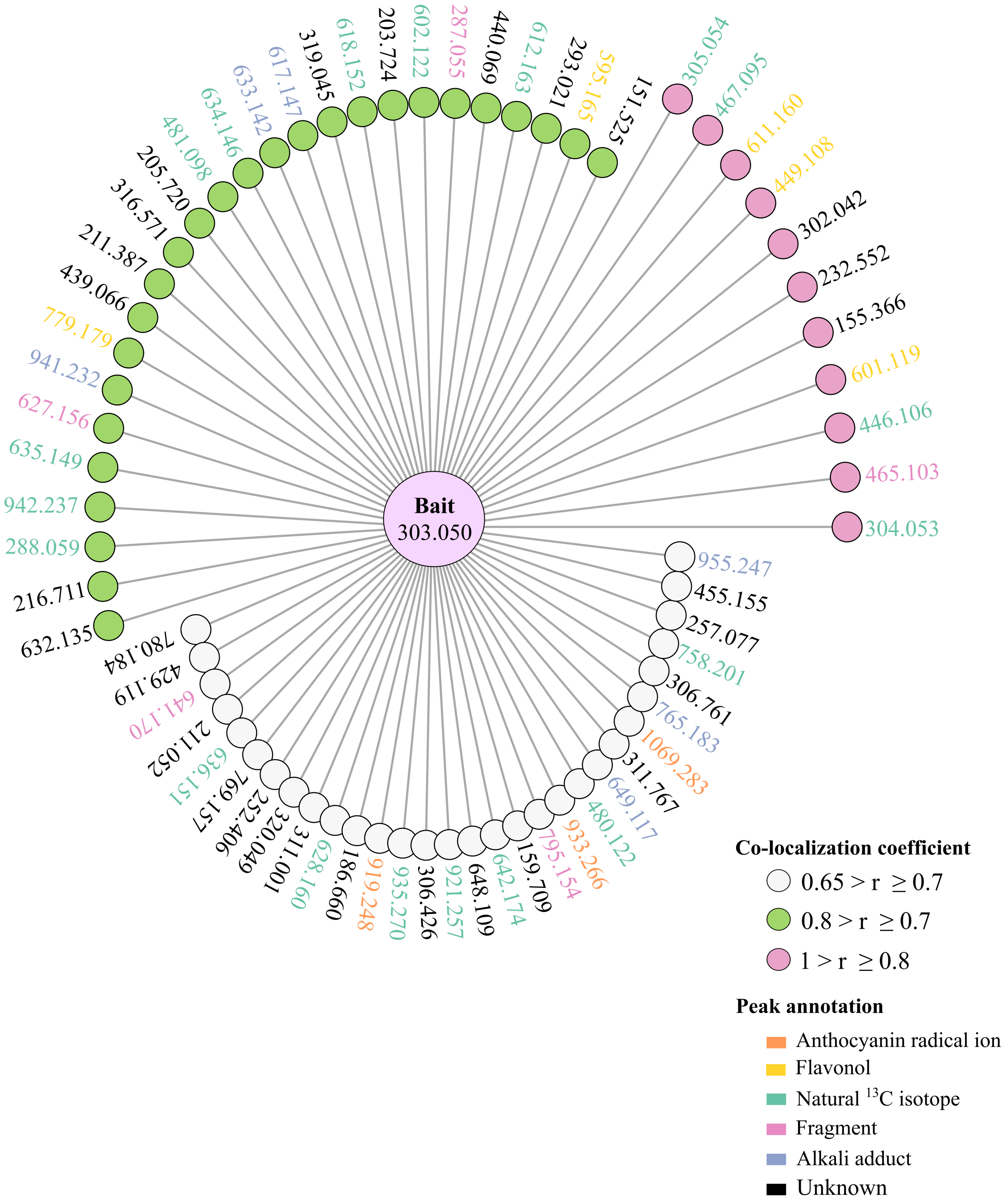
Bait
303.050
305.054
467.095
611.160
449.108
302.042
232.552
155.366
601.119
446.106
465.103
304.053
151.525
595.165
293.021
612.163
440.069
287.055
602.122
203.724
618.152
319.045
617.147
633.142
634.146
481.098
205.720
316.571
211.387
439.066
779.179
941.232
627.156
635.149
942.237
288.059
216.711
632.135
955.247
455.155
257.077
758.201
306.761
765.183
1069.283
311.767
649.117
480.122
933.266
795.154
159.709
642.174
648.109
921.257
306.426
935.270
919.248
186.660
628.160
311.001
320.049
252.406
769.157
636.151
211.052
641.170
429.119
780.184
Co-localization coefficient
0.65 > r ≥ 0.7
0.8 > r ≥ 0.7
1 > r ≥ 0.8
Peak annotation
Anthocyanin radical ion
Flavonol
Natural 13C isotope
Fragment
Alkali adduct
Unknown

Figure S11 Colocalization networks for the anthocyanidin delphinidin. (a) The delphinidin radical ion ($[M]^{+\cdot}$, *m/z* 303.050) was used as bait, and 67 ions were found colocalized with it. They were classified into 3 groups according to their PCC values, in which 11 ions were with PCC ≥ 0.8 (highlighted by purple solid circles), 26 with 0.8 > PCC ≥ 0.7 (highlighted by green solid circles), and 30 with 0.7 > PCC value ≥ 0.65 (highlighted by white solid circles). These ions were then identified by inspecting the raw MALDI imaging data and in-parallel LC-MS/MS analysis of tomato skin homogenates. The ions were categorized into anthocyanin radical ions (indicated by orange color), flavonols (indicated by yellow color), natural ^{13}C isotopes (indicated by green color), fragments (indicated by purple color), alkali adduct (indicated by blue color), and unknown ions (indicated by black color).

Reference

1. Bond NJ, Koulman A, Griffin JL, et al. massPix: an R package for annotation and interpretation of mass spectrometry imaging data for lipidomics. Metabolomics 2017; 13:128
