# Supplementary material for: PICA: Pixel Intensity Correlation Analysis for Deconvolution and Metabolite Identification in Mass Spectrometry Imaging
Source: Anal Chem. 2023 Jan 3;95(2):1652–62. doi: 10.1021/acs.analchem.2c04778 (PMC9850408; doi:10.1021/acs.analchem.2c04778)
Supplement: Supplementary file 2 — ac2c04778_si_002.pdf [file ac2c04778_si_002.pdf]

An Reproducible Example of Colocalization Analysis

Yonghui Dong

(1) Load packages and read data

The dataset is downloaded from Metabolights. Accession number: MTBLS487. The data was obtained in positive ion mode over an m/z range of 300–2000 using a Thermo MALDI LTQ Orbitrap XL instrument (Thermo Fisher Scientific, Bremen, Germany). The spatial resolution was 50 micrometers. For more information about the dataset, readers are kindly referred to the original publication (Hall et al., 2017).

```
## Main package and the version, R version 4.2.0
library(Cardinal)  #(v2.14.0)
library(BiocParallel) #(v1.30.4)
library(plotly) #(v4.10.1)

## Read imzML dataset
path <- paste0("../Data/test_POS", ".imzML")
Brain <- readMSIData(path, resolution = 3, units = "ppm",
                     mass.range = c(300, 2000), attach.only = T)
```

(2) Pre-processing

```
Brain2 <-
  Brain %>%
  normalize(method = "tic") %>%
  peakPick(method = "mad", SNR = 6) %>%
  peakAlign(tolerance = 3, units = "ppm") %>%
  process(BPPARAM = SerialParam())
```

Check the ion image of m/z 844.527 before and after pre-processing in order to make sure that chosen pre-processing parameters are fine.

```
darkmode()
##(2.1) before pre-processing
image(Brain, mz = 844.527, smooth.image = "gaussian", plusminus = 0.03, colorscale = magma,
      contrast.enhance = "suppression", normalize.image = "linear")
```

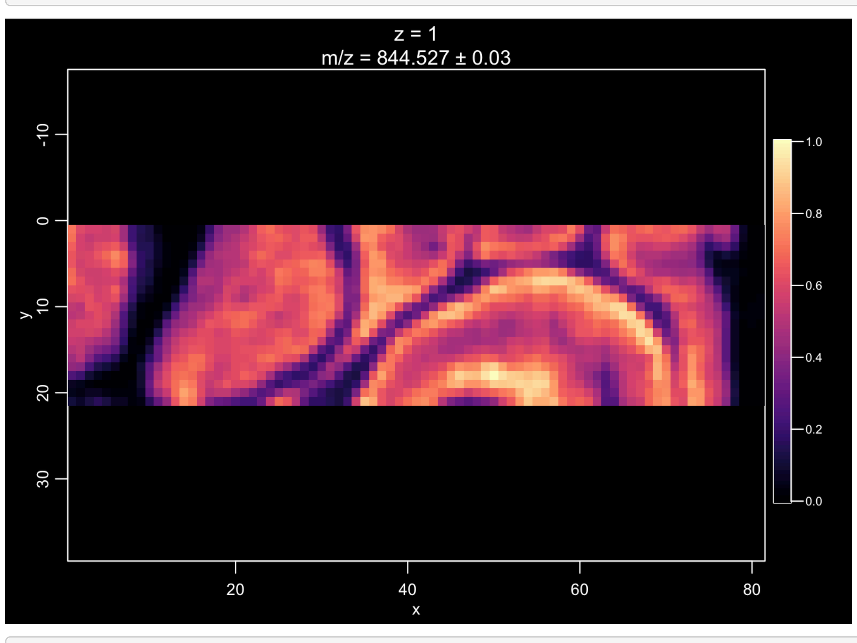


```
##(2.2) after pre-processing
image(Brain2, mz = 844.527, smooth.image = "gaussian", plusminus = 0.03, colorscale = magma,
      contrast.enhance = "suppression", normalize.image = "linear")
```

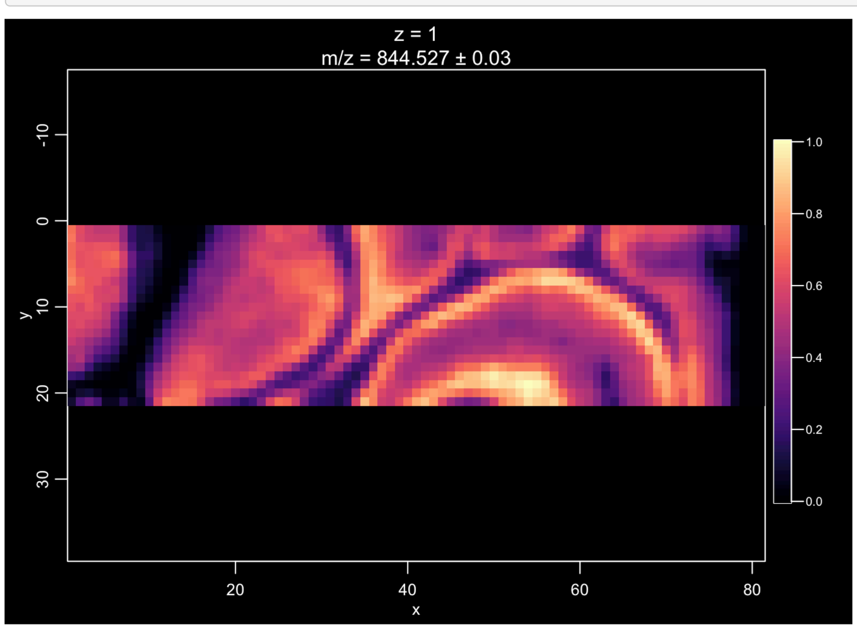

(3) Co-localization

Here first 100 colocalized ions are retained. Three colocalization measures were calculated. `correlation` is Pearson's correlation, `M1` is match score, and `M2` is Manders' colocalization coefficients. Here Pearson's correlation is used.

```
#(3.1) co-localization PC36:1
coloc_826 <- colocalized(Brain2, mz= 826.572, n = 100, BPPARAM = SerialParam())
#(3.2) co-localization PC38:6
coloc_844 <- colocalized(Brain2, mz= 844.525, n = 100, BPPARAM = SerialParam())
#(3.3) co-localization PC40:6
coloc_872 <- colocalized(Brain2, mz= 872.557, n = 100, BPPARAM = SerialParam())
```

(4) Extract in-source fragments, and create pseudo MS/MS spectrum

```
#(4.1) PC36:1
mycoloc <- as.data.frame(coloc_826)
MSe <- mycoloc[mycoloc$correlation >= 0.90,]
## extract intensity
int = as.vector(rep(NA, dim(MSe)[1]))
for (i in 1:length(MSe$mz)) {
  int[i] <- sum(spectra(Brain2)[features(Brain2,  mz = MSe$mz[i]),])
}
## interactive plot
spec = cbind.data.frame(mz = MSe$mz, Int = int)
p = ggplot(spec, aes(x = mz, ymax = Int/max(Int)*100, ymin = 0, colour = "red")) +
  geom_linerange() +
  scale_y_continuous(expand = c(0, 0), limits = c(0, 100*1.1)) +
  theme_bw() +
  ggtitle("m/z 826.577")
ggplotly(p)
```

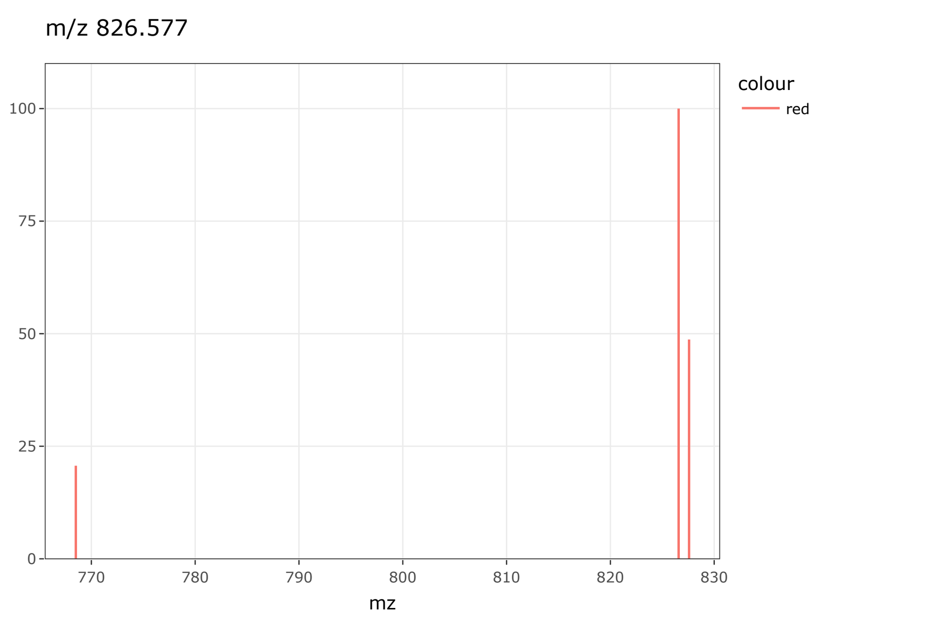


```
#(4.2) PC38:6
mycoloc <- as.data.frame(coloc_844)
MSe <- mycoloc[mycoloc$correlation >= 0.90,]
## extract intensity
int = as.vector(rep(NA, dim(MSe)[1]))
for (i in 1:length(MSe$mz)) {
  int[i] <- sum(spectra(Brain2)[features(Brain2,  mz = MSe$mz[i]),])
}
## interactive plot
spec = cbind.data.frame(mz = MSe$mz, Int = int)
p = ggplot(spec, aes(x = mz, ymax = Int/max(Int)*100, ymin = 0, colour = "red")) +
  geom_linerange() +
  scale_y_continuous(expand = c(0, 0), limits = c(0, 100*1.1)) +
  theme_bw() +
  ggtitle("m/z 844.528")
ggplotly(p)
```

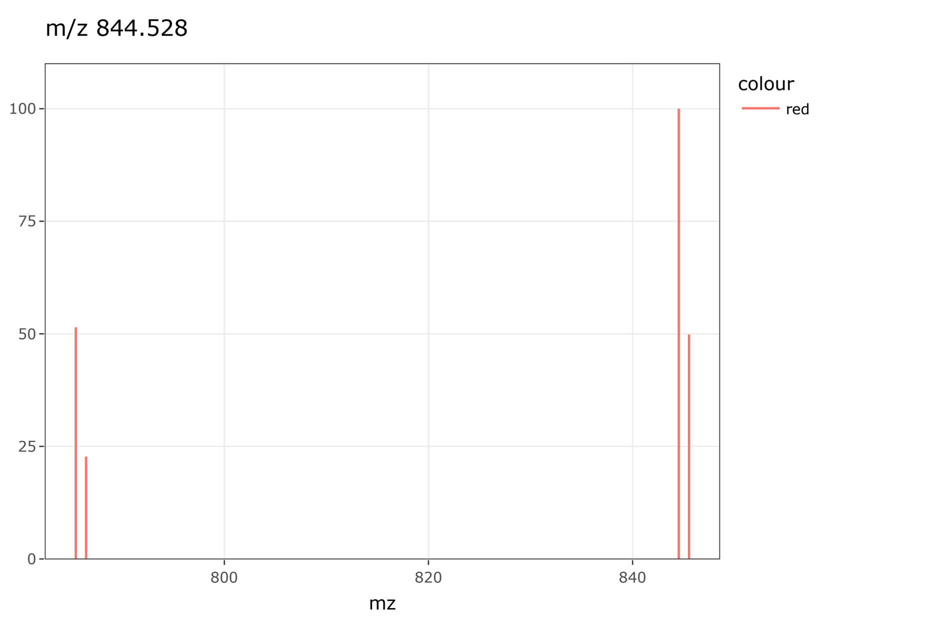


```
#(4.3) PC40:6
mycoloc <- as.data.frame(coloc_872)
MSe <- mycoloc[mycoloc$correlation >= 0.90,]
## extract intensity
int = as.vector(rep(NA, dim(MSe)[1]))
for (i in 1:length(MSe$mz)) {
  int[i] <- sum(spectra(Brain2)[features(Brain2,  mz = MSe$mz[i]),])
}
## interactive plot
spec = cbind.data.frame(mz = MSe$mz, Int = int)
p = ggplot(spec, aes(x = mz, ymax = Int/max(Int)*100, ymin = 0, colour = "red")) +
  geom_linerange() +
  scale_y_continuous(expand = c(0, 0), limits = c(0, 100*1.1)) +
  theme_bw()+
  ggtitle("m/z 872.559")
ggplotly(p)
```

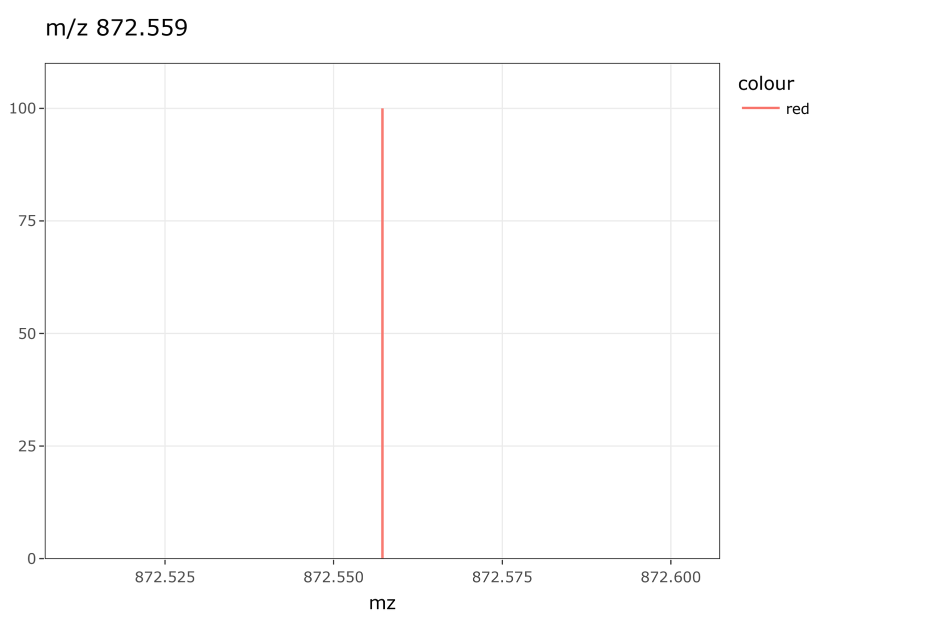

(5) Plot MALDI images

```
#(5.1) PC36:1
mycoloc <- as.data.frame(coloc_826)
MSe <- mycoloc[mycoloc$correlation >= 0.90,]
pdf(file = file.path("Result/colocalization_826.pdf"), onefile = TRUE)
for(i in 1:dim(MSe)[1]){
  darkmode()
  print(image(Brain2, mz = MSe$mz[i], smooth.image = "gaussian",
              plusminus = 0.003, colorscale=magma,
              contrast.enhance="suppression", normalize.image = "linear"))
  legend("topleft", legend= paste("correlation = ", round(MSe$correlation[i], 2)))
}
dev.off()

#(5.2) PC38:6
mycoloc <- as.data.frame(coloc_844)
MSe <- mycoloc[mycoloc$correlation >= 0.90,]
pdf(file = file.path("Result/colocalization_844.pdf"), onefile = TRUE)
for(i in 1:dim(MSe)[1]){
  darkmode()
  print(image(Brain2, mz = MSe$mz[i], smooth.image = "gaussian",
              plusminus = 0.003, colorscale=magma,
              contrast.enhance="suppression", normalize.image = "linear"))
  legend("topleft", legend= paste("correlation = ", round(MSe$correlation[i], 2)))
}
dev.off()

#(5.3) PC38:6
mycoloc <- as.data.frame(coloc_872)
MSe <- mycoloc[mycoloc$correlation >= 0.90,]
pdf(file = file.path("Result/colocalization_872.pdf"), onefile = TRUE)
for(i in 1:dim(MSe)[1]){
  darkmode()
  print(image(Brain2, mz = MSe$mz[i], smooth.image = "gaussian",
              plusminus = 0.003, colorscale=magma,
              contrast.enhance="suppression", normalize.image = "linear"))
  legend("topleft", legend= paste("correlation = ", round(MSe$correlation[i], 2)))
}
dev.off()
```

Session information

```
sessioninfo::session_info()
```

```
## ─ Session info ───────────────────────────────────────────────────────────────
##  setting  value
##  version  R version 4.2.0 (2022-04-22)
##  os       macOS Big Sur/Monterey 10.16
##  system   x86_64, darwin17.0
##  ui       X11
##  language (EN)
##  collate  en_US.UTF-8
##  ctype    en_US.UTF-8
##  tz       Asia/Jerusalem
##  date     2022-12-10
##  pandoc   2.19.2 @ /Applications/RStudio.app/Contents/MacOS/quarto/bin/tools/ (via rmarkdown)
##
## ─ Packages ───────────────────────────────────────────────────────────────────
##  package      * version  date (UTC) lib source
##  abind          1.4-5    2016-07-21 [1] CRAN (R 4.2.0)
##  assertthat     0.2.1    2019-03-21 [1] CRAN (R 4.2.0)
##  biglm          0.9-2.1  2020-11-27 [1] CRAN (R 4.2.0)
##  Biobase        2.56.0   2022-04-26 [1] Bioconductor
##  BiocGenerics * 0.42.0   2022-04-26 [1] Bioconductor
##  BiocParallel * 1.30.4   2022-10-13 [1] Bioconductor
##  bitops         1.0-7    2021-04-24 [1] CRAN (R 4.2.0)
##  bslib          0.4.1    2022-11-02 [1] CRAN (R 4.2.0)
##  cachem         1.0.6    2021-08-19 [1] CRAN (R 4.2.0)
##  Cardinal     * 2.14.0   2022-04-26 [1] Bioconductor
##  cli            3.4.1    2022-09-23 [1] CRAN (R 4.2.0)
##  codetools      0.2-18   2020-11-04 [1] CRAN (R 4.2.0)
##  colorspace     2.0-3    2022-02-21 [1] CRAN (R 4.2.0)
##  crosstalk      1.2.0    2021-11-04 [1] CRAN (R 4.2.0)
##  data.table     1.14.6   2022-11-16 [1] CRAN (R 4.2.0)
##  DBI            1.1.3    2022-06-18 [1] CRAN (R 4.2.0)
##  digest         0.6.30   2022-10-18 [1] CRAN (R 4.2.0)
##  dplyr          1.0.10   2022-09-01 [1] CRAN (R 4.2.0)
##  EBImage      * 4.38.0   2022-05-15 [1] Bioconductor
##  ellipsis       0.3.2    2021-04-29 [1] CRAN (R 4.2.0)
##  evaluate       0.18     2022-11-07 [1] CRAN (R 4.2.0)
##  fansi          1.0.3    2022-03-24 [1] CRAN (R 4.2.0)
##  farver         2.1.1    2022-07-06 [1] CRAN (R 4.2.0)
##  fastmap        1.1.0    2021-01-25 [1] CRAN (R 4.2.0)
##  fftwtools      0.9-11   2021-03-01 [1] CRAN (R 4.2.0)
##  generics       0.1.3    2022-07-05 [1] CRAN (R 4.2.0)
##  ggplot2      * 3.3.6    2022-05-03 [1] CRAN (R 4.2.0)
##  glue           1.6.2    2022-02-24 [1] CRAN (R 4.2.0)
##  gtable         0.3.1    2022-09-01 [1] CRAN (R 4.2.0)
##  highr          0.9      2021-04-16 [1] CRAN (R 4.2.0)
##  htmltools      0.5.3    2022-07-18 [1] CRAN (R 4.2.0)
##  htmlwidgets    1.5.4    2021-09-08 [1] CRAN (R 4.2.0)
##  httr           1.4.4    2022-08-17 [1] CRAN (R 4.2.0)
##  irlba          2.3.5.1  2022-10-03 [1] CRAN (R 4.2.0)
##  jpeg           0.1-9    2021-07-24 [1] CRAN (R 4.2.0)
##  jquerylib      0.1.4    2021-04-26 [1] CRAN (R 4.2.0)
##  jsonlite       1.8.3    2022-10-21 [1] CRAN (R 4.2.0)
##  knitr          1.41     2022-11-18 [1] CRAN (R 4.2.0)
##  labeling       0.4.2    2020-10-20 [1] CRAN (R 4.2.0)
##  lattice        0.20-45  2021-09-22 [1] CRAN (R 4.2.0)
##  lazyeval       0.2.2    2019-03-15 [1] CRAN (R 4.2.0)
##  lifecycle      1.0.3    2022-10-07 [1] CRAN (R 4.2.0)
##  locfit         1.5-9.6  2022-07-11 [1] CRAN (R 4.2.0)
##  magrittr       2.0.3    2022-03-30 [1] CRAN (R 4.2.0)
##  MASS           7.3-58.1 2022-08-03 [1] CRAN (R 4.2.0)
##  Matrix         1.5-3    2022-11-11 [1] CRAN (R 4.2.0)
##  matter         1.22.0   2022-04-26 [1] Bioconductor
##  mclust         6.0.0    2022-10-31 [1] CRAN (R 4.2.0)
##  munsell        0.5.0    2018-06-12 [1] CRAN (R 4.2.0)
##  nlme           3.1-160  2022-10-10 [1] CRAN (R 4.2.0)
##  pillar         1.8.1    2022-08-19 [1] CRAN (R 4.2.0)
##  pkgconfig      2.0.3    2019-09-22 [1] CRAN (R 4.2.0)
##  plotly       * 4.10.1   2022-11-07 [1] CRAN (R 4.2.0)
##  png            0.1-7    2013-12-03 [1] CRAN (R 4.2.0)
##  ProtGenerics * 1.28.0   2022-04-26 [1] Bioconductor
##  purrr          0.3.5    2022-10-06 [1] CRAN (R 4.2.0)
##  R6             2.5.1    2021-08-19 [1] CRAN (R 4.2.0)
##  RCurl          1.98-1.9 2022-10-03 [1] CRAN (R 4.2.0)
##  rlang          1.0.6    2022-09-24 [1] CRAN (R 4.2.0)
##  rmarkdown      2.18     2022-11-09 [1] CRAN (R 4.2.0)
##  rstudioapi     0.14     2022-08-22 [1] CRAN (R 4.2.0)
##  S4Vectors    * 0.34.0   2022-04-26 [1] Bioconductor
##  sass           0.4.4    2022-11-24 [1] CRAN (R 4.2.0)
##  scales         1.2.1    2022-08-20 [1] CRAN (R 4.2.0)
##  sessioninfo    1.2.2    2021-12-06 [1] CRAN (R 4.2.0)
##  signal         0.7-7    2021-05-25 [1] CRAN (R 4.2.0)
##  sp             1.5-1    2022-11-07 [1] CRAN (R 4.2.0)
##  stringi        1.7.8    2022-07-11 [1] CRAN (R 4.2.0)
##  stringr        1.4.1    2022-08-20 [1] CRAN (R 4.2.0)
##  tibble         3.1.8    2022-07-22 [1] CRAN (R 4.2.0)
##  tidyr          1.2.1    2022-09-08 [1] CRAN (R 4.2.0)
##  tidyselect     1.2.0    2022-10-10 [1] CRAN (R 4.2.0)
##  tiff           0.1-11   2022-01-31 [1] CRAN (R 4.2.0)
##  utf8           1.2.2    2021-07-24 [1] CRAN (R 4.2.0)
##  vctrs          0.5.1    2022-11-16 [1] CRAN (R 4.2.0)
##  viridisLite    0.4.1    2022-08-22 [1] CRAN (R 4.2.0)
##  withr          2.5.0    2022-03-03 [1] CRAN (R 4.2.0)
##  xfun           0.35     2022-11-16 [1] CRAN (R 4.2.0)
##  yaml           2.3.6    2022-10-18 [1] CRAN (R 4.2.0)
##
##  [1] /Library/Frameworks/R.framework/Versions/4.2/Resources/library
##
## ──────────────────────────────────────────────────────────────────────────────
```
